# A high-quality chromosomal genome assembly of the sea cucumber *Chiridota heheva* and its hydrothermal adaptation

**DOI:** 10.1093/gigascience/giad107

**Published:** 2024-01-04

**Authors:** Yujin Pu, Yang Zhou, Jun Liu, Haibin Zhang

**Affiliations:** Institute of Deep-sea Science and Engineering, Chinese Academy of Sciences, Sanya 572000, China; University of Chinese Academy of Sciences, Beijing 100049, China; Institute of Deep-sea Science and Engineering, Chinese Academy of Sciences, Sanya 572000, China; Institute of Deep-sea Science and Engineering, Chinese Academy of Sciences, Sanya 572000, China; Institute of Deep-sea Science and Engineering, Chinese Academy of Sciences, Sanya 572000, China

**Keywords:** *Chiridota heheva*, Hi-C, positively selected gene, gene family, unique gene

## Abstract

**Background:**

*Chiridota heheva* is a cosmopolitan holothurian well adapted to diverse deep-sea ecosystems, especially chemosynthetic environments. Besides high hydrostatic pressure and limited light, high concentrations of metal ions also represent harsh conditions in hydrothermal environments. Few holothurian species can live in such extreme conditions. Therefore, it is valuable to elucidate the adaptive genetic mechanisms of *C. heheva* in hydrothermal environments.

**Findings:**

Herein, we report a high-quality reference genome assembly of *C. heheva* from the Kairei vent, which is the first chromosome-level genome of Apodida. The chromosome-level genome size was 1.43 Gb, with a scaffold N50 of 53.24 Mb and BUSCO completeness score of 94.5%. Contig sequences were clustered, ordered, and assembled into 19 natural chromosomes. Comparative genome analysis found that the expanded gene families and positively selected genes of *C. heheva* were involved in the DNA damage repair process. The expanded gene families and the unique genes contributed to maintaining iron homeostasis in an iron-enriched environment. The positively selected gene *RFC2* with 10 positively selected sites played an essential role in DNA repair under extreme environments.

**Conclusions:**

This first chromosome-level genome assembly of *C. heheva* reveals the hydrothermal adaptation of holothurians. As the first chromosome-level genome of order Apodida, this genome will provide the resource for investigating the evolution of class Holothuroidea.

## Data Description

### Context

Hydrothermal vents are one of the typical deep-sea chemosynthetically driven ecosystems with a wide array of animals and chemosynthetic microbes. The hydrothermal vent environment is characterized by rapid changes in temperature, acidic pH, sulfur compounds, metal, methane, hydrogen, carbon dioxide, and other toxic chemistry, besides high hydrostatic pressure and darkness of the deep sea [1–9]. However, these inhospitable environments have been reported as crucial enrichment areas for deep-sea life.

Hydrothermal habitat fauna commonly adapt to the unusual environment with the uncommon physical and chemical properties of vent fluids. Diverse fauna, including Annelida, Arthropoda, Mollusca, Echinodermata, Cnidaria, and Chordata, have been described in hydrothermal vents; these vent fauna survive on their unique strategies in extreme conditions [9]. According to the previous studies, typical vent fauna, such as crab (*Austinograea rodriguezensis*) [10], shrimps (*Rimicaris kairei*) [11], *Rimicaris* sp. [12], mussel (*Gigantidas vrijenhoeki*) [13], *Bathymodiolus* mussels [14], and scaly-foot gastropods (*Chrysomallon squamiferum*) [11, 15–18], were evolved to enhance their tolerance of high temperature, metal ion enrichment, and sulfur-rich conditions. The adaptive mechanism is like a hard exoskeleton to endure the thermal stress [10], the ion-binding enzymes, or respiratory proteins for ion homeostasis and detoxification [11, 14–17]. Besides the unusual conditions of hydrothermal vents, the adaptations for high hydrostatic pressure and limited light are inevitable. Among various deep-sea fauna, DNA repair, degenerated ossicles, protein activity protection, and cell cycle maintenance have evolved to high hydrostatic pressure adaptation [5, 19–21]. The white body color, unpigmented skin, scales, and long-wavelength light sensors of marine fauna were ubiquitous in the light-limited deep sea [6, 19, 20, 22, 23]. Gathering knowledge about the genetic basis of adaptation to deep-sea extreme environments is particularly interesting.

Holothurians are widely distributed in several ecosystems’ oceans, and more than 1,800 species have been accepted [24]. Few holothurian species can live in such extreme conditions of hydrothermal environments. *Chiridota heheva* (NCBI:txid2743191; marinespecies.org:taxname:242131) has features of inhabiting in all biotopes of the deep-sea ocean [9, 21, 25–27]. The cold seep adaptations of *C. heheva* have been reported by Zhang et al. [21]. However, genome information on *C. heheva* in the hydrothermal vent is currently unavailable. In the present study, we obtained the genome of *C. heheva* with the sample collected in the Kairei vent. The Kairei vent is an ultramafic-hosted system that was discovered in the Indian Ocean. Kairei fluids are highly enriched in dissolved Fe (5,400 µM) that leach from the host rock [9, 15, 28, 29]. We obtained a chromosome-level genome of *C. heheva* by Hi-C technology with an integrated comprehensive gene set. Moreover, comparative genomic analyses were performed to investigate the hydrothermal vent adaptive mechanisms of *C. heheva*. Finally, together with other published genomic data from vent animals, these assembly results can add more information, which will help to gain insights into the adaptation of the whole vent fauna.

## Methods

### Sampling and sequencing

**Figure 1:**
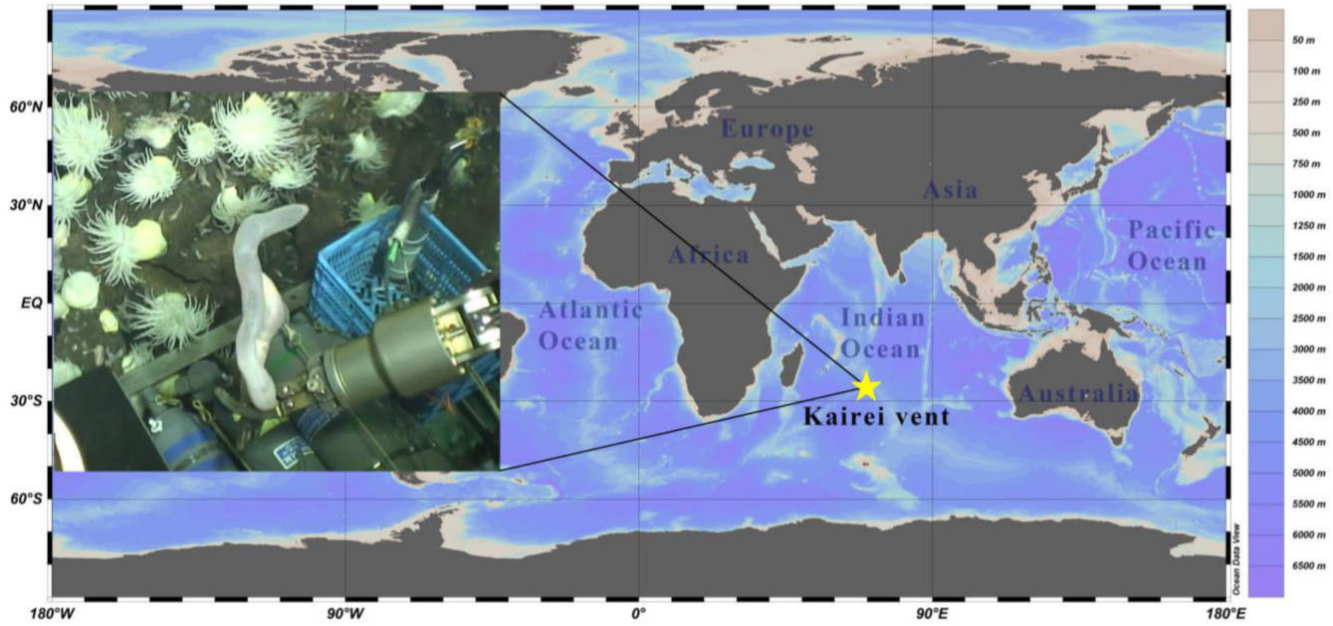
The sampling site at the Kairei vent field of the Indian Ocean and the photo *in situ* at a depth of 2,428 m. Credit: IDSSE Deep-sea Scientific Research Image and Video Database.

The *C. heheva* individual used for genomic sequencing was collected by the maned submersible vehicle *Shenhaiyongshi* from the Kairei vent field in the Mid-Indian Ocean (70.40°E, 25.32°S), with a depth of 2,428 m, on 7 February 2019 (Fig. 1). The sample was dissected and frozen in liquid nitrogen, then sent to the Institute of Deep-sea Science and Engineering, Chinese Academy of Sciences, Sanya, China, and subsequent storage at −80°C for further analysis.

The high-molecular-weight genomic DNA (gDNA) was prepared manually from body-wall tissue following a modified protocol described previously [30]. Briefly, tissue was ground with liquid nitrogen freezing and digested at 65°C in sodium dodecyl sulfate (SDS) buffer (50 mM Tris-HCl, 50 mM EDTA, 3% SDS (w/v)) for 1 hour. Then the lysate was treated by phenol/chloroform isolated and isopropanol precipitation. The gDNA was assessed and sheared to ∼15-kb fragment length for Pacific Biosciences (PacBio) HiFi sequencing. The HiFi SMRTbell library was constructed with the SMRTbell Express Template Prep Kit 2.0 (Pacific Biosciences), and the HiFi reads were sequenced using 1 cell on SMRT cells 8 M on a PacBio Sequel II platform (PacBio Sequel II System, RRID:SCR_017990). For genome annotation, the total RNA was isolated from gonad and body-wall tissues using an RNeasy Plus Universal Kit (QIAGEN). The total RNA was used to obtain complementary DNA by reverse transcribing, and then 150-bp paired-end reads were generated on the Illumina NovaSeq 6000 platform (Illumina NovaSeq 6000 Sequencing System, RRID:SCR_016387). Novogene Company conducted the sequencing processes above.

Hi-C library preparation and sequencing from body-wall tissue have been done following the standard protocol described previously [31]: briefly, crosslinking the grounded body-wall tissue with 4% formaldehyde, digesting the DNA with restriction enzyme MboΙ (GATC), making the DNA ends with biotin-14-dCTP, ligating the blunt-end fragments, and shearing the DNA into 200- to 600-bp fragments by sonication. Finally, the Hi-C sequencing library was constructed and conducted on the Illumina NovaSeq 6000 sequencing platform (PE 150 bp). Novogene Company performed the experiments and sequencing.

### Genome assembly and annotation

Hifiasm version 0.16.1-r375 (RRID:SCR_021069) with the default parameters setting was used for the PacBio HiFi reads assembly [32]. Purge_dups version 1.2.5 (RRID:SCR_021173) was used for redundancy purge of the primary genome and obtained the clean genome without the duplicate contigs [33]. Juicer version 1.6 (RRID:SCR_017226) was used to analyze Hi-C reads combined with the contig-level genome [34]. 3D-DNA version 190716 (3D de novo assembly, RRID:SCR_017227) was used to primarily correct misjoin, order, and orient in the scaffold and obtain the potential chromosomal groups [35]. Juicebox version 1.11.08 was then used to manually order the scaffolds of the result from 3D-DNA [36]. The tool 3D-DNA was used again to obtain the final chromosome assembly for further analysis [35]. The completeness of the chromosome-level genome was assessed using BUSCO version 5.4.6 (RRID:SCR_015008) with the metazoa_odb10 lineage data set (954 orthologs) [37]. The assembly quality was also evaluated using Inspector version 1.0.1, which only relied on third-generation sequencing reads [38].

RepeatModeler version 2.0.1 (RRID:SCR_015027) [39] and RepeatMasker version open-4.0.6 (RRID:SCR_012954) [40] were used for searching repetitive elements in the final genome assembly and generated a soft-masked genome with a nonredundant dataset of repetitive elements. Subsequently, gene structure annotation in the soft-masked genome was predicted by *ab initio* and evidence-based gene prediction. Augustus version 3.4.3 (RRID:SCR_008417) [41], GlimmerHMM version 3.0.4 (RRID:SCR 002654) [42], and GeneID version 1.4.5 (Entrez Gene, RRID:SCR_002473) [43] were used in *ab initio* gene prediction. Moreover, Exonerate version 2.2.0 (RRID: SCR_016088) was employed for protein homologous annotation in evidence-based gene prediction [44]. PASA version 2.5.2 (RRID:SCR_014656) was applied for transcriptomic annotation in evidence-based gene prediction [45]. EVidenceModeler version 1.1.1 (RRID:SCR_014659) produced a weighed consensus protein set by combining the results from *ab initio* gene models and evidence-based gene models [46]. The protein set was used for gene functional annotation as follows. DIAMOND BLASTP version 2.0.14 was used to search protein function in the nr database of NCBI [47]; InterProScan version 5 (RRID:SCR_005829) was employed to predict the protein family membership, functional domains, and sites in Swiss-Prot and Pfam [48]; and KAAS (KEGG Automatic Annotation Server) was applied for KEGG pathways annotated online [49].

### Orthology prediction and phylogenomic analysis

Protein sets of night echinoderm species (*Anneissia japonica, Acanthaster planci, Asterias rubens, Plazaster borealis, Ophiothrix spiculata, Strongylocentrotus purpuratus, Lytechinus variegatus, Apostichopus japonicus*, and *C. heheva*) were employed in the orthology identification with *Homo sapiens* as the out-group (Supplementary Table S1). OrthoFinder version 2.5.4 (RRID:SCR_017118) was applied to determine and cluster gene families among these 10 metazoan species [50]. A total of 495 single-copy orthologs among these species were multiply aligned with MAFFT version 7.475 (RRID:SCR_011811) [51], then concatenated and used for constructing a phylogenomic tree using RAxML version 8.2.3 (RRID:SCR_006086) [52] based on the substitution model of GTRGAMMA with 100 bootstraps. The divergence time among these species was estimated using MCMCTREE in PAML version 4.9 (RRID:SCR_014932) [53]. Based on the TimeTree database (RRID:SCR_021162) [54], Deuterostomia (515.5–636.1 million years ago [Mya] ), Echinodermata (509.0–549.0 Mya), and Eleutherozoa (480.0–488.0 Mya) were applied for the calibration time.

### Genome synteny analysis

Chromosome-level genome in our study of *C. heheva* (CHEH_vent1.0) and *A. japonicus* (AJH1.0) [55] was selected as comparisons for syntenic analysis. BLAST version 2.9.0 (BLAST Similarity Search, RRID:SCR_008419) with parameter “-evalue 1e-10” was used to identify similar gene pairs [56]. JCVI version 0.18 (RRID:SCR_021641) was used to perform protein sequence alignment between CHEH_vent1.0 and AJH1.0 and filter the BLAST results with parameter “–cscore =0.5,” then search for syntenic blocks in all the genes (jcvi, RRID:SCR_021641) [57]. Subsequently, JCVI was also used to visualize the syntenic results with the graphic command.

### Gene family analysis

Based on orthologous gene families and phylogenetic relationships above, CAFE version 4.2.1 (RRID:SCR_005983) [58] was used to detect the gene family expansion and contraction. Gene Ontology (GO) enrichment and KEGG pathway enrichment were performed online [59] and used to investigate the functional properties of the expansion gene families. A conditional *P* value was calculated for each gene family, and a significantly accelerated rate of expansion families was left when *P* values were lower than 0.05.

### Genes under positive selection

As the number of single-copy orthologous genes from OrthoFinder is limited, orthologs were identified as reciprocal best blast hits using the RBH Ortholog pipeline [60]. A total of 3,269 orthologs identified above were used for tests for positive selection. MAFFT version 7.475 (RRID:SCR_011811) [51] was used for multiple alignments, and the alignments of the corresponding DNA codon sequences were further trimmed by trimAl version 1.4.1 (RRID:SCR_017334) [61]. Positively selected genes and amino acid sites were assessed with the branch model and branch-site model using codeml in PAML package version 4.9 (RRID:SCR_014932) [53]. A likelihood ratio test was conducted, and the false discovery rate (FDR) correction was performed for multiple comparisons. Genes and sites with a corrected FDR <0.05 were defined as putatively evolving under positive selection.

### Unique genes

Protein sets of 6 echinoderms (*A. japonica, A. planci, O. spiculata, S. purpuratus*, the Haima cold seep *C. heheva*, and the Kairei vent *C. heheva*) were employed for orthologous clusters analysis. OrthoVenn3 [62] was used to identify clusters based on the OrthoFinder algorithm among the 6 echinoderms. The cluster results were visualized by UpSet (RRID:SCR_022731) [63], which could support the overlapped clusters among diverse species and provide the unique clusters among each species. Based on the unique clusters of the Kairei vent *C. heheva*, the unique genes were collected from the unique clusters and used for GO enrichment. The UpSet and GO enrichment analyses were automatically run on the OrthoVenn3 platform.

## Results

### Chromosome-scale genome assembly and completeness evaluation

The CCS HiFi reads with 29.25 Gb were sequenced on the PacBio Sequel II platform (Supplementary Table S2). In order to create continuity in the genome assembly, 159.59 Gb of Hi-C reads were further prepared on the Illumina NovaSeq 6000 sequencing platform (∼111 × genome coverage) (Supplementary Table S2). RNA reads with 13.29 Gb were generated on the Illumina NovaSeq 6000 sequencing platform utilized for genome annotation (Supplementary Table S2).

**Figure 2:**
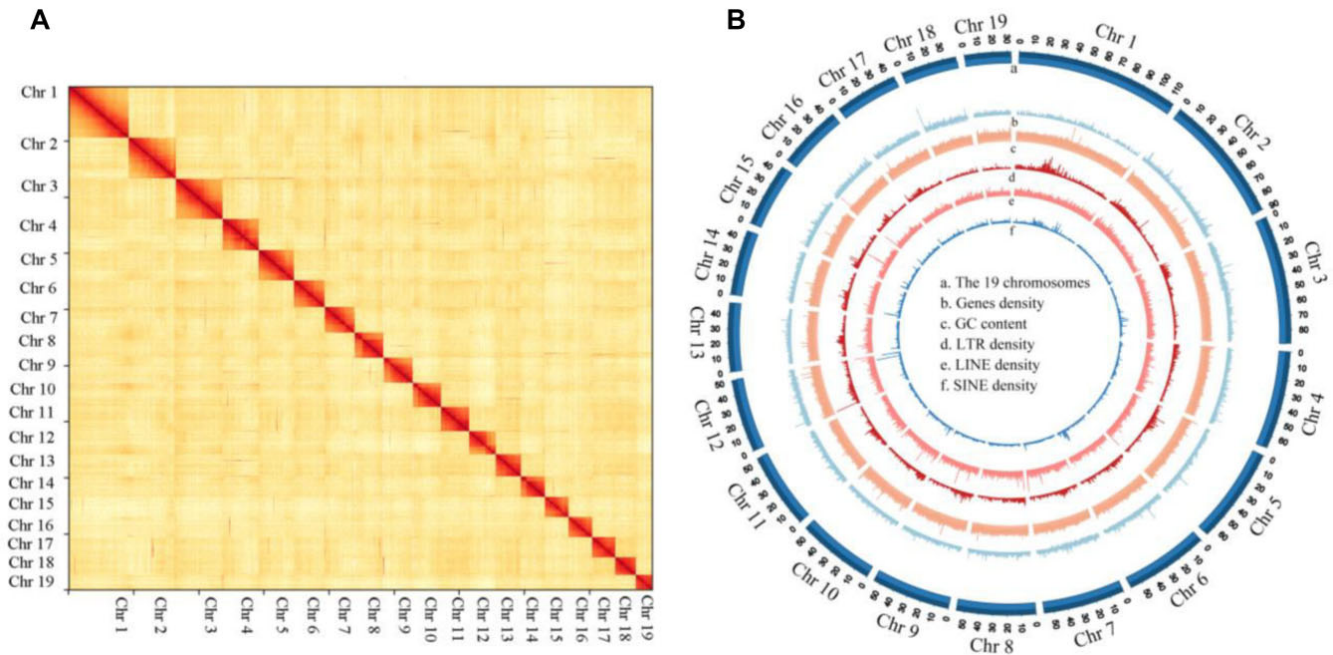
Genome assembly and sequencing analysis of the Kairei vent *Chiridota heheva*. (A) Hi-C interaction heatmap. (B) High-quality assembly of 19 chromosomes with genes coverage, GC content, and repetitive elements of long terminal repeat, LINE, and SINE.

Our chromosome-level genome assembly of *C. heheva* (CHEH_vent1.0) was performed using both HiFi reads and Hi-C reads. The total size of the final assembly was 1.43 Gb with an N50 of 53.24 Mb, consisting of 19 chromosome-level scaffolds with lengths ranging from 30 to 115 Mb (Fig. 2, Table 1). The genome size of this species in the Haima cold seep is 1.107 Gb [21], about three-quarters of the genome size in the Kairei vent. BUSCO [37], with a database of metazoan_odb10, was used to evaluate the completeness of genome, and the BUSCO score was higher in the Kairei vent *C. heheva* (94.50%) when compared with the Haima cold seed *C. heheva* (89.60%) [21]. As the output of Inspector, a high mapping rate (99.76%) suggests better completeness of the assembly, the similarity of alignment depth (20.3745) and sequencing depth (20.3857) indicates a good assembly, and a low error rate (*E*, 0.0011), calculated as QV = −10log_10_*E* based on quality value (QV, 29.7695), reveals high accuracy of the assembly [38].

**Table 1:** Assembly statistics of the *Chiridota heheva* genome assembly

Assembly statistics	Value
Genome size (bp)	1,434,753,151
Number of scaffolds	1,399
Number of chromosome-scale scaffolds	19
N50 of scaffolds (bp)	53,240,875
L50 of scaffolds	11
Chromosome-scale scaffolds (bp)	1,431,787,880
GC content of the genome (%)	37.1231
Error rate	0.0021
**BUSCO analysis**	
Library	Metazoan_odb10 (954)
Complete	94.50% (902)
Complete and single copy	93.50% (892)
Complete and duplicated	1.00% (10)
Fragmented	2.80% (27)
Missing	2.70% (25)
**Inspector analysis**	
Mapping rate	99.76%
Depth	20.3745
Quality value (QV)	29.7695
Error rate (*E*, from QV = −10log_10_*E*)	0.0011

### Annotation of repetitive elements and protein-coding genes

Repetitive element annotation identified that in 70.80% (1.02 Gb) of the whole-genome assembly, the long interspersed nuclear elements (LINEs) were the largest class of the transposable elements annotated; other predominant repetitive elements are summarized in Table 2. Compared with other echinoderms (Supplemental Table S3), the repetitive gene percentage of *C. heheva* (Kairei vent) in this study is more than that in the Haima cold seep (56.64%) [21] and only less than that of *Paelopatides* sp. Yap (73.93%) [23], and the percentage of shallow water *A. japonicus* is only 27.20% [55, 64]. After repeat masking, protein-coding genes annotated used a combination of *ab initio*, homology-based, and transcript evidence–predicted approaches, and a total of 32,434 were successfully identified (Table 3). Interproscan, KEGG, NR, and UniProt were employed for functional annotations, and 24,606 genes were mapped to at least 1 database (Table 3).

**Table 2:** Repeatitive elements of the *Chiridota heheva* genome assembly

Assembly feature	Number of elements	Value (bp)
DNA	15,768	87,173,116
LINE	305,867	353,178,525
SINE	63,461	11,551,395
Long terminal repeat	16,380	23,197,590
Low complexity	27,548	1,906,077
Satellite	44,353	22,459,277
Simple repeat	365,433	399,894,357
Small RNA	18,648	2,502,674
Total	70.80%	1,016,101,549
Unknown	1,635,141	380,635,876

**Table 3:** Annotation statistics of the *Chiridota heheva* genome assembly

Databases of gene annotation	Value
Number of predicted protein-coding genes	32,434
Number of annotated protein-coding genes	24,606
Number of genes annotation to Interproscan	16,086
Number of genes annotation to GO	10,566
Number of genes annotation to Pfam	14,171
Number of genes annotation to KEGG	7,244
Number of genes annotation to NR	18,038
Number of genes annotation to Swiss-Prot	10,711
Number of genes annotation toTrEMBL	17,697

### Phylogenetic and syntenic relationship

**Figure 3:**
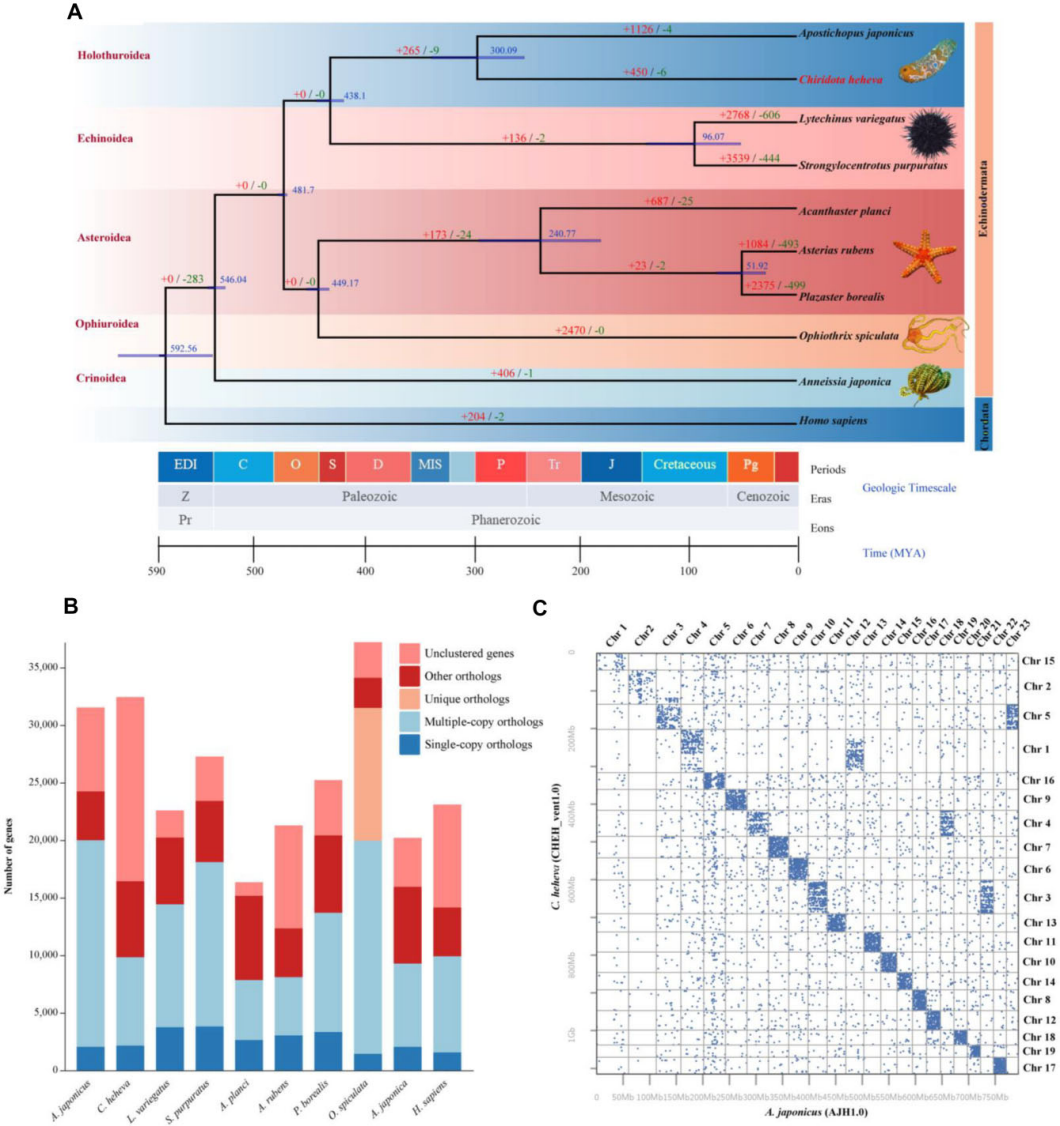
Phylogenetic and syntenic relationships. (A) Phylogenetic relationship and divergence time based on 10 metazoan species orthologous from OrthoFinder. The number on the branches represents gene family expansion (red) or contraction (green) (B) Statistics of orthologous gene numbers in these species. Single-copy orthologs, genes that have only 1 copy in each species and have homologs in other species; Multiple-copy orthologs, genes that have more than 1 copy in each species, together with homologs in other species; Unique orthologs, genes in each species without homologs in other species; Other orthologs, orthologs that do not belong to any type of the above orthologs; Unclustered genes, genes that do not cluster. (C) Synteny between the Kairei vent *Chiridota heheva* and *Apostichopus japonicus* in dot plot.

In order to investigate the phylogenetic relationship between *C. heheva* and other metazoans, 9 species were selected for the phylogenomic tree reconstruction (Supplementary Table S1). A total of 495 single-copy genes in all species with high completeness genomes were used to construct a phylogenomic tree (Fig. 3A, B; Supplemental Fig. S1). *C. heheva* and *A. japonicus* appeared as a sister clade in holothurians and diverged from other echinoderms approximately 438.1 Mya, and the divergence time of holothurians in this study supported the view that holothurians had evolved by the Ordovician [65–68].

The syntenic blocks were detected between *C. heheva* and *A. japonicus* using JCVI [57] and shown as a dot plot (Fig. 3C). The results showed most of the chromosomes of *C. heheva* were highly conserved with *A. japonicus*, except for chromosomes 1, 3, 4, and 5 of *C. heheva*. The results also indicated that events of chromosomal fissions and fusions occurred during the evolutionary history of Holothuroidea, resulting in the variable numbers of chromosomes between *C. heheva* and *A. japonicus*. Based on the chromosomal fissions and fusions, chromosome 1 of *C. heheva* corresponded to chromosomes 4 and 12 of *A. japonicus*. In contrast, while chromosome 3 corresponded to chromosomes 10 and 21, chromosome 4 corresponded to chromosomes 7 and 17, and chromosome 5 corresponded to chromosomes 3 and 23, respectively. The findings of high identity between the 19 chromosomes of *C. heheva* and 23 chromosomes of *A. japonicus* suggested that they share similar gene sets of their origins despite the chromosomal fissions or fusions.

### Gene family evolution

**Figure 4:**
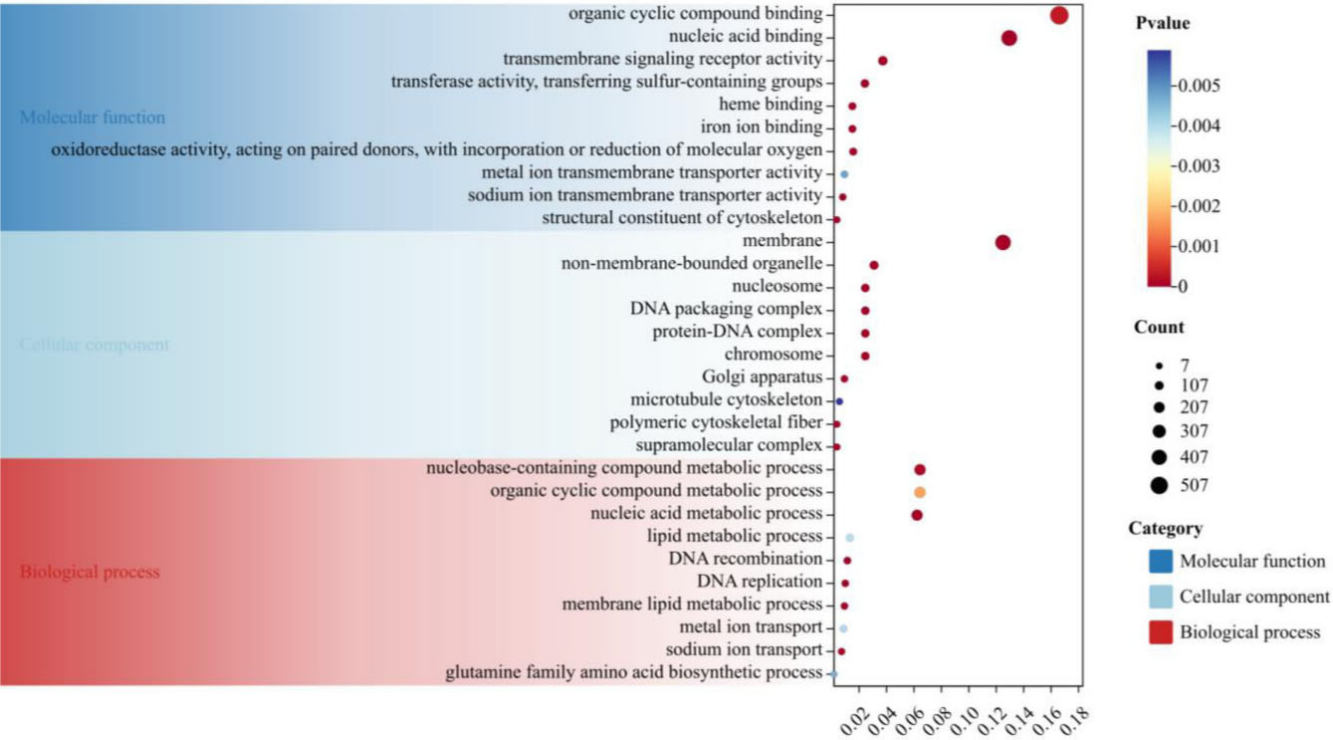
GO enrichment analysis of expanded gene families of the Kairei vent *Chiridota heheva*.

Based on the phylogenomic tree (Fig. 3A), gene family analysis was performed using CAFE [58]. In this analysis, 450 gene family expansions and 6 contractions have been observed in the *C. heheva* lineage since the last common ancestor of *A. japonicus* and *C. heheva* (Fig. 3A). Collectively, these expanded gene families of *C. heheva* were mainly enriched in membrane functions, nucleoside processes of DNA repair, and protein activity (Fig. 4; Supplementary Table S4). Membrane-associated processes have been described that were particularly susceptible to perturbation under conditions of high hydrostatic pressure, including reducing the fluidity of lipid bilayers and denaturing membrane-associated proteins [19, 69, 70]. Biological membranes were mainly composed of phospholipids, sterols (generally cholesterol), glycolipids, and proteins [70, 71]. Phospholipids, with a hydrophilic phosphate group head and hydrophobic fatty acid tails, constitute a significant component that forms the lipid bilayers [71]. Changes in lipid composition modulate membrane fluidity, especially the proportion of unsaturated fatty acids [19, 23, 70–72]. In our results, lipid metabolisms were activated to respond to high hydrostatic pressure, including essential fatty acids of arachidonic acid (AA) metabolism, linoleic acid (LA) metabolism, alpha-linolenic acid (ALA) metabolism, and so on (Fig. 4; Supplementary Table S4). AA, LA, and ALA are polyunsaturated fatty acids (PUFAs) metabolized into various PUFAs during stress (Supplementary Fig. S2). Phospholipid-bound AA is the substrate for the synthesis of a range of biologically active compounds, including prostaglandins, thromboxanes, leukotrienes, epoxyeicosatrienoic acids, and hydroxyeicosatetraenoic acids [73]. LA is important in the biosynthesis of AA, while ALA is the precursor of eicosapentaenoic acid (EPA) and docosahexaenoic acid (DHA) and then converts into EPA and DHA through metabolism [72]. Some of the PUFAs may bind to receptors on the membrane of cells and relate to membrane fluidity adaptation to compensate for environmental changes, especially AA and DHA, which have been reported in previous studies [19, 23, 72, 73]. Iron is crucial in living organisms and intimately involved in numerous biological processes [74, 75]. Iron in organisms is mainly bound to heme (heme iron), transported by transferrin (TF-bound iron), and stored in ferritin (FT-stored iron) for biological functions, which are redox-inert iron in nontoxic forms [74]. Nontransferrin-bound iron and other free iron released from iron-bound proteins are potentially toxic because excess redox-active iron induces oxidative stress and causes cell damage [74, 75]. Deep-sea fauna explored in iron-rich hydrothermal vents have several mechanisms for maintaining iron homeostasis [9, 14, 16, 17]. Cytochrome P450 (CYP) was reported to play an essential role in the regulation of iron levels to maintain cellular redox homeostasis and against oxidative stress [75]. P450 and transferrin are the expanded gene families, which are enriched in iron ion binding and metal ion transport, respectively, suggesting adaptation to the iron-rich environment of the Kairei vent. High hydrostatic pressure may damage DNA in deep-sea fauna susceptibility [20]. The response of DNA repair was variously described in previous studies, including DNA damage detection, replication, recombination, splicing, excision, endonuclease, and so on [5, 20, 23, 76]. The expanded gene families enriched in DNA replication, recombination, DNA-associated protein, and nucleic acid binding suggest a capability of DNA repair. Hydrostatic pressure inhibits protein functions by affecting folding and enzyme activity [19, 21]. These expanded gene families clustered in protein synthesis, and activities of various enzymes may contribute to ensuring the functions of the protein.

### Positively selected genes

**Figure 5:**
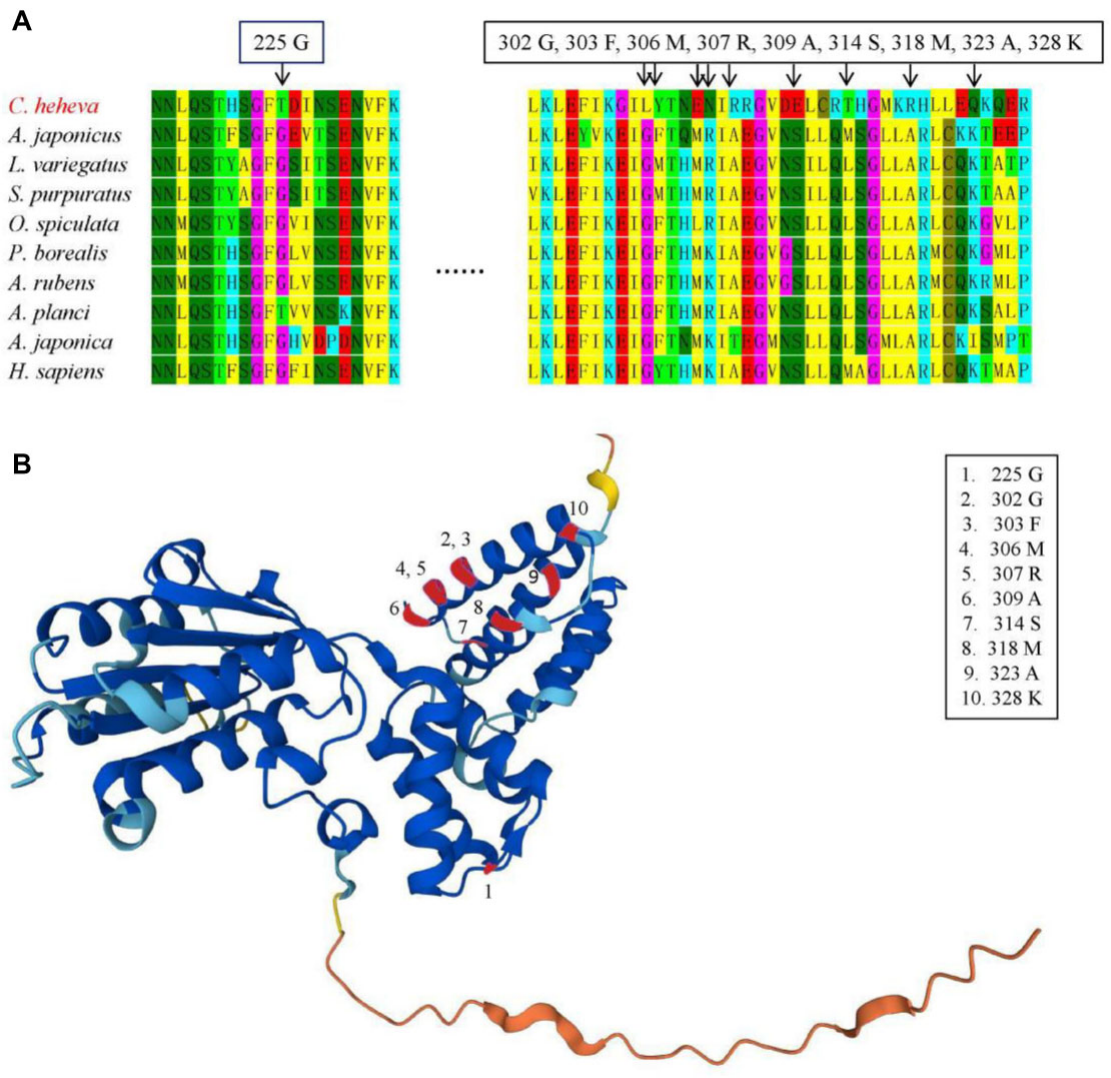
Positively selected amino acid sites of gene *RFC2* in the Kairei vent *Chiridota heheva*. (A) Ten positively selected amino sites in protein sequence. (B) Distribution of 10 positively selected amino sites in 3-dimensional structure from AlphaFold.

The positively selected genes support the genetic basis for environmental adaptation. Compared with the other 9 metazoans, 28 positively selected genes were identified in *C. heheva* (Table 4). According to the GO enrichment analysis, positively selected genes were mainly enriched for various processes, including cyclic compound binding, ion binding, nucleotide binding, ATP binding, DNA binding, DNA repair, and stress response (Supplementary Table S5). Based on the KEGG pathway enrichment, positively selected genes were mainly involved in the Fanconi anemia pathway, an essential component of the DNA damage response and DNA repair [77]. Some genes of deep-sea fauna were positively selected during the adaption to the environment. DNA repair genes have been selected for deep-sea adaptation and may play an important role in maintaining the fidelity of genetic materials in deep-sea environments [5, 20, 23, 76]. Among these processes, at least 11 positively selected genes (*POLB, FAN1, RFC2, KDM2A, FARSA, SPG7, BRCA1, TTLL9, DCLK1, LDHD*, and *SIRT4*) were involved in DNA repair (Table 4; Supplementary Table S5). Therein, DNA repair gene *BRCA1* had been found to protect DNA from high pressure in hadal *Paelopatides* sp. Yap [23]. Furthermore, gene *RFC2*, which functions as DNA replication, nucleotide excision repair, mismatch repair, DNA repair, and recombination proteins, has 10 positively selected sites (Fig. 5A, B). These 10n positively selected sites may enhance the DNA repair abilities of *RFC2* and reveal potential pathways for enhancing the high hydrostatic pressure tolerance. These results indicated that DNA damage repair was mainly reflected in the positively selected genes of the *C. heheva* genome.

**Table 4:** The positively selected genes of *Chiridota heheva* from the Kairei vent

Gene	Description	FDR
*MAEA*	Macrophage erythroblast attacher	2.99E-04
*POLB*	DNA polymerase beta	2.99E-04
*SOD1*	Superoxide dismutase, Cu-Zn family	2.99E-04
*URB1*	Nucleolar preribosomal-associated protein 1	1.21E-03
*FAN1*	Fanconi-associated nuclease 1	1.21E-03
*RFC2*	Replication factor C subunit 2	1.87E-03
*FARSA*	Phenylalanyl-tRNA synthetase alpha chain	4.87E-03
*NUP88*	Nuclear pore complex protein *Nup88*	5.02E-03
*KDM2A*	F-box and leucine-rich repeat protein 11	5.16E-03
*RFT1*	Oligosaccharide translocation protein *RFT1*	8.10E-03
*RNF216*	E3 ubiquitin-protein ligase *RNF216*	1.08E-02
*SPG7*	Spastic paraplegia 7	1.40E-02
*BBOX1*	Gamma-butyrobetaine dioxygenase	1.40E-02
*RPS16*	Small subunit ribosomal protein *S16e*	1.40E-02
*BRCA1*	Breast cancer type 1 susceptibility protein	1.60E-02
*SDR42E1*	Short-chain dehydrogenase/reductase family 42E member 1	2.05E-02
*SSF1_2*	Ribosome biogenesis protein *SSF1/2*	2.37E-02
*LSM4*	U6 snRNA-associated Sm-like protein *LSm4*	2.64E-02
*PSTK*	O-phosphoseryl-tRNA(Sec) kinase	3.13E-02
*ESCO1*	N-acetyltransferase	3.14E-02
*TTLL9*	Tubulin polyglutamylase *TTLL9*	3.20E-02
*HOGA1*	4-Hydroxy-2-oxoglutarate aldolase	3.43E-02
*DCLK1*	Doublecortin-like kinase 1	3.80E-02
*LDHD*	D-lactate dehydrogenase (cytochrome)	4.14E-02
*TEP1*	Telomerase protein component 1	4.14E-02
*SIRT4*	NAD^+^-dependent protein deacetylase sirtuin 4	4.14E-02
*SLC35F5*	Solute carrier family 35, member F5	4.22E-02
*SEH1*	Nucleoporin *SEH1*	4.47E-02

### Unique gene evolution

**Figure 6:**
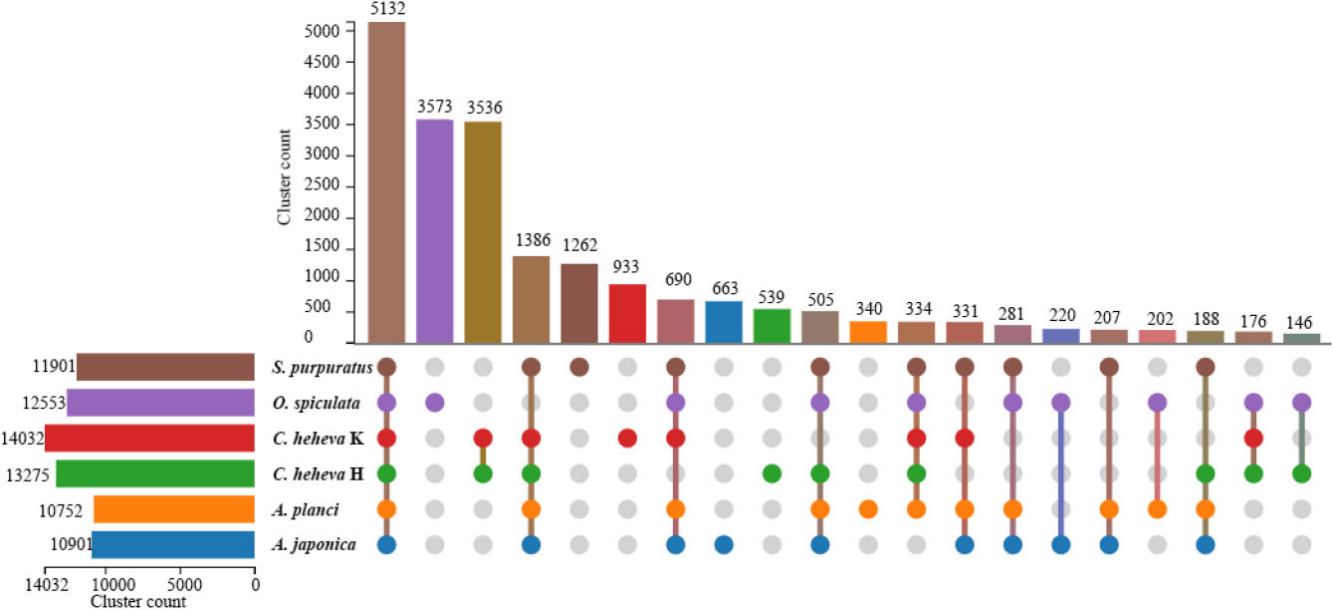
UpSet relationship of protein sets of 6 echinoderms (*A. japonica, A. planci, O. spiculata, S. purpuratus*, the Haima cold seep *C. heheva*, and the Kairei vent *C. heheva*).

**Figure 7:**
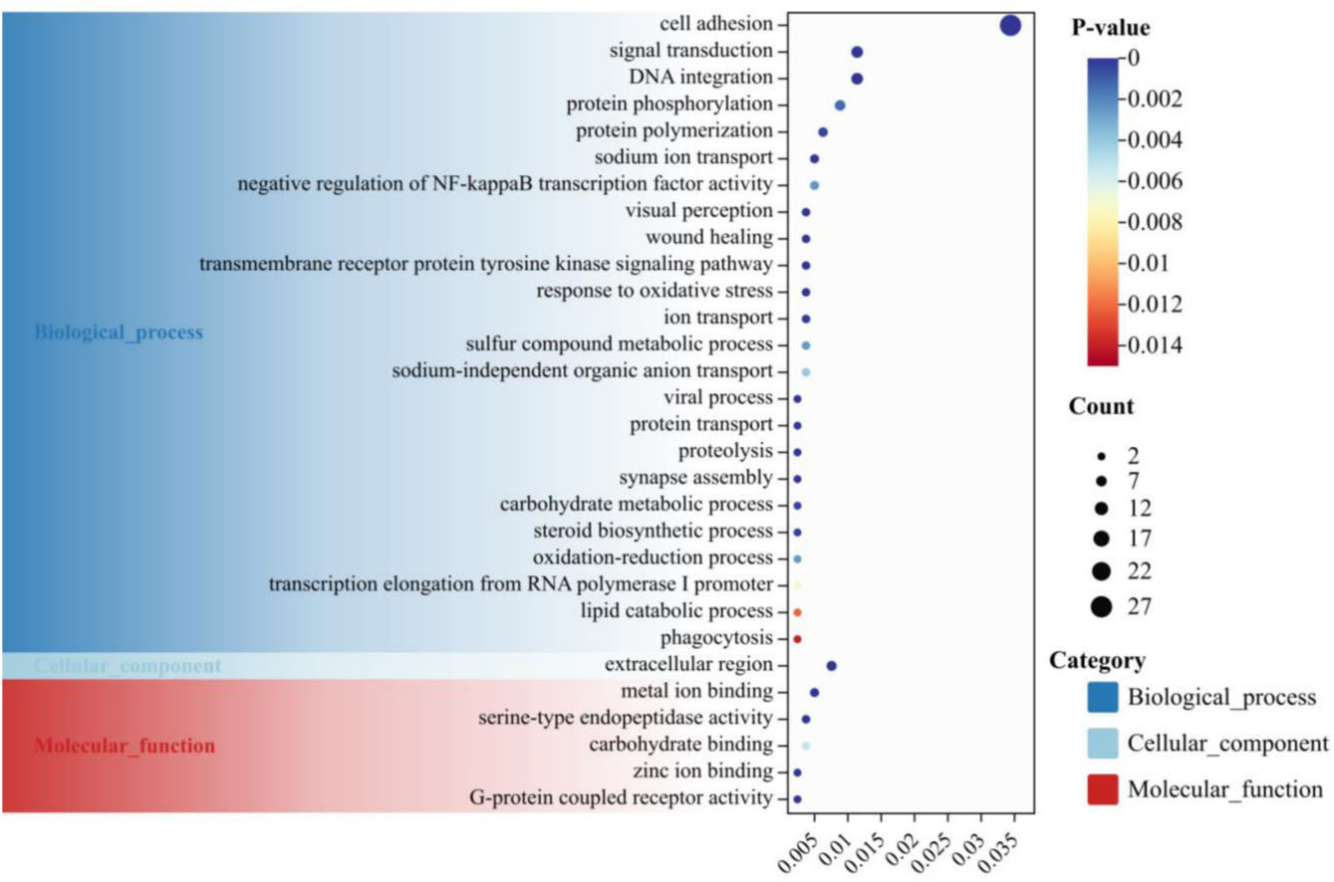
GO enrichment analysis of the unique genes of the Kairei vent *Chiridota heheva*.

In order to investigate the environment-specific adaptation of the Kairei vent *C. heheva*, we identified the unique clusters based on the protein sets of 6 echinoderms. Results of UpSet show that 14,032 clusters were identified in the Kairei vent *C. heheva* and 5,132 clusters were shared with the other 5 echinoderms. In contrast, 933 clusters were identified only in the Kairei vent *C. heheva* and were considered unique clusters (Fig. 6). In the GO enrichment analysis, the unique genes of the Kairei vent *C. heheva* were significantly enriched in the category of ion binding and transport, including sodium ion transport, ion transport, sodium-independent organic anion transport, metal ion binding, and zinc ion binding (Fig. 7; Supplementary Table S6). Among the ion-relative terms, metal ion binding (GO:0046872) exerts an iron ion-binding function through CYP2B14P (cytochrome P450, family 2, subfamily b, polypeptide 14), and CYP450 has acted as a function in maintaining iron homeostasis [75]. The metal ion-binding enriched term of the unique genes of the Kairei vent *C. heheva* suggested environment-specific adaptation due to the Kairei vent being rich in iron [15, 17, 29].

## Conclusions

The first chromosome-level genome, *C. heheva* living in a hydrothermal vent, was assembled and annotated. A total of 19 chromosomal scaffolds were constructed with an N50 of 53.24 Mb. The BUSCO score of 94.5% confirmed the completeness of the genome. Comparative genome analysis results indicated that several positively selected and expanded genes were involved in the DNA repair. Furthermore, the expanded genes and the unique genes contributed to iron ion binding for maintaining iron homeostasis in an iron-rich environment adaptation. This dataset will provide a valuable resource for further studies on hydrothermal vent adaptations of vent fauna.

## Abbreviations

AA: arachidonic acid; ALA: alpha-linolenic acid; BLAST: Basic Local Alignment Search Tool; bp: base pairs; BUSCO: Benchmarking Universal Single-Copy Orthologs; CCS: circular consensus sequencing; FDR: false discovery rate; Gb: gigabase pairs; GC: guanine cytosine; gDNA: genomic DNA; GO: Gene Ontology; Hi-C: High-Throughput Chromosome Conformation Capture; HiFi: high fidelity; KEGG: Kyoto Encyclopedia of Genes and Genomes; LA: linoleic acid; LINE: long interspersed nuclear element; LTR: long terminal repeat; Mb: megabase pairs; NCBI: National Center for Biotechnology Information; NR: NCBI’s nonredundant database; PacBio: Pacific Biosciences; PE: paired end; PUFA: polyunsaturated fatty acid; RBH: reciprocal best hit; SINE: short interspersed nuclear element.

## Supplementary Material

giad107_GIGA-D-23-00018_Original_Submission

giad107_GIGA-D-23-00018_Revision_1

giad107_GIGA-D-23-00018_Revision_2

giad107_Response_to_Reviewer_Comments_Original_Submission

giad107_Response_to_Reviewer_Comments_Revision_1

giad107_Reviewer_1_Report_Original_Submission Muhua Wang -- 3/22/2023 Reviewed

giad107_Reviewer_1_Report_Revision_1 Muhua Wang -- 7/10/2023 Reviewed

giad107_Reviewer_2_Report_Original_Submission Phillip Davidson -- 3/27/2023 Reviewed

giad107_Reviewer_2_Report_Revision_1 Phillip Davidson -- 7/18/2023 Reviewed

giad107_Reviewer_3_Report_Original_Submission Joseph F. Ryan -- 4/5/2023 Reviewed

giad107_Reviewer_3_Report_Revision_1 Joseph F. Ryan -- 7/29/2023 Reviewed

giad107_Supplemental_Files

## Data Availability

The final genome assembly and other associated raw data described in this study are available on ScienceDB [78]. The raw sequencing reads were also deposited at NCBI under BioProject PRJNA934972. All additional supporting data are available in the *GigaScience* repository, GigaDB [79].

## Additional Files

**Supplementary Fig. S1**. BUSCO completeness assessment of gene sets from 10 genomes in phylogenomic analysis.

**Supplementary Fig. S2**. Essential fatty acid metabolism in brief.

**Supplementary Table S1**. Datasets used for the 10 species in phylogenomic analysis.

**Supplementary Table S2**. Statistics of raw sequencing data.

**Supplementary Table S3**. Statistics of repetitive elements percentage in related echinoderms.

**Supplementary Table S4**. GO and KEGG enrichment of expanded gene families of the Kairei vent *Chiridota heheva*.

**Supplementary Table S5**. GO and KEGG enrichment of positively selected genes of the Kairei vent *Chiridota heheva*.

**Supplementary Table S6**. GO enrichment of unique genes of the Kairei vent *Chiridota heheva*.

**Supplementary Table S7**. Reagent and software.

**Supplementary Text S1**. Commands for analyses.
