# Supplementary material for: A high-quality chromosomal genome assembly of the sea cucumber Chiridota heheva and its hydrothermal adaptation
Source: Gigascience. 2024 Jan 4;13:giad107. doi: 10.1093/gigascience/giad107 (PMC10764150; doi:10.1093/gigascience/giad107)
Supplement: giad107_GIGA-D-23-00018_Original_Submission [file giad107_giga-d-23-00018_original_submission.pdf]

GigaScience

The chromosome-level genome of Chiridota heheva (Holothuroidea, Apodida,Chiridotidae) provides insights into adaptation to the hydrothermal environment

--Manuscript Draft--

**Manuscript Number:**	GIGA-D-23-00018
**Full Title:**	The chromosome-level genome of Chiridota heheva (Holothuroidea, Apodida,Chiridotidae) provides insights into adaptation to the hydrothermal environment
**Article Type:**	Data Note
**Funding Information:**	Strategic Priority Research Program of the Chinese Academy of Sciences (CAS) (XDA22050303) — Prof. Jun Liu<br>National Key Research and Development Program of China (2016YFC0304905) — Prof. Jun Liu<br>the major scientific and technological projects of Hainan Province (ZDKJ2019011) — professor Haibin Zhang
**Abstract:**	Abstract<br>Background: Chiridota heheva is one of the cosmopolitan holothurian, which has been found well adapted to diverse deep-sea ecosystems, especially chemosynthetic environments. Besides high hydrostatic pressure and limited light, high concentrations of metal ions also represent harsh conditions in the hydrothermal vent. Few holothurian species can live in such extreme condition. Therefore, it is valuable to elucidate the adaptive genetic mechanisms of C. heheva to hydrothermal environment.<br>Findings: Herein we report a high-quality reference genome assembly of C. heheva from the Kairei vent, which is the first chromosome-level genome of Apodida. The chromosome-level genome size was 1.4 Gb, with a N50 of 53.24 Mb and BUSCO score of 92.40%. Contig sequences were clustered, ordered, and assembled into 19 large scaffolds, each corresponding to a natural chromosome. We found that C. heheva has expanded gene families, positively selected genes, and unique genes involved in DNA protection under high hydrostatic pressure, and contributed to regulating mechanism of iron ions which enriched in surrounding vent fluid. Positively selected gene RCF2 with 10 positively selected sites played an essential role in DNA repair under high hydrostatic pressure. Unique gene FTH1 could enhance iron tolerance by keeping it in a nontoxic state in an iron-rich hydrothermal vent environment.<br>Conclusions: This first chromosome-level genome assembly of C. heheva provides the insight of how holothurians adapt to hydrothermal envinronment. As it also the first chromosome-level genome of Order Apodida, it will help for investigating the evolution of Class Holothuroidea.
**Corresponding Author:**	Haibin Zhang, Ph.D<br>Institute of Deep-sea Science and Engineering Chinese Academy of Sciences<br>Sanya, Hainan CHINA
**Corresponding Author Secondary Information:**	
**Corresponding Author's Institution:**	Institute of Deep-sea Science and Engineering Chinese Academy of Sciences
**Corresponding Author's Secondary Institution:**	
**First Author:**	Yujin Pu
**First Author Secondary Information:**	
**Order of Authors:**	Yujin Pu<br>Yang Zhou

Powered by Editorial Manager® and ProduXion Manager® from Aries Systems Corporation

	Jun Liu
	Haibin Zhang, Ph.D
Order of Authors Secondary Information:	
Additional Information:	
Question	Response
Are you submitting this manuscript to a special series or article collection?	No
**Experimental design and statistics**<br><br>Full details of the experimental design and statistical methods used should be given in the Methods section, as detailed in our Minimum Standards Reporting Checklist. Information essential to interpreting the data presented should be made available in the figure legends.<br><br>Have you included all the information requested in your manuscript?	Yes
**Resources**<br><br>A description of all resources used, including antibodies, cell lines, animals and software tools, with enough information to allow them to be uniquely identified, should be included in the Methods section. Authors are strongly encouraged to cite Research Resource Identifiers (RRIDs) for antibodies, model organisms and tools, where possible.<br><br>Have you included the information requested as detailed in our Minimum Standards Reporting Checklist?	Yes
**Availability of data and materials**<br><br>All datasets and code on which the conclusions of the paper rely must be either included in your submission or deposited in publicly available repositories (where available and ethically appropriate), referencing such data using	Yes

Powered by Editorial Manager® and ProduXion Manager® from Aries Systems Corporation

a unique identifier in the references and in the "Availability of Data and Materials" section of your manuscript.<br><br>Have you have met the above requirement as detailed in our Minimum Standards Reporting Checklist?	

Powered by Editorial Manager® and ProduXion Manager® from Aries Systems Corporation

Manuscript
Click here to access/download;Manuscript;Manuscript-submission.docx

The chromosome-level genome of *Chiridota heheva* (Holothuroidea, Apodida,Chiridotidae) provides insights into adaptation to the hydrothermal environment

Yujin Pu, Yang Zhou, Jun Liu, Haibin Zhang*

[1] Institute of Deep-sea Science and Engineering, Chinese Academy of Sciences, Sanya 572000, China

[2] University of Chinese Academy of Sciences, Beijing 100049, China

*Correspondding address. Haibin Zhang, Institute of Deep-sea Science and Engineering, Chinese Academy of Sciences, Sanya 572000, China. E-mail: hzhang@idsse.ac.cn

Abstract

Background: *Chiridota heheva* is one of the cosmopolitan holothurian, which has been found well adapted to diverse deep-sea ecosystems, especially chemosynthetic environments. Besides high hydrostatic pressure and limited light, high concentrations of metal ions also represent harsh conditions in the hydrothermal vent. Few holothurian species can live in such extreme condition. Therefore, it is valuable to elucidate the adaptive genetic mechanisms of *C. heheva* to hydrothermal environment.

Findings: Herein we report a high-quality reference genome assembly of *C. heheva* from the Kairei vent, which is the first chromosome-level genome of Apodida. The chromosome-level genome size was 1.4 Gb, with a N50 of 53.24 Mb and BUSCO score of 92.40%. Contig sequences were clustered, ordered, and assembled into 19

large scaffolds, each corresponding to a natural chromosome. We found that *C. heheva* has expanded gene families, positively selected genes, and unique genes involved in DNA protection under high hydrostatic pressure, and contributed to regulating mechanism of iron ions which enriched in surrounding vent fluid. Positively selected gene *RCF2* with 10 positively selected sites played an essential role in DNA repair under high hydrostatic pressure. Unique gene *FTH1* could enhance iron tolerance by keeping it in a nontoxic state in an iron-rich hydrothermal vent environment.

Conclusions: This first chromosome-level genome assembly of *C. heheva* provides the insight of how holothurians adapt to hydrothermal envinronment. As it also the first chromosome-level genome of Order Apodida, it will help for investigating the evolution of Class Holothuroidea.

Keywords: *Chiridota heheva,* Hi-C, positively selected gene, gene family, unique gene

Date Description

Context

Hydrothermal vents are one of the typical deep-sea chemosynthetically-driven ecosystems that inhabit a wide array of animals and chemosynthetic microbes. The hydrothermal vent environment is characterized by rapid changes in temperature, acidic pH, sulfur compounds, metal, methane, hydrogen, carbon dioxide, and other toxic chemistry, besides high hydrostatic pressure and darkness of the deep sea [1-9].

However, these inhospitable environments had reported as key areas of enrichment for deep-sea life.

In order to survive in hydrothermal vents, unusual environmental adaptability of fauna has evolved to adapt to the uncommon physical and chemical properties of vent fluids. Diverse fauna, including Annelida, Arthropoda, Mollusca, Echinodermata, Cnidaria, and Chordata have been described in hydrothermal vents, these vent faunas survive on their unique strategies in the extreme conditions [9]. The hydrothermal vent adaptation of Arthropoda had reported as following. Crab *Austinograea rodriguezensis* in Onnuri vent evolved a hard exoskeleton to endure the barotraumatic and thermal stress [10]. Shrimps *Rimicaris kairei* and *Mirocaris indica* prefer to gather on the chimney structures hosting high-temperature fluids in the Solitaire hydrothermal field based on their high-temperature tolerance [11]. The symbiotic microbial of shrimp *R. kairei* in iron-rich Kairei vent were fueled by the iron for chemosynthesis [8]. Shrimp *Rimicaris* sp. of hydrothermal vent in Desmos adapted to H_2S-rich environment associated with sulfur metabolism and detoxification [12]. On the other hand, hydrothermal vent adaptation of Mollusca had studied a lot in previous researches. Onnuri vent mussel *Gigantidas vrijenhoeki* obtain nutrients through symbiotic relationships with sulfur-oxidizing and methane-oxidizing bacteria [13]. *Bathymodiolus* mussels from hydrothermal vents usually had higher metal concentrations (Fe, Cr, Cd, and Pb) in the metal-rich environment, in which the enriched Fe was binding for enzymes, respiratory proteins, and structural elements, and the Fe content was regulated by CAT for homeostasis and detoxification [14].

Scaly-food gastropods *Chrysomallon squamiferum* could fixate iron as iron sulfide in scales by biomineralization under iron-rich Kairei vent, enhance metal ions tolerance by increasing the expression of metal tolerance protein 9 (*MTP9*), both pathways above were involved in intracellular homoeostasis maintaining [11, 15-17]. Unlike Kairei vent *C. squamiferum,* the Tiancheng *C. squamiferum* was coated in zinc sulfide by different mechanism [18]. Detoxification processes of those enriched chemicals that dissolved in hydrothermal vent fluids occurred in vent fauna, indicating that vent fauna has a unique adaptive mechanism for these extreme ecosystems. Besides the unusual conditions of hydrothermal vents, vent fauna also needs to adapt to common deep-sea conditions as high hydrostatic pressure and limited light. In previous studies, deep-sea endemic species have developed abilities to survival in the deep sea with high hydrostatic pressure and darkness. In deep-sea fauna, DNA repair, degenerated ossicles, protein activity protection, and cell cycle maintenance have evolved to high hydrostatic pressure adaptation[5, 19-21]. The white body colour, unpigmented skin, scales, and long-wavelength light sensors of marine fauna were ubiquitous in the light-limited deep sea [6, 19-20, 22-23]. To gather knowledge about the genetic basis of adaptation to deep-sea extreme environments are of particular interest.

Holothurians are widely distributed in several ecosystems' ocean, and more than 1,800 specie have been accepted at present [24]. Few holothurian species can live in such extreme condition of hydrothermal environments. Chiridotidae is one of the represented families of Holothurians in the deep-sea hydrothermal vents [9]. *Chiridota heheva*, a cosmopolitan Chiridotidae holothurian, has been found in

hydrothermal vents, cold seeps, and other organic falls [25-26]. *C. heheva* may be an ideal model for studying how marine fauna have adapted to extreme deep-sea chemosynthetic environments. The cold seep adaptations of *C. heheva* have been reported by Zhang et al. [21]. However, genome information on *C. heheva* in hydrothermal vent is currently unavailable. In the present study, we sequenced the genome of *C. heheva* with sample collected in the Kairei vent. Kairei vent is an ultramafic-hosted system was discovered in the Indian Ocean. Kairei fluids are highly enriched in dissolved Fe (5,400 μM) that leach from the host rock [9, 15, 27-28]. We obtained a chromosome-level genome of *C. heheva* by Hi-C technology with an integrated comprehensive gene set. Moreover, comparative genomic analyses were performed to investigate the hydrothermal vent adaptive mechanisms of *C. heheva*. Finally, together with other published genomic data from vent animals, this assembly results can add more information which will helpful to gain insights into the adaptation of the whole vent fauna.

Methods

Sampling and sequencing

The *C. heheva* individual used for genomic sequencing was collected by the maned submersible vehicle '*Shenhaiyongshi*' from the Kairei vent filed in the Mid-Indian Ocean (70.40°E, 25.32°S), with a depth of 2428 m, on 7 February 2019 (Fig. 1). The sample was dissected and frozen in liquid nitrogen, then send to the Institute of Deep-sea Science and Engineering, Chinese Academy of Science, Sanya,

China, and subsequent storage at -80℃ for further analysis.

The high-molecular-weight genomic DNA (gDNA) was prepared manually from body-wall tissue following a modified protocol described previously [29]. Briefly, tissue was ground with liquid nitrogen freezing and digested at 65℃ in SDS (sodium dodecyl sulfate) buffer [50 mM Tris-HCl, 50 mM EDTA, 3% SDS (w/v)] for 1 h. Then the lysate was treated by Phenol/Chloroform isolated and Isopropanol precipitation. The gDNA was assessed and sheared to ~15 kb fragment length for Pacific Biosciences (PacBio) HiFi sequencing. The HiFi SMRTbell library was constructed with SMRTbell Express Template Prep Kit 2.0 (Pacific Biosciences, California, USA), and the HiFi reads were sequenced using 1 cell on SMRT cells 8M on a PacBio Sequel II platform (PacBio Sequel II System, RRID:SCR_017990). For genome annotation, the total RNA was isolated from gonad and body-wall tissues using a RNeasy Plus Universal Kit (QIAGEN, Hilden, Germany). The total RNA was used to obtain cDNA by reverse transcribing, and then 150 bp paired-end reads were generated on Illumina NovaSeq 6000 platform (Illumina NovaSeq 6000 Sequencing System, RRID:SCR_016387). The sequencing processes above were conducted by Novogene Company, Tianjin, China.

Hi-C library preparation and sequencing from body-wall tissue have been done following the standard protocol described previously [30]. Briefly, crosslinking the grounded body-wall tissue with 4% formaldehyde, digesting the DNA with restriction enzyme MboI (GATC), making the DNA ends with biotin-14-dCTP, ligating the blunt-end fragments, shearing the DNA into 200- to 600 bp fragments by sonication.

Finally, the Hi-C sequencing library was constructed and conducted on the Illumina NovaSeq-6000 sequencing platform (PE 150bp). The experiments and sequencing were performed by Novogene Company, Tianjin, China.

Genome assembly and annotation

Hifiasm version 0.16.1-r375 (Hifiasm, RRID:SCR_021069) with default parameters setting was used for PacBio HiFi reads assembly [31]. Purge_dups version 1.2.5 (purge dups, RRID:SCR_021173) was used for redundancy purge of the primary genome and obtained the scaffold-level genome [32]. Juicer version 1.6 (Juicer, RRID:SCR_017226) was used to analyze Hi-C reads combined with scaffold-level genome [33]. 3D-DNA version 190716 (3D de novo assembly, RRID:SCR_017227) was used to primarily correct misjoin, order and orient in the scaffold and obtained the potential chromosomal groups [34]. Juicerbox version 1.11.08 was then used to manually order the scaffolds of the result from 3D-DNA [35]. The tool 3D-DNA was used again to obtain the final chromosome assembly for further analysis [34]. The completeness of the chromosome-level genome was assessed using BUSCO version 5.1.2 (BUSCO, RRID:SCR_015008) with the metazoa_odb10 lineage data set (954 orthologs) [36].

RepeatModeler version 2.0.1 (RepeatModeler, RRID:SCR_015027) [37] and RepeatMasker version open-4.0.6 (RepeatMasker, RRID:SCR_012954) [38] were used for searching repetitive elements in the final genome assembly and generated a soft-masked genome with non-redundant data set of repetitive elements. Subsequently,

gene structure annotation in the soft-masked genome was predicted by *ab initio* and evidence-based gene prediction as follow. Augustus version 3.4.3 (Augustus, RRID:SCR_008417) [39], GlimmerHMM version 3.0.4 (GlimmerHMM, RRID:SCR 002654) [40], and GeneID version 1.4.5 (Entrez Gene, RRID:SCR_002473) [41] were used in *ab initio* gene prediction. Moreover, Exonerate version 2.2.0 (Exonerate, RRID: SCR_016088) was employed for protein homologous annotation in evidence-based gene prediction [42]. PASA version 2.5.2 (PASA, RRID:SCR_014656) was applied for transcriptomic annotation in evidence-based gene prediction [43]. EVidenceModeler version 1.1.1 (EVidenceModeler, RRID:SCR_014659) produced a weighed consensus protein set by combining the results from *ab initio* gene models and evidence-based gene models [44]. The protein set was used for gene functional annotation as follows. DIAMOND BLASTP version 2.0.14 was used to search protein function in the nr database of NCBI [45], Interproscan version 5 (InterProScan, RRID:SCR_005829) was employed to predict the protein family membership, functional domains and sites in Swiss-Prot, Pfam [46], and KAAS (KEGG Automatic Annotation Server) was applied for KEGG pathways annotated online (https://www.genome.jp/tools/kaas/).

Orthology prediction and phylogenomic analysis

Protein sets of night echinoderm species (*Anneissia japonica*, *Acanthaster planci*, *Asterias rubens*, *Plazaster borealis*, *Ophiothrix spiculata*, *Strongylocentrotus purpuratus*, *Lytechinus variegatus*, *Apostichopus japonicus*, and *C. heheva*) were

employed in the orthology identification with *Homo sapiens* as the outgroup (Supplementary Table S1). OrthoFinder version 2.5.4 (OrthoFinder, RRID:SCR_017118) was applied to determine and cluster gene families among these 10 metazoan species [47]. A total of 495 single-copy orthologs among these species were multiple aligned with MAFFT version 7.475 (MAFFT, RRID:SCR_011811) [48], then concatenated and used for constructing a phylogenomic tree using RAxML version 8.2.3 (RAxML, RRID:SCR_006086) [49] based on the substitution model of GTRGAMMA with 100 bootstraps. The divergence time among these species was estimated using MCMCTREE in PAML version 4.9 (PAML, RRID:SCR_014932) [500]. The calibration times were derived from the TimeTree database (http://www.timetree.org/).

Genome synteny analysis

Chromosome-level genome in our study of *C. heheva* (CHEH_vent1.0) and *A. japonicus* (AJH1.0) [51] was selected as comparisons for syntenic analysis. BLAST version 2.9.0 (BLAST Similarity Search, RRID:SCR_008419) with parameter "-evalue 1e-10" was used to identify similar gene pairs [52]. JCVI version 0.18 (RRID:SCR_021641) was used to filter the BLAST results with parameter "--cscore =0.5" and search for syntenic blocks in all the genes (jcvi, RRID:SCR_021641) [53]. Subsequently, JCVI was also used to visualize the syntenic results with the graphic command.

Gene family analysis

Based on orthologous gene families and phylogenetic relationships above, CAFE version 4.2.1 (CAFE, RRID: SCR_005983) [54] was used to detect the gene family expansion and contraction. GO enrichment and KEGG pathway enrichment were performed online (https://www.genescloud.cn/) and were used to investigate the functional properties of the expansion gene families. A conditional P value was calculated for each gene family, and a significantly accelerated rate of expansion families was left while P values were lower than 0.05.

Genes under positive selected

As the number of single-copy orthologous genes from OrthoFinder is limited, orthologs were identified as reciprocal best blast hits using the RBH Ortholog pipeline [55]. A total of 3,269 orthologs identified above were used for positively selected analysis. MAFFT version 7.475 (MAFFT, RRID:SCR_011811) [48] was used for multiple aligned, and the alignments of the corresponding DNA codon sequences further trimmed by trimAl version 1.4.1 (trimAl, RRID:SCR_017334) [56]. Positively selected genes and amino acid sites were assessed with the branch model and branch-site model using codeml in PAML package version 4.9 (PAML, RRID:SCR_014932) [50]. A likelihood ratio test was conducted, and the false discovery rate (FDR) correction was performed for multiple comparisons. Genes and sites with a corrected FDR <0.05 were defined as positively selected.

Unique genes

Based on the GO and KEGG annotation results, the Kairei vent *C. heheva* was compared with Haima cold seep *C. heheva* [21] and *A. japonicus* [51], then obtained its unique genes and visualized by TBtools version 1.1043 (TBtools, RRID:SCR_023018) [57] with Venn diagram function. The functional enrichment analysis of these unique genes was performed online (https://www.genescloud.cn/) by GO and KEGG enrichment.

Results

Chromosome-scale genome assembly and completeness evaluation

The CCS HiFi reads with 29.25 Gb were sequenced on the PacBio Sequel II platform (Supplementary Table S2). In order to create continuity in the genome assembly, 159.59 Gb of Hi-C reads were further prepared on Illumina NovaSeq 6000 sequencing platform (~ 111 × genome coverage) (Supplementary Table S2). RNA reads with 13.29 Gb were generated on Illumina NovaSeq 6000 sequencing platform utilized for genome annotation (Supplementary Table S2).

Our chromosome-level genome assembly of *C. heheva* (CHEH_vent1.0) was performed using both HiFi reads and Hi-C reads. The total size of the final assembly was 1.43 Gb with an N50 of 53.24 Mb, consisting of 19 chromosome-level scaffolds with lengths ranging from 30 to 115 Mb (Fig. 2, Table 1). The genome size of this species in the Haima cold seep is 1.107 Gb [21], only three-quarters of the genome

size in Kairei vent. BUSCO [36] with a database of metazoan_odb10 was then used to evaluate the integrity of the genome assembly, the results as 92.4% of the conserved genes indicated the high integrity of our assembled genome in CHEH_vent1.0 (Table 1).

Annotation of repetitive elements and protein-coding genes

Repetitive element annotation identified that in 70.80% (1.02 Gb) of the whole genome assembly, the long interspersed nuclear elements (LINEs) were the largest class of the transposable elements (TEs) annotated, other predominant repetitive elements are summarized in Table 2. Compared with other echinoderms (Supplementary Table S3), the repetitive genes percentage of *C. heheva* (Kairei vent) in this study is more than that 56.64% in Haima Cold Seep [21], and only less than *Paelopatides sp.* Yap 73.93% [23], and the percentage of shallow water *A. japonicus* only have 27.20% [51, 58]. After repeat masking, protein-coding genes annotated were using a combination of *ab initio*, homology-based, and transcript-evidence predicted approaches, and a total of 32,434 were successfully identified (Table 3). Interproscan, KEGG, NR, and UniProt were employed for functional annotations, and 24,606 genes were mapped to at least one database (Table 3). BUSCO benchmarking value of this gene set was summarized as 95.00% of completeness, reflecting high integrity (Table 3).

Phylogenetic and syntenic relationship

In order to investigate the phylogenetic relationship between *C. heheva* and other metazoans, 9 species were selected for the phylogenomic tree reconstruction (Supplementary Table S1). A total of 495 single-copy genes in all species with high completeness genomes were used to construct a phylogenomic tree (Fig. 3A, B; Supplemental Fig. S1). *C. heheva* and *A. japonicus* appeared as a sister clade in holothurians, and diverged from other echinoderms approximately 438.1 Mya. The divergence time of holothurians in this study supported the view that holothurians had evolved by the Ordovician [59-62].

The syntenic blocks were detected between *C. heheva* and *A. japonicus* using JCVI [53] and shown as a dot plotter (Fig. 3C). The results indicated *C. heheva* has a relatively conserved relationship with *A. japonicus*, except chromosomes 1, 3, 4, and 5 of *C. heheva*. We identified chromosome fission or fusion events between the 19 chromosomes of *C. heheva* and 23 chromosomes of *A. japonicus*. Chromosome 1 of *C. heheva* corresponded to chromosomes 4 and 12 of *A. japonicus*, while chromosome 3 corresponded to chromosomes 10 and 21, chromosome 4 corresponded to chromosomes 7 and 17, and chromosome 5 corresponded to chromosomes 3 and 23, respectively.

Gene family evolution

Based on the phylogenomic tree (Fig. 3A), gene family analysis was performed using CAFE [54]. Compared with the other 9 metazoans, 450 gene families were

expanded, and 6 were contracted in *C. heheva* (Fig. 3A). Collectively, these expanded gene families of *C. heheva* were mainly enriched in membrane functions, nucleoside processes of DNA repair, and proteins activity (Fig. 4; Supplementary Table S4). Membrane-associated processes have been described that were particularly susceptible to perturbation under conditions of high hydrostatic pressure, including reducing the fluidity of lipid bilayers and denaturing membrane-associated proteins [19, 63]. The composition of unsaturated fatty acids is involved in membrane fluidity and maintained transport functions [19, 23, 64]. These expanded gene families related to lipid metabolism may support a high percentage of unsaturated fatty acids in the membrane. Deep-sea fauna explored in iron-rich hydrothermal vents evolved strategies to tolerate high loads of iron, such as transforming toxic iron to safe iron status, and transporting iron for maintaining homeostasis [9, 14, 16-17]. These expanded gene families associated with iron ion binding and metal ion transport suggested adapting to the iron-rich environment of the Kairei vent. High hydrostatic pressure may damage DNA in deep-sea fauna susceptibly [20]. The responding of DNA repair was variously described in previous studies, including DNA damage detection, replication, recombination, splicing, excision, endonuclease, and so on [5, 20, 23, 65]. These expanded gene families enriched in DNA replication, recombination, DNA-associated protein, and nucleic acid binding may enhance DNA-protected abilities. High hydrostatic pressure inhibits protein functions by affecting folding and enzyme activity [19, 21]. These expanded gene families clustered in protein synthesis and activities of various enzymes were contributed to

ensuring the functions of the protein.

Positively selected genes

The positively selected genes support the genetic basis for environmental adaptation. Compared with the other 9 metazoans, 28 positively selected genes were identified in *C. heheva* (Table 4). According to GO enrichment and KEGG enrichment functional analysis, positively selected genes were significantly enriched for various processes including cyclic compound binding, ion binding, nucleotide binding, ATP binding, and DNA binding and damage repair (Supplementary Table S5). Deep-sea fauna obtained positively selected genes through adaptive evolution. DNA repair genes had been selected for deep-sea adaptation, and may play an important role in maintaining the fidelity of genetic materials in deep-sea environments [5, 20, 23, 65]. Among these processes, at least 11 positively selected genes (*POLB*, *FAN1*, *RFC2*, *KDM2A*, *FARSA*, *SPG7*, *BRCA1*, *TTLL9*, *DCLK1*, *LDHD*, and *SIRT4*) involved in DNA damage that mainly induced by high hydrostatic pressure (Table 4; Supplementary Table S5). Therein DNA repair gene *BRCA1* had been found to protect DNA from high pressure in hadal *Paelopatides* sp. Yap [23]. Furthermore, gene *RCF2*, which functions as DNA replication, nucleotide excision repair, mismatch repair, DNA repair and recombination proteins, has 10 positively selected sites (Fig. 5A, B). These 10 positively selected sites may enhance the DNA repair abilities of *RCF2* and reveal potential pathways for enhancing the high hydrostatic pressure tolerance.

Unique genes evolution

To investigate the environment-specific genes, Kairei vent *C. heheva* was compared with Haima Cold Seep *C. heheva* and shallow water *A. japonicus*. As the Venn diagram shows, 305 unique genes in KEGG annotation and 330 unique genes in GO annotation were found in Kairei vent *C. heheva* (Fig. 6A, B). Functional enrichment analyses of GO and KEGG show that unique genes were significantly enriched for various enzymatic activity regulation, cellular homeostasis, substances binding, development, and transport (Fig. 6C; Supplementary Table S6). As the vent fauna in the Kairei vent, fauna had to adapt Kairei iron-rich environment [15, 17, 28]. Terms of acireductone dioxygenase [iron(II)-requiring] activity, ferric iron binding, iron ion homeostasis, transition metal ion homeostasis, and cellular chemical homeostasis were enriched to adapt the iron-rich environment in the Kairei vent. The mainly unique gene above is ferritin heavy chain 1 (*FTH1*), this gene encodes the heavy subunit of ferritin, the major intracellular iron storage protein, and the function of ferritin is the storage of iron in a soluble and nontoxic state. For the deep-sea adaptation of high hydrostatic pressure, unique genes were also enriched in nucleotide-excision repair and homologous recombination pathway for DNA repair.

Conclusions

The first chromosome-level genome *C. heheva* living in hydrothermal vent was assembled and annotated. A number of 19 chromosomal scaffolds are constructed with N50 of 53.24 Mb. The completeness of the genome was confirmed by the

BUSCO score of 92.4%. Comparative genome analyses results indicated a number of positive selected, expansion, and unique genes had involved in DNA protection and repair process. Furthermore, these expansion genes and uniqueness had contributed to the deposit of iron in the nontoxic state in iron-rich environment adaptation. This data set will provide a valuable resource for further studies on hydrothermal vent adaptations of vent fauna.

Data Availability

The final genome assembly and other associated raw data described in this study are available on ScienceDB (https://doi.org/10.57760/sciencedb.07077). The raw sequencing reads were also deposited at NCBI under BioProject PRJNA934972.

Abbreviations

Gb: Gigabase pairs; Mb: Megabase pairs; BUSCO: Benchmarking Universal Single-Copy Orthologs; PE: Paired End; HiFi: High-Fidelity; Hi-C: High-Throughput Chromosome Conformation Capture; BLAST: Basic Local Alignment Search Tool; NCBI: National Center for Biotechnology Information; KASS: KEGG Automatic Annotation Server; KEGG: Kyoto Encyclopedia of Genes and Genomes; GO: Gene Ontology; NR: NCBI's nonredundant database; RBH: Reciprocal Best Hit; CCS: Circular Consensus Sequencing; bp: Base Pairs; GC: Guanine Cytosine; LINE: Long Interspersed Nuclear Element; LTR: Long Terminal Repeat; SINE: Short Interspersed Nuclear Elements; FDR: False Discovery Rate.

Additional Files

Supplementary Figure S1. BUSCO completeness assessment of gene sets from 10 genomes in phylogenomic analysis.

Supplementary Table S1. Data sets used for the 10 species in phylogenomic analysis.

Supplementary Table S2. Statistics of raw sequencing data.

Supplementary Table S3. Statistics of repetivite elements percentage in related echinoderms.

Supplementary Table S4. GO and KEGG enrichment of expanded gene families of *Chiridota heheva*.

Supplementary Table S5. GO and KEGG enrichment of positively selected genes of *Chiridota heheva*.

Supplementary Table S6. GO and KEGG enrichment of unique genes of *Chiridota heheva*.

Competing Interests

The authors declare that they have no competing interests.

Funding

This study was financially supported by the major scientific and technological projects of Hainan Province (ZDKJ2019011), Strategic Priority Research Program of the Chinese Academy of Sciences (CAS) (XDA22050303), National Key Research

and Development Program of China (2016YFC0304905).

Authors' Contributions

Haibin Zhang led the project. Haibin Zhang and Yujin Pu conceived this study. Yujin Pu performed the experiments by Jun Liu assistance. Yujin Pu assembled the genome and analyzed the genomic data with assistance by Yang Zhou. Yujin Pu wrote the first draft of the manuscript. All authors reviewed the manuscript.

Acknowledgments

We thank the captains, crews, and scientific staffs on the R/V *Tansuo 1*, and the pilots of HOV *Shenhaiyongshi*, for their supported by sample collection. We also thank Dr. El-Hadji Malick Cisse (Hainan University), for improving the language and grammar of this manuscript. Special thanks to the reviewers for their helpful comments and constructive suggestions on the manuscript.

References

1. Miyazaki, JI, Beppu, S, Kajio, S, et al. Dispersal ability and environmental adaptability of deep-sea mussels *Bathymodiolus* (Mytilidae: Bathymodiolinae). Open Journal of Marine Science 2013; 3(1):31-39.
2. Brazelton, W. Hydrothermal vents. Curr Biol 2017; 27(11), 450-452.
3. Sun, J, Zhang, Y, Xu, T, et al. Adaptation to deep-sea chemosynthetic environments as revealed by mussel genomes. Nat Ecol Evol 2017; 1(5): 1-7.
4. Wang, ZF, Shi, XJ, Sun, LX, et al. Evolution of mitochondrial energy metabolism genes associated with hydrothermal vent adaption of Alvinocaridid shrimps. Genes Genom 2017; 39(12), 1367-1376.
5. Cheng, J, Hui, M., Sha, ZL. Transcriptomic analysis reveals insights into deep-sea adaptations of the dominant species, *Shinkaia crosnieri* (Crustacea: Decapoda: Anomura), inhabiting both hydrothermal vents and cold seeps. BMC Genomics 2019; 20(1): 1-16.
6. Lutz, RA. Hydrothermal vent fauna. Reference module in Encyclopedia of Ocean Sciences (Third Edition), 2019, 2: 715-727.
7. Snelgrove, PVR, Grassle, JF. Deep-sea fauna. Reference module in Encyclopedia of Ocean Sciences (Third Edition), 2019; 2: 706-714.
8. Methou, P, Hikosaka, M, Chen C, et al. Symbiont community composition in *Rimicaris kairei* shrimps from Indian Ocean vents with notes on Mineralogy. Appl Environ Microb 2022; 88(8): e00185-22.

9. Prakash, LS, Fernandes, SO, Ingole, B,et al. Biogeochemical Characteristics of Hydrothermal Systems in the Indian Ocean. Systems Biogeochemistry of Major Marine Biomes 2022; 285-313.

10. Cho, B, Kim, D, Bae, H, et al. Unique characteristics of the exoskeleton of bythograeid crab, *Austinograea rodriguezensis* in the Indian Ocean hydrothermal vent (Onnuri vent field). Integr Comp Biol 2020; 60(1): 24-32.

11. Nakamura, K, Watanabe, H, Miyazaki, J, et al. Discovery of new hydrothermal activity and chemosynthetic fauna on the Central Indian Ridge at 18-20 °S. PLoS one 2012; 7(3): e32965.

12. Zhang, J, Sun, QL, Luan, ZD, et al. Comparative transcriptome analysis of *Rimicaris* sp. reveals novel molecular features associated with survival in deep-sea hydrothermal vent. Sci Rep-UK 2017; 7(1): 1-16.

13. Jang, SJ, Ho, PT, Jun, SY, et al. A newly discovered Gigantidas bivalve mussel from the Onnuri Vent Field in the northern Central Indian Ridge. Deep-sea Res Pt I: Oceanographic Research Papers 2020; 161: 103299.

14. Zhou, L, Cao, L, Wang, XC, et al. Metal adaptation strategies of deep-sea Bathymodiolus mussels from a cold seep and three hydrothermal vents in the West Pacific. Sci Total Environ 2020; 707:136046.

15. Warén, A, Bengtson, S, Goffredi, SK, et al. A hot-vent gastropod with iron sulfide dermal sclerites. Science 2003; 302, 1007-1007

16. Okada, S, Chen, C, Watsuji, T, et al. The making of natural iron sulfide nanoparticles in a hot vent snail. PNAS 2019; 116(41): 20376-20381.

17. Sun, J, Chen, C, Miyamoto, N, et al. The Scaly-foot snail genome and implications for the origins of biomineralised armour. Nat Commun 2020a; 11(1): 1-12.

18. Sun, J, Zhou, YD, Chen, C, et al. Nearest vent, dearest friend: biodiversity of Tiancheng vent field reveals cross-ridge similarities in the Indian Ocean. Roy Soc open Sci 2020b; 7(3): 200110.

19. Wang, K, Shen, YJ, Yang, YZ, et al. Morphology and genome of a snailfish from the Mariana Trench provide insights into deep-sea adaptation. Nat Ecol Evol 2019; 3(5), 823-833.

20. Liu, RY, Liu, J, Zhang, HB. Positive selection analysis reveals the deep-sea adaptation of a hadal sea cucumber (*Paelopatides* sp.) to the Mariana Trench. J Oceanol Limnol 2021; 39(1), 266-281.

21. Zhang, L, He, J, Tan, PP, et al. The genome of an apodid holothuroid (*Chiridota heheva*) provides insights into its adaptation to a deep-sea reducing environment. Commun Biol 2022; 5, 224.

22. Yuan, JB, Zhang, XJ, Gao, Y, et al. Adaptation and molecular evidence for convergence in decapod crustaceans from deep- sea hydrothermal vent environments. Mol Ecol 2020; 29(20): 3954-3969.

23. Shao G, He T, Mu Y, et al. The genome of a hadal sea cucumber reveals novel adaptive strategies to deep-sea environments. Iscience 2022; 105545.

24. WoRMS. https://www.marinespecies.org/aphia.php?p=taxdetails&id=123083. Accessed 11 October 2022.

25. Thomas, EA, Liu, RY, Amon, D, et al. *Chiridota heheva*-the cosmopolitan holothurian. Mar Biodivers 2020; 50(6): 1-13.

26. Sun, SE, Sha, ZL, Xiao N. The first two complete mitogenomes of the order Apodida from deep-sea chemoautotrophic environments: New insights into the gene rearrangement, origin and evolution of the deep-sea sea cucumbers. Comparative Biochemistry and Physiology Part D: Genomics and Proteomics, 2021; 39: 100839.

27. Humphris, SE, Fornari, DJ. Hydrothermal vents in an unusual geotectonic setting: the Kairei and Edmond vent fields, Central Indian Ridge[C]//AGU Fall Meeting Abstracts. 2001, 2001: OS41A-0444.

28. Wang, YJ, Han, XQ, Petersen, S, et al. Trace metal distribution in sulfide minerals from Ultramafic-Hosted hydrothermal systems: examples from the Kairei vent field, central indian ridge. Minerals 2018; 8(11):526.

29. Xia, YM, Chen, FS, Du, Y, et al. A modified SDS-based DNA extraction method from raw soybean. Bioscience Rep 2019; 39(2):BSR20182271.

30. Belton, J-M, McCord, RP, Gibcus, JH, et al. Hi-C: A comprehensive technique to capture the conformation of genomes. Methods 2012; 58(3), 268-276.

31. Cheng, H, Concepcion, GT, Feng, X, et al. Haplotype-resolved de novo assembly using phased assembly graphs with hifiasm. Nat Methods 2021; 18, 170-175.

32. Guan, DF, McCarthy, SA, Wood, J, et al. Identifying and removing haplotypic duplication in primary genome assemblies. Bioinformatics 2020; 36(9): 2896–2898.

33. Durand, NC, Robinson, JT, Shamim, MS, et al. Juicebox Provides a Visualization System for Hi-C Contact Maps with Unlimited Zoom. Cell Syst 2016; 3(1), 99-101.

34. Dudchenko, O, Batra, SS, Omer, AD, et al. De novo assembly of the Aedes aegypti genome using Hi-C yields chromosome-length scaffolds . Science 2017; 356(6333): 92-95.

35. Durand, NC, Shamim, MS, Machol, I, et al. Juicer provides a one-click system for analyzing loop-resolution Hi-C experiments. Cell Syst 2016; 3(1), 95-98.

36. Manni, M, Berkeley, MR, Seppey, M, et al. BUSCO update: Novel and streamlined workflows along with broader and deeper phylogenetic coverage for scoring of eukaryotic, prokaryotic, and viral genomes. Mol Biol Evol 2021; 38(10):4647-4654.

37. Flynn, JM, Hubley, R, Goubert, C, et al. RepeatModeler2 for automated genomic discovery of transposable element families. PNASciences 2020; 117(17), 9451–9457.

38. Tarailo-Graovac, M, Chen N. Using RepeatMasker to identify repetitive elements in genomic sequences. Curr Protoc Bioinform. 2009; 25(1):4.10.1-4.10.14

39. Stanke, M, Diekhans, M, Baertsch, R, et al. Using native and syntenically mapped cDNA alignments to improve de novo gene finding. Bioinformatics 2008; 24(5), 637-644.

40. Majoros, WH, Pertea, M, Salzberg, SL. TigrScan and GlimmerHMM: two open source ab initio eukaryotic gene-finders. Bioinformatics 2004;

20(16):2878-2879.

41. Alioto, T, Blanco, E, Parra, G, et al. Using geneid to Identify Genes. Current Protocols in Bioinformatics 2018; e56.

42. Slater, GS, Birney, E. Automated generation of heuristics for biological sequence comparison. BMC Bioinformatics 2005; 6:31.

43. Haas, BJ, Zeng, Q, Pearson, MD, et al. Approaches to Fungal Genome Annotation. Mycology 2011; 2(3):118-141.

44. Haas, BJ, Salzberg, SL, Zhu, W, et al. Automated eukaryotic gene structure annotation using EVidenceModeler and the Program to Assemble Spliced Alignments. Genome Biol 2008; 9(1), R7.

45. Buchfink, B, Reuter, K, Drost, H-G. Sensitive protein alignments at tree-of-life scale using DIAMOND. Nat Methods 2021;18(4):366-8.

46. Jones, P, Binns, D, Chang, H-Y, et al. InterProScan 5: genome-scale protein function classification. Bioinformatics 2014; 30(9), 123-1240.

47. Emms, DM, Kelly, S. OrthoFinder: phylogenetic orthology inference for comparative genomics. Genome Biol 2019; 20(1).

48. Katoh, K., Standley, D. M. MAFFT Multiple sequence alignment software version 7: Improvements in performance and usability. Mol Biol Evol 2013; 30(4), 772-780.

49. Stamatakis, A. RAxML version 8: a tool for phylogenetic analysis and post-analysis of large phylogenies. Bioinformatics 2014; 30(9), 1312-1313.

50. Yang, Z. PAML 4: Phylogenetic analysis by maximum likelihood. Mol Biol and Evol 2007; 24(8), 1586-1591.

51. Wang, YX, Yang, YJ, Li, YL, et al. Identification of sex determination locus in sea cucumber *Apostichopus japonicus* using genome-wide association study. BMC Genomics 2022; 23, 391.

52. Camacho, C, Coulouris, G, Avagyan, V, Ma, et al. BLAST+: architecture and applications. BMC Bioinformatics 2009; 10:421.

53. Tang, HB, Krishnakumar V, Li, J. jcvi: JCVI utility libraries. Zenodo 2015.

54. De Bie, T, Cristianini, N, Demuth, JP, et al. CAFE: a computational tool for the study of gene family evolution. Bioinformatics 2006; 22(10), 1269-1271.

55. Barker, MS, Dlugosch, KM, Dinh L, et al. EvoPipes.net: Bioinformatic tools for ecological and evolutionary genomics. Evol Bioinform 2010; 6.

56. Capella-Gutiérrez, S, Silla-Martínez, JM, Gabaldón, T. trimAl: a tool for automated alignment trimming in large-scale phylogenetic analyses. Bioinformatics 2009; 25(15):1972-3.

57. Chen, CJ, Chen, H, Zhang Y, et al. TBtools: an integrative toolkit developed for interactive analyses of big biological data. Mol plant 2020; 13(8): 1194-1202.

58. Jo, J, Oh, J, Lee, HG, et al. Draft genome of the sea cucumber *Apostichopus japonicus* and genetic polymorphism among color variants. Gigascience 2017; 6(1): giw006.

59. Reich M. Different pathways in early evolution of the holothurian calcareous ring. Progress in Echinoderm Palaeobiology 2015; 19: 137-145.

60. Reich M. The early evolution and diversification of holothurians (Echinozoa). Echinoderms: Durham: Taylor and Francis Group, London, 2010a: 55-59.

61. Reich M. The oldest synallactid sea cucumber (Echinodermata: Holothuroidea: Aspidochirotida). Paläontologische Zeitschrift 2010b; 84(4): 541-546.

62. Pierrat, J, Bédier, A, Eeckhaut, I, et al. Sophistication in a seemingly simple creature: a review of wild holothurian nutrition in marine ecosystems. Biol Rev 2022; 97(1): 273-298.

63. Siebenaller, JF, Garrett, DJ. The effects of the deep-sea environment on transmembrane signaling. Comparative Biochemistry and Physiology Part B: Biochemistry and Molecular Biology 2002; 131(4): 675-694.

64. Koyama, S, Kobayashi, H, Inoue, A, et al. Effects of the piezo-tolerance of cultured deep-sea eel cells on survival rates, cell proliferation, and cytoskeletal structures. Extremophiles 2005; 9(6): 449-460.

65. Gan, ZB, Yuan, JB, Liu, XM, et al. Comparative transcriptomic analysis of deep-and shallow-water barnacle species (Cirripedia, Poecilasmatidae) provides insights into deep-sea adaptation of sessile crustaceans. BMC genomics 2020; 21(1): 1-13.

Figure legends

Figure 1. The sampling site at the Kairei vent filed of Indian Ocean and the photo in situ at a depth of 2428 m.

Figure 2. Genome assembly and sequencing analysis of *Chiridota heheva*. (A) Hi-C interaction heat map. (B) High-quality assembly of 19 chromosomes with genes coverage, GC content, and repetitive elements of LTR, LINE and SINE.

Figure 3. Phylogenetic and syntenic relationships. (A) Phylogenetic relationship and divergence time in 10 metazoan species. The number on the branches represents of gene family expansion (red) or contraction (green) (B) Statistics of orthologous gene numbers in these species. Single-copy orthologs, gene that have only one copy in each species and have homologs in other species; Multiple-copy orthologs, gene that have more than one copy in each species, together with homologs in other species; Unique orthologs, gene in each species without homologs in other species; Other orthologs, orthologs that do not belong to any type of the above orthologs; Unclustered genes, gene that do not clustered. (C) Synteny between *Chiridota heheva* and *Apostichopus japonicus* in dot plotter.

Figure 4. GO enrichment analysis of expanded gene families of *Chiridota heheva*.

Figure 5. Positively selected amino acid sites of gene *RFC2* in *Chiridota heheva*. (A) 10 positively selected amino sites in protein sequence. (B) Distribution of 10 positively selected amino sites in three dimensional structure from AlphaFold.

Figure 6. Unique genes between Kairei vent *Chiridota heheva*, Haima cold seep *Chiridota heheva*, and *Apostichopus japonicus*. (A) Shared and unique genes in KEGG annotation. (B) Shared and unique genes in GO annotation. (C) GO

enrichment analysis of unique genes of *Chiridota heheva*.

Table 1. Assembly statistics of the *Chiridota heheva* genome assembly.

**Assembly statistics**	**Value**
Genome size (bp)	1,434,753,151
Number of scaffolds	1399
Number of chromosome-scale scaffolds	19
N50 of contigs (bp)	370,957
N50 of scaffolds (bp)	53,240,875
L50 of scaffolds	11
Chromosome-scale scaffolds (bp)	1,431,787,880
GC content of the genome (%)	37.1231
Error rate	0.00206
**BUSCO analysis**	
Library	Metazoan_odb10 (954)
Complete	92.40% (881)
Complete and single copy	91.10% (869)
Complete and duplicated	1.30% (12)
Fragmented	3.00% (29)
Missing	4.60% (44)

Table 2. Repeatitive elements of the Chiridota heheva genome assembly.

**Assembly feature**	**Number of elements**	**Value (bp)**
DNA	15,768	87,173,116
LINE	305,867	353,178,525
SINE	63,461	11,551,395
LTR	16,380	23,197,590
Low complexity	27,548	1,906,077
Satellite	44,353	22,459,277
Simple repeat	365,433	399,894,357
Small RNA	18,648	2,502,674
Total	70.8%	1,016,101,549
Unknown	1,635,141	380,635,876

Table 3. Annotation statistics of *Chiridota heheva* genome assembly.

**Databases of genes annotation**	**Value**
Number of predicted genes	32,434
Number of predicted protein-coding genes	24,606
Number of genes annotation to Interproscan	16,086
Number of genes annotation to GO	10,566
Number of genes annotation to Pfam	14,171
Number of genes annotation to KEGG	7,244

Number of genes annotation to NR	18,038
Number of genes annotation to Swiss-Prot	10,711
Number of genes annotation toTrEMBL	17,697
**BUSCO analysis**	
Complete	95.0% (907)
Complete and single copy	94.2% (899)
Complete and duplicated	0.8% (8)
Fragmented	1.5% (14)
Missing	3.5% (33)
Total BUSCO groups searched	954

Table 4. The positively selected genes of *Chiridota heheva* from the Kairei vent.

**Gene**	**Description**	**FDR**
MAEA	Macrophage erythroblast attacher	2.99E-04
POLB	DNA polymerase beta	2.99E-04
SOD1	Superoxide dismutase, Cu-Zn family	2.99E-04
URB1	Nucleolar pre-ribosomal-associated protein 1	1.21E-03
FAN1	Fanconi-associated nuclease 1	1.21E-03
RFC2	Replication factor C subunit 2	1.87E-03
FARSA	Phenylalanyl-tRNA synthetase alpha chain	4.87E-03
NUP88	Nuclear pore complex protein Nup88	5.02E-03
KDM2A	F-box and leucine-rich repeat protein 11	5.16E-03
RFT1	Oligosaccharide translocation protein *RFT1*	8.10E-03
RNF216	E3 ubiquitin-protein ligase *RNF216*	1.08E-02
SPG7	Spastic paraplegia 7	1.40E-02
BBOX1	Gamma-butyrobetaine dioxygenase	1.40E-02
RPS16	Small subunit ribosomal protein S16e	1.40E-02
BRCA1	Breast cancer type 1 susceptibility protein	1.60E-02
SDR42E1	Short-chain dehydrogenase/reductase family 42E member 1	2.05E-02
SSF1_2	Ribosome biogenesis protein *SSF1/2*	2.37E-02
LSM4	U6 snRNA-associated Sm-like protein LSm4	2.64E-02
PSTK	O-phosphoseryl-tRNA(Sec) kinase	3.13E-02
ESCO1	N-acetyltransferase	3.14E-02
TTLL9	tubulin polyglutamylase *TTLL9*	3.20E-02
HOGA1	4-hydroxy-2-oxoglutarate aldolase	3.43E-02
DCLK1	Doublecortin-like kinase 1	3.80E-02
LDHD	D-lactate dehydrogenase (cytochrome)	4.14E-02
TEP1	telomerase protein component 1	4.14E-02
SIRT4	NAD+-dependent protein deacetylase sirtuin 4	4.14E-02
SLC35F5	Solute carrier family 35, member F5	4.22E-02
SEH1	Nucleoporin *SEH1*	4.47E-02

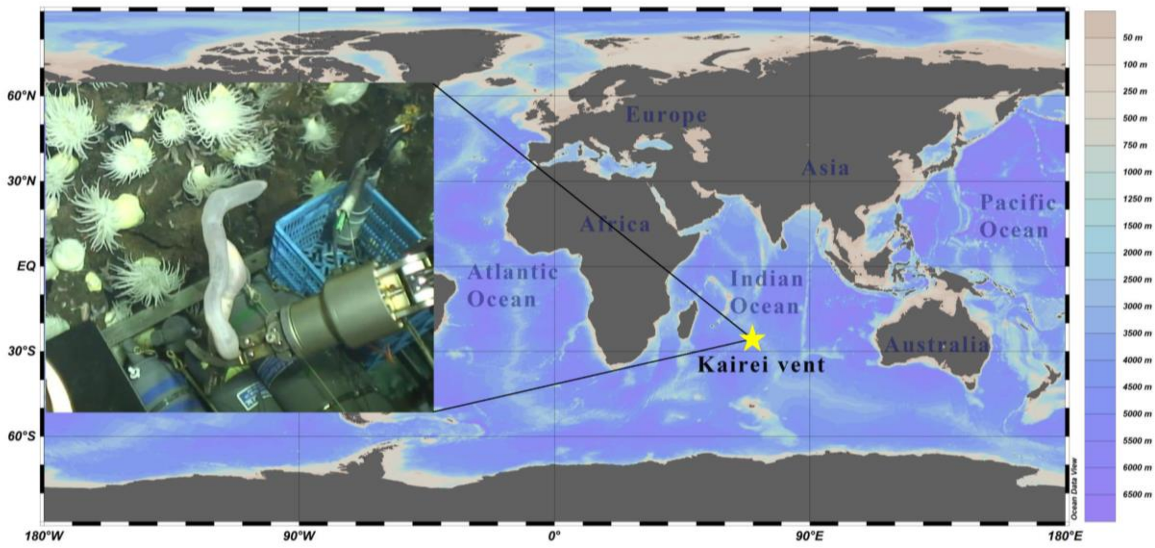

Figure 1

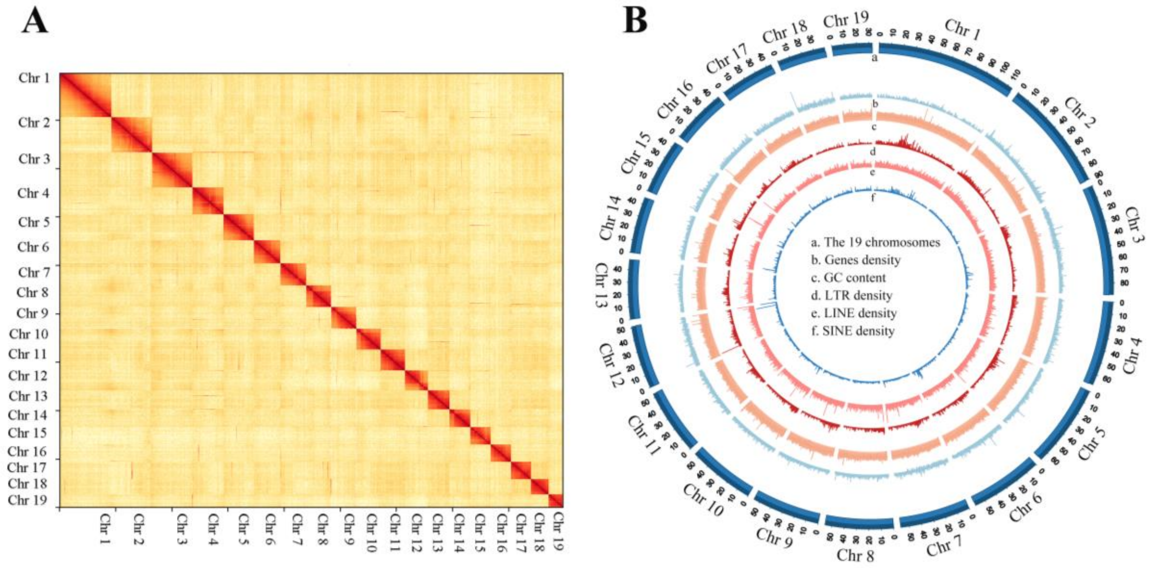

Figure 2

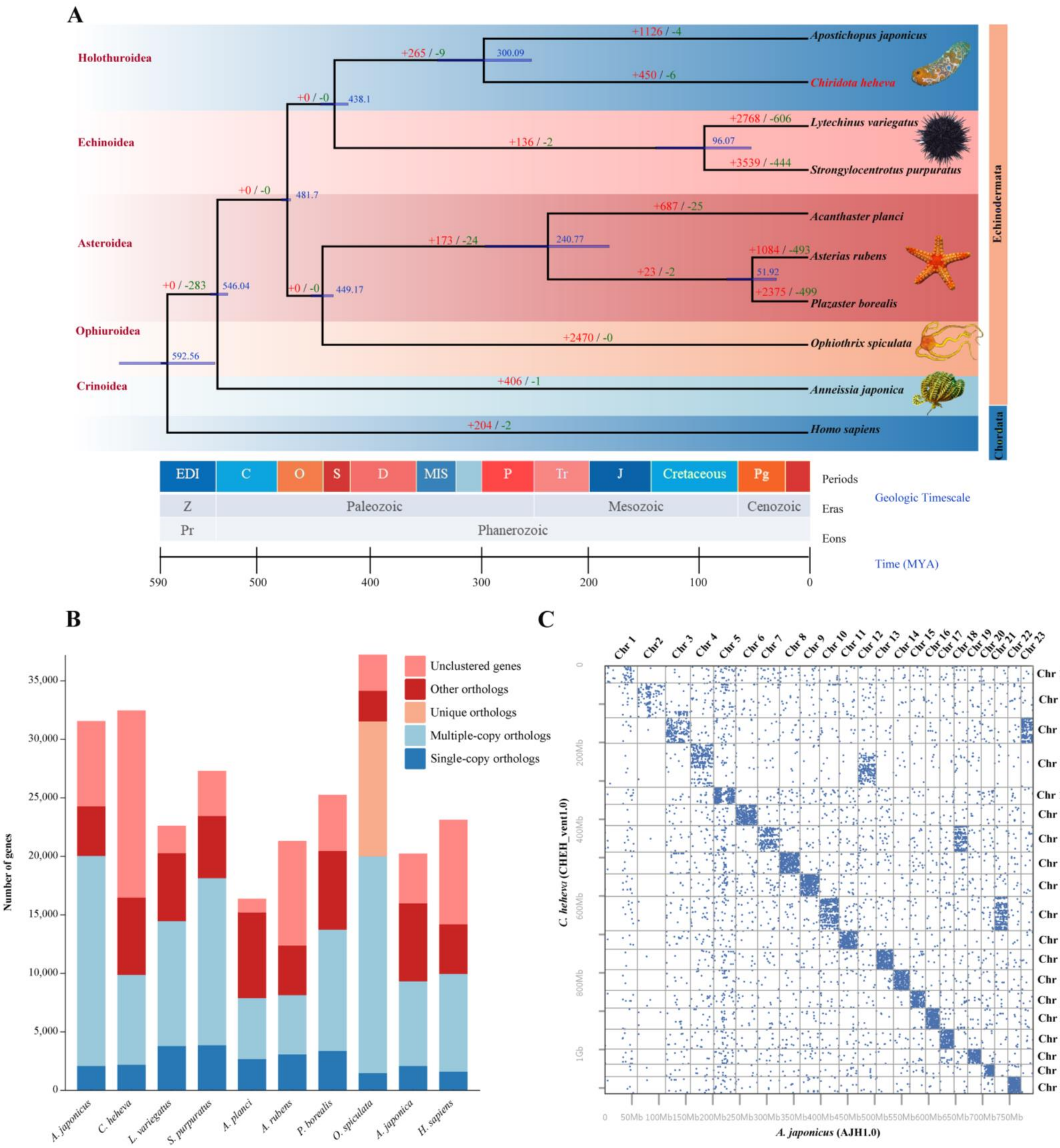
A
Holothuroidea
Echinoidea
Asteroidea
Ophiuroidea
Crinoidea
Apostichopus japonicus
Chiridota heheva
Lytechinus variegatus
Strongylocentrotus purpuratus
Acanthaster planci
Asterias rubens
Plazaster borealis
Ophiothrix spiculata
Anneissia japonica
Homo sapiens
Echinodermata
Chordata
+1126 / -4
+265 / -9
300.09
+450 / -6
+0 / -0
438.1
+2768 / -606
+136 / -2
96.07
+3539 / -444
+0 / -0
481.7
+687 / -25
+173 / -24
240.77
+1084 / -493
+23 / -2
51.92
+2375 / -499
+0 / -283
546.04
+0 / -0
449.17
+2470 / -0
592.56
+406 / -1
+204 / -2
EDI
C
O
S
D
MIS
P
Tr
J
Cretaceous
Pg
Periods
Z
Paleozoic
Mesozoic
Cenozoic
Eras
Geologic Timescale
Pr
Phanerozoic
Eons
Time (MYA)
590
500
400
300
200
100
0
B
Number of genes
35,000
30,000
25,000
20,000
15,000
10,000
5,000
0
Unclustered genes
Other orthologs
Unique orthologs
Multiple-copy orthologs
Single-copy orthologs
A. japonicus
C. heheva
L. variegatus
S. purpuratus
A. planci
A. rubens
P. borealis
O. spiculata
A. japonica
H. sapiens
C
Chr 1
Chr2
Chr 3
Chr 4
Chr 5
Chr 6
Chr 7
Chr 8
Chr 9
Chr 10
Chr 11
Chr 12
Chr 13
Chr 14
Chr 15
Chr 16
Chr 17
Chr 18
Chr 19
Chr 20
Chr 21
Chr 22
Chr 23
Chr 15
Chr 2
Chr 5
Chr 1
Chr 16
Chr 9
Chr 4
Chr 7
Chr 6
Chr 3
Chr 13
Chr 11
Chr 10
Chr 14
Chr 8
Chr 12
Chr 18
Chr 19
Chr 17
C. heheva (CHEH_vent1.0)
0
200Mb
400Mb
600Mb
800Mb
1Gb
0
50Mb
100Mb
150Mb
200Mb
250Mb
300Mb
350Mb
400Mb
450Mb
500Mb
550Mb
600Mb
650Mb
700Mb
750Mb
A. japonicus (AJH1.0)

Figure 3

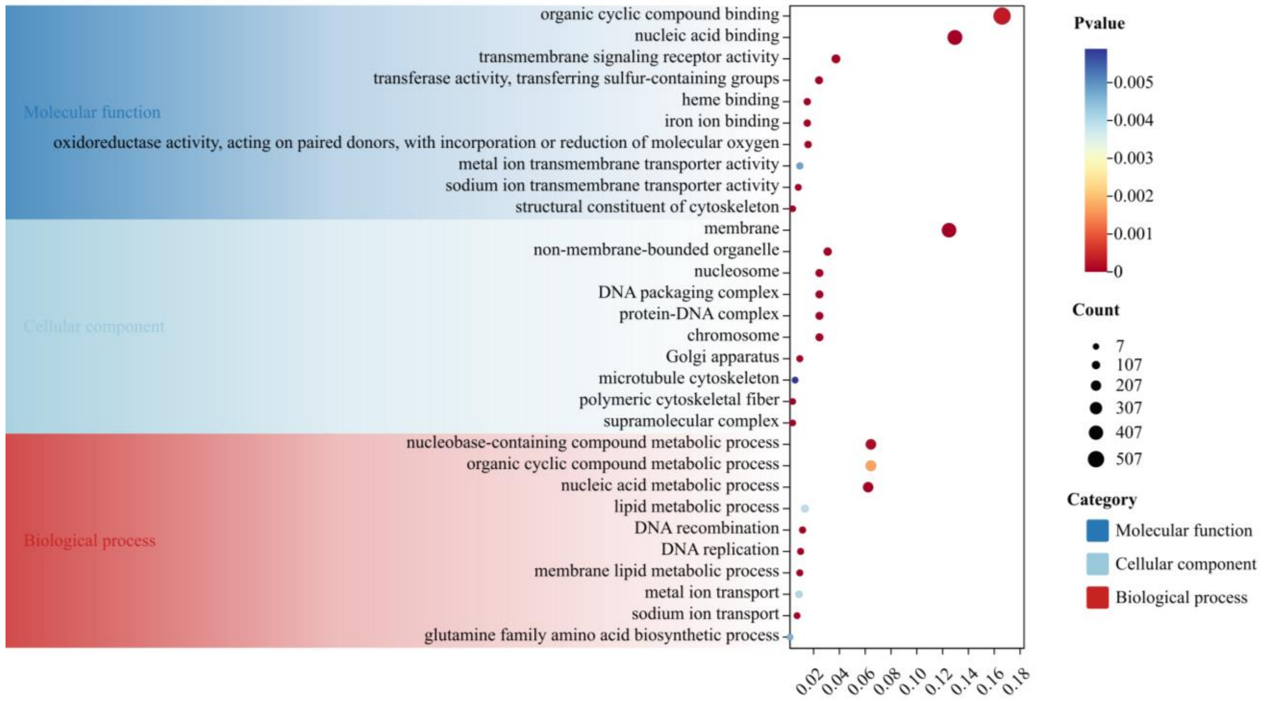

Figure 4

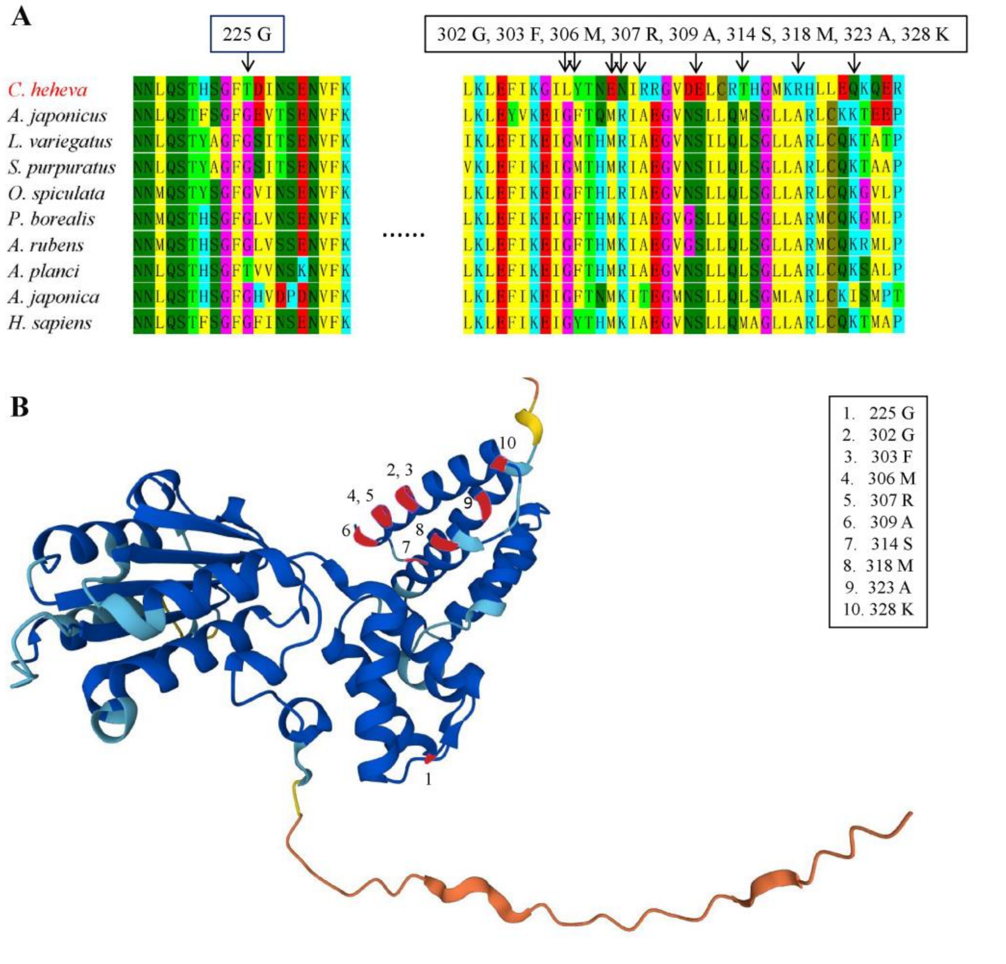

Figure 5

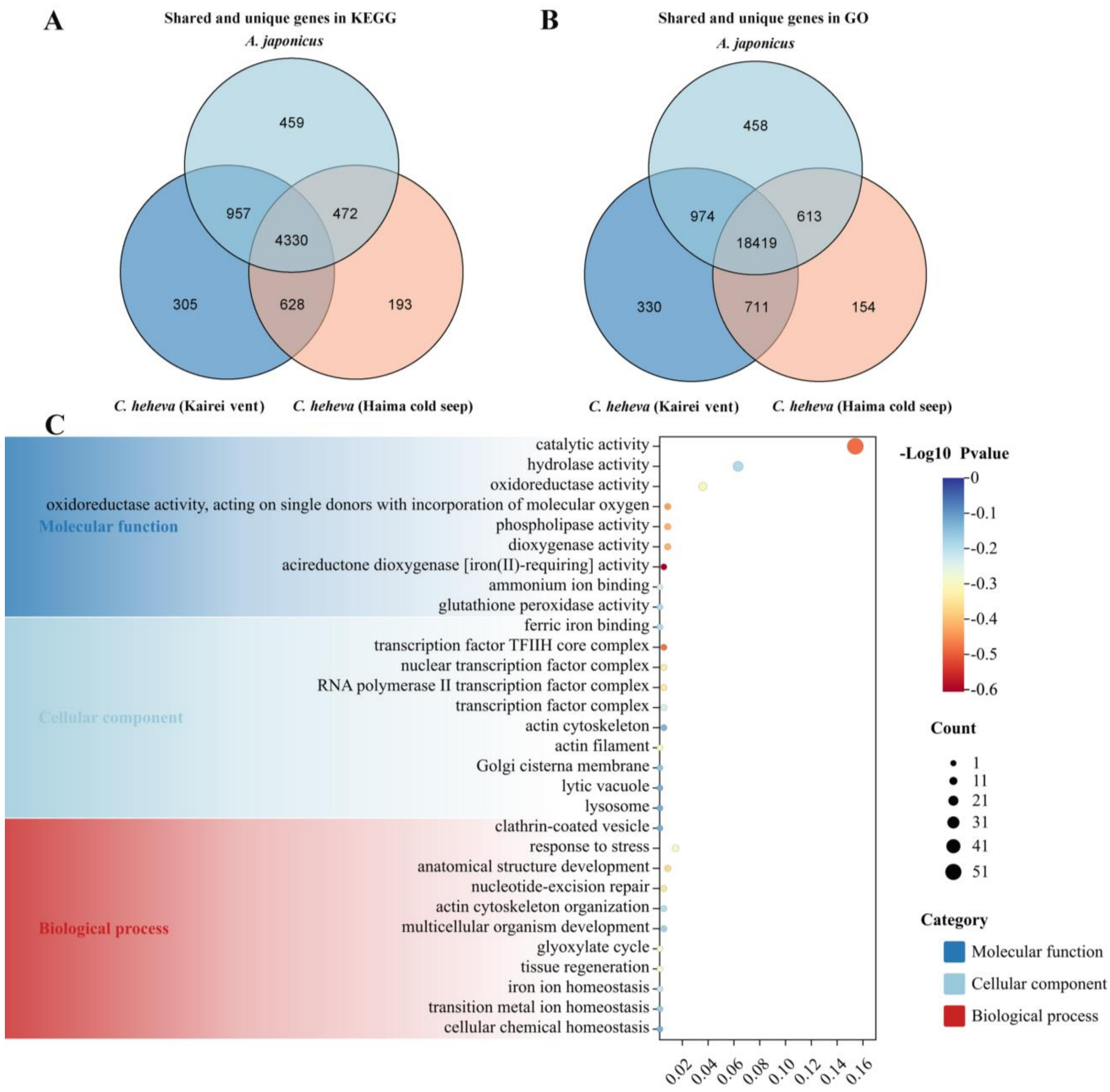
A
Shared and unique genes in KEGG
A. japonicus
459
957
472
4330
305
628
193
C. heheva (Kairei vent)
C. heheva (Haima cold seep)
B
Shared and unique genes in GO
A. japonicus
458
974
613
18419
330
711
154
C. heheva (Kairei vent)
C. heheva (Haima cold seep)
C
Molecular function
Cellular component
Biological process
catalytic activity
hydrolase activity
oxidoreductase activity
oxidoreductase activity, acting on single donors with incorporation of molecular oxygen
phospholipase activity
dioxygenase activity
acireductone dioxygenase [iron(II)-requiring] activity
ammonium ion binding
glutathione peroxidase activity
ferric iron binding
transcription factor TFIIH core complex
nuclear transcription factor complex
RNA polymerase II transcription factor complex
transcription factor complex
actin cytoskeleton
actin filament
Golgi cisterna membrane
lytic vacuole
lysosome
clathrin-coated vesicle
response to stress
anatomical structure development
nucleotide-excision repair
actin cytoskeleton organization
multicellular organism development
glyoxylate cycle
tissue regeneration
iron ion homeostasis
transition metal ion homeostasis
cellular chemical homeostasis
0.02
0.04
0.06
0.08
0.10
0.12
0.14
0.16
-Log10 Pvalue
0
-0.1
-0.2
-0.3
-0.4
-0.5
-0.6
Count
1
11
21
31
41
51
Category
Molecular function
Cellular component
Biological process

Figure 6

Click here to access/download
Supplementary Material
Supplementary Fig. S1.png

Click here to access/download
Supplementary Material
Supplementary Table S1..xlsx

Click here to access/download
Supplementary Material
Supplementary Table S2..xlsx

Click here to access/download
Supplementary Material
Supplementary Table S3..xlsx

Click here to access/download
Supplementary Material
Supplementary Table S4..xlsx

Click here to access/download
Supplementary Material
Supplementary Table S5.xlsx

Click here to access/download
Supplementary Material
Supplementary Table S6.xlsx
