# Supplementary material for: A high-quality chromosomal genome assembly of the sea cucumber Chiridota heheva and its hydrothermal adaptation
Source: Gigascience. 2024 Jan 4;13:giad107. doi: 10.1093/gigascience/giad107 (PMC10764150; doi:10.1093/gigascience/giad107)
Supplement: giad107_GIGA-D-23-00018_Revision_1 [file giad107_giga-d-23-00018_revision_1.pdf]

GigaScience

A high-quality chromosomal genome assembly of the sea cucumber Chiridota heheva and its hydrothermal adaptation

--Manuscript Draft--

**Manuscript Number:**	GIGA-D-23-00018R1
**Full Title:**	A high-quality chromosomal genome assembly of the sea cucumber Chiridota heheva and its hydrothermal adaptation
**Article Type:**	Data Note
**Funding Information:**	the major scientific and technological projects of Hainan Province (ZDKJ2019011) — professor Haibin Zhang<br>Strategic Priority Research Program of the Chinese Academy of Sciences (CAS) (XDA22050303) — Prof. Jun Liu<br>National Key Research and Development Program of China (2016YFC0304905) — Prof. Jun Liu
**Abstract:**	Abstract<br>Background: Chiridota heheva is one of the cosmopolitan holothurian, which has been found well adapted to diverse deep-sea ecosystems, especially chemosynthetic environments. Besides high hydrostatic pressure and limited light, high concentrations of metal ions also represent harsh conditions in hydrothermal environment. Few holothurian species can live in such extreme condition. Therefore, it is valuable to elucidate the adaptive genetic mechanisms of C. heheva to hydrothermal environment.<br>Findings: Herein we report a high-quality reference genome assembly of C. heheva from the Kairei vent, which is the first chromosome-level genome of Apodida. The chromosome-level genome size was 1.43 Gb, with a scaffold N50 of 53.24 Mb and BUSCO completeness score of 94.5 %. Contig sequences were clustered, ordered, and assembled into 19 natural chromosome. Comparative genome analysis found that the expanded gene families and positively selected genes of C. heheva were involved in DNA damage repair process, and the expanded genes and the unique genes were contributed to maintaining the iron homeostasis in iron-enriched environment. Positively selected gene RFC2 with 10 positively selected sites was played an essential role in DNA repair under the extreme environment.<br>Conclusions: This first chromosome-level genome assembly of C. heheva reveals the hydrothermal adaptation of holothurians. As the first chromosome-level genome of Order Apodida, this genome will provide the resource for investigating the evolution of Class Holothuroidea.
**Corresponding Author:**	Haibin Zhang, Ph.D<br>Institute of Deep-sea Science and Engineering Chinese Academy of Sciences<br>Sanya, Hainan CHINA
**Corresponding Author Secondary Information:**	
**Corresponding Author's Institution:**	Institute of Deep-sea Science and Engineering Chinese Academy of Sciences
**Corresponding Author's Secondary Institution:**	
**First Author:**	Yujin Pu
**First Author Secondary Information:**	
**Order of Authors:**	Yujin Pu<br>Yang Zhou<br>Jun Liu<br>Haibin Zhang, Ph.D

Powered by Editorial Manager® and ProduXion Manager® from Aries Systems Corporation

**Order of Authors Secondary Information:**	
**Response to Reviewers:**	Response to Reviewers<br><br>Reviewer #1: Pu et al. reported a chromosome-level genome assembly of C. heheva collected from hydrothermal vent. The authors dissected the genetic basis of adaptation in vent-dwelling C. heheva by performing protein family expansion/contraction and positively selected gene analysis. Here are my comments of the manuscript:<br><br>1.There are many grammatical problems in the manuscript. These include errors in tenses, verb conjugation, and definite and indefinite articles. Please check the grammar of the entire manuscript again.<br><br>We have carefully revised the manuscript according to the reviewers' comments and suggestions, and also have re-scrutinized to polish the grammar with the help of a friendly native English speaker. These changes of grammar revision will not influence the content and framework of this paper. Here we did not list the changes but marked in blue in the revised manuscript.<br><br>2.Line 279, I am not convinced that expansion of protein family related to lipid metabolism suggested vent-dwelling C. heheva had more fatty acid in the membrane. Fatty acid is just one kind of lipid.<br><br>Thank you for pointing out this issue. According to the comment, we have added more information about the compositions of membrane, lipid metabolisms, and membrane fluidity in the revised manuscript (lines 258-276). Besides the revised part in the revised manuscript, supplementary figure S2 was made for clear understanding of essential fatty acid metabolisms.<br><br>3.Line 282, specify what are toxic iron and safe iron status.<br><br>Thank you for pointing out this issue. The redox-inert iron are in the safe form, which often bound to proteins or stored in ferritin. The redox-active iron are in the potentially toxic and fatal form, through the excess redox-active iron could generate reactive oxygen species (ROS) and lead to cell damages. We have re-written this part and added more information in the revised manuscript (lines 28-287).<br><br>4.Line 283, specify what are "These expanded gene families".<br><br>Thank you for pointing out this issue. “These expanded gene families” are the enriched gene families of iron ion binding and metal ion transport. We have re-written this part in the revised manuscript (lines 287-289).<br><br>5.Line 289, What is the relationship between DNA-protected abilities and DNA replication, recombination, DNA-associated protein? I am not convinced that expansion of protein family related to DNA replication implied vent-dwelling C. heheva had better ability in DNA protected ability.<br><br>We are very sorry for our incorrect using of “DNA-protected abilities” in here. We have modified this part in the revised manuscript (lines 293-295).<br><br>6.Line 301, how did "deep-sea fauna obtained PSGs through adaptive evolution"? Gene were positively selected during adaptive evolution.<br><br>Thank you for pointing out this issue. Actually, natural selection, both positive and negative, is pervasive in many genomes. The positive selection of amino acid site may causes changes in genetic traits, which changes of positively selected genes lead to well adaption to deep-sea environment through molecular adaptive evolution. We have re-written this part in the revised manuscript (lines 305-306).<br><br>7.Line 307, explain how genes "mainly induced by high hydrostatic pressure".<br><br>Thank you for pointing out this issue. The temperature of the chimneys is extremely high (~ 350 °C), and mix directly with ambient seawater (~ 2°C). Although the sample

Powered by Editorial Manager® and ProduXion Manager® from Aries Systems Corporation

of this study was collected next to the chimney, the temperature of this sample may cold as ambient sea water. The effects of high hydrostatic pressure on DNA are often difficult to disentangle from effects caused by low temperature that are often characteristic of the deep sea. For the hydrothermal vent, DNA damages may causes by high hydrostatic pressure, low temperature, and other toxic components that emitted with fluid.
Therefore, we have deleted " that mainly induced by high hydrostatic pressure" in the revised manuscript.

8.Line 323, terms cannot be enriched to adapt the iron-rich environment. Only organisms can adapt to a specific environment.

Thank you so much for your careful check and your suggestion. All the part of unique genes evolution have re-written in the revised manuscript.

9. Line 330, explain the relationship between the unique genes and DNA repair. I don't think that the unique genes identified in the study are related to DNA repair.

Thank you for pointing out this issue. Some of the unique genes, such as TFIIH, SSB, and RAD51, are enriched in categories related to DNA repair. All the part of unique genes evolution have re-written in the revised manuscript.

Reviewer #2: In this manuscript, Pu et al present a chromosome-level genome assembly of the holothurian Chiridota heheva. The assembly and annotation process was carried out with an up-to-date sequencing and methodological strategy and has resulted in a high quality genome assembly of this species, such that two assemblies of these species are now available. This assembly has the nice addition of HiC scaffolding which has resulted in chromosome-length scaffolds. This work is accompanied by gene family expansion analyses and tests for positive selection in coding sequence to identify putatively adaptive changes in genes associated with living in hydrothermal environments.

For clarity, I've organized my comments into two sections: 1) Rational and Significance and 2) Methods and Results. These comments each have major and minor components. Overall, while the assembly itself is nice and now available at a chromosome-level, I think the description of the rationale of this study and how it is distinguished from the already published assembly is needed. As written, the ability to discern hydrothermal adaptations from a more general suite of adaptations associated with the deep sea is unclear. In addition, more methodological detail and revision is needed with each of the functional genomic analyses, and reconciling these results with those already published will be helpful in revising their interpretation.

Rational and Significance

Major Comments

1.The assembly reported in this manuscript represents the second assembly for this species. The rationale for generating this assembly is stated to be that the individual C. heheva was collected from population inhabiting the Kairei hydrothermal vent (herein C. heheva K) whereas the earlier published version of this species genome was from an individual collected from a population inhabiting a different deep sea environment, a cold seep (herein C. heheva H: Zhang et al, 2022 Comms Bio). However, C. heheva is a cosmopolitan holothurian found in several harsh deep sea environments, so it is not clear whether any genetic-signatures of adaptations detected in this assembly are truly associated with hydrothermal vent adaptation or not. Given these individuals (K and H) represent the same species, presently it is difficult to say whether the genome assembly of single individual is an appropriate measure of local adaption to hydrothermal vent habitats unless these populations been isolated for a very long amount of time (which should be reported). Otherwise, population genetic data would be needed in this case. It is possible that this species is instead adapted to harsh deep sea environments broadly-sepaking, so the utility of comparisons between single individuals is difficult to interpret. Given a genome assembly of this species is already published, more information justifying the rational of this study is needed in this regard.

Powered by Editorial Manager® and ProduXion Manager® from Aries Systems Corporation

We gratefully appreciate for your careful check and valuable suggestion.
In the one hand, chiridotid holothurians are often the most frequently observed in the hydrothermal vent according to previous reports (Zhou et al., 2018; Sun et al., 2020; Thomas et al., 2020; Sun et al., 2021; Prakash et al., 2022). This point was mentioned in the manuscript (lines 86-87). That indicates our sample of C. heheva that collected from the Kairei vent is not occasional.
In the other hand, Indian Ocean is well-known as the hydrothermal fields including Dodo, Solitaire, Edmond, Kairei, Longqi, Tiancheng, Pelagia, Wocan, Onnuri, Old City, Sonne, Yokoniwa, Mount Jourdanne, Tianzuo, Yuhuang-1, Duanqiao-1, and so on (Nakamura et al., 2012; Prakash et al., 2022). Besides the hydrostatic pressure, the high contents of chemicals, which diffusing in from the surrounding vent fluid, is the principal characters of hydrothermal vent. The effects of extreme hydrothermal vent conditions are often difficult to disentangle from effects caused by hydrostatic pressure that are often characteristic of the deep sea. Iron-rich condition is one of the signatures in the Kairei vent, the iron-rich adaptation has observed in the previous studies (Warén et al., 2003; Nakamura et al., 2012; Okada et al., 2019). In our study, we found some evidences for iron-rich adaptation in gene families enrichment and the unique genes. These evidences could be found in gene family evolution and unique genes evolution of results part in the manuscript.
Nevertheless, samples of C. heheva that collected from hydrothermal vent are limited up to now. The observation of population adaptation for hydrothermal vent is required in the further study.

The main references in here.
Zhou, Y, Zhang, DS, Zhang, RY, et al. Characterization of vent fauna at three hydrothermal vent fields on the Southwest Indian Ridge: Implications for biogeography and interannual dynamics on ultraslow-spreading ridges. Deep Sea Research Part I: Oceanographic Research Papers, 2018; 137: 1-12.
Thomas, EA, Liu, RY, Amon, D, et al. Chiridota heheva-the cosmopolitan holothurian. Mar Biodivers 2020; 50(6): 1-13.
Sun, J, Zhou, YD, Chen, C, et al. Nearest vent, dearest friend: biodiversity of Tiancheng vent field reveals cross-ridge similarities in the Indian Ocean. Roy Soc open Sci 2020b; 7(3): 200110.
Sun, SE, Sha, ZL, Xiao N. The first two complete mitogenomes of the order Apodida from deep-sea chemoautotrophic environments: New insights into the gene rearrangement, origin and evolution of the deep-sea sea cucumbers. Comparative Biochemistry and Physiology Part D: Genomics and Proteomics, 2021; 39: 100839.
Prakash, LS, Fernandes, SO, Ingole, B, et al. Biogeochemical characteristics of hydrothermal systems in the Indian Ocean. Systems Biogeochemistry of Major Biomes, 2022; 285-313.
Zhang, L, He, J, Tan, PP, et al. The genome of an apodid holothuroid (Chiridota heheva) provides insights into its adaptation to a deep-sea reducing environment. Commun Biol 2022; 5, 224.
Nakamura, K, Watanabe, H, Miyazaki, J, et al. Discovery of new hydrothermal activity and chemosynthetic fauna on the Central Indian Ridge at 18-20 °S. PLoS one 2012; 7(3): e32965
Okada, S, Chen, C, Watsuji, T, et al. The making of natural iron sulfide nanoparticles in a hot vent snail. PNAS 2019; 116(41): 20376-20381.
Warén, A, Bengtson, S, Goffredi, SK, et al. A hot-vent gastropod with iron sulfide dermal sclerites. Science 2003; 302, 1007.

2. Along the same lines, I think more effort is needed to distinguish this study from the previously published one of the same species in terms of the manuscript's writing. The phrasing of some of the writing is a little too close for comfort to what is already published. For example:
a. Introduction: Lines 86-90: "Chiridota heheva, a cosmopolitan Chiridotidae holothurian, has been found in hydrothermal vents, cold seeps, and other organic falls [25-26]. C. heheva may be an ideal model for studying how marine fauna have adapted to extreme deep-sea chemosynthetic environments." Vs Zhang: "Chiridota heheva Pawson & Vance, 2004 (Apodida: Chiridotidae) is one of the few echinoderms that occupies all three types of chemosynthetic ecosystems (hydrothermal vent, cold seep, and organic fall)… "The cosmopolitan distribution and special lifestyle of C. heheva make it an ideal model to study adaptation to deep-sea reducing environments in nonsymbiotic animals."

We gratefully appreciate for your valuable content and suggestion.
The main purpose of this study is reported the first chromosome-level genome of hydrothermal vent C. heheva with the preliminary investigation of hydrothermal adaptation. In this study, the adaptation for iron-rich environment was found in in the analysis of gene family evolution and unique gene evolution. It is the main point that could distinguish between hydrothermal vent C. heheva and cold seep C. heheva. Considering the suggestion, we have added some information of iron-rich adaptation in gene family evolution and unique genes evolution of the revised manuscript.

According to the comment that the close writing, we have checked the writing around our manuscript carefully and re-written some sentences we found and the one you mentioned above, in order to clean up the closed sentences. Please check the changes in the revised manuscript.

b. the titles are similar: "The chromosome-level genome of Chiridota heheva (Holothuroidea, Apodida,Chiridotidae) provides insights into adaptation to the hydrothermal environment" vs Zhang: "The genome of an apodid holothuroid (Chiridota heheva) provides insights into its adaptation to a deep-sea reducing environment"

Thank you for your suggestion. We have changed the title from “Chromosome-level genome report and relevant analysis reveals insights into deep-sea adaptation of Chiridota heheva (Holothuroidea, Apodida,Chiridotidae), inhabiting in hydrothermal environment” to “A high-quality chromosomal genome assembly of the sea cucumber Chiridota heheva and its hydrothermal adaptation” in the revised manuscript.

3. This study replicates many of the same functional genomic analyses as Zhang et al (gene family expansion, tests for positive selection) but no overlap in results are reported. More on this in the next section below, but this represents a unique opportunity to compare two independent studies of the same species to validate predictions associated with deep sea adaptation. However, these cross-study comparisons are not mentioned even though they would be an effective way of distinguishing cold seep vs hydrothermal adaptation, should they exist.

We gratefully appreciate for your valuable comment. We also consider the works as reviewer suggested that comparative analysis between the hydrothermal vent C. heheva and cold seep C. heheva, but we did not found the useful information to support the adaption in this study. We have collected some chiridotid holothurians which distributed in a variety of habitats. The comparative genomic analysis of hydrothermal vent C. heheva and cold seep C. heheva will considered in our further study, that the comparative genomic analysis of the chiridotid holothurians in three types of chemosynthetic ecosystems. We really hope you will interest in our further study.
Moreover, in compared to the C. heheva genome of Zhang et al. (2022) reported, our C. heheva genome from hydrothermal vent while Zhang’s genome from cold seep, our assembly level is chromosome while Zhang’s is contig, our genome completeness is high as 94.50 % (BUSCO) while Zhang’s completeness is 89.60 % (BUSCO). Thus, it is worth to report the chromosome-level genome assembly of hydrothermal vent C. heheva, and additional with preliminarily investigated of the vent-specific adaptation.

Minor

1. The second paragraph of the introduction is quite lengthy as it summarizes many adaptations associated with deep sea habitats. These species and adaptations list could probably be shortened, particularly for a Data Note type of manuscript.

Thank you for your nice suggestion. We have checked the second paragraph and re-written in the revised manuscript. We have tried our best to make this part clearly in short sentences.

Results and Methods

Major:

1. Tests were positive selection occurred at the species level as C. heheva was compared to several other metazoans. A very similar analysis was carried out in Zhang et al (also C heheva) yet there appears to be little to no overlap is genes with evidence of positive selection detected in both studies. I realize these animals came from different habitats, but shared species-level signatures of positive selection would be expected here, particularly in regard to deep sea adaption, broadly speaking. This large discrepancy is concerning and needs justification or revision.

Thank you for pointing out this issue. We have noted this issue and repeated positive selected analysis again, but the results was same as before. We have checked some references and found similar problem (Liu et al., 2021; Shao et al., 2022).
In the one hand, positive selection is inferred if the ratio of nonsynonymous substitutions per nonsynonymous site to synonymous substitutions per synonymous site (dN/dS) is statistically significantly greater than one in a test of the neutral null hypothesis dN/dS = 1. Positively selected analysis is affected by multiple factors, such as the species in the background branches, the completeness of genome assembly, and the results of genomic annotation. In our study, the background branches (Homo sapiens, Anneissia japonica, Acanthaster planci, Asterias rubens, Plazaster borealis, Ophiothrix spiculata, Strongylocentrotus purpuratus, Lytechinus variegatus, and Apostichopus japonicus) are based on metazoan, while the background branches (A. japonicus, P. parvimensis, S. Purpuratus, L. variegatus, A. planci, and A. japonica) are based on Echinozoa. The different ranges of background branches and the some different species lead to the difference of positive selected analysis in both studies by the dN/dS accumulation. The similar issue have found in positive selected analysis for Paelopatides sp. (Liu et al., 2021; Shao et al., 2022).
In the other hand, we found the regulated genes of DNA damage repair in both cold seep C. heheva (MSH4) and hydrothermal vent C. heheva (POLB, FAN1, RFC2, KDM2A, FARSA, SPG7, BRCA1, TTLL9, DCLK1, LDHD, and SIRT4). The DNA damage repair adaptation were found in both studies, that suggested the similar adaptation to deep-sea environment.

The references in here.
Liu, RY, Liu, J, Zhang, HB. Positive selection analysis reveals the deep-sea adaptation of a hadal sea cucumber (Paelopatides sp.) to the Mariana Trench. J Oceanol Limnol 2021; 39(1), 266-281.
Shao G, He T, Mu Y, et al. The genome of a hadal sea cucumber reveals novel adaptive strategies to deep-sea environments. Iscience 2022; 105545.

2. To detect signatures of positive selection associated with hydrothermal vent adaptation, the protein set from the published C. heheva H assembly could have been included in the tests for selection alongside the other 9 species. This would help rule out cold seep vs hydrothermal adaptation, if such signatures exist.

Thank you for your valuable suggestion. Considering the suggestion, we have repeated the positive selection analysis that the protein set of Haima cold seep C. heheva has added to. We found that the positive selection genes are same as before. According to the positive selection analysis is based on orthologous genes, we need to identify the candidate orthologous genes from protein sets of the two C. heheva and other 9 species in first. In theory, the protein sets of same species often has a high similarity and may provide the same orthologs for positive selection analysis. In order to confirm this view, we searched the orthogroups of the protein sets between hydrothermal vent C. heheva and cold seep C. heheva by OrthoFinder. The overlapped orthogroups (12608) between the two C. heheva cover 100 % of the orthogroups in hydrothermal vent C. heheva (12608) and 95.70 % of the orthogroups in cold seep C. heheva (13175). The candidate orthologs of the two C. heheva for positive selection analysis is provided from the overlapped orthogroups (12608), which result to as the same positive selection genes after the added protein set of cold seep C. heheva added. In addition, we found the candidate orthologs of hydrothermal vent C. heheva completely overlap whether the cold deep C. heheva was added or not. Therefore, the gene changes of cold seep vs hydrothermal vent adaptation is not including in those orthologs, and can not affected results of positive selection analysis in this study.

Powered by Editorial Manager® and ProduXion Manager® from Aries Systems Corporation

	3. I have a similar concern with the gene family expansion analyses. Again, the overlap between the results in this study and Zhang are not discussed, yet this would be a straight forward way to validate the results. Given how distantly related C heheva is to the other species in the comparison, there would almost certainly be a number of shared expanded families in the C. heheva K (this study) and C. heheva H (Zhang) genomes, along with some population-specific adaptations.<br><br>Thank you for the valuable suggestion. According to gene-family analysis, we performed gene-family analysis based on the phylogenetic tree of 10 metazoan species, while Zhang et al. (2022) performed that of 7 echinoderms. In theory, the different species included in gene-family analysis is the main that inevitably affect the results. In addition, we have searched our expanded gene families, which significantly enriched in the processes of membrane fluidity maintaining, iron homeostasis regulation, and DNA damage repair, then compared with the expanded families in C. heheva H (Zhang et al., 2022), the shared expanded gene families was found in DNA damage repair as HTH psq-type domain and HTH CENPB-type domain of PTHR19303 (in our study) vs transposon of PTHR19303 (in Zhang's study) . As we known, the DNA damage repair adaptation is broadly-speaking in various fauna in the deep sea.<br><br>4. The BUSCO completeness score of the entire genome is 92.4%, yet the BUSCO completeness score of the annotations (protein mode I assume) is reported at 95%. Given this difference is not modest, it is unclear to me how one can find more single-copy orthologs in the annotations of a genome rather than the genome itself. In theory, everything in a protein set derived from the genome would be in the genome itself. Typically it is the other way around - more BUSCOs identified across the entire genome, and a subset of these are annotated. To me this suggests a discrepancy is how the BUSCOs were searched (was Augustus optimized in genome mode?) or a versioning issue. This difference should be addressed.<br><br>Thank you for pointing out this issue. First of all, we have to update the BUSCO's completeness from 92.4 % (BUSCO version 4.1.4) to 94.5 % (BUSCO version 5.4.6) according to editor's requirement. The BUSCO results have uploaded to GigaDB as required. For the BUSCO's results, the completeness of the assembly was evaluated using BUSCO (version 5.4.6), and then the completeness of protein set after annotated was also evaluated using BUSCO (version 5.4.6), then the results were provided files that list all sequences and their matched status in BUSCO dataset. In order to obtain high complete protein set for next analyses, we compared the two files and found the intersection. Based on the intersection of protein set, we added the sequences, which missed in the BUSCO evaluated by genome or protein set, to the final protein set for followed analyses. Finally, the final protein set was evaluated using BUSCO (version 5.4.6) again. Thus, the completeness of annotated protein set may slightly higher than completeness of genome.<br>Here, we list the BUSCO results in short summary of genome and the final protein set as following.<br>Genome<br>BUSCO version is: 5.4.6<br>C:94.5%[S:93.5%,D:1.0%],F:2.8%,M:2.7%,n:954<br>902 Complete BUSCOs (C)<br>892 Complete and single-copy BUSCOs (S)<br>10 Complete and duplicated BUSCOs (D)<br>27 Fragmented BUSCOs (F)<br>25 Missing BUSCOs (M)<br>954 Total BUSCO groups searched<br>The final protein set<br>BUSCO version is: 5.4.6<br>C:95.0%[S:94.2%,D:0.8%],F:1.5%,M:3.5%,n:954<br>907 Complete BUSCOs (C)<br>899 Complete and single-copy BUSCOs (S)<br>8 Complete and duplicated BUSCOs (D)<br>14 Fragmented BUSCOs (F)<br>33 Missing BUSCOs (M)<br>954 Total BUSCO groups searched<br><br>5. The methodology underlying the "Unique Gene" set of analyses should be better

Powered by Editorial Manager® and ProduXion Manager® from Aries Systems Corporation

explained in the results and the methods. Does this mean genes found in C. heheva K but not in C. heheva H? The phrase "unique genes in KEGG annotation and 330 unique genes in GO annotation" on lines 317-318 is unclear. Does this mean KEGG and GO enrichments analyses were run on the sets of unique genes? If so, what was the background set of genes for these enrichment analyses.

Thank you for pointing out this issue and the suggestion. In the former manuscript, we run the unique genes analysis based on KEGG annotation and GO annotation among three species of the Kairei vent C. heheva, the Haima cold seep C. heheva, and shallow water A. japonicus. Considering your comment in the next (6), we have run the unique genes analysis by OrthoVenn3 based on OrthoFinder algorithm, the section of unique genes in method part and result have been re-written in the manuscript.

6. In the unique genes list, ferritin heavy chain 1 (FTH1) is highlighted. If my understanding of "uniqueness" is correct, this means FTH1 is not found in the published C. heheva H assembly, but is found in the C. heheva K assembly reported here. It is proposed that this gene may play an outsized role in iron processing in C. heheva K. However, a quick blast search of FTH1 identifies this a homolog of this gene in many metazoans (several echinoderms, mammals, among others). So, I am not sure this gene is unique to C. heheva K, but may instead be lost in C. heheva H (or not annotated for technical reasons). Including other echinoderms in the "unique gene" analyses would perhaps provide a better way of accessing species-specific gains in genetic content. I think the Orthofinder results would also provide this information. Overall, the methods and interpretation of the unique genes results need revising.

We gratefully appreciate for your careful check and your valuable suggestion. It is really true as your comment that ferritin, consists of heavy-chain subunits (FTH1) and light-chain subunits (FTL), is a homolog in many metazoans. Indeed, the loss of FTH1 in other two species may result from annotation methods and genome completeness. In former manuscript, the protein sets of the three species (C. heheva K, C. heheva H, and A. japonicus) was annotated to database of KEGG and GO followed the same protocol. Then, the relative unique genes was performed by Venn analysis and based on results of KEGG annotation and GO annotation. The gene FTH1 was found in the subset of C. heheva K, that means FTH1 do not shared to the other two species and become the relative unique gene of C. heheva K. FTH1 has an function on iron-rich detoxifcation and iron homeostasis, which is great conform to high content of iron in Kairei vent.
Considering the suggestion of OrthoFinder that provide species-specific genes, we have performed orthologous clusters analysis by OrthoVenn3 based on OrthoFinder algorithm among the protein sets of 6 echinoderms (A. japonica, A. planci, O. spiculata, S. purpuratus, and C. heheva of the Kairei vent and Haima cold seep) .
The section of unique genes in method part and result have been re-written in the manuscript.

Minor:

1. Line 20: "scaffold N50"
2. Line 20-21: BUSCO completeness score
3. Line 22: Instead of "natural chromosome" another way of phrasing could be "19 large scaffolds predicted to represent each of the species' chromosomes"
4. Line 23: "Species-specific" genes instead of "unique" genes? Unique is ambiguous here
5. Line 136-137: After purging dups, the genome is still contig-level. Scaffolds are generated from HiC data later
6. Line 141: Juicebox*
7. Were any scaffolds manually repaired in JuiceBox. If so, this should be described
8. Line 190: JCVI should be expanded here for the first mention
9. Line 203: "tests for positive selection" rather than "positively selected analysis"
10. Line 211: "evolving under positive selection" rather than "positively selected"
11. Line 233: How does 92.4% compare to other quality sea cucumber or echinoderm genomes? Is this high?

We gratefully appreciate for pointing out these issues and your valuable suggestion in above. We have made the corrections according to the above comments in the

Powered by Editorial Manager® and ProduXion Manager® from Aries Systems Corporation

manuscript. For the genome completeness, we have to update the value from 92.4 % (BUSCO version 4.1.4) to 94.5 % (BUSCO version 5.4.6) according to editor's requirement of using the latest BUSCO software (Version 5.2+). The 94.5 % (BUSCO version 5.4.6) completeness of the Kairei vent C. heheva is higher than that 89.6 % (BUSCO version 4.0.5) completeness of the Haima cold seep C. heheva.

Reviewer #3: This is a high-quality genome assembly of Chiridota heheva, an apodid sea cucumber. It is the first chromosome-level genome assembly of an apodid sea cucumber. The specimen comes from a very inaccessible part of the ocean and this fact alone makes this resource incredibly valuable. The genomic work is high quality. This resource will be invaluable to a wide range of researchers including those studying other sea cucumbers or echinoderms more widely, those studying deep-sea organisms, those studying organisms associated with chemosynthetic environments. The following are a few general thoughts about the paper:

1. All command lines run as part of these analyses should be provided. Providing command lines removes any ambiguity as to how the analyses were carried out. See the docx file in the supplementary materials of Zhou et al. 2017 (https://doi.org/10.1093/molbev/msx302) for an excellent example of how to include command lines.

Thank you for the suggestion. We have checked the docx file in the supplementary materials of Zhou et al. 2017 (https://doi.org/10.1093/molbev/msx302), and have provided the command of analyses in the a word file as supplementary, please check the supplementary files.

2. The manuscript would be greatly improved if it included a resources table, which includes key reagents, software versions, etc. A good example of one is in the following publication just after the References section: https://www.cell.com/cell/pdf/S0092-8674(23)00107-1.pdf (this would not be a replacement for methods section)

Thank you for the suggestion. Considering to the suggestion, we have read the resources table of Shao et al. 2023 (https://www.cell.com/cell/pdf/S0092-8674(23)00107-1.pdf), the key reagent and resource of our study have added in a supplementary table, please the supplementary files.

3. The current version of the manuscript does not directly address the major differences between the cold-seep C. heheva and and vent C. heheva. The assemblies are >300 megabases different in size. There are >300 genes present in vent C. heheva that are absent from the cold-seep C. heheva assembly (LINE 317). Is this an issue with the assembly or are these separate species? Is there other evidence that can be brought to address this issue (e.g., nucleotide variability)?

Thank you for pointing out this issue.
Indeed, the differences between the cold-seep C. heheva and and vent C. heheva should be considered in the further study. In fact, we have collected some chiridotid holothurians that distributed in many ecosystems, the differences between the chiridotids is the essential parts for environment adaptation on chiridotids. However, compared to the C. heheva genome of Zhang et al. (2022) reported, our C. heheva genome was collected from hydrothermal vent while Zhang's genome was colected from cold seep, our assembly level is chromosome while Zhang's is contig, our genome completeness is high as 94.50 % (BUSCO) while Zhang's completeness is 89.60 % (BUSCO). It is worth to report the chromosome-level genome assembly of hydrothermal vent C. heheva, and provide the high quality of chromosome-level genome in hydrothermal vent for studinng the adaptation in deep-sea extreme environment.
According to the assembly results and genome annotation, the difference of genome sizes is 323 Mb between the Kairei vent C. heheva genome (1.43 Gb) and the Haima cold seep C. heheva genome (1.107 Gb), the difference sizes of repetitive elements is 338 Mb between the of the Kairei vent C. heheva (70.80 %, 1.012 Gb) and of . Based on the repetitive elements, the Kairei vent C. heheva (56.40 %, 624 Mb). That suggested the bigger size of the Kairei vent C. heheva than the Haima cold seep C. heheva may result from the high percentage of the repetitive elements. Moreover, the

	different completeness of genome assembly also lead to the difference sizes (15 Mb, 338 Mb - 323 Mb) both genomes.<br><br>The following are some specific points:<br><br>1. LINE 271: "Compared with the other 9 metazoans, 450 gene families were expanded, and 6 were contracted in C. heheva (Fig. 3A)."<br>-- This is vague as written. The CAFE analyses provide information relative to ancestors and this should be communicated. For example, the 450 gene family expansions and 6 contractions occurred since the last common ancestor of A. japonicus and C. heheva.<br><br>Thank you for pointing out this issue. According to the suggestion, we have re-written the part in the revised manuscript (lines 251-253).<br><br>2. LINE 306: "involved in DNA damage"<br>-- More accurate to say the genes are involved in "DNA repair" or "response to DNA damage."<br><br>Thank you for your suggestion. According to the suggestion, we have re-written this part in the revised manuscript (line 311).<br><br>3. Figure 3 legend should mention that orthologous results are based on CAFE analysis.<br><br>Thank you so much for your careful check. According to the suggestion, we have added the orthologous results to Figure 3 legend in the revised manuscript.
**Additional Information:**	
**Question**	**Response**
Are you submitting this manuscript to a special series or article collection?	No
**Experimental design and statistics**<br><br>Full details of the experimental design and statistical methods used should be given in the Methods section, as detailed in our Minimum Standards Reporting Checklist. Information essential to interpreting the data presented should be made available in the figure legends.<br><br>Have you included all the information requested in your manuscript?	Yes
**Resources**<br><br>A description of all resources used, including antibodies, cell lines, animals and software tools, with enough information to allow them to be uniquely	Yes

identified, should be included in the Methods section. Authors are strongly encouraged to cite Research Resource Identifiers (RRIDs) for antibodies, model organisms and tools, where possible.<br><br>Have you included the information requested as detailed in our Minimum Standards Reporting Checklist?	
**Availability of data and materials**<br><br>All datasets and code on which the conclusions of the paper rely must be either included in your submission or deposited in publicly available repositories (where available and ethically appropriate), referencing such data using a unique identifier in the references and in the "Availability of Data and Materials" section of your manuscript.<br><br>Have you have met the above requirement as detailed in our Minimum Standards Reporting Checklist?	Yes

Manuscript_revised Click here to access/download;Manuscript;Manuscript_revised.docx

A high-quality chromosomal genome assembly of the sea cucumber *Chiridota heheva* and its hydrothermal adaptation

Yujin Pu, Yang Zhou, Jun Liu, Haibin Zhang*

[1] Institute of Deep-sea Science and Engineering, Chinese Academy of Sciences, Sanya 572000, China

[2] University of Chinese Academy of Sciences, Beijing 100049, China

*Correspondding address. Haibin Zhang, Institute of Deep-sea Science and Engineering, Chinese Academy of Sciences, Sanya 572000, China. E-mail: hzhang@idsse.ac.cn

Abstract

Background: *Chiridota heheva* is one of the cosmopolitan holothurian, which has been found well adapted to diverse deep-sea ecosystems, especially chemosynthetic environments. Besides high hydrostatic pressure and limited light, high concentrations of metal ions also represent harsh conditions in hydrothermal environment. Few holothurian species can live in such extreme condition. Therefore, it is valuable to elucidate the adaptive genetic mechanisms of *C. heheva* to hydrothermal environment.

Findings: Herein we report a high-quality reference genome assembly of *C. heheva* from the Kairei vent, which is the first chromosome-level genome of Apodida. The chromosome-level genome size was 1.43 Gb, with a scaffold N50 of 53.24 Mb and BUSCO completeness score of 94.5 %. Contig sequences were clustered, ordered, and assembled into 19 natural chromosome. Comparative genome analysis found that the

expanded gene families and positively selected genes of *C. heheva* were involved in DNA damage repair process, and the expanded genes and the unique genes were contributed to maintaining the iron homeostasis in iron-enriched environment. Positively selected gene *RFC2* with 10 positively selected sites was played an essential role in DNA repair under the extreme environment.

Conclusions: This first chromosome-level genome assembly of *C. heheva* reveals the hydrothermal adaptation of holothurians. As the first chromosome-level genome of Order Apodida, this genome will provide the resource for investigating the evolution of Class Holothuroidea.

Keywords: *Chiridota heheva*, Hi-C, positively selected gene, gene family, unique gene

Date Description

Context

Hydrothermal vents are one of the typical deep-sea chemosynthetically-driven ecosystems that inhabit a wide array of animals and chemosynthetic microbes. The hydrothermal vent environment is characterized by rapid changes in temperature, acidic pH, sulfur compounds, metal, methane, hydrogen, carbon dioxide, and other toxic chemistry, besides high hydrostatic pressure and darkness of the deep sea [1-9]. However, these inhospitable environments had reported as key areas of enrichment for deep-sea life.

The hydrothermal habitat fauna are commonly adapt to the unusual environment

with the uncommon physical and chemical properties of vent fluids. Diverse fauna, including Annelida, Arthropoda, Mollusca, Echinodermata, Cnidaria, and Chordata have been described in hydrothermal vents, these vent faunas survive on their unique strategies in the extreme conditions [9]. According to the previous studies of common vent fauna, such as crab *Austinograea rodriguezensis* [10], shrimps *Rimicaris kairei* [11], *Rimicaris* sp. [12], mussel *Gigantidas vrijenhoeki* [13], *Bathymodiolus* mussels [14], and scaly-food gastropods *Chrysomallon squamiferum* [11, 15-18], were evolved to enhance their tolerance of high temperature, metal ion enrichment, and sulfur rich conditions. The adaptive mechanism is like a hard exoskeleton to endure the thermal stress [10], the ion binding enzymes or respiratory proteins for ion homeostasis and detoxification [11, 14-17]. Besides the unusual conditions of hydrothermal vents, the adaptations for high hydrostatic pressure and limited light are inevitable. Among various deep-sea fauna, DNA repair, degenerated ossicles, protein activity protection, and cell cycle maintenance have evolved to high hydrostatic pressure adaptation[5, 19-21]. The white body colour, unpigmented skin, scales, and long-wavelength light sensors of marine fauna were ubiquitous in the light-limited deep sea [6, 19-20, 22-23]. To gather knowledge about the genetic basis of adaptation to deep-sea extreme environments are of particular interest.

Holothurians are widely distributed in several ecosystems' ocean, and more than 1,800 specie have been accepted at present [24]. Few holothurian species can live in such extreme condition of hydrothermal environments. *Chiridota heheva*, with the features of inhabiting in all biotopes of the deep-sea ocean [9, 21, 25-27], is a

representative case to explore the molecular basis of an adaptive trait in deep-sea extreme conditions. The cold seep adaptations of *C. heheva* have been reported by Zhang et al. [21]. However, genome information on *C. heheva* in hydrothermal vent is currently unavailable. In the present study, we sequenced the genome of *C. heheva* with sample collected in the Kairei vent. Kairei vent is an ultramafic-hosted system was discovered in the Indian Ocean. Kairei fluids are highly enriched in dissolved Fe (5,400 μM) that leach from the host rock [9, 15, 28-29]. We obtained a chromosome-level genome of *C. heheva* by Hi-C technology with an integrated comprehensive gene set. Moreover, comparative genomic analyses were performed to investigate the hydrothermal vent adaptive mechanisms of *C. heheva*. Finally, together with other published genomic data from vent animals, this assembly results can add more information which will helpful to gain insights into the adaptation of the whole vent fauna.

Methods

Sampling and sequencing

The *C. heheva* individual used for genomic sequencing was collected by the maned submersible vehicle '*Shenhaiyongshi*' from the Kairei vent filed in the Mid-Indian Ocean (70.40°E, 25.32°S), with a depth of 2,428 m, on 7 February 2019 (Fig. 1). The sample was dissected and frozen in liquid nitrogen, then send to the Institute of Deep-sea Science and Engineering, Chinese Academy of Science, Sanya, China, and subsequent storage at -80℃ for further analysis.

The high-molecular-weight genomic DNA (gDNA) was prepared manually from body-wall tissue following a modified protocol described previously [30]. Briefly, tissue was ground with liquid nitrogen freezing and digested at 65℃ in SDS (sodium dodecyl sulfate) buffer [50 mM Tris-HCl, 50 mM EDTA, 3% SDS (w/v)] for 1 h. Then the lysate was treated by Phenol/Chloroform isolated and Isopropanol precipitation. The gDNA was assessed and sheared to ~15 kb fragment length for Pacific Biosciences (PacBio) HiFi sequencing. The HiFi SMRTbell library was constructed with SMRTbell Express Template Prep Kit 2.0 (Pacific Biosciences, California, USA), and the HiFi reads were sequenced using 1 cell on SMRT cells 8M on a PacBio Sequel II platform (PacBio Sequel II System, RRID:SCR_017990). For genome annotation, the total RNA was isolated from gonad and body-wall tissues using a RNeasy Plus Universal Kit (QIAGEN, Hilden, Germany). The total RNA was used to obtain cDNA by reverse transcribing, and then 150 bp paired-end reads were generated on Illumina NovaSeq 6000 platform (Illumina NovaSeq 6000 Sequencing System, RRID:SCR_016387). The sequencing processes above were conducted by Novogene Company, Tianjin, China.

Hi-C library preparation and sequencing from body-wall tissue have been done following the standard protocol described previously [31]. Briefly, crosslinking the grounded body-wall tissue with 4% formaldehyde, digesting the DNA with restriction enzyme MboI (GATC), making the DNA ends with biotin-14-dCTP, ligating the blunt-end fragments, shearing the DNA into 200- to 600 bp fragments by sonication. Finally, the Hi-C sequencing library was constructed and conducted on the Illumina

NovaSeq-6000 sequencing platform (PE 150bp). The experiments and sequencing were performed by Novogene Company, Tianjin, China.

Genome assembly and annotation

Hifiasm version 0.16.1-r375 (Hifiasm, RRID:SCR_021069) with default parameters setting was used for PacBio HiFi reads assembly [32]. Purge_dups version 1.2.5 (purge dups, RRID:SCR_021173) was used for redundancy purge of the primary genome and obtained the clean genome without the duplicate contigs [33]. Juicer version 1.6 (Juicer, RRID:SCR_017226) was used to analyze Hi-C reads combined with contig-level genome [34]. 3D-DNA version 190716 (3D de novo assembly, RRID:SCR_017227) was used to primarily correct misjoin, order and orient in the scaffold and obtained the potential chromosomal groups [35]. Juicebox version 1.11.08 was then used to manually order the scaffolds of the result from 3D-DNA [36]. The tool 3D-DNA was used again to obtain the final chromosome assembly for further analysis [35]. The completeness of the chromosome-level genome was assessed using BUSCO version 5.1.2 (BUSCO, RRID:SCR_015008) with the metazoa_odb10 lineage data set (954 orthologs) [37].

RepeatModeler version 2.0.1 (RepeatModeler, RRID:SCR_015027) [38] and RepeatMasker version open-4.0.6 (RepeatMasker, RRID:SCR_012954) [39] were used for searching repetitive elements in the final genome assembly and generated a soft-masked genome with non-redundant data set of repetitive elements. Subsequently, gene structure annotation in the soft-masked genome was predicted by *ab initio* and

evidence-based gene prediction as follow. Augustus version 3.4.3 (Augustus, RRID:SCR_008417) [40], GlimmerHMM version 3.0.4 (GlimmerHMM, RRID:SCR 002654) [41], and GeneID version 1.4.5 (Entrez Gene, RRID:SCR_002473) [42] were used in *ab initio* gene prediction. Moreover, Exonerate version 2.2.0 (Exonerate, RRID: SCR_016088) was employed for protein homologous annotation in evidence-based gene prediction [43]. PASA version 2.5.2 (PASA, RRID:SCR_014656) was applied for transcriptomic annotation in evidence-based gene prediction [44]. EVidenceModeler version 1.1.1 (EVidenceModeler, RRID:SCR_014659) produced a weighed consensus protein set by combining the results from *ab initio* gene models and evidence-based gene models [45]. The protein set was used for gene functional annotation as follows. DIAMOND BLASTP version 2.0.14 was used to search protein function in the nr database of NCBI [46], Interproscan version 5 (InterProScan, RRID:SCR_005829) was employed to predict the protein family membership, functional domains and sites in Swiss-Prot, Pfam [47], and KAAS (KEGG Automatic Annotation Server) was applied for KEGG pathways annotated online (https://www.genome.jp/tools/kaas/).

Orthology prediction and phylogenomic analysis

Protein sets of night echinoderm species (*Anneissia japonica*, *Acanthaster planci*, *Asterias rubens*, *Plazaster borealis*, *Ophiothrix spiculata*, *Strongylocentrotus purpuratus*, *Lytechinus variegatus*, *Apostichopus japonicus*, and *C. heheva*) were employed in the orthology identification with *Homo sapiens* as the outgroup

(Supplementary Table S1). OrthoFinder version 2.5.4 (OrthoFinder, RRID:SCR_017118) was applied to determine and cluster gene families among these 10 metazoan species [48]. A total of 495 single-copy orthologs among these species were multiple aligned with MAFFT version 7.475 (MAFFT, RRID:SCR_011811) [49], then concatenated and used for constructing a phylogenomic tree using RAxML version 8.2.3 (RAxML, RRID:SCR_006086) [50] based on the substitution model of GTRGAMMA with 100 bootstraps. The divergence time among these species was estimated using MCMCTREE in PAML version 4.9 (PAML, RRID:SCR_014932) [51]. The calibration times were derived from the TimeTree database (http://www.timetree.org/).

Genome synteny analysis

Chromosome-level genome in our study of *C. heheva* (CHEH_vent1.0) and *A. japonicus* (AJH1.0) [52] was selected as comparisons for syntenic analysis. BLAST version 2.9.0 (BLAST Similarity Search, RRID:SCR_008419) with parameter "-evalue 1e-10" was used to identify similar gene pairs [53]. JCVI version 0.18 (RRID:SCR_021641) was used to perform protein sequence alignment between CHEH_vent1.0 and AJH1.0 and filter the BLAST results with parameter "--cscore =0.5", then search for syntenic blocks in all the genes (jcvi, RRID:SCR_021641) [54]. Subsequently, JCVI was also used to visualize the syntenic results with the graphic command.

Gene family analysis

Based on orthologous gene families and phylogenetic relationships above, CAFE version 4.2.1 (CAFE, RRID: SCR_005983) [55] was used to detect the gene family expansion and contraction. GO enrichment and KEGG pathway enrichment were performed online (https://www.genescloud.cn/) and were used to investigate the functional properties of the expansion gene families. A conditional P value was calculated for each gene family, and a significantly accelerated rate of expansion families was left while P values were lower than 0.05.

Genes under positive selected

As the number of single-copy orthologous genes from OrthoFinder is limited, orthologs were identified as reciprocal best blast hits using the RBH Ortholog pipeline [56]. A total of 3,269 orthologs identified above were used for tests for positive selection. MAFFT version 7.475 (MAFFT, RRID:SCR_011811) [49] was used for multiple aligned, and the alignments of the corresponding DNA codon sequences further trimmed by trimAl version 1.4.1 (trimAl, RRID:SCR_017334) [57]. Positively selected genes and amino acid sites were assessed with the branch model and branch-site model using codeml in PAML package version 4.9 (PAML, RRID:SCR_014932) [51]. A likelihood ratio test was conducted, and the false discovery rate (FDR) correction was performed for multiple comparisons. Genes and sites with a corrected FDR <0.05 were defined as evolving under positive selection.

Unique genes

Protein sets of six echinoderms (*A. japonica*, *A. planci*, *O. spiculata*, *S. purpuratus*, the Haima cold seep *C. heheva*, and the Kairei vent *C. heheva*) were employed for orthologous clusters analysis. OrthoVenn3 [58], which is freely accessible at https://orthovenn3.bioinfotoolkits.net, was used to identify clusters basaed on OrthoFinder algorithm among the six echinoderms. The cluster results were visualized by UpSet (RRID:SCR_022731) [59], which could support the overlapped clusters among diverse species and provide the unique clusters among each species. Based on the unique clusters of the Kairei vent *C. heheva*, the unique genes were collected from in the unique clusters and were used to GO enrichment. The UpSet and GO enrichment analyses were automatically run on OrthoVenn3 platform.

Results

Chromosome-scale genome assembly and completeness evaluation

The CCS HiFi reads with 29.25 Gb were sequenced on the PacBio Sequel II platform (Supplementary Table S2). In order to create continuity in the genome assembly, 159.59 Gb of Hi-C reads were further prepared on Illumina NovaSeq 6000 sequencing platform (~ 111 × genome coverage) (Supplementary Table S2). RNA reads with 13.29 Gb were generated on Illumina NovaSeq 6000 sequencing platform utilized for genome annotation (Supplementary Table S2).

Our chromosome-level genome assembly of *C. heheva* (CHEH_vent1.0) was

performed using both HiFi reads and Hi-C reads. The total size of the final assembly was 1.43 Gb with an N50 of 53.24 Mb, consisting of 19 chromosome-level scaffolds with lengths ranging from 30 to 115 Mb (Fig. 2, Table 1). The genome size of this species in the Haima cold seep is 1.107 Gb [21], only three-quarters of the genome size in Kairei vent. BUSCO [37] with a database of metazoan_odb10 was then used to evaluate the integrity of the genome assembly, the results as 94.5% of the conserved genes indicated the high integrity of our assembled genome in CHEH_vent1.0 (Table 1).

Annotation of repetitive elements and protein-coding genes

Repetitive element annotation identified that in 70.80% (1.02 Gb) of the whole genome assembly, the long interspersed nuclear elements (LINEs) were the largest class of the transposable elements (TEs) annotated, other predominant repetitive elements are summarized in Table 2. Compared with other echinoderms (Supplemental Table S3), the repetitive genes percentage of *C. heheva* (Kairei vent) in this study is more than that 56.64% in Haima Cold Seep [21], and only less than *Paelopatides sp.* Yap 73.93% [23], and the percentage of shallow water *A. japonicus* only have 27.20% [52, 60]. After repeat masking, protein-coding genes annotated were using a combination of *ab initio*, homology-based, and transcript-evidence predicted approaches, and a total of 32,434 were successfully identified (Table 3). Interproscan, KEGG, NR, and UniProt were employed for functional annotations, and 24,606 genes were mapped to at least one database (Table 3).

Phylogenetic and syntenic relationship

In order to investigate the phylogenetic relationship between *C. heheva* and other metazoans, 9 species were selected for the phylogenomic tree reconstruction (Supplementary Table S1). A total of 495 single-copy genes in all species with high completeness genomes were used to construct a phylogenomic tree (Fig. 3A, B; Supplemental Fig. S1). *C. heheva* and *A. japonicus* appeared as a sister clade in holothurians, and diverged from other echinoderms approximately 438.1 Mya. The divergence time of holothurians in this study supported the view that holothurians had evolved by the Ordovician [61-65].

The syntenic blocks were detected between *C. heheva* and *A. japonicus* using JCVI [54] and shown as a dot plotter (Fig. 3C). The results indicated *C. heheva* has a relatively conserved relationship with *A. japonicus*, except chromosomes 1, 3, 4, and 5 of *C. heheva*. We identified chromosome fission or fusion events between the 19 chromosomes of *C. heheva* and 23 chromosomes of *A. japonicus*. Chromosome 1 of *C. heheva* corresponded to chromosomes 4 and 12 of *A. japonicus*, while chromosome 3 corresponded to chromosomes 10 and 21, chromosome 4 corresponded to chromosomes 7 and 17, and chromosome 5 corresponded to chromosomes 3 and 23, respectively.

Gene family evolution

Based on the phylogenomic tree (Fig. 3A), gene family analysis was performed using CAFE [55]. Compared with the other 9 metazoans, the 450 gene families

expansions and 6 contractions occurred since the last common ancestor of *A. japonicus* and *C. heheva* (Fig. 3A). Collectively, these expanded gene families of *C. heheva* were mainly enriched in membrane functions, nucleoside processes of DNA repair, and proteins activity (Fig. 4; Supplementary Table S4). Membrane-associated processes have been described that were particularly susceptible to perturbation under conditions of high hydrostatic pressure, including reducing the fluidity of lipid bilayers and denaturing membrane-associated proteins [19, 65-66]. Biological membranes are mainly composed of phospholipids, sterols (generally cholesterol), glycolipids, and proteins [66-67]. Phospholipids, with hydrophilic the phosphate group head and hydrophobic the fatty acid tails, are a major component that form the lipid bilayers [67]. Membrane fluidity is modulated by changes in lipid composition, especially the proportion of unsaturated fatty acids [19, 23, 66, 67-68]. As our results, lipid metabolisms were activated to response to high hydrostatic pressure, including essential fatty acid of arachidonic acid (AA) metabolism, linoleic acid (LA) metabolism, alpha-linolenic acid (ALA) metabolism, and so on (Fig. 4; Supplementary Table S4). AA, LA, and ALA are polyunsaturated fatty acids (PUFAs), which is metabolized into various PUFAs during stress (Supplementary Figure S2). Phospholipid-bound AA is the substrate for the synthesis of a range of biologically active compounds, including prostaglandins (PGs), thromboxanes (TXs), and leukotrienes (LTs), epoxyeicosatrienoic acids (EETs), and hydroxyeicosatetraenoic acids (HETEs) [69]. LA is importance in the biosynthesis of AA, while ALA is the precursor of EPA and DHA and then converts into EPA and DHA through metabolism

[68]. Some of the PUFAs may bound to receptor on the membrane of cells and related to membrane fluidity adaptation to compensate for environmental changes, especially AA and DHA have been reported in previous studies [19, 23, 68-69]. Iron plays a crucial role in living organisms, and is intimately involvement in numerous biological processes [Ravingerová et al., 2020, Song et al., 2022]. Iron in organisms are mainly bound to heme (heme-iron), transported by transferrin (TF-bound iron), and stored in ferritin (FT-stored iron) for biological functions, which are redox-inert iron in nontoxic forms [Ravingerová et al., 2020]. Nontransferrin-bound iron (NTBI) and other free iron released from iron-bound proteins are potentially toxic form, due to excess redox-active iron induces oxidative stress and causes cell damage [70-71]. Deep-sea fauna, explored in iron-rich hydrothermal vents, have several mechanisms to maintaining the iron homeostasis [9, 14, 16-17]. Cytochrome P450 (CYP) was reported as an important role in the regulation of iron levels to maintain cellular redox homeostasis and against oxidative stress [71]. P450 and transferrin are the expanded gene families, which enriched in iron ion binding and metal ion transport respectively, suggesting the adapting to the iron-rich environment of the Kairei vent. High hydrostatic pressure may damage DNA in deep-sea fauna susceptibly [20]. The responding of DNA repair was variously described in previous studies, including DNA damage detection, replication, recombination, splicing, excision, endonuclease, and so on [5, 20, 23, 72]. The expanded gene families enriched in DNA replication, DNA recombination, DNA-associated protein, and nucleic acid binding, may suggestion a capability of DNA repair. High hydrostatic pressure inhibits protein

functions by affecting folding and enzyme activity [19, 21]. These expanded gene families clustered in protein synthesis and activities of various enzymes were contributed to ensuring the functions of the protein.

Positively selected genes

The positively selected genes support the genetic basis for environmental adaptation. Compared with the other 9 metazoans, 28 positively selected genes were identified in *C. heheva* (Table 4). According to GO enrichment and KEGG enrichment functional analysis, positively selected genes were significantly enriched for various processes including cyclic compound binding, ion binding, nucleotide binding, ATP binding, and DNA binding and damage repair (Supplementary Table S5). Some genes of deep-sea fauna were positively selected during the adaption to environment. DNA repair genes had been selected for deep-sea adaptation, and may play an important role in maintaining the fidelity of genetic materials in deep-sea environments [5, 20, 23, 72]. Among these processes, at least 11 positively selected genes (*POLB*, *FAN1*, *RFC2*, *KDM2A*, *FARSA*, *SPG7*, *BRCA1*, *TTLL9*, *DCLK1*, *LDHD*, and *SIRT4*) involved in DNA repair (Table 4; Supplementary Table S5). Therein DNA repair gene *BRCA1* had been found to protect DNA from high pressure in hadal *Paelopatides* sp. Yap [23]. Furthermore, gene *RFC2,* which functions as DNA replication, nucleotide excision repair, mismatch repair, DNA repair and recombination proteins, has 10 positively selected sites (Fig. 5A, B). These 10 positively selected sites may enhance the DNA repair abilities of *RFC2* and reveal potential pathways for enhancing the

high hydrostatic pressure tolerance.

Unique genes evolution

In order to investigate the environment-specific adaptation of the Kairei vent *C. heheva*, we identified the unique clusters based on the protein sets of six echinoderms. As the results of UpSet shows, a total of 14,032 clusters were identified in the Kairei vent *C. heheva*, and 5,132 clusters were shared with the other five echinoderms, while 933 clusters were identified only in the Kairei vent *C. heheva* and were considered as unique clusters (Fig. 6). As the results of GO enrichment analysis, the unique genes of the Kairei vent *C. heheva* were significantly enriched in category of iron binding and transport, including sodium ion transport, ion transport, sodium-independent organic anion transport, metal ion binding, and zinc ion binding (Fig. 7). Among the ion-relative terms, metal ion binding (GO:0046872) exerts an iron ion binding function through CYP2B14P (Cytochrome P450, family 2, subfamily b, polypeptide 14), and CYP 450 has reported that acted as a function of iron homeostasis maintaining [71]. The metal ion binding enriched term of the unique genes of the Kairei vent *C. heheva* suggested environment-specific adaptation, due to the Kairei vent are rich in iron [15, 17, 29].

Conclusions

The first chromosome-level genome *C. heheva* living in hydrothermal vent was assembled and annotated. A number of 19 chromosomal scaffolds are constructed

with N50 of 53.24 Mb. The completeness of the genome was confirmed by the BUSCO score of 94.5%. Comparative genome analyses results indicated a number of positively selected genes and expanded genes were involved in DNA repair process. Furthermore, the expanded genes and the unique genes were contributed to iron ion binding for maintaining the iron homeostasis in iron-rich environment adaptation. This data set will provide a valuable resource for further studies on hydrothermal vent adaptations of vent fauna.

Data Availability

The final genome assembly and other associated raw data described in this study are available on ScienceDB (https://doi.org/10.57760/sciencedb.07077). The raw sequencing reads were also deposited at NCBI under BioProject PRJNA934972.

Abbreviations

Gb: Gigabase pairs; Mb: Megabase pairs; BUSCO: Benchmarking Universal Single-Copy Orthologs; PE: Paired End; HiFi: High-Fidelity; Hi-C: High-Throughput Chromosome Conformation Capture; BLAST: Basic Local Alignment Search Tool; NCBI: National Center for Biotechnology Information; KASS: KEGG Automatic Annotation Server; KEGG: Kyoto Encyclopedia of Genes and Genomes; GO: Gene Ontology; NR: NCBI's nonredundant database; RBH: Reciprocal Best Hit; CCS: Circular Consensus Sequencing; bp: Base Pairs; GC: Guanine Cytosine; LINE: Long Interspersed Nuclear Element; LTR: Long Terminal Repeat; SINE: Short Interspersed

Nuclear Elements; FDR: False Discovery Rate.

Additional Files

Supplementary Figure S1. BUSCO completeness assessment of gene sets from 10 genomes in phylogenomic analysis.

Supplementary Figure S2. Essential fatty acids metabolism in brief.

Supplementary Table S1. Data sets used for the 10 species in phylogenomic analysis.

Supplementary Table S2. Statistics of raw sequencing data.

Supplementary Table S3. Statistics of repetitive elements percentage in related echinoderms.

Supplementary Table S4. GO and KEGG enrichment of expanded gene families of *Chiridota heheva*.

Supplementary Table S5. GO and KEGG enrichment of positively selected genes of *Chiridota heheva*.

Supplementary Table S6. GO and KEGG enrichment of unique genes of *Chiridota heheva*.

Competing Interests

The authors declare that they have no competing interests.

Funding

This study was financially supported by the major scientific and technological

projects of Hainan Province (ZDKJ2019011), Strategic Priority Research Program of the Chinese Academy of Sciences (CAS) (XDA22050303), National Key Research and Development Program of China (2016YFC0304905).

Authors’ Contributions

Haibin Zhang led the project. Haibin Zhang and Yujin Pu conceived this study. Yujin Pu performed the experiments by Jun Liu assistance. Yujin Pu assembled the genome and analyzed the genomic data with assistance by Yang Zhou. Yujin Pu wrote the first draft of the manuscript. All authors reviewed the manuscript.

Acknowledgments

We thank the captains, crews, and scientific staffs on the R/V *Tansuo 1*, and the pilots of HOV *Shenhaiyongshi*, for their supported by sample collection. We also thank Dr. El-Hadji Malick Cisse (Hainan University), for improving the language and grammar of this manuscript. Special thanks to the reviewers for their helpful comments and constructive suggestions on the manuscript.

References

1. Miyazaki, JI, Beppu, S, Kajio, S, et al. Dispersal ability and environmental adaptability of deep-sea mussels *Bathymodiolus* (Mytilidae: Bathymodiolinae). Open Journal of Marine Science 2013; 3(1):31-39.
2. Brazelton, W. Hydrothermal vents. Curr Biol 2017; 27(11): 450-452.
3. Sun, J, Zhang, Y, Xu, T, et al. Adaptation to deep-sea chemosynthetic environments as revealed by mussel genomes. Nat Ecol Evol 2017; 1(5): 1-7.
4. Wang, ZF, Shi, XJ, Sun, LX, et al. Evolution of mitochondrial energy metabolism genes associated with hydrothermal vent adaption of Alvinocaridid shrimps. Genes Genom 2017; 39(12): 1367-1376.
5. Cheng, J, Hui, M., Sha, ZL. Transcriptomic analysis reveals insights into deep-sea adaptations of the dominant species, *Shinkaia crosnieri* (Crustacea: Decapoda: Anomura), inhabiting both hydrothermal vents and cold seeps. BMC Genomics 2019; 20(1): 1-16.
6. Lutz, RA. Hydrothermal vent fauna. Reference module in Encyclopedia of Ocean Sciences (Third Edition), 2019; 2: 715-727.
7. Snelgrove, PVR, Grassle, JF. Deep-sea fauna. Reference module in Encyclopedia of Ocean Sciences (Third Edition), 2019; 2: 706-714.
8. Methou, P, Hikosaka, M, Chen C, et al. Symbiont community composition in *Rimicaris kairei* shrimps from Indian Ocean vents with notes on Mineralogy. Appl Environ Microb 2022; 88(8): e00185-22.

9. Prakash, LS, Fernandes, SO, Ingole, B,et al. Biogeochemical Characteristics of Hydrothermal Systems in the Indian Ocean. Systems Biogeochemistry of Major Marine Biomes 2022; 285-313.

10. Cho, B, Kim, D, Bae, H, et al. Unique characteristics of the exoskeleton of bythograeid crab, *Austinograea rodriguezensis* in the Indian Ocean hydrothermal vent (Onnuri vent field). Integr Comp Biol 2020; 60(1): 24-32.

11. Nakamura, K, Watanabe, H, Miyazaki, J, et al. Discovery of new hydrothermal activity and chemosynthetic fauna on the Central Indian Ridge at 18-20 °S. PLoS one 2012; 7(3): e32965.

12. Zhang, J, Sun, QL, Luan, ZD, et al. Comparative transcriptome analysis of *Rimicaris* sp. reveals novel molecular features associated with survival in deep-sea hydrothermal vent. Sci Rep-UK 2017; 7(1): 1-16.

13. Jang, SJ, Ho, PT, Jun, SY, et al. A newly discovered Gigantidas bivalve mussel from the Onnuri Vent Field in the northern Central Indian Ridge. Deep-sea Res Pt I: Oceanographic Research Papers 2020; 161: 103299.

14. Zhou, L, Cao, L, Wang, XC, et al. Metal adaptation strategies of deep-sea Bathymodiolus mussels from a cold seep and three hydrothermal vents in the West Pacific. Sci Total Environ 2020; 707:136046.

15. Warén, A, Bengtson, S, Goffredi, SK, et al. A hot-vent gastropod with iron sulfide dermal sclerites. Science 2003; 302: 1007-1007

16. Okada, S, Chen, C, Watsuji, T, et al. The making of natural iron sulfide nanoparticles in a hot vent snail. PNAS 2019; 116(41): 20376-20381.

17. Sun, J, Chen, C, Miyamoto, N, et al. The Scaly-foot snail genome and implications for the origins of biomineralised armour. Nat Commun 2020a; 11(1): 1-12.

18. Sun, J, Zhou, YD, Chen, C, et al. Nearest vent, dearest friend: biodiversity of Tiancheng vent field reveals cross-ridge similarities in the Indian Ocean. Roy Soc open Sci 2020b; 7(3): 200110.

19. Wang, K, Shen, YJ, Yang, YZ, et al. Morphology and genome of a snailfish from the Mariana Trench provide insights into deep-sea adaptation. Nat Ecol Evol 2019; 3(5): 823-833.

20. Liu, RY, Liu, J, Zhang, HB. Positive selection analysis reveals the deep-sea adaptation of a hadal sea cucumber (*Paelopatides* sp.) to the Mariana Trench. J Oceanol Limnol 2021; 39(1), 266-281.

21. Zhang, L, He, J, Tan, PP, et al. The genome of an apodid holothuroid (*Chiridota heheva*) provides insights into its adaptation to a deep-sea reducing environment. Commun Biol 2022; 5: 224.

22. Yuan, JB, Zhang, XJ, Gao, Y, et al. Adaptation and molecular evidence for convergence in decapod crustaceans from deep- sea hydrothermal vent environments. Mol Ecol 2020; 29(20): 3954-3969.

23. Shao G, He T, Mu Y, et al. The genome of a hadal sea cucumber reveals novel adaptive strategies to deep-sea environments. Iscience 2022; 105545.

24. WoRMS. https://www.marinespecies.org/aphia.php?p=taxdetails&id=123083. Accessed 11 October 2022.

25. Thomas, EA, Liu, RY, Amon, D, et al. *Chiridota heheva*-the cosmopolitan holothurian. Mar Biodivers 2020; 50(6): 1-13.

26. Sun, SE, Sha, ZL, Xiao N. The first two complete mitogenomes of the order Apodida from deep-sea chemoautotrophic environments: New insights into the gene rearrangement, origin and evolution of the deep-sea sea cucumbers. Comparative Biochemistry and Physiology Part D: Genomics and Proteomics, 2021; 39: 100839.

27. Zhou, Y, Zhang, DS, Zhang, RY, et al. Characterization of vent fauna at three hydrothermal vent fields on the Southwest Indian Ridge: Implications for biogeography and interannual dynamics on ultraslow-spreading ridges. Deep Sea Research Part I: Oceanographic Research Papers 2018; 137: 1-12.

28. Humphris, SE, Fornari, DJ. Hydrothermal vents in an unusual geotectonic setting: the Kairei and Edmond vent fields, Central Indian Ridge[C]//AGU Fall Meeting Abstracts. 2001, 2001: OS41A-0444.

29. Wang, YJ, Han, XQ, Petersen, S, et al. Trace metal distribution in sulfide minerals from Ultramafic-Hosted hydrothermal systems: examples from the Kairei vent field, central indian ridge. Minerals 2018; 8(11):526.

30. Xia, YM, Chen, FS, Du, Y, et al. A modified SDS-based DNA extraction method from raw soybean. Bioscience Rep 2019; 39(2): BSR20182271.

31. Belton, J-M, McCord, RP, Gibcus, JH, et al. Hi-C: A comprehensive technique to capture the conformation of genomes. Methods 2012; 58(3): 268-276.

32. Cheng, H, Concepcion, GT, Feng, X, et al. Haplotype-resolved de novo assembly

using phased assembly graphs with hifiasm. Nat Methods 2021; 18: 170-175.

33. Guan, DF, McCarthy, SA, Wood, J, et al. Identifying and removing haplotypic duplication in primary genome assemblies. Bioinformatics 2020; 36(9): 2896-2898.

34. Durand, NC, Shamim, MS, Machol, I, et al. Juicer provides a one-click system for analyzing loop-resolution Hi-C experiments. Cell Syst 2016; 3(1): 95-98.

35. Dudchenko, O, Batra, SS, Omer, AD, et al. De novo assembly of the Aedes aegypti genome using Hi-C yields chromosome-length scaffolds . Science 2017; 356(6333): 92-95.

36. Durand, NC, Robinson, JT, Shamim, MS, et al. Juicebox Provides a Visualization System for Hi-C Contact Maps with Unlimited Zoom. Cell Syst 2016; 3(1): 99-101.

37. Manni, M, Berkeley, MR, Seppey, M, et al. BUSCO update: Novel and streamlined workflows along with broader and deeper phylogenetic coverage for scoring of eukaryotic, prokaryotic, and viral genomes. Mol Biol Evol 2021; 38(10): 4647-4654.

38. Flynn, JM, Hubley, R, Goubert, C, et al. RepeatModeler2 for automated genomic discovery of transposable element families. PNASciences 2020; 117(17): 9451-9457.

39. Tarailo-Graovac, M, Chen N. Using RepeatMasker to identify repetitive elements in genomic sequences. Curr Protoc Bioinform. 2009; 25(1): 4.10.1-4.10.14

40. Stanke, M, Diekhans, M, Baertsch, R, et al. Using native and syntenically mapped

cDNA alignments to improve de novo gene finding. Bioinformatics 2008; 24(5): 637-644.

41. Majoros, WH, Pertea, M, Salzberg, SL. TigrScan and GlimmerHMM: two open source ab initio eukaryotic gene-finders. Bioinformatics 2004; 20(16): 2878-2879.

42. Alioto, T, Blanco, E, Parra, G, et al. Using geneid to Identify Genes. Current Protocols in Bioinformatics 2018; e56.

43. Slater, GS, Birney, E. Automated generation of heuristics for biological sequence comparison. BMC Bioinformatics 2005; 6:31.

44. Haas, BJ, Zeng, Q, Pearson, MD, et al. Approaches to Fungal Genome Annotation. Mycology 2011; 2(3):118-141.

45. Haas, BJ, Salzberg, SL, Zhu, W, et al. Automated eukaryotic gene structure annotation using EVidenceModeler and the Program to Assemble Spliced Alignments. Genome Biol 2008; 9(1): R7.

46. Buchfink, B, Reuter, K, Drost, H-G. Sensitive protein alignments at tree-of-life scale using DIAMOND. Nat Methods 2021;18(4):366-8.

47. Jones, P, Binns, D, Chang, H-Y, et al. InterProScan 5: genome-scale protein function classification. Bioinformatics 2014; 30(9): 123-1240.

48. Emms, DM, Kelly, S. OrthoFinder: phylogenetic orthology inference for comparative genomics. Genome Biol 2019; 20(1).

49. Katoh, K., Standley, D. M. MAFFT Multiple sequence alignment software version 7: Improvements in performance and usability. Mol Biol Evol 2013; 30(4): 772-780.

50. Stamatakis, A. RAxML version 8: a tool for phylogenetic analysis and post-analysis of large phylogenies. Bioinformatics 2014; 30(9): 1312-1313.

51. Yang, Z. PAML 4: Phylogenetic analysis by maximum likelihood. Mol Biol and Evol 2007; 24(8): 1586-1591.

52. Wang, YX, Yang, YJ, Li, YL, et al. Identification of sex determination locus in sea cucumber *Apostichopus japonicus* using genome-wide association study. BMC Genomics 2022; 23: 391.

53. Camacho, C, Coulouris, G, Avagyan, V, Ma, et al. BLAST+: architecture and applications. BMC Bioinformatics 2009; 10:421.

54. Tang, HB, Krishnakumar V, Li, J. jcvi: JCVI utility libraries. Zenodo 2015.

55. De Bie, T, Cristianini, N, Demuth, JP, et al. CAFE: a computational tool for the study of gene family evolution. Bioinformatics 2006; 22(10): 1269-1271.

56. Barker, MS, Dlugosch, KM, Dinh L, et al. EvoPipes.net: Bioinformatic tools for ecological and evolutionary genomics. Evol Bioinform 2010; 6.

57. Capella-Gutiérrez, S, Silla-Martínez, JM, Gabaldón, T. trimAl: a tool for automated alignment trimming in large-scale phylogenetic analyses. Bioinformatics 2009; 25(15):1972-3.

58. Sun, JH, Lu, F, Luo, YJ, et al. OrthoVenn3: an integrated platform for exploring and visualizing orthologous data across genomes, Nucleic Acids Research 2023; gkad313.

59. Lex, A, Gehlenborg, N, Strobelt, H.et al. UpSet: visualization of intersecting sets. IEEE Trans. Vis. Comput. Graph. 2014; 20:1983-1992.

60. Jo, J, Oh, J, Lee, HG, et al. Draft genome of the sea cucumber *Apostichopus japonicus* and genetic polymorphism among color variants. Gigascience 2017; 6(1): giw006.

61. Reich M. Different pathways in early evolution of the holothurian calcareous ring. Progress in Echinoderm Palaeobiology 2015; 19: 137-145.

62. Reich M. The early evolution and diversification of holothurians (Echinozoa). Echinoderms: Durham: Taylor and Francis Group, London, 2010a: 55-59.

63. Reich M. The oldest synallactid sea cucumber (Echinodermata: Holothuroidea: Aspidochirotida). Paläontologische Zeitschrift 2010b; 84(4): 541-546.

64. Pierrat, J, Bédier, A, Eeckhaut, I, et al. Sophistication in a seemingly simple creature: a review of wild holothurian nutrition in marine ecosystems. Biol Rev 2022; 97(1): 273-298.

65. Siebenaller, JF, Garrett, DJ. The effects of the deep-sea environment on transmembrane signaling. Comparative Biochemistry and Physiology Part B: Biochemistry and Molecular Biology 2002; 131(4): 675-694.

66. Montagne, K, Uchiyama, H, Furukawa, KS, et al. Hydrostatic pressure decreases membrane fluidity and lipid desaturase expression in chondrocyte progenitor cells. J Biomech 2014; 47(2): 354-359.

67. Tamby, A, Sinninghe Damsté, JS, Villanueva, L. Microbial membrane lipid adaptations to high hydrostatic pressure in the marine environment. Front Mol Biosci 2023; 9:1058381

68. Shrestha, N, Holland, OJ, Kent, NL, et al. Maternal high linoleic acid alters

placental fatty acid composition. Nutrients 2020; 12(8): 2183.

69. Wang, B, Wu, LJ, Chen, J, et al. Metabolism pathways of arachidonic acids: mechanisms and potential therapeutic targets. Sig Transduct Target Ther 2021; 6: 94.

70. Ravingerovά, T, Kindernay, L, Bartekovά, M, et al. The molecular mechanisms of iron metabolism and its role in cardiac dysfunction and cardioprotection. Int. J. Mol. Sci. 2020; 21: 7889.

71. Song, YS, Annalora, AJ, Marcus, CB, et al. Cytochrome P450 1B1: a key regulator of ocular iron homeostasis and oxidative stress. Cells 2022; 11: 2930.

72. Gan, ZB, Yuan, JB, Liu, XM, et al. Comparative transcriptomic analysis of deep-and shallow-water barnacle species (Cirripedia, Poecilasmatidae) provides insights into deep-sea adaptation of sessile crustaceans. BMC genomics 2020; 21(1): 1-13.

Figure legends

Figure 1. The sampling site at the Kairei vent filed of Indian Ocean and the photo in situ at a depth of 2428 m.

Figure 2. Genome assembly and sequencing analysis of the Kairei vent *Chiridota heheva*. (A) Hi-C interaction heat map. (B) High-quality assembly of 19 chromosomes with genes coverage, GC content, and repetitive elements of LTR, LINE and SINE.

Figure 3. Phylogenetic and syntenic relationships. (A) Phylogenetic relationship and divergence time based on 10 metazoan species orthologous from OrthoFinder. The number on the branches represents of gene family expansion (red) or contraction (green) (B) Statistics of orthologous gene numbers in these species. Single-copy orthologs, gene that have only one copy in each species and have homologs in other species; Multiple-copy orthologs, gene that have more than one copy in each species, together with homologs in other species; Unique orthologs, gene in each species without homologs in other species; Other orthologs, orthologs that do not belong to any type of the above orthologs; Unclustered genes, gene that do not clustered. (C) Synteny between the Kairei vent *Chiridota heheva* and *Apostichopus japonicus* in dot plotter.

Figure 4. GO enrichment analysis of expanded gene families of the Kairei vent *Chiridota heheva*.

Figure 5. Positively selected amino acid sites of gene *RFC2* in the Kairei vent *Chiridota heheva*. (A) 10 positively selected amino sites in protein sequence. (B)

Distribution of 10 positively selected amino sites in three dimensional structure from AlphaFold.

Figure 6. UpSet relationship of protein sets of six echinoderms (*A. japonica*, *A. planci*, *O. spiculata*, *S. purpuratus*, the Haima cold seep *C. heheva*, and the Kairei vent *C. heheva*).

Figure 7. GO enrichment analysis of the unique of the Kairei vent *Chiridota heheva*.

Table 1. Assembly statistics of the *Chiridota heheva* genome assembly.

**Assembly statistics**	**Value**
Genome size (bp)	1,434,753,151
Number of scaffolds	1399
Number of chromosome-scale scaffolds	19
N50 of scaffolds (bp)	53,240,875
L50 of scaffolds	11
Chromosome-scale scaffolds (bp)	1,431,787,880
GC content of the genome (%)	37.1231
Error rate	0.00206
**BUSCO analysis**	
Library	Metazoan_odb10 (954)
Complete	94.5% (902)
Complete and single copy	93.5% (892)
Complete and duplicated	1.0% (10)
Fragmented	2.8% (27)
Missing	2.7% (25)

Table 2. Repeatitive elements of the Chiridota heheva genome assembly.

**Assembly feature**	**Number of elements**	**Value (bp)**
DNA	15,768	87,173,116
LINE	305,867	353,178,525
SINE	63,461	11,551,395
LTR	16,380	23,197,590
Low complexity	27,548	1,906,077
Satellite	44,353	22,459,277
Simple repeat	365,433	399,894,357
Small RNA	18,648	2,502,674
Total	70.8%	1,016,101,549
Unknown	1,635,141	380,635,876

Table 3. Annotation statistics of *Chiridota heheva* genome assembly.

**Databases of genes annotation**	**Value**
Number of predicted genes	32,434
Number of predicted protein-coding genes	24,606
Number of genes annotation to Interproscan	16,086
Number of genes annotation to GO	10,566
Number of genes annotation to Pfam	14,171
Number of genes annotation to KEGG	7,244
Number of genes annotation to NR	18,038

Number of genes annotation to Swiss-Prot	10,711
Number of genes annotation toTrEMBL	17,697
**BUSCO analysis**	
Complete	95.0% (907)
Complete and single copy	94.2% (899)
Complete and duplicated	0.8% (8)
Fragmented	1.5% (14)
Missing	3.5% (33)
Total BUSCO groups searched	954

Table 4. The positively selected genes of *Chiridota heheva* from the Kairei vent.

**Gene**	**Description**	**FDR**
MAEA	Macrophage erythroblast attacher	2.99E-04
POLB	DNA polymerase beta	2.99E-04
SOD1	Superoxide dismutase, Cu-Zn family	2.99E-04
URB1	Nucleolar pre-ribosomal-associated protein 1	1.21E-03
FAN1	Fanconi-associated nuclease 1	1.21E-03
RFC2	Replication factor C subunit 2	1.87E-03
FARSA	Phenylalanyl-tRNA synthetase alpha chain	4.87E-03
NUP88	Nuclear pore complex protein Nup88	5.02E-03
KDM2A	F-box and leucine-rich repeat protein 11	5.16E-03
RFT1	Oligosaccharide translocation protein *RFT1*	8.10E-03
RNF216	E3 ubiquitin-protein ligase *RNF216*	1.08E-02
SPG7	Spastic paraplegia 7	1.40E-02
BBOX1	Gamma-butyrobetaine dioxygenase	1.40E-02
RPS16	Small subunit ribosomal protein S16e	1.40E-02
BRCA1	Breast cancer type 1 susceptibility protein	1.60E-02
SDR42E1	Short-chain dehydrogenase/reductase family 42E member 1	2.05E-02
SSF1_2	Ribosome biogenesis protein *SSF1/2*	2.37E-02
LSM4	U6 snRNA-associated Sm-like protein LSm4	2.64E-02
PSTK	O-phosphoseryl-tRNA(Sec) kinase	3.13E-02
ESCO1	N-acetyltransferase	3.14E-02
TTLL9	tubulin polyglutamylase *TTLL9*	3.20E-02
HOGA1	4-hydroxy-2-oxoglutarate aldolase	3.43E-02
DCLK1	Doublecortin-like kinase 1	3.80E-02
LDHD	D-lactate dehydrogenase (cytochrome)	4.14E-02
TEP1	telomerase protein component 1	4.14E-02
SIRT4	NAD+-dependent protein deacetylase sirtuin 4	4.14E-02
SLC35F5	Solute carrier family 35, member F5	4.22E-02
SEH1	Nucleoporin *SEH1*	4.47E-02

Manuscript_revised_with_marks Click here to access/download;Manuscript;Manuscript_revised_with_marks.

~~The chromosome-level genome of *Chiridota heheva* (Holothuroidea, Apodida,Chiridotidae) provides insights into adaptation to the hydrothermal environment~~

A high-quality chromosomal genome assembly of the sea cucumber *Chiridota heheva* and its hydrothermal adaptation

Yujin Pu, Yang Zhou, Jun Liu, Haibin Zhang*

[1] Institute of Deep-sea Science and Engineering, Chinese Academy of Sciences, Sanya 572000, China

[2] University of Chinese Academy of Sciences, Beijing 100049, China

*Correspondding address. Haibin Zhang, Institute of Deep-sea Science and Engineering, Chinese Academy of Sciences, Sanya 572000, China. E-mail: hzhang@idsse.ac.cn

Abstract

Background: *Chiridota heheva* is one of the cosmopolitan holothurian, which has been found well adapted to diverse deep-sea ecosystems, especially chemosynthetic environments. Besides high hydrostatic pressure and limited light, high concentrations of metal ions also represent harsh conditions in~~ the~~ hydrothermal ~~vent~~environment. Few holothurian species can live in such extreme condition. Therefore, it is valuable to elucidate the adaptive genetic mechanisms of *C. heheva* to hydrothermal environment.

Findings: Herein we report a high-quality reference genome assembly of *C. heheva*

from the Kairei vent, which is the first chromosome-level genome of Apodida. The chromosome-level genome size was 1.43 Gb, with a scaffold N50 of 53.24 Mb and BUSCO completeness score of ~~92.40~~94.5 %. Contig sequences were clustered, ordered, and assembled into 19~~ large scaffolds, each corresponding to a~~ natural chromosome. Comparative genome analysis ~~We~~ found that ~~*C. heheva* has~~the expanded gene families~~,~~ and positively selected genes ~~, and unique genes~~of *C. heheva* were involved in DNA ~~protection under high hydrostatic pressure~~damage repair process, and the expanded genes and the unique genes were contributed to ~~regulating mechanism of~~maintaining the iron ~~ions~~homeostasis in iron-enriched environment ~~which enriched in surrounding vent fluid~~. Positively selected gene ~~*RCF2*~~*RFC2* with 10 positively selected sites was played an essential role in DNA repair under ~~high hydrostatic pressure~~the extreme environment. ~~Unique gene *FTH1* could enhance iron tolerance by keeping it in a nontoxic state in an iron-rich hydrothermal vent environment.~~

Conclusions: This first chromosome-level genome assembly of *C. heheva* ~~provides the insight of how holothurians adapt to hydrothermal envinronment~~reveals the hydrothermal adaptation of holothurians. As~~ it also~~ the first chromosome-level genome of Order Apodida, this genome will provide the resource ~~it will help~~ for investigating the evolution of Class Holothuroidea.

Keywords: *Chiridota heheva*, Hi-C, positively selected gene, gene family, unique gene

Date Description

Context

Hydrothermal vents are one of the typical deep-sea chemosynthetically-driven ecosystems that inhabit a wide array of animals and chemosynthetic microbes. The hydrothermal vent environment is characterized by rapid changes in temperature, acidic pH, sulfur compounds, metal, methane, hydrogen, carbon dioxide, and other toxic chemistry, besides high hydrostatic pressure and darkness of the deep sea [1-9]. However, these inhospitable environments had reported as key areas of enrichment for deep-sea life.

The hydrothermal habitat fauna are commonly adapt to the unusual environment with the uncommon physical and chemical properties of vent fluids.~~In order to survive in hydrothermal vents, unusual environmental adaptability of fauna has evolved to adapt to the uncommon physical and chemical properties of vent fluids.~~ Diverse fauna, including Annelida, Arthropoda, Mollusca, Echinodermata, Cnidaria, and Chordata have been described in hydrothermal vents, these vent faunas survive on their unique strategies in the extreme conditions [9]. According to the previous studies of common vent fauna, such as crab *Austinograea rodriguezensis* [10], shrimps *Rimicaris kairei* [11], *Rimicaris* sp. [12], mussel *Gigantidas vrijenhoeki* [13], *Bathymodiolus* mussels [14], and scaly-food gastropods *Chrysomallon squamiferum* [11, 15-18], were evolved to enhance their tolerance of high temperature, metal ion enrichment, and sulfur rich conditions. The adaptive mechanism is like a hard exoskeleton to endure the thermal stress [10], the ion binding enzymes or respiratory

proteins for ion homeostasis and detoxification [11, 14-17]. The hydrothermal vent adaptation of Arthropoda had reported as following. Crab *Austinograea rodriguezensis* in Onnuri vent evolved a hard exoskeleton to endure the barotraumatic and thermal stress [10]. Shrimps *Rimicaris kairei* and *Mirocaris indica* prefer to gather on the chimney structures hosting high-temperature fluids in the Solitaire hydrothermal field based on their high-temperature tolerance [11]. The symbiotic microbial of shrimp *R. kairei* in iron-rich Kairei vent were fueled by the iron for chemosynthesis [8]. Shrimp *Rimicaris* sp. of hydrothermal vent in Desmos adapted to H_2S-rich environment associated with sulfur metabolism and detoxification [12]. On the other hand, hydrothermal vent adaptation of Mollusca had studied a lot in previous researches. Onnuri vent mussel *Gigantidas vrijenhoeki* obtain nutrients through symbiotic relationships with sulfur-oxidizing and methane-oxidizing bacteria [13]. *Bathymodiolus* mussels from hydrothermal vents usually had higher metal concentrations (Fe, Cr, Cd, and Pb) in the metal-rich environment, in which the enriched Fe was binding for enzymes, respiratory proteins, and structural elements, and the Fe content was regulated by CAT for homeostasis and detoxification [14]. Scaly-food gastropods *Chrysomallon squamiferum* could fixate iron as iron sulfide in scales by biomineralization under iron-rich Kairei vent, enhance metal ions tolerance by increasing the expression of metal tolerance protein 9 (*MTP9*), both pathways above were involved in intracellular homoeostasis maintaining [11, 15-17]. Unlike Kairei vent *C. squamiferum*, the Tiancheng *C. squamiferum* was coated in zinc sulfide by different mechanism [18]. Detoxification processes of those enriched chemicals

that dissolved in hydrothermal vent fluids occurred in vent fauna, indicating that vent fauna has a unique adaptive mechanism for these extreme ecosystems. Besides the unusual conditions of hydrothermal vents, vent fauna also needs tothe adaptations for adapt to common deep-sea conditions as high hydrostatic pressure and limited light are inevitable. In previous studies, deep-sea endemic species have developed abilities to survival in the deep sea with high hydrostatic pressure and darkness. Among various In deep-sea fauna, DNA repair, degenerated ossicles, protein activity protection, and cell cycle maintenance have evolved to high hydrostatic pressure adaptation[5, 19-21]. The white body colour, unpigmented skin, scales, and long-wavelength light sensors of marine fauna were ubiquitous in the light-limited deep sea [6, 19-20, 22-23]. To gather knowledge about the genetic basis of adaptation to deep-sea extreme environments are of particular interest.

Holothurians are widely distributed in several ecosystems' ocean, and more than 1,800 specie have been accepted at present [24]. Few holothurian species can live in such extreme condition of hydrothermal environments. *Chiridota heheva*, with the features of inhabiting in all biotopes of the deep-sea ocean [9, 21, 25-27], is a representative case to explore the molecular basis of an adaptive trait in deep-sea extreme conditions. Chiridotidae is one of the represented families of Holothurians in the deep-sea hydrothermal vents [9]. *Chiridota heheva*, a cosmopolitan Chiridotidae holothurian, has been found in hydrothermal vents, cold seeps, and other organic falls [25-26]. *C. heheva* may be an ideal model for studying how marine fauna have adapted to extreme deep-sea chemosynthetic environments. The cold seep adaptations

of *C. heheva* have been reported by Zhang et al. [21]. However, genome information on *C. heheva* in hydrothermal vent is currently unavailable. In the present study, we sequenced the genome of *C. heheva* with sample collected in the Kairei vent. Kairei vent is an ultramafic-hosted system was discovered in the Indian Ocean. Kairei fluids are highly enriched in dissolved Fe (5,400 μM) that leach from the host rock [9, 15, 28-~~28~~29]. We obtained a chromosome-level genome of *C. heheva* by Hi-C technology with an integrated comprehensive gene set. Moreover, comparative genomic analyses were performed to investigate the hydrothermal vent adaptive mechanisms of *C. heheva*. Finally, together with other published genomic data from vent animals, this assembly results can add more information which will helpful to gain insights into the adaptation of the whole vent fauna.

Methods

Sampling and sequencing

The *C. heheva* individual used for genomic sequencing was collected by the maned submersible vehicle '*Shenhaiyongshi*' from the Kairei vent filed in the Mid-Indian Ocean (70.40°E, 25.32°S), with a depth of 2,428 m, on 7 February 2019 (Fig. 1). The sample was dissected and frozen in liquid nitrogen, then send to the Institute of Deep-sea Science and Engineering, Chinese Academy of Science, Sanya, China, and subsequent storage at -80℃ for further analysis.

The high-molecular-weight genomic DNA (gDNA) was prepared manually from body-wall tissue following a modified protocol described previously [30]. Briefly,

tissue was ground with liquid nitrogen freezing and digested at 65℃ in SDS (sodium dodecyl sulfate) buffer [50 mM Tris-HCl, 50 mM EDTA, 3% SDS (w/v)] for 1 h. Then the lysate was treated by Phenol/Chloroform isolated and Isopropanol precipitation. The gDNA was assessed and sheared to ~15 kb fragment length for Pacific Biosciences (PacBio) HiFi sequencing. The HiFi SMRTbell library was constructed with SMRTbell Express Template Prep Kit 2.0 (Pacific Biosciences, California, USA), and the HiFi reads were sequenced using 1 cell on SMRT cells 8M on a PacBio Sequel II platform (PacBio Sequel II System, RRID:SCR_017990). For genome annotation, the total RNA was isolated from gonad and body-wall tissues using a RNeasy Plus Universal Kit (QIAGEN, Hilden, Germany). The total RNA was used to obtain cDNA by reverse transcribing, and then 150 bp paired-end reads were generated on Illumina NovaSeq 6000 platform (Illumina NovaSeq 6000 Sequencing System, RRID:SCR_016387). The sequencing processes above were conducted by Novogene Company, Tianjin, China.

Hi-C library preparation and sequencing from body-wall tissue have been done following the standard protocol described previously [31]. Briefly, crosslinking the grounded body-wall tissue with 4% formaldehyde, digesting the DNA with restriction enzyme MboI (GATC), making the DNA ends with biotin-14-dCTP, ligating the blunt-end fragments, shearing the DNA into 200- to 600 bp fragments by sonication. Finally, the Hi-C sequencing library was constructed and conducted on the Illumina NovaSeq-6000 sequencing platform (PE 150bp). The experiments and sequencing were performed by Novogene Company, Tianjin, China.

Genome assembly and annotation

Hifiasm version 0.16.1-r375 (Hifiasm, RRID:SCR_021069) with default parameters setting was used for PacBio HiFi reads assembly [32]. Purge_dups version 1.2.5 (purge dups, RRID:SCR_021173) was used for redundancy purge of the primary genome and obtained the ~~scaffold-level~~clean genome without the duplicate contigs [33]. Juicer version 1.6 (Juicer, RRID:SCR_017226) was used to analyze Hi-C reads combined with ~~scaffold~~contig-level genome [34]. 3D-DNA version 190716 (3D de novo assembly, RRID:SCR_017227) was used to primarily correct misjoin, order and orient in the scaffold and obtained the potential chromosomal groups [~~34~~35]. ~~Juicerbox~~Juicebox version 1.11.08 was then used to manually order the scaffolds of the result from 3D-DNA [~~35~~36]. The tool 3D-DNA was used again to obtain the final chromosome assembly for further analysis [35]. The completeness of the chromosome-level genome was assessed using BUSCO version 5.1.2 (BUSCO, RRID:SCR_015008) with the metazoa_odb10 lineage data set (954 orthologs) [~~36~~37].

RepeatModeler version 2.0.1 (RepeatModeler, RRID:SCR_015027) [~~37~~38] and RepeatMasker version open-4.0.6 (RepeatMasker, RRID:SCR_012954) [39] were used for searching repetitive elements in the final genome assembly and generated a soft-masked genome with non-redundant data set of repetitive elements. Subsequently, gene structure annotation in the soft-masked genome was predicted by *ab initio* and evidence-based gene prediction as follow. Augustus version 3.4.3 (Augustus, RRID:SCR_008417) [40], GlimmerHMM version 3.0.4 (GlimmerHMM, RRID:SCR 002654) [~~40~~41], and GeneID version 1.4.5 (Entrez Gene, RRID:SCR_002473) [42]

were used in *ab initio* gene prediction. Moreover, Exonerate version 2.2.0 (Exonerate, RRID: SCR_016088) was employed for protein homologous annotation in evidence-based gene prediction [43]. PASA version 2.5.2 (PASA, RRID:SCR_014656) was applied for transcriptomic annotation in evidence-based gene prediction [44]. EVidenceModeler version 1.1.1 (EVidenceModeler, RRID:SCR_014659) produced a weighed consensus protein set by combining the results from *ab initio* gene models and evidence-based gene models [45]. The protein set was used for gene functional annotation as follows. DIAMOND BLASTP version 2.0.14 was used to search protein function in the nr database of NCBI [46], Interproscan version 5 (InterProScan, RRID:SCR_005829) was employed to predict the protein family membership, functional domains and sites in Swiss-Prot, Pfam [47], and KAAS (KEGG Automatic Annotation Server) was applied for KEGG pathways annotated online (https://www.genome.jp/tools/kaas/).

Orthology prediction and phylogenomic analysis

Protein sets of night echinoderm species (*Anneissia japonica*, *Acanthaster planci*, *Asterias rubens*, *Plazaster borealis*, *Ophiothrix spiculata*, *Strongylocentrotus purpuratus*, *Lytechinus variegatus*, *Apostichopus japonicus*, and *C. heheva*) were employed in the orthology identification with *Homo sapiens* as the outgroup (Supplementary Table S1). OrthoFinder version 2.5.4 (OrthoFinder, RRID:SCR_017118) was applied to determine and cluster gene families among these 10 metazoan species [48]. A total of 495 single-copy orthologs among these species

were multiple aligned with MAFFT version 7.475 (MAFFT, RRID:SCR_011811) [49], then concatenated and used for constructing a phylogenomic tree using RAxML version 8.2.3 (RAxML, RRID:SCR_006086) [50] based on the substitution model of GTRGAMMA with 100 bootstraps. The divergence time among these species was estimated using MCMCTREE in PAML version 4.9 (PAML, RRID:SCR_014932) [51~~0~~]. The calibration times were derived from the TimeTree database (http://www.timetree.org/).

Genome synteny analysis

Chromosome-level genome in our study of *C. heheva* (CHEH_vent1.0) and *A. japonicus* (AJH1.0) [52] was selected as comparisons for syntenic analysis. BLAST version 2.9.0 (BLAST Similarity Search, RRID:SCR_008419) with parameter “-evalue 1e-10” was used to identify similar gene pairs [53]. JCVI version 0.18 (RRID:SCR_021641) was used to perform protein sequence alignment between CHEH_vent1.0 and AJH1.0 and filter the BLAST results with parameter “--cscore =0.5” ~~and~~, then search for syntenic blocks in all the genes (jcvi, RRID:SCR_021641) [54]. Subsequently, JCVI was also used to visualize the syntenic results with the graphic command.

Gene family analysis

Based on orthologous gene families and phylogenetic relationships above, CAFE version 4.2.1 (CAFE, RRID: SCR_005983) [55] was used to detect the gene family

expansion and contraction. GO enrichment and KEGG pathway enrichment were performed online (https://www.genescloud.cn/) and were used to investigate the functional properties of the expansion gene families. A conditional P value was calculated for each gene family, and a significantly accelerated rate of expansion families was left while P values were lower than 0.05.

Genes under positive selected

As the number of single-copy orthologous genes from OrthoFinder is limited, orthologs were identified as reciprocal best blast hits using the RBH Ortholog pipeline [56]. A total of 3,269 orthologs identified above were used for tests for positive selection~~positively selected analysis~~. MAFFT version 7.475 (MAFFT, RRID:SCR_011811) [49] was used for multiple aligned, and the alignments of the corresponding DNA codon sequences further trimmed by trimAl version 1.4.1 (trimAl, RRID:SCR_017334) [57]. Positively selected genes and amino acid sites were assessed with the branch model and branch-site model using codeml in PAML package version 4.9 (PAML, RRID:SCR_014932) [~~50~~51]. A likelihood ratio test was conducted, and the false discovery rate (FDR) correction was performed for multiple comparisons. Genes and sites with a corrected FDR <0.05 were defined as evolving under positive selection~~positively selected~~.

Unique genes

Protein sets of six echinoderms (*A. japonica*, *A. planci*, *O. spiculata*, *S.*

purpuratus, the Haima cold seep *C. heheva*, and the Kairei vent *C. heheva*) were employed for orthologous clusters analysis. OrthoVenn3 [58], which is freely accessible at https://orthovenn3.bioinfotoolkits.net, was used to identify clusters basaed on OrthoFinder algorithm among the six echinoderms. The cluster results were visualized by UpSet (RRID:SCR_022731) [59], which could support the overlapped clusters among diverse species and provide the unique clusters among each species. Based on the unique clusters of the Kairei vent *C. heheva*, the unique genes were collected from in the unique clusters and were used to GO enrichment. The UpSet and GO enrichment analyses were automatically run on OrthoVenn3 platform.Based on the GO and KEGG annotation results, the Kairei vent *C. heheva* was compared with Haima cold seep *C. heheva* [21] and *A. japonicus* [51], then obtained its unique genes and visualized by TBtools version 1.1043 (TBtools, RRID:SCR_023018) [57] with Venn diagram function. The functional enrichment analysis of these unique genes was performed online (https://www.genescloud.cn/) by GO and KEGG enrichment

Field Code Changed

Results

Chromosome-scale genome assembly and completeness evaluation

The CCS HiFi reads with 29.25 Gb were sequenced on the PacBio Sequel II platform (Supplementary Table S2). In order to create continuity in the genome assembly, 159.59 Gb of Hi-C reads were further prepared on Illumina NovaSeq 6000 sequencing platform (~ 111 × genome coverage) (Supplementary Table S2). RNA reads with 13.29 Gb were generated on Illumina NovaSeq 6000 sequencing platform

utilized for genome annotation (Supplementary Table S2).

Our chromosome-level genome assembly of *C. heheva* (CHEH_vent1.0) was performed using both HiFi reads and Hi-C reads. The total size of the final assembly was 1.43 Gb with an N50 of 53.24 Mb, consisting of 19 chromosome-level scaffolds with lengths ranging from 30 to 115 Mb (Fig. 2, Table 1). The genome size of this species in the Haima cold seep is 1.107 Gb [21], only three-quarters of the genome size in Kairei vent. BUSCO [37] with a database of metazoan_odb10 was then used to evaluate the integrity of the genome assembly, the results as ~~92.4~~94.5% of the conserved genes indicated the high integrity of our assembled genome in CHEH_vent1.0 (Table 1).

Annotation of repetitive elements and protein-coding genes

Repetitive element annotation identified that in 70.80% (1.02 Gb) of the whole genome assembly, the long interspersed nuclear elements (LINEs) were the largest class of the transposable elements (TEs) annotated, other predominant repetitive elements are summarized in Table 2. Compared with other echinoderms (Supplemental Table S3), the repetitive genes percentage of *C. heheva* (Kairei vent) in this study is more than that 56.64% in Haima Cold Seep [21], and only less than *Paelopatides sp.* Yap 73.93% [23], and the percentage of shallow water *A. japonicus* only have 27.20% [52, ~~58~~60]. After repeat masking, protein-coding genes annotated were using a combination of *ab initio*, homology-based, and transcript-evidence predicted approaches, and a total of 32,434 were successfully identified (Table 3).

Interproscan, KEGG, NR, and UniProt were employed for functional annotations, and 24,606 genes were mapped to at least one database (Table 3).~~ BUSCO benchmarking value of this gene set was summarized as 95.00% of completeness, reflecting high integrity (Table 3).~~

Phylogenetic and syntenic relationship

In order to investigate the phylogenetic relationship between *C. heheva* and other metazoans, 9 species were selected for the phylogenomic tree reconstruction (Supplementary Table S1). A total of 495 single-copy genes in all species with high completeness genomes were used to construct a phylogenomic tree (Fig. 3A, B; Supplemental Fig. S1). *C. heheva* and *A. japonicus* appeared as a sister clade in holothurians, and diverged from other echinoderms approximately 438.1 Mya. The divergence time of holothurians in this study supported the view that holothurians had evolved by the Ordovician [61-~~62~~65].

The syntenic blocks were detected between *C. heheva* and *A. japonicus* using JCVI [~~53~~54] and shown as a dot plotter (Fig. 3C). The results indicated *C. heheva* has a relatively conserved relationship with *A. japonicus*, except chromosomes 1, 3, 4, and 5 of *C. heheva*. We identified chromosome fission or fusion events between the 19 chromosomes of *C. heheva* and 23 chromosomes of *A. japonicus*. Chromosome 1 of *C. heheva* corresponded to chromosomes 4 and 12 of *A. japonicus*, while chromosome 3 corresponded to chromosomes 10 and 21, chromosome 4 corresponded to chromosomes 7 and 17, and chromosome 5 corresponded to

chromosomes 3 and 23, respectively.

Gene family evolution

Based on the phylogenomic tree (Fig. 3A), gene family analysis was performed using CAFE [55]. Compared with the other 9 metazoans,~~–~~ the 450 gene families expansions and 6 contractions occurred since the last common ancestor of *A. japonicus* and *C. heheva*~~450 gene families were expanded, and 6 were contracted in *C. heheva*~~ (Fig. 3A). Collectively, these expanded gene families of *C. heheva* were mainly enriched in membrane functions, nucleoside processes of DNA repair, and proteins activity (Fig. 4; Supplementary Table S4). Membrane-associated processes have been described that were particularly susceptible to perturbation under conditions of high hydrostatic pressure, including reducing the fluidity of lipid bilayers and denaturing membrane-associated proteins [19, 65-66]. Biological membranes are mainly composed of phospholipids, sterols (generally cholesterol), glycolipids, and proteins [66-67]. Phospholipids, with hydrophilic the phosphate group head and hydrophobic the fatty acid tails, are a major component that form the lipid bilayers [67]. Membrane fluidity is modulated by changes in lipid composition, especially the proportion of unsaturated fatty acids [19, 23, 66, 67-68]. As our results, lipid metabolisms were activated to response to high hydrostatic pressure, including essential fatty acid of arachidonic acid (AA) metabolism, linoleic acid (LA) metabolism, alpha-linolenic acid (ALA) metabolism, and so on (Fig. 4; Supplementary Table S4). AA, LA, and ALA are polyunsaturated fatty acids (PUFAs),

which is metabolized into various PUFAs during stress (Supplementary Figure S2). Phospholipid-bound AA is the substrate for the synthesis of a range of biologically active compounds, including prostaglandins (PGs), thromboxanes (TXs), and leukotrienes (LTs), epoxyeicosatrienoic acids (EETs), and hydroxyeicosatetraenoic acids (HETEs) [69]. LA is importance in the biosynthesis of AA, while ALA is the precursor of EPA and DHA and then converts into EPA and DHA through metabolism [68]. Some of the PUFAs may bound to receptor on the membrane of cells and related to membrane fluidity adaptation to compensate for environmental changes, especially AA and DHA have been reported in previous studies [19, 23, 68-69]. Iron plays a crucial role in living organisms, and is intimately involvement in numerous biological processes [Ravingerová et al., 2020, Song et al., 2022]. Iron in organisms are mainly bound to heme (heme-iron), transported by transferrin (TF-bound iron), and stored in ferritin (FT-stored iron) for biological functions, which are redox-inert iron in nontoxic forms [Ravingerová et al., 2020]. Nontransferrin-bound iron (NTBI) and other free iron released from iron-bound proteins are potentially toxic form, due to excess redox-active iron induces oxidative stress and causes cell damage [70-71]. Deep-sea fauna, explored in iron-rich hydrothermal vents, have several mechanisms to maintaining the iron homeostasis~~The composition of unsaturated fatty acids is involved in membrane fluidity and maintained transport functions [19, 23, 64]. These expanded gene families related to lipid metabolism may support a high percentage of unsaturated fatty acids in the membrane. Deep-sea fauna explored in iron-rich hydrothermal vents evolved strategies to tolerate high loads of iron, such as~~

transforming toxic iron to safe iron status, and transporting iron for maintaining homeostasis [9, 14, 16-17]. Cytochrome P450 (CYP) was reported as an important role in the regulation of iron levels to maintain cellular redox homeostasis and against oxidative stress [71]. P450 and transferrin are theThese expanded gene families, which enriched in associated with iron ion binding and metal ion transport respectively, suggesteding the adapting to the iron-rich environment of the Kairei vent. High hydrostatic pressure may damage DNA in deep-sea fauna susceptibly [20]. The responding of DNA repair was variously described in previous studies, including DNA damage detection, replication, recombination, splicing, excision, endonuclease, and so on [5, 20, 23, 72]. The expanded gene families enriched in DNA replication, DNA recombination, DNA-associated protein, and nucleic acid binding, may suggestion a capability of DNA repair.These expanded gene families enriched in DNA replication, recombination, DNA-associated protein, and nucleic acid binding may enhance DNA protected abilities. High hydrostatic pressure inhibits protein functions by affecting folding and enzyme activity [19, 21]. These expanded gene families clustered in protein synthesis and activities of various enzymes were contributed to ensuring the functions of the protein.

Positively selected genes

The positively selected genes support the genetic basis for environmental adaptation. Compared with the other 9 metazoans, 28 positively selected genes were identified in *C. heheva* (Table 4). According to GO enrichment and KEGG enrichment

functional analysis, positively selected genes were significantly enriched for various processes including cyclic compound binding, ion binding, nucleotide binding, ATP binding, and DNA binding and damage repair (Supplementary Table S5). Some genes of deep-sea fauna were positively selected during the adaption to environment.Deep-sea fauna obtained positively selected genes through adaptive evolution. DNA repair genes had been selected for deep-sea adaptation, and may play an important role in maintaining the fidelity of genetic materials in deep-sea environments [5, 20, 23, 72]. Among these processes, at least 11 positively selected genes (*POLB*, *FAN1*, *RFC2*, *KDM2A*, *FARSA*, *SPG7*, *BRCA1*, *TTLL9*, *DCLK1*, *LDHD*, and *SIRT4*) involved in DNA damagerepair that mainly induced by high hydrostatic pressure (Table 4; Supplementary Table S5). Therein DNA repair gene *BRCA1* had been found to protect DNA from high pressure in hadal *Paelopatides* sp. Yap [23]. Furthermore, gene *RCF2RFC2*, which functions as DNA replication, nucleotide excision repair, mismatch repair, DNA repair and recombination proteins, has 10 positively selected sites (Fig. 5A, B). These 10 positively selected sites may enhance the DNA repair abilities of *RCF2RFC2* and reveal potential pathways for enhancing the high hydrostatic pressure tolerance.

Unique genes evolution

In order to investigate the environment-specific adaptation of the Kairei vent *C. heheva*, we identified the unique clusters based on the protein sets of six echinoderms. As the results of UpSet shows, a total of 14,032 clusters were identified in the Kairei

vent *C. heheva*, and 5,132 clusters were shared with the other five echinoderms, while 933 clusters were identified only in the Kairei vent *C. heheva* and were considered as unique clusters (Fig. 6). As the results of GO enrichment analysis, the unique genes of the Kairei vent *C. heheva* were significantly enriched in category of iron binding and transport, including sodium ion transport, ion transport, sodium-independent organic anion transport, metal ion binding, and zinc ion binding (Fig. 7). Among the ion-relative terms, metal ion binding (GO:0046872) exerts an iron ion binding function through CYP2B14P (Cytochrome P450, family 2, subfamily b, polypeptide 14), and CYP 450 has reported that acted as a function of iron homeostasis maintaining [71]. The metal ion binding enriched term of the unique genes of the Kairei vent *C. heheva* suggested environment-specific adaptation, due to the Kairei vent are rich in iron [15, 17, 29].~~To investigate the environment-specific genes, Kairei vent *C. heheva* was compared with Haima Cold Seep *C. heheva* and shallow water *A. japonicus*. As the Venn diagram shows, 305 unique genes in KEGG annotation and 330 unique genes in GO annotation were found in Kairei vent *C. heheva* (Fig. 6A, B). Functional enrichment analyses of GO and KEGG show that unique genes were significantly enriched for various enzymatic activity regulation, cellular homeostasis, substances binding, development, and transport (Fig. 6C; Supplementary Table S6). As the vent fauna in the Kairei vent, fauna had to adapt Kairei iron-rich environment [15, 17, 28]. Terms of acireductone dioxygenase [iron(II)-requiring] activity, ferric iron binding, iron ion homeostasis, transition metal ion homeostasis, and cellular chemical homeostasis were enriched to adapt the iron-rich environment in the Kairei~~

~~vent. The mainly unique gene above is ferritin heavy chain 1 (*FTH1*), this gene encodes the heavy subunit of ferritin, the major intracellular iron storage protein, and the function of ferritin is the storage of iron in a soluble and nontoxic state. For the deep-sea adaptation of high hydrostatic pressure, unique genes were also enriched in nucleotide excision repair and homologous recombination pathway for DNA repair.~~

Conclusions

The first chromosome-level genome *C. heheva* living in hydrothermal vent was assembled and annotated. A number of 19 chromosomal scaffolds are constructed with N50 of 53.24 Mb. The completeness of the genome was confirmed by the BUSCO score of ~~92.4~~94.5%. Comparative genome analyses results indicated a number of positively selected genes and expanded genes were~~, expansion, and unique genes had~~ involved in DNA ~~protection and~~ repair process. Furthermore, the~~se~~ expan~~sion~~ded genes and the unique genes were~~ness had~~ contributed to ~~the deposit of iron in the nontoxic state~~iron ion binding for maintaining the iron homeostasis in iron-rich environment adaptation. This data set will provide a valuable resource for further studies on hydrothermal vent adaptations of vent fauna.

Data Availability

The final genome assembly and other associated raw data described in this study are available on ScienceDB (https://doi.org/10.57760/sciencedb.07077). The raw sequencing reads were also deposited at NCBI under BioProject PRJNA934972.

Abbreviations

Gb: Gigabase pairs; Mb: Megabase pairs; BUSCO: Benchmarking Universal Single-Copy Orthologs; PE: Paired End; HiFi: High-Fidelity; Hi-C: High-Throughput Chromosome Conformation Capture; BLAST: Basic Local Alignment Search Tool; NCBI: National Center for Biotechnology Information; KASS: KEGG Automatic Annotation Server; KEGG: Kyoto Encyclopedia of Genes and Genomes; GO: Gene Ontology; NR: NCBI's nonredundant database; RBH: Reciprocal Best Hit; CCS: Circular Consensus Sequencing; bp: Base Pairs; GC: Guanine Cytosine; LINE: Long Interspersed Nuclear Element; LTR: Long Terminal Repeat; SINE: Short Interspersed Nuclear Elements; FDR: False Discovery Rate.

Additional Files

Supplementary Figure S1. BUSCO completeness assessment of gene sets from 10 genomes in phylogenomic analysis.

Supplementary Figure S2. Essential fatty acids metabolism in brief.

Supplementary Table S1. Data sets used for the 10 species in phylogenomic analysis.

Supplementary Table S2. Statistics of raw sequencing data.

Supplementary Table S3. Statistics of repetitive~~repetivite~~ elements percentage in related echinoderms.

Supplementary Table S4. GO and KEGG enrichment of expanded gene families of the Kairei vent *Chiridota heheva*.

Supplementary Table S5. GO and KEGG enrichment of positively selected genes of

the Kairei vent *Chiridota heheva*.

Supplementary Table S6. GO and KEGG enrichment of unique genes of *Chiridota heheva*.

Competing Interests

The authors declare that they have no competing interests.

Funding

This study was financially supported by the major scientific and technological projects of Hainan Province (ZDKJ2019011), Strategic Priority Research Program of the Chinese Academy of Sciences (CAS) (XDA22050303), National Key Research and Development Program of China (2016YFC0304905).

Authors' Contributions

Haibin Zhang led the project. Haibin Zhang and Yujin Pu conceived this study. Yujin Pu performed the experiments by Jun Liu assistance. Yujin Pu assembled the genome and analyzed the genomic data with assistance by Yang Zhou. Yujin Pu wrote the first draft of the manuscript. All authors reviewed the manuscript.

Acknowledgments

We thank the captains, crews, and scientific staffs on the R/V *Tansuo 1*, and the pilots of HOV *Shenhaiyongshi*, for their supported by sample collection. We also thank Dr.

El-Hadji Malick Cisse (Hainan University), for improving the language and grammar of this manuscript. Special thanks to the reviewers for their helpful comments and constructive suggestions on the manuscript.

References

1. Miyazaki, JI, Beppu, S, Kajio, S, et al. Dispersal ability and environmental adaptability of deep-sea mussels *Bathymodiolus* (Mytilidae: Bathymodiolinae). Open Journal of Marine Science 2013; 3(1):31-39.
2. Brazelton, W. Hydrothermal vents. Curr Biol 2017; 27(11): 450-452.
3. Sun, J, Zhang, Y, Xu, T, et al. Adaptation to deep-sea chemosynthetic environments as revealed by mussel genomes. Nat Ecol Evol 2017; 1(5): 1-7.
4. Wang, ZF, Shi, XJ, Sun, LX, et al. Evolution of mitochondrial energy metabolism genes associated with hydrothermal vent adaption of Alvinocaridid shrimps. Genes Genom 2017; 39(12): 1367-1376.
5. Cheng, J, Hui, M., Sha, ZL. Transcriptomic analysis reveals insights into deep-sea adaptations of the dominant species, *Shinkaia crosnieri* (Crustacea: Decapoda: Anomura), inhabiting both hydrothermal vents and cold seeps. BMC Genomics 2019; 20(1): 1-16.
6. Lutz, RA. Hydrothermal vent fauna. Reference module in Encyclopedia of Ocean Sciences (Third Edition), 2019; 2: 715-727.
7. Snelgrove, PVR, Grassle, JF. Deep-sea fauna. Reference module in Encyclopedia of Ocean Sciences (Third Edition), 2019; 2: 706-714.
8. Methou, P, Hikosaka, M, Chen C, et al. Symbiont community composition in *Rimicaris kairei* shrimps from Indian Ocean vents with notes on Mineralogy. Appl Environ Microb 2022; 88(8): e00185-22.

9. Prakash, LS, Fernandes, SO, Ingole, B,et al. Biogeochemical Characteristics of Hydrothermal Systems in the Indian Ocean. Systems Biogeochemistry of Major Marine Biomes 2022; 285-313.

10. Cho, B, Kim, D, Bae, H, et al. Unique characteristics of the exoskeleton of bythograeid crab, *Austinograea rodriguezensis* in the Indian Ocean hydrothermal vent (Onnuri vent field). Integr Comp Biol 2020; 60(1): 24-32.

11. Nakamura, K, Watanabe, H, Miyazaki, J, et al. Discovery of new hydrothermal activity and chemosynthetic fauna on the Central Indian Ridge at 18-20 °S. PLoS one 2012; 7(3): e32965.

12. Zhang, J, Sun, QL, Luan, ZD, et al. Comparative transcriptome analysis of *Rimicaris* sp. reveals novel molecular features associated with survival in deep-sea hydrothermal vent. Sci Rep-UK 2017; 7(1): 1-16.

13. Jang, SJ, Ho, PT, Jun, SY, et al. A newly discovered Gigantidas bivalve mussel from the Onnuri Vent Field in the northern Central Indian Ridge. Deep-sea Res Pt I: Oceanographic Research Papers 2020; 161: 103299.

14. Zhou, L, Cao, L, Wang, XC, et al. Metal adaptation strategies of deep-sea Bathymodiolus mussels from a cold seep and three hydrothermal vents in the West Pacific. Sci Total Environ 2020; 707:136046.

15. Warén, A, Bengtson, S, Goffredi, SK, et al. A hot-vent gastropod with iron sulfide dermal sclerites. Science 2003; 302: 1007-1007

16. Okada, S, Chen, C, Watsuji, T, et al. The making of natural iron sulfide nanoparticles in a hot vent snail. PNAS 2019; 116(41): 20376-20381.

17. Sun, J, Chen, C, Miyamoto, N, et al. The Scaly-foot snail genome and implications for the origins of biomineralised armour. Nat Commun 2020a; 11(1): 1-12.

18. Sun, J, Zhou, YD, Chen, C, et al. Nearest vent, dearest friend: biodiversity of Tiancheng vent field reveals cross-ridge similarities in the Indian Ocean. Roy Soc open Sci 2020b; 7(3): 200110.

19. Wang, K, Shen, YJ, Yang, YZ, et al. Morphology and genome of a snailfish from the Mariana Trench provide insights into deep-sea adaptation. Nat Ecol Evol 2019; 3(5): 823-833.

20. Liu, RY, Liu, J, Zhang, HB. Positive selection analysis reveals the deep-sea adaptation of a hadal sea cucumber (*Paelopatides* sp.) to the Mariana Trench. J Oceanol Limnol 2021; 39(1), 266-281.

21. Zhang, L, He, J, Tan, PP, et al. The genome of an apodid holothuroid (*Chiridota heheva*) provides insights into its adaptation to a deep-sea reducing environment. Commun Biol 2022; 5: 224.

22. Yuan, JB, Zhang, XJ, Gao, Y, et al. Adaptation and molecular evidence for convergence in decapod crustaceans from deep- sea hydrothermal vent environments. Mol Ecol 2020; 29(20): 3954-3969.

23. Shao G, He T, Mu Y, et al. The genome of a hadal sea cucumber reveals novel adaptive strategies to deep-sea environments. Iscience 2022; 105545.

24. WoRMS. https://www.marinespecies.org/aphia.php?p=taxdetails&id=123083. Accessed 11 October 2022.

25. Thomas, EA, Liu, RY, Amon, D, et al. *Chiridota heheva*-the cosmopolitan holothurian. Mar Biodivers 2020; 50(6): 1-13.

26. Sun, SE, Sha, ZL, Xiao N. The first two complete mitogenomes of the order Apodida from deep-sea chemoautotrophic environments: New insights into the gene rearrangement, origin and evolution of the deep-sea sea cucumbers. Comparative Biochemistry and Physiology Part D: Genomics and Proteomics, 2021; 39: 100839.

~~26.~~27. Zhou, Y, Zhang, DS, Zhang, RY, et al. Characterization of vent fauna at three hydrothermal vent fields on the Southwest Indian Ridge: Implications for biogeography and interannual dynamics on ultraslow-spreading ridges. Deep Sea Research Part I: Oceanographic Research Papers 2018; 137: 1-12.

~~27.~~28. Humphris, SE, Fornari, DJ. Hydrothermal vents in an unusual geotectonic setting: the Kairei and Edmond vent fields, Central Indian Ridge[C]//AGU Fall Meeting Abstracts. 2001, 2001: OS41A-0444.

~~28.~~29. Wang, YJ, Han, XQ, Petersen, S, et al. Trace metal distribution in sulfide minerals from Ultramafic-Hosted hydrothermal systems: examples from the Kairei vent field, central indian ridge. Minerals 2018; 8(11):526.

~~29.~~30. Xia, YM, Chen, FS, Du, Y, et al. A modified SDS-based DNA extraction method from raw soybean. Bioscience Rep 2019; 39(2): BSR20182271.

~~30.~~31. Belton, J-M, McCord, RP, Gibcus, JH, et al. Hi-C: A comprehensive technique to capture the conformation of genomes. Methods 2012; 58(3): 268-276.

~~31.~~32. Cheng, H, Concepcion, GT, Feng, X, et al. Haplotype-resolved de novo

assembly using phased assembly graphs with hifiasm. Nat Methods 2021; 18: 170-175.

~~32.~~33. Guan, DF, McCarthy, SA, Wood, J, et al. Identifying and removing haplotypic duplication in primary genome assemblies. Bioinformatics 2020; 36(9): 2896-2898.

~~33.~~34. Durand, NC, Shamim, MS, Machol, I, et al. Juicer provides a one-click system for analyzing loop-resolution Hi-C experiments. Cell Syst 2016; 3(1): 95-98.~~Durand, NC, Robinson, JT, Shamim, MS, et al. Juicebox Provides a Visualization System for Hi-C Contact Maps with Unlimited Zoom. Cell Syst 2016; 3(1): 99-101.~~

~~34.~~35. Dudchenko, O, Batra, SS, Omer, AD, et al. De novo assembly of the Aedes aegypti genome using Hi-C yields chromosome-length scaffolds . Science 2017; 356(6333): 92-95.

~~35.~~36. Durand, NC, Robinson, JT, Shamim, MS, et al. Juicebox Provides a Visualization System for Hi-C Contact Maps with Unlimited Zoom. Cell Syst 2016; 3(1): 99-101.~~Durand, NC, Shamim, MS, Machol, I, et al. Juicer provides a one-click system for analyzing loop-resolution Hi-C experiments. Cell Syst 2016; 3(1): 95-98.~~

~~36.~~37. Manni, M, Berkeley, MR, Seppey, M, et al. BUSCO update: Novel and streamlined workflows along with broader and deeper phylogenetic coverage for scoring of eukaryotic, prokaryotic, and viral genomes. Mol Biol Evol 2021; 38(10): 4647-4654.

~~37.~~38. Flynn, JM, Hubley, R, Goubert, C, et al. RepeatModeler2 for automated genomic discovery of transposable element families. PNASciences 2020; 117(17): 9451-9457.

~~38.~~39. Tarailo-Graovac, M, Chen N. Using RepeatMasker to identify repetitive elements in genomic sequences. Curr Protoc Bioinform. 2009; 25(1): 4.10.1-4.10.14

~~39.~~40. Stanke, M, Diekhans, M, Baertsch, R, et al. Using native and syntenically mapped cDNA alignments to improve de novo gene finding. Bioinformatics 2008; 24(5): 637-644.

~~40.~~41. Majoros, WH, Pertea, M, Salzberg, SL. TigrScan and GlimmerHMM: two open source ab initio eukaryotic gene-finders. Bioinformatics 2004; 20(16): 2878-2879.

~~41.~~42. Alioto, T, Blanco, E, Parra, G, et al. Using geneid to Identify Genes. Current Protocols in Bioinformatics 2018; e56.

~~42.~~43. Slater, GS, Birney, E. Automated generation of heuristics for biological sequence comparison. BMC Bioinformatics 2005; 6:31.

~~43.~~44. Haas, BJ, Zeng, Q, Pearson, MD, et al. Approaches to Fungal Genome Annotation. Mycology 2011; 2(3):118-141.

~~44.~~45. Haas, BJ, Salzberg, SL, Zhu, W, et al. Automated eukaryotic gene structure annotation using EVidenceModeler and the Program to Assemble Spliced Alignments. Genome Biol 2008; 9(1): R7.

~~45.~~46. Buchfink, B, Reuter, K, Drost, H-G. Sensitive protein alignments at tree-of-life

scale using DIAMOND. Nat Methods 2021;18(4):366-8.

~~46.~~47. Jones, P, Binns, D, Chang, H-Y, et al. InterProScan 5: genome-scale protein function classification. Bioinformatics 2014; 30(9): 123-1240.

~~47.~~48. Emms, DM, Kelly, S. OrthoFinder: phylogenetic orthology inference for comparative genomics. Genome Biol 2019; 20(1).

~~48.~~49. Katoh, K., Standley, D. M. MAFFT Multiple sequence alignment software version 7: Improvements in performance and usability. Mol Biol Evol 2013; 30(4): 772-780.

~~49.~~50. Stamatakis, A. RAxML version 8: a tool for phylogenetic analysis and post-analysis of large phylogenies. Bioinformatics 2014; 30(9): 1312-1313.

~~50.~~51. Yang, Z. PAML 4: Phylogenetic analysis by maximum likelihood. Mol Biol and Evol 2007; 24(8): 1586-1591.

~~51.~~52. Wang, YX, Yang, YJ, Li, YL, et al. Identification of sex determination locus in sea cucumber *Apostichopus japonicus* using genome-wide association study. BMC Genomics 2022; 23: 391.

~~52.~~53. Camacho, C, Coulouris, G, Avagyan, V, Ma, et al. BLAST+: architecture and applications. BMC Bioinformatics 2009; 10:421.

~~53.~~54. Tang, HB, Krishnakumar V, Li, J. jcvi: JCVI utility libraries. Zenodo 2015.

~~54.~~55. De Bie, T, Cristianini, N, Demuth, JP, et al. CAFE: a computational tool for the study of gene family evolution. Bioinformatics 2006; 22(10): 1269-1271.

~~55.~~56. Barker, MS, Dlugosch, KM, Dinh L, et al. EvoPipes.net: Bioinformatic tools for ecological and evolutionary genomics. Evol Bioinform 2010; 6.

~~56.~~57. Capella-Gutiérrez, S, Silla-Martínez, JM, Gabaldón, T. trimAl: a tool for automated alignment trimming in large-scale phylogenetic analyses. Bioinformatics 2009; 25(15):1972-3.

~~57.~~58. ~~Chen, CJ, Chen, H, Zhang Y, et al. TBtools: an integrative toolkit developed for interactive analyses of big biological data. Mol plant 2020; 13(8): 1194-1202.~~Sun, JH, Lu, F, Luo, YJ, et al. OrthoVenn3: an integrated platform for exploring and visualizing orthologous data across genomes, Nucleic Acids Research 2023; gkad313.

59. Lex, A, Gehlenborg, N, Strobelt, H.et al. UpSet: visualization of intersecting sets. IEEE Trans. Vis. Comput. Graph. 2014; 20:1983-1992.

~~58.~~60. Jo, J, Oh, J, Lee, HG, et al. Draft genome of the sea cucumber *Apostichopus japonicus* and genetic polymorphism among color variants. Gigascience 2017; 6(1): giw006.

~~59.~~61. Reich M. Different pathways in early evolution of the holothurian calcareous ring. Progress in Echinoderm Palaeobiology 2015; 19: 137-145.

~~60.~~62. Reich M. The early evolution and diversification of holothurians (Echinozoa). Echinoderms: Durham: Taylor and Francis Group, London, 2010a: 55-59.

~~61.~~63. Reich M. The oldest synallactid sea cucumber (Echinodermata: Holothuroidea: Aspidochirotida). Paläontologische Zeitschrift 2010b; 84(4): 541-546.

~~62.~~64. Pierrat, J, Bédier, A, Eeckhaut, I, et al. Sophistication in a seemingly simple creature: a review of wild holothurian nutrition in marine ecosystems. Biol Rev 2022; 97(1): 273-298.

~~63.~~65. Siebenaller, JF, Garrett, DJ. The effects of the deep-sea environment on

transmembrane signaling. Comparative Biochemistry and Physiology Part B: Biochemistry and Molecular Biology 2002; 131(4): 675-694.

66. Montagne, K, Uchiyama, H, Furukawa, KS, et al. Hydrostatic pressure decreases membrane fluidity and lipid desaturase expression in chondrocyte progenitor cells. J Biomech 2014; 47(2): 354-359.

67. Tamby, A, Sinninghe Damsté, JS, Villanueva, L. Microbial membrane lipid adaptations to high hydrostatic pressure in the marine environment. Front Mol Biosci 2023; 9:1058381

68. Shrestha, N, Holland, OJ, Kent, NL, et al. Maternal high linoleic acid alters placental fatty acid composition. Nutrients 2020; 12(8): 2183.

69. Wang, B, Wu, LJ, Chen, J, et al. Metabolism pathways of arachidonic acids: mechanisms and potential therapeutic targets. Sig Transduct Target Ther 2021; 6: 94.

70. Ravingerová, T, Kindernay, L, Barteková, M, et al. The molecular mechanisms of iron metabolism and its role in cardiac dysfunction and cardioprotection. Int. J. Mol. Sci. 2020; 21: 7889.

71. Song, YS, Annalora, AJ, Marcus, CB, et al. Cytochrome P450 1B1: a key regulator of ocular iron homeostasis and oxidative stress. Cells 2022; 11: 2930.

~~64. Koyama, S, Kobayashi, H, Inoue, A, et al. Effects of the piezo-tolerance of cultured deep-sea eel cells on survival rates, cell proliferation, and cytoskeletal structures. Extremophiles 2005; 9(6): 449-460.~~

72. Gan, ZB, Yuan, JB, Liu, XM, et al. Comparative transcriptomic analysis of deep-and shallow-water barnacle species (Cirripedia, Poecilasmatidae) provides

Formatted: Numbered + Level: 1 + Numbering Style: 1, 2, 3, … + Start at: 1 + Alignment: Left + Aligned at: 0" + Indent at: 0"

insights into deep-sea adaptation of sessile crustaceans. BMC genomics 2020; 21(1): 1-13.

Figure legends

Figure 1. The sampling site at the Kairei vent filed of Indian Ocean and the photo in situ at a depth of 2428 m.

Figure 2. Genome assembly and sequencing analysis of the Kairei vent *Chiridota heheva*. (A) Hi-C interaction heat map. (B) High-quality assembly of 19 chromosomes with genes coverage, GC content, and repetitive elements of LTR, LINE and SINE.

Figure 3. Phylogenetic and syntenic relationships. (A) Phylogenetic relationship and divergence time based on 10 metazoan species orthologous from OrthoFinder ~~in 10 metazoan species~~. The number on the branches represents of gene family expansion (red) or contraction (green) (B) Statistics of orthologous gene numbers in these species. Single-copy orthologs, gene that have only one copy in each species and have homologs in other species; Multiple-copy orthologs, gene that have more than one copy in each species, together with homologs in other species; Unique orthologs, gene in each species without homologs in other species; Other orthologs, orthologs that do not belong to any type of the above orthologs; Unclustered genes, gene that do not clustered. (C) Synteny between the Kairei vent *Chiridota heheva* and *Apostichopus japonicus* in dot plotter.

Figure 4. GO enrichment analysis of expanded gene families of the Kairei vent *Chiridota heheva*.

Figure 5. Positively selected amino acid sites of gene *RFC2* in the Kairei vent *Chiridota heheva*. (A) 10 positively selected amino sites in protein sequence. (B)

Distribution of 10 positively selected amino sites in three dimensional structure from AlphaFold.

Figure 6. UpSet relationship of protein sets of six echinoderms (*A. japonica*, *A. planci*, *O. spiculata*, *S. purpuratus*, the Haima cold seep *C. heheva*, and the Kairei vent *C. heheva*).

Figure 7. GO enrichment analysis of the unique of the Kairei vent *Chiridota heheva*.

Table 1. Assembly statistics of the *Chiridota heheva* genome assembly.

**Assembly statistics**	**Value**
Genome size (bp)	1,434,753,151
Number of scaffolds	1399
Number of chromosome-scale scaffolds	19
N50 of scaffolds (bp)	53,240,875
L50 of scaffolds	11
Chromosome-scale scaffolds (bp)	1,431,787,880
GC content of the genome (%)	37.1231
Error rate	0.00206
**BUSCO analysis**	
Library	Metazoan_odb10 (954)
Complete	992.404.5% (881902)
Complete and single copy	91.1093.5% (869892)
Complete and duplicated	1.1.30% (1210)
Fragmented	3.02.80% (2927)
Missing	4.62.70% (4425)

Table 2. Repeatitive elements of the Chiridota heheva genome assembly.

**Assembly feature**	**Number of elements**	**Value (bp)**
DNA	15,768	87,173,116
LINE	305,867	353,178,525
SINE	63,461	11,551,395
LTR	16,380	23,197,590
Low complexity	27,548	1,906,077
Satellite	44,353	22,459,277
Simple repeat	365,433	399,894,357
Small RNA	18,648	2,502,674
Total	70.8%	1,016,101,549
Unknown	1,635,141	380,635,876

Table 3. Annotation statistics of *Chiridota heheva* genome assembly.

**Databases of genes annotation**	**Value**
Number of predicted genes	32,434
Number of predicted protein-coding genes	24,606
Number of genes annotation to Interproscan	16,086
Number of genes annotation to GO	10,566
Number of genes annotation to Pfam	14,171
Number of genes annotation to KEGG	7,244
Number of genes annotation to NR	18,038

Number of genes annotation to Swiss-Prot	10,711
Number of genes annotation toTrEMBL	17,697
**BUSCO analysis**	
Complete	95.0% (907)
Complete and single copy	94.2% (899)
Complete and duplicated	0.8% (8)
Fragmented	1.5% (14)
Missing	3.5% (33)
Total BUSCO groups searched	954

Table 4. The positively selected genes of *Chiridota heheva* from the Kairei vent.

**Gene**	**Description**	**FDR**
MAEA	Macrophage erythroblast attacher	2.99E-04
POLB	DNA polymerase beta	2.99E-04
SOD1	Superoxide dismutase, Cu-Zn family	2.99E-04
URB1	Nucleolar pre-ribosomal-associated protein 1	1.21E-03
FAN1	Fanconi-associated nuclease 1	1.21E-03
RFC2	Replication factor C subunit 2	1.87E-03
FARSA	Phenylalanyl-tRNA synthetase alpha chain	4.87E-03
NUP88	Nuclear pore complex protein Nup88	5.02E-03
KDM2A	F-box and leucine-rich repeat protein 11	5.16E-03
RFT1	Oligosaccharide translocation protein *RFT1*	8.10E-03
RNF216	E3 ubiquitin-protein ligase *RNF216*	1.08E-02
SPG7	Spastic paraplegia 7	1.40E-02
BBOX1	Gamma-butyrobetaine dioxygenase	1.40E-02
RPS16	Small subunit ribosomal protein S16e	1.40E-02
BRCA1	Breast cancer type 1 susceptibility protein	1.60E-02
SDR42E1	Short-chain dehydrogenase/reductase family 42E member 1	2.05E-02
SSF1_2	Ribosome biogenesis protein *SSF1/2*	2.37E-02
LSM4	U6 snRNA-associated Sm-like protein LSm4	2.64E-02
PSTK	O-phosphoseryl-tRNA(Sec) kinase	3.13E-02
ESCO1	N-acetyltransferase	3.14E-02
TTLL9	tubulin polyglutamylase *TTLL9*	3.20E-02
HOGA1	4-hydroxy-2-oxoglutarate aldolase	3.43E-02
DCLK1	Doublecortin-like kinase 1	3.80E-02
LDHD	D-lactate dehydrogenase (cytochrome)	4.14E-02
TEP1	telomerase protein component 1	4.14E-02
SIRT4	NAD+-dependent protein deacetylase sirtuin 4	4.14E-02
SLC35F5	Solute carrier family 35, member F5	4.22E-02
SEH1	Nucleoporin *SEH1*	4.47E-02

Figure_rivised
Click here to access/download;Figure;Figure_revised.doc

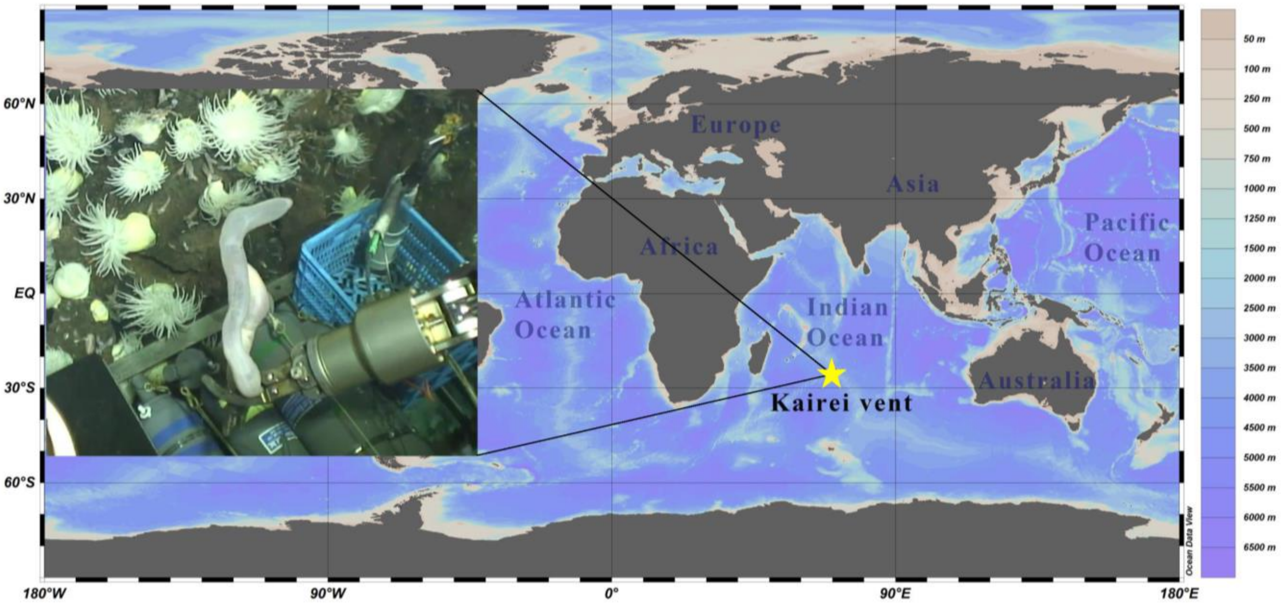

Figure 1

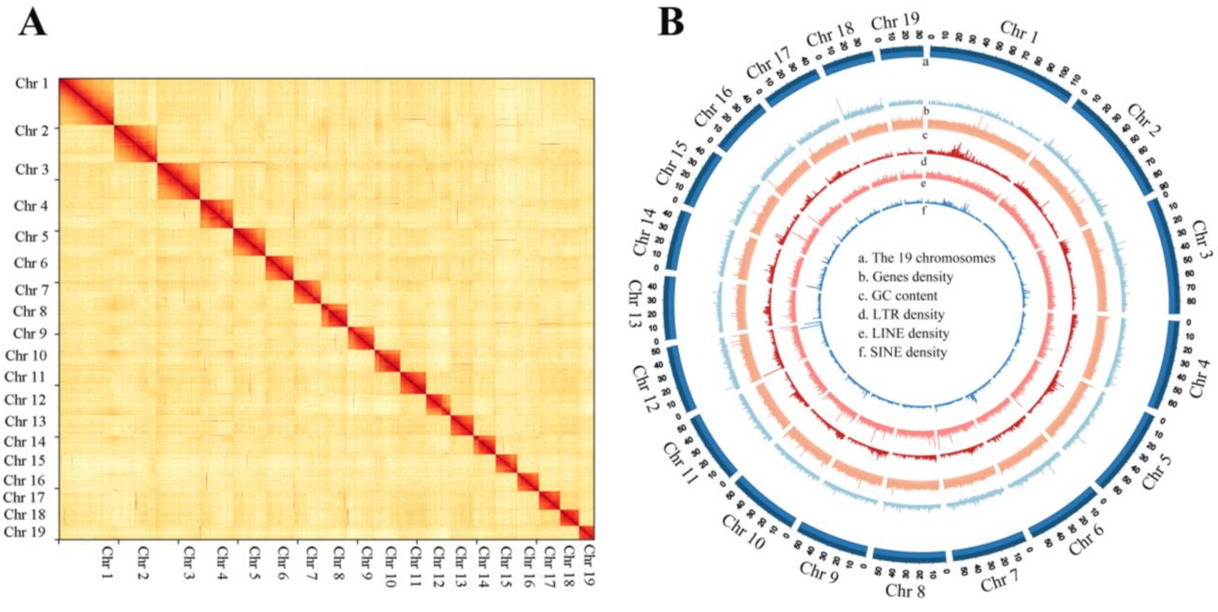

Figure 2

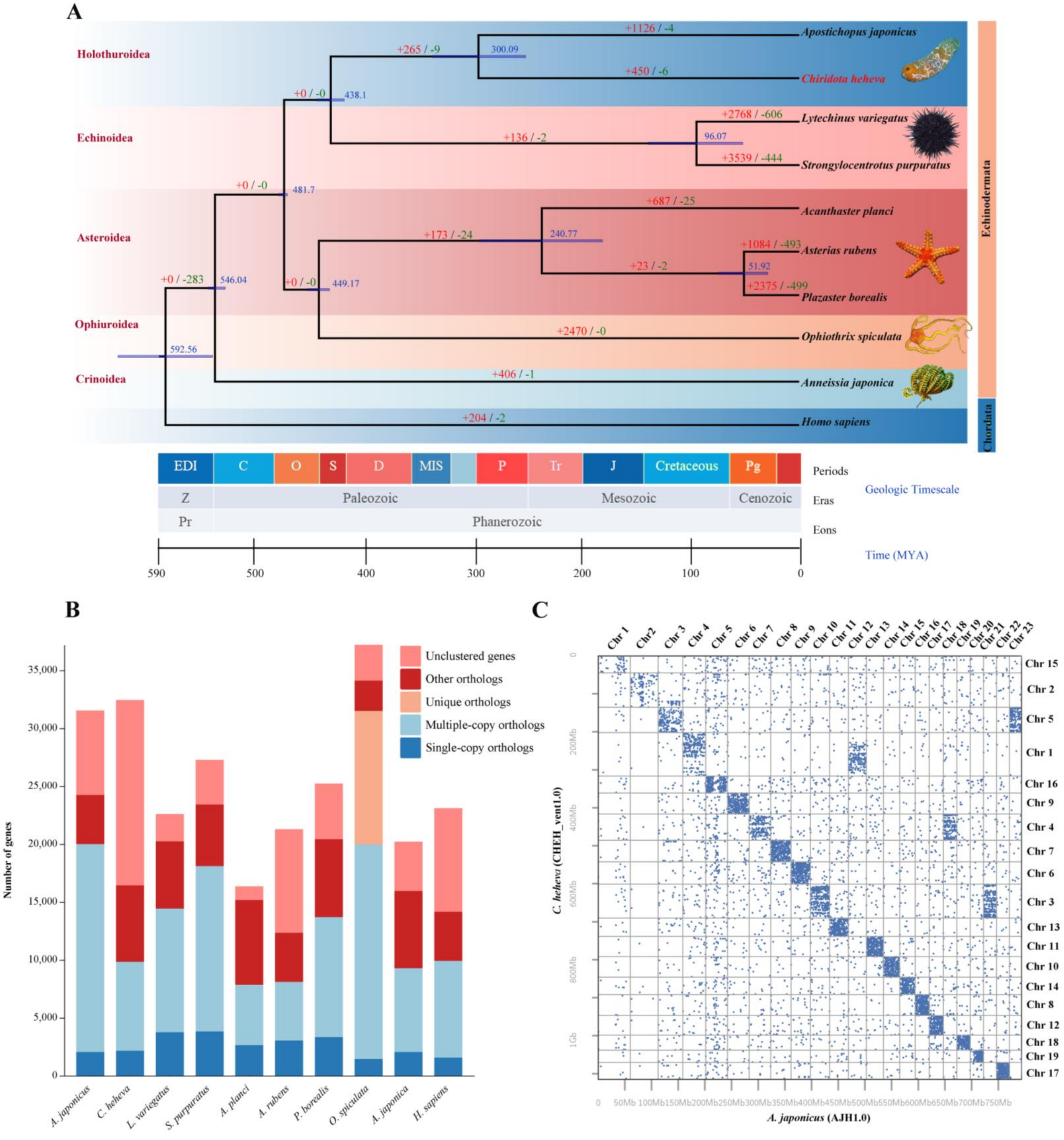
A
Holothuroidea
Echinoidea
Asteroidea
Ophiuroidea
Crinoidea
+1126 / -4
Apostichopus japonicus
+265 / -9
300.09
+450 / -6
Chiridota heheva
+0 / -0
438.1
+2768 / -606
Lytechinus variegatus
+136 / -2
96.07
+3539 / -444
Strongylocentrotus purpuratus
+0 / -0
481.7
+687 / -25
Acanthaster planci
+173 / -24
240.77
+1084 / -493
Asterias rubens
+23 / -2
51.92
+2375 / -499
Plazaster borealis
+0 / -283
546.04
+0 / -0
449.17
+2470 / -0
Ophiothrix spiculata
592.56
+406 / -1
Anneissia japonica
+204 / -2
Homo sapiens
Echinodermata
Chordata
EDI
C
O
S
D
MIS
P
Tr
J
Cretaceous
Pg
Periods
Z
Paleozoic
Mesozoic
Cenozoic
Eras
Pr
Phanerozoic
Eons
Geologic Timescale
Time (MYA)
590
500
400
300
200
100
0
B
Number of genes
35,000
30,000
25,000
20,000
15,000
10,000
5,000
0
Unclustered genes
Other orthologs
Unique orthologs
Multiple-copy orthologs
Single-copy orthologs
A. japonicus
C. heheva
L. variegatus
S. purpuratus
A. planci
A. rubens
P. borealis
O. spiculata
A. japonica
H. sapiens
C
Chr 1
Chr 2
Chr 3
Chr 4
Chr 5
Chr 6
Chr 7
Chr 8
Chr 9
Chr 10
Chr 11
Chr 12
Chr 13
Chr 14
Chr 15
Chr 16
Chr 17
Chr 18
Chr 19
Chr 20
Chr 21
Chr 22
Chr 23
Chr 15
Chr 2
Chr 5
Chr 1
Chr 16
Chr 9
Chr 4
Chr 7
Chr 6
Chr 3
Chr 13
Chr 11
Chr 10
Chr 14
Chr 8
Chr 12
Chr 18
Chr 19
Chr 17
C. heheva (CHEH_vent1.0)
0
200Mb
400Mb
600Mb
800Mb
1Gb
0
50Mb
100Mb
150Mb
200Mb
250Mb
300Mb
350Mb
400Mb
450Mb
500Mb
550Mb
600Mb
650Mb
700Mb
750Mb
A. japonicus (AJH1.0)

Figure 3

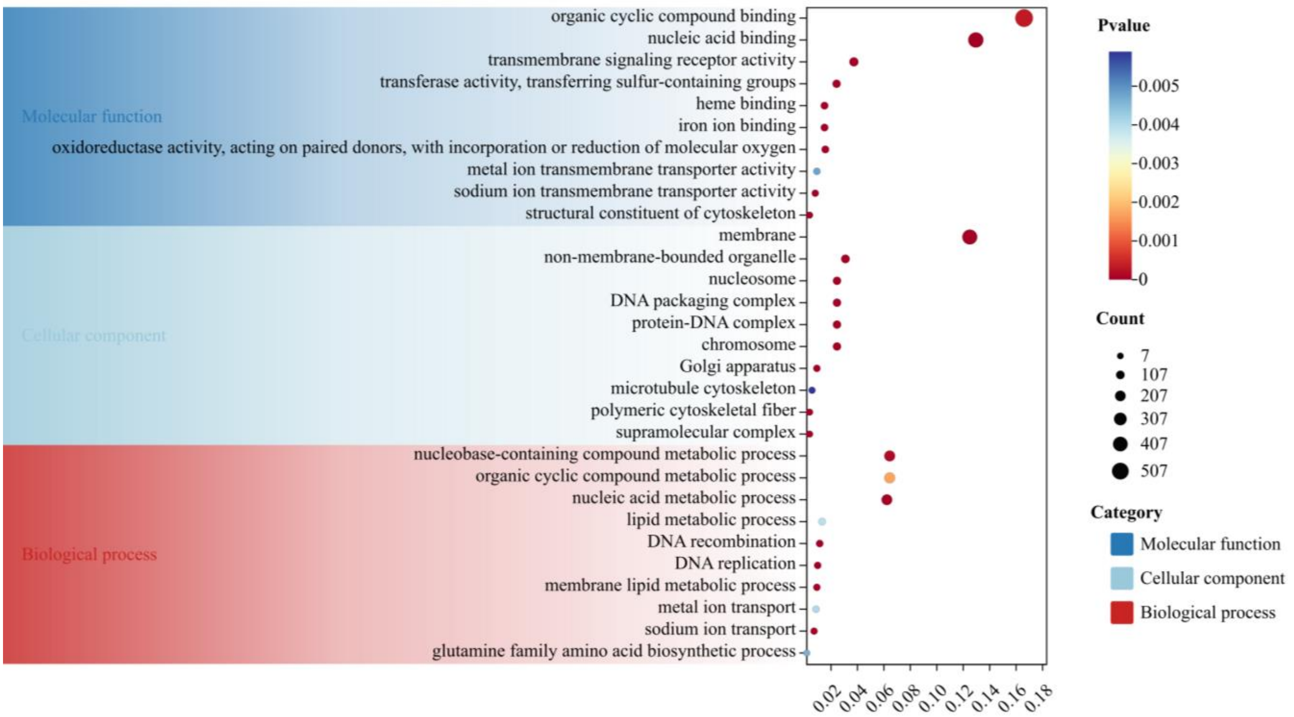
Molecular function
Cellular component
Biological process
organic cyclic compound binding
nucleic acid binding
transmembrane signaling receptor activity
transferase activity, transferring sulfur-containing groups
heme binding
iron ion binding
oxidoreductase activity, acting on paired donors, with incorporation or reduction of molecular oxygen
metal ion transmembrane transporter activity
sodium ion transmembrane transporter activity
structural constituent of cytoskeleton
membrane
non-membrane-bounded organelle
nucleosome
DNA packaging complex
protein-DNA complex
chromosome
Golgi apparatus
microtubule cytoskeleton
polymeric cytoskeletal fiber
supramolecular complex
nucleobase-containing compound metabolic process
organic cyclic compound metabolic process
nucleic acid metabolic process
lipid metabolic process
DNA recombination
DNA replication
membrane lipid metabolic process
metal ion transport
sodium ion transport
glutamine family amino acid biosynthetic process
0.02 0.04 0.06 0.08 0.10 0.12 0.14 0.16 0.18
Pvalue
0.005
0.004
0.003
0.002
0.001
0
Count
7
107
207
307
407
507
Category
Molecular function
Cellular component
Biological process

Figure 4

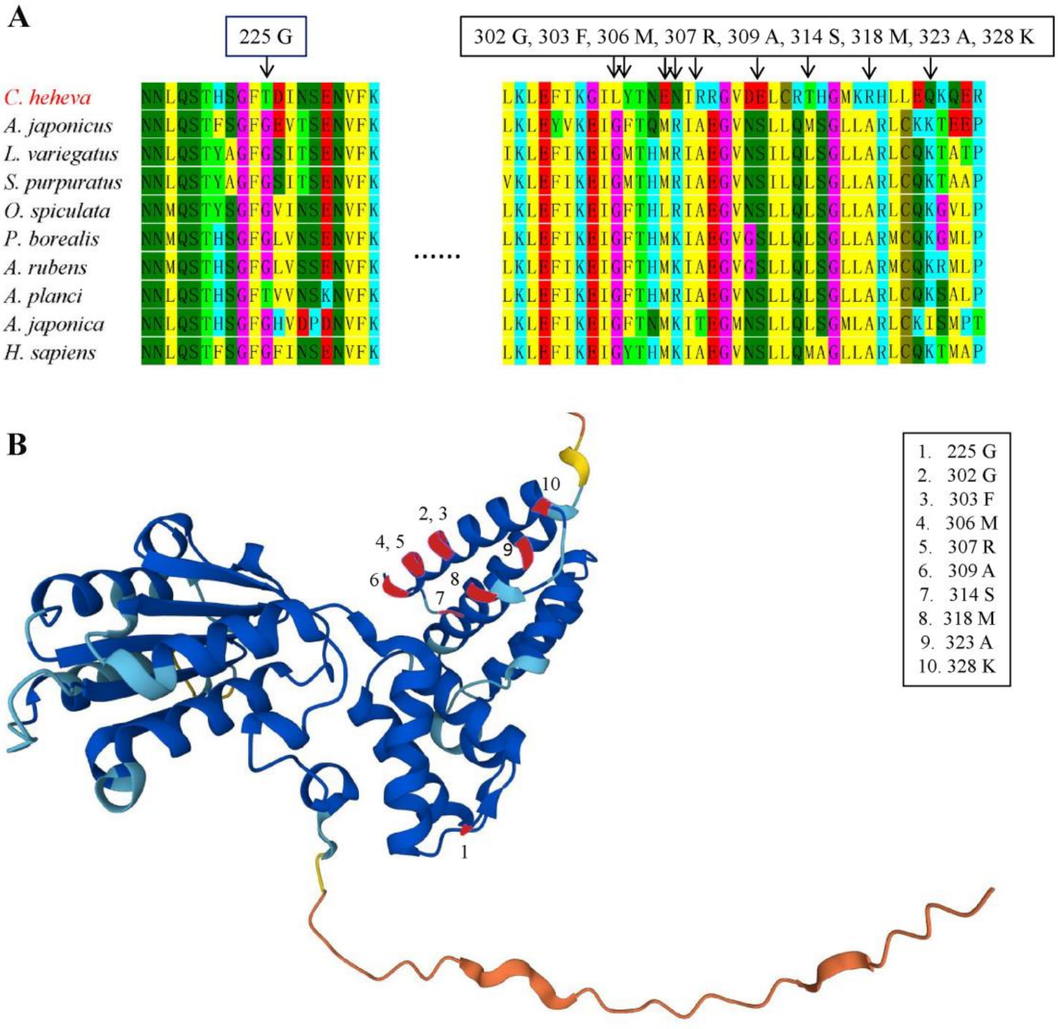
A
225 G
302 G, 303 F, 306 M, 307 R, 309 A, 314 S, 318 M, 323 A, 328 K
C. heheva
A. japonicus
L. variegatus
S. purpuratus
O. spiculata
P. borealis
A. rubens
A. planci
A. japonica
H. sapiens
......
B
1. 225 G
2. 302 G
3. 303 F
4. 306 M
5. 307 R
6. 309 A
7. 314 S
8. 318 M
9. 323 A
10. 328 K

Figure 5

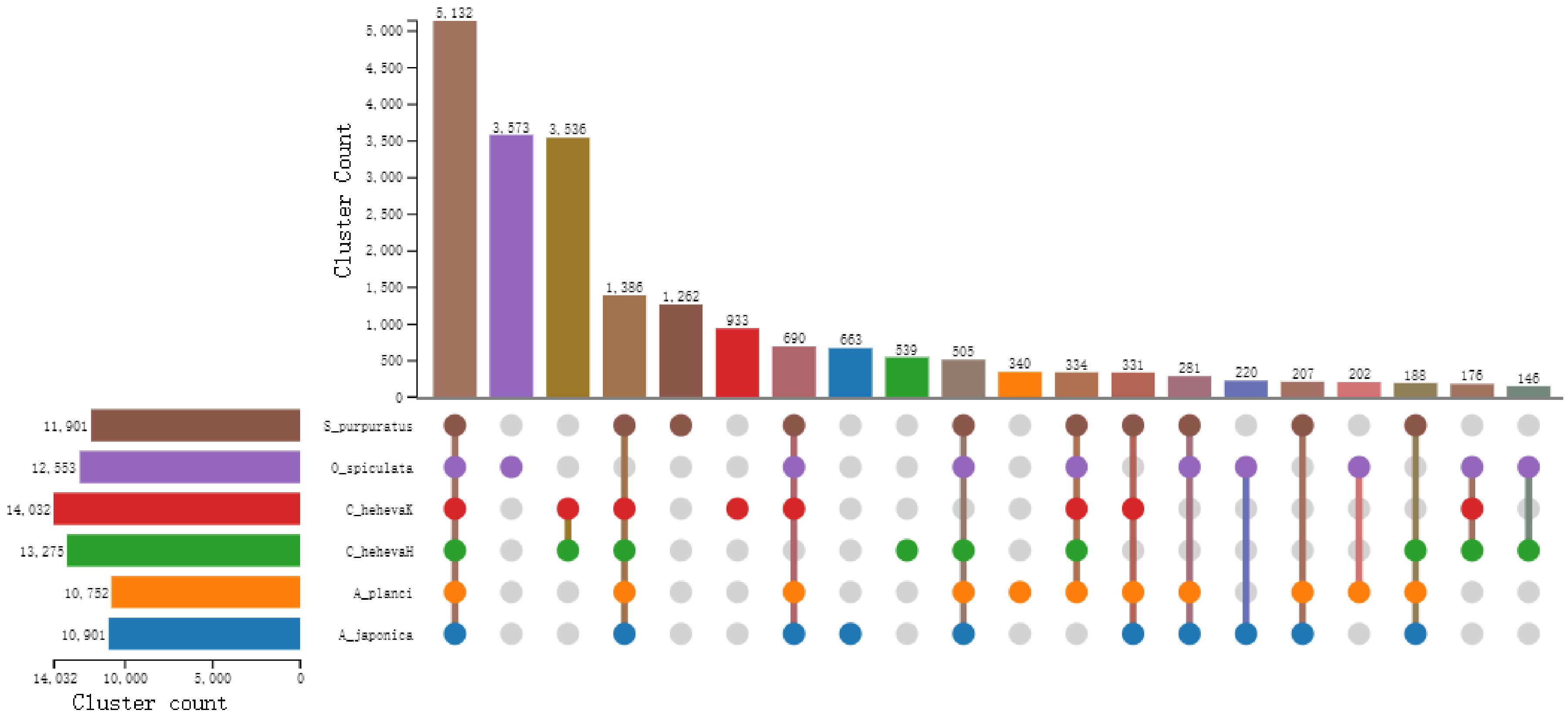

Figure 6

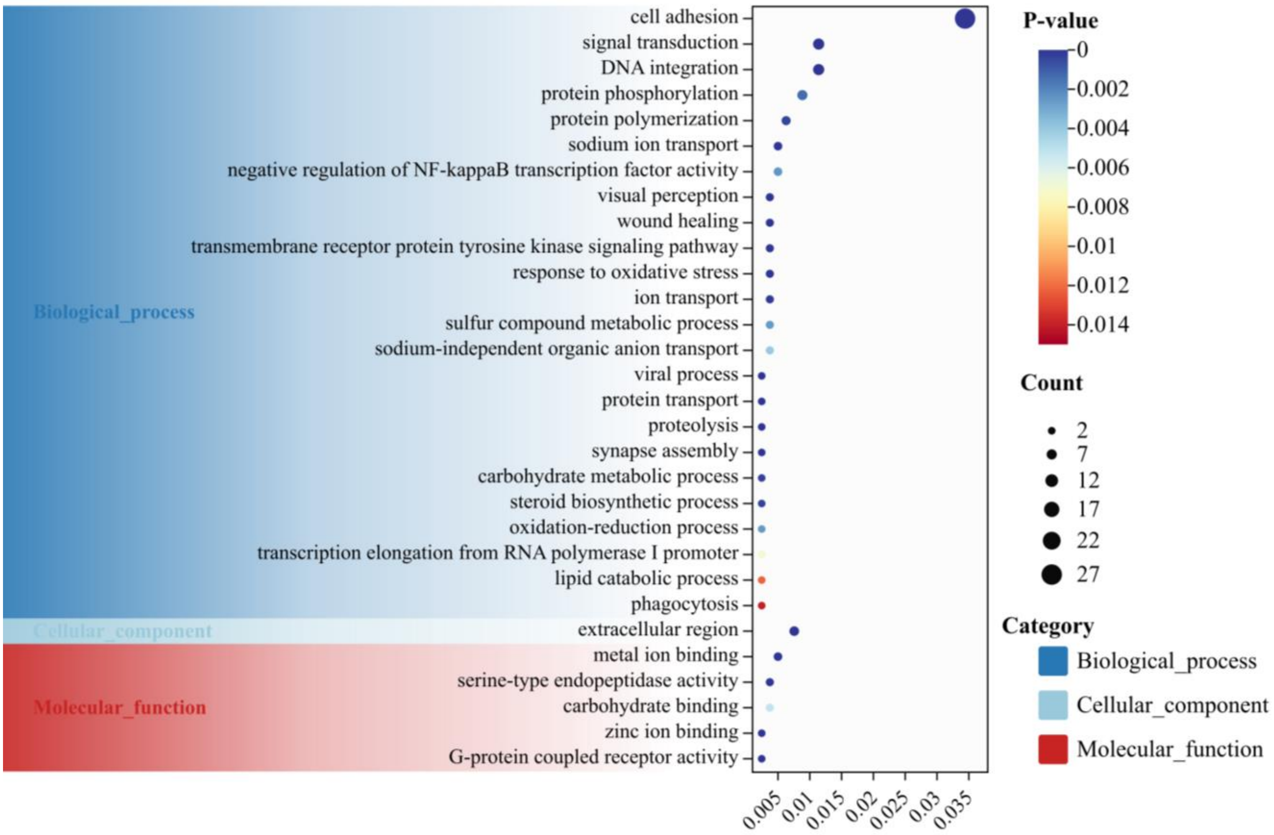
Biological_process
Cellular_component
Molecular_function
cell adhesion
signal transduction
DNA integration
protein phosphorylation
protein polymerization
sodium ion transport
negative regulation of NF-kappaB transcription factor activity
visual perception
wound healing
transmembrane receptor protein tyrosine kinase signaling pathway
response to oxidative stress
ion transport
sulfur compound metabolic process
sodium-independent organic anion transport
viral process
protein transport
proteolysis
synapse assembly
carbohydrate metabolic process
steroid biosynthetic process
oxidation-reduction process
transcription elongation from RNA polymerase I promoter
lipid catabolic process
phagocytosis
extracellular region
metal ion binding
serine-type endopeptidase activity
carbohydrate binding
zinc ion binding
G-protein coupled receptor activity
0.005
0.01
0.015
0.02
0.025
0.03
0.035
P-value
0
0.002
0.004
0.006
0.008
0.01
0.012
0.014
Count
2
7
12
17
22
27
Category
Biological_process
Cellular_component
Molecular_function

Figure 7

Click here to access/download
Supplementary Material
Supplementary Fig. S2.png

Click here to access/download
Supplementary Material
Supplementary Table S6 Reagent and software.xlsx

Click here to access/download
Supplementary Material
Supplementary S1 Commands for analyses.docx
