# Supplementary material for: A high-quality chromosomal genome assembly of the sea cucumber Chiridota heheva and its hydrothermal adaptation
Source: Gigascience. 2024 Jan 4;13:giad107. doi: 10.1093/gigascience/giad107 (PMC10764150; doi:10.1093/gigascience/giad107)
Supplement: giad107_GIGA-D-23-00018_Revision_2 [file giad107_giga-d-23-00018_revision_2.pdf]

GigaScience

A high-quality chromosomal genome assembly of the sea cucumber Chiridota heheva and its hydrothermal adaptation

--Manuscript Draft--

**Manuscript Number:**	GIGA-D-23-00018R2
**Full Title:**	A high-quality chromosomal genome assembly of the sea cucumber Chiridota heheva and its hydrothermal adaptation
**Article Type:**	Data Note
**Funding Information:**	The major scientific and technological projects of Hainan Province (ZDKJ2019011) — professor Haibin Zhang<br>Strategic Priority Research Program of the Chinese Academy of Sciences (CAS) (XDA22050303) — Prof. Jun Liu<br>National Key Research and Development Program of China (2016YFC0304905) — Prof. Jun Liu
**Abstract:**	Abstract<br>Background: Chiridota heheva is a cosmopolitan holothurian well adapted to diverse deep-sea ecosystems, especially chemosynthetic environments. Besides high hydrostatic pressure and limited light, high concentrations of metal ions also represent harsh conditions in hydrothermal environments. Few holothurian species can live in such extreme conditions. Therefore, it is valuable to elucidate the adaptive genetic mechanisms of C. heheva in hydrothermal environments.<br>Findings: Herein, we report a high-quality reference genome assembly of C. heheva from the Kairei vent, which is the first chromosome-level genome of Apodida. The chromosome-level genome size was 1.43 Gb, with a scaffold N50 of 53.24 Mb and BUSCO completeness score of 94.5 %. Contig sequences were clustered, ordered, and assembled into 19 natural chromosomes. Comparative genome analysis found that the expanded gene families and positively selected genes of C. heheva were involved in the DNA damage repair process. The expanded gene families and the unique genes were contributed to maintaining iron homeostasis in an iron-enriched environment. The positively selected gene RFC2 with ten positively selected sites played an essential role in DNA repair under extreme environments.<br>Conclusions: This first chromosome-level genome assembly of C. heheva reveals the hydrothermal adaptation of holothurians. As the first chromosome-level genome of Order Apodida, this genome will provide the resource for investigating the evolution of Class Holothuroidea.
**Corresponding Author:**	Haibin Zhang, Ph.D<br>Institute of Deep-sea Science and Engineering Chinese Academy of Sciences<br>Sanya, Hainan CHINA
**Corresponding Author Secondary Information:**	
**Corresponding Author's Institution:**	Institute of Deep-sea Science and Engineering Chinese Academy of Sciences
**Corresponding Author's Secondary Institution:**	
**First Author:**	Yujin Pu
**First Author Secondary Information:**	
**Order of Authors:**	Yujin Pu<br>Yang Zhou<br>Jun Liu<br>Haibin Zhang, Ph.D
**Order of Authors Secondary Information:**	

Powered by Editorial Manager® and ProduXion Manager® from Aries Systems Corporation

Response to Reviewers:	Response to Reviewers

Response to Reviewers

Reviewer #1: The authors provided more information regarding the analyses of gene family and positively selected genes. However, there are still several problems of the manuscript.

1. There are still many grammatical problems in the revised manuscript. For example:
    1) Line 33, "Date Description"
    2) Line 178, "Genes under positlve selected"
Please check the grammar of the entire manuscript again.

Thank you so much for your careful check. We have checked the grammar and corrected the grammar in the revised manuscript.
For the example mentioned by the reviewer, we have corrected as follows:
Line 33, the statement “Date Description” was corrected as “Data Description.”
Line 178, the statement “Genes under positive selected” was corrected as “Genes under positive selection.”

2.Line 158, the authors should specify all the divergence time they used as calibration time in the MCMCtree analysis.

Thank you for pointing out this issue. We have specified the calibration time according per the reviewer’s suggestion in the revised manuscript (lines 157-159).

3.Line 212, Zhang et al. (2022) estimated the size of C. heheva genome to be 1.2Gb. And the size of their assembled genome is 1.1Gb, which is closed to the estimated size. However, the authors didn't estimate the genome size of the sequenced individual, and claimed that their assembly is better as their assembly is three quarters larger than Zhang et al's assembly. This is not appropriate.

Thank you for pointing out this issue. On one hand, for the first report of one species’ genome, the estimated genome size is often used to determine the coverage depth of Nanopore or PacBio reads. The estimated genome size is also used as the required parameter for WTDBG2 or NextDenovo running of genome assembly. In our study, the basic information of the C. heheva genome has described as Zhang et al. (2022). The sequencing amount and coverage depth of our individual were determined based on genome size as previous report (Zhang et al., 2022). The assembled genome size of our individual is 1.43 Gb, which is also close to the estimated size (1.23 Gb) of the same species (Zhang et al., 2022). The genome assembly of our individual was running by Hifiasm without the parameter of genome size. That is why we did not estimate the genome size of our individual.
On the other hand, to assess the quality of the C. heheva genome assembly, the completeness of the genome assembly was assessed using BUSCO. Complete BUSCOs percentage of C. heheva in the Kairei vent (94.5%) is higher than that in the Haima cold seep (89.6%) (Zhang et al., 2022), which indicate the integrity of our assembled genome is better than Zhang et al.’s (2022). We are very sorry for our incorrect writing. We have re-written this part in the revised manuscript (lines 215-222).

4.Line 214, There are several approaches and softwares (ie. Mercury) to evaluate the quality and completeness of genome assembly (ie. Mercury). However, the author just used the result of BUSCO to evaluate the completeness of genome assembly. It is important to evaluate the quality of the assembly.

BUSCO is the most widely used method for assessing genome assembly based on the conserved metazoan gene set database. We have performed the BUSCO evaluation of our genome assembly and compared the BUSCO results of genome assembly between our individual and Zhang et al. (2022). We have re-written this part in the revised manuscript (lines 215-217).
Considering the reviewer’s suggestion, we also assessed genome assembly quality using Inspector, which evaluates assemblies with only third-generation sequencing reads. We have added the Inspector’s result in the revised manuscript (lines 218-222) and Table 1. The recommended approach, Merqury, was not performed here due to the lack of Illumina dataset.

Powered by Editorial Manager® and ProduXion Manager® from Aries Systems Corporation

5.Line 240, the authors identified the syntenic blocks between C. heheva and A. japonicus. But they didn't draw any conclusion about this analysis.

Thank you for pointing out this issue. According to the reviewer's suggestion, we have re-written this part in the revised manuscript (lines 247-258).

6.Line 302, the authors should describe the results of GO and KEGG separately.

Thank you for pointing out this issue. We agree with the reviewer's suggestion. We have re-written this part to separate the results of GO and KEGG in the revised manuscript (lines 313-318).

Reviewer #2: I thank the authors for their time and detailed revision of their manuscript and addressing the comments. I have a few remaining comments listed here. Overall, I think this genomic resource will be valuable to the broader scientific community. For clarity, my comments reference the line numbers on the version of the manuscript where changes are tracked:

Major Comment:

1.I very respectfully suggest that this manuscript receive another serious round of grammatical corrections from a fluent English speaker as there remain a number of issues, some of which make it difficult to understand the message that the authors are conveying. I think it is important that these language revisions occur prior to publication. I've listed the first three I have come across below as examples:

Line 14: change "is one of the" to "is a"
Line 17: change "environment" to "environments"
Line 25: change "condition" to "conditions"

We apologize for the errors of our manuscript. We have changed "is one of the" to "is a" (line 11), have changed "environment" to "environments" (line 14), and have changed "condition" to "conditions" (line 15). The manuscript has also been double-checked, and the grammar issues we found have been corrected in the revised manuscript by using the track changes mode. We hope that the language level has been substantially improved.

2.Data Availability: I can see the files uploaded to ScienceDB, however, I was unable to access or find any sequencing reads under the provided BioProject ID. Ensuring the accessibility of these data will be important prior to publication.

During the process of creating a data submission, we set the data protection period until December 31, 2023. Before this period, our BioProject's data is available at https://dataview.ncbi.nlm.nih.gov/object/PRJNA934972?reviewer=6lphqpajfogvnfb8laqf4ljajf for reviewers. Please check the reviewer's link for more information. We have also provided this URL to the publisher to share with reviewers. After this period, the data is automatically converted to open access status and its data files are accessible to all visitors.

3.Other minor comments:
Line 46: Hydrothermal vents do not inhabit animals, animals inhabit hydrothermal vents.
Line 226: "aligned" to "alignment"
Line 231: "evolving" to "putatively evolving".
Line 291: "dot plot"
Line 356: "were contributed" to "may contribute"
Line 694: I think the word "genes" is missing after "unique"

We agree with these suggestions. We have modified "Hydrothermal vents are one of the typical deep-sea chemosynthetically-driven ecosystems that inhabit a wide array of animals and chemosynthetic microbes" to "Hydrothermal vents are one of the typical deep-sea chemosynthetically-driven ecosystems with a wide array of animals and chemosynthetic microbes" (lines 35-36), have modified "aligned" to "alignments" (line 183), have modified "evolving" to "putatively evolving" (line 189), have modified "dot

	plotter" to "dot plot" (247), have modified "were contributed" to "may contribute" (line 306), and have modified "unique" to "unique genes" of Figure 7 legend (line 624).<br>We will be happy to edit the text further, based on helpful comments from the reviewers.<br><br>Reviewer #3: 1. The commands file (Supplementary file S1) is adequate although it is impossible to repeat the codeml analysis without details provided in the control file ("paml4.9j/bin/codeml $name_ctl #prepare tree file and ctl file before codeml"). Please provide the contents of the control file in this document.<br>The Resources file looks solid.<br><br>Thank you for pointing out this issue. According to the reviewer's suggestion, we have added the contents of the preparations of tree and ctl files in "Supplementary text S1 Commands for analyses_revised2".<br><br>2.In comment 3, I inquired about the difference between the genomes of C. heheva K and C. heheva H, in terms of size (>300MB difference in size) and gene number (>300 difference in number of genes). The authors address the difference in size. The authors do not address the difference in gene number.<br><br>We are very sorry for our negligence. Thanks for giving us the opportunity to respond to the comments. In the new version, we have re-written the parts of "Unique genes" in Methods and "Unique genes evolution" in Results after have carefully considered all comments from the reviewers. In this part, orthologous clusters were identified using OrthoVenn3 across a range of six echinoderms (A. japonica, A. planci, O. spiculata, S. purpuratus, the Haima cold seep C. heheva, and the Kairei vent C. heheva). The results of OrthoVenn3 presented orthologous clusters in each species, as well as unique and shared homologous gene clusters among species. Those unique genes of the Kairei vent C. heheva were included in the species specific clusters of C. heheva. We hope that this part is clearer now and thanks again for handling our manuscript.<br><br><br>3.I suggested an example of a sentence of how the CAFE analysis should be discussed relative to how it was discussed in the main manuscript. The authors incorporated this sentence into the manuscript. This is fine, except the sentence needs clarification. It currently reads: "Compared with the other 9 metazoans, the 450 gene families expansions and 6 contractions occurred since the last common ancestor of A. japonicus and C. heheva (Fig. 3A)." The sentence as it is written is ambiguous and the authors introduced a grammar mistake by making "family" plural. The following would make it more clear: "In this analysis, 450 gene family expansions and 6 contractions were observed in the C. heheva lineage, since the last common ancestor of A. japonicus and C. heheva (Fig. 3A)."<br><br>We apologize for the grammar mistakes of our manuscript. According to the reviewer's suggestion, we have re-written this sentence in the revised manuscript (lines 261-263). We have now worked on both language and readability and have also involved English speakers for language corrections. We really hope that the flow and language level have been substantially improved.
**Additional Information:**	
**Question**	**Response**
Are you submitting this manuscript to a special series or article collection?	No
**Experimental design and statistics**<br><br><br>Full details of the experimental design and statistical methods used should be given in the Methods section, as detailed in our Minimum Standards Reporting Checklist. Information essential to interpreting the	Yes

data presented should be made available in the figure legends.<br><br>Have you included all the information requested in your manuscript?	
**Resources**<br><br>A description of all resources used, including antibodies, cell lines, animals and software tools, with enough information to allow them to be uniquely identified, should be included in the Methods section. Authors are strongly encouraged to cite Research Resource Identifiers (RRIDs) for antibodies, model organisms and tools, where possible.<br><br>Have you included the information requested as detailed in our Minimum Standards Reporting Checklist?	Yes
**Availability of data and materials**<br><br>All datasets and code on which the conclusions of the paper rely must be either included in your submission or deposited in publicly available repositories (where available and ethically appropriate), referencing such data using a unique identifier in the references and in the "Availability of Data and Materials" section of your manuscript.<br><br>Have you have met the above requirement as detailed in our Minimum Standards Reporting Checklist?	Yes

Manuscript Click here to access/download;Manuscript;Manuscript_Nov 22_revision.docx

A high-quality chromosomal genome assembly of the sea cucumber *Chiridota heheva* and its hydrothermal adaptation

Yujin Pu, Yang Zhou, Jun Liu, Haibin Zhang*

[1] Institute of Deep-sea Science and Engineering, Chinese Academy of Sciences, Sanya 572000, China

[2] University of Chinese Academy of Sciences, Beijing 100049, China

*Correspondding address. Haibin Zhang, Institute of Deep-sea Science and Engineering, Chinese Academy of Sciences, Sanya 572000, China. E-mail: hzhang@idsse.ac.cn

Yujin Pu [0000-0002-8294-013X];

Yang Zhou [0000-0002-9129-9110];

Jun Liu [0000-0003-1925-752X];

Haibin Zhang [0000-0001-5429-9851]

Abstract

Background: *Chiridota heheva* is a cosmopolitan holothurian well adapted to diverse deep-sea ecosystems, especially chemosynthetic environments. Besides high hydrostatic pressure and limited light, high concentrations of metal ions also represent harsh conditions in hydrothermal environments. Few holothurian species can live in such extreme conditions. Therefore, it is valuable to elucidate the adaptive genetic mechanisms of *C. heheva* in hydrothermal environments.

Findings: Herein, we report a high-quality reference genome assembly of *C. heheva*

from the Kairei vent, which is the first chromosome-level genome of Apodida. The chromosome-level genome size was 1.43 Gb, with a scaffold N50 of 53.24 Mb and BUSCO completeness score of 94.5 %. Contig sequences were clustered, ordered, and assembled into 19 natural chromosomes. Comparative genome analysis found that the expanded gene families and positively selected genes of *C. heheva* were involved in the DNA damage repair process. The expanded gene families and the unique genes were contributed to maintaining iron homeostasis in an iron-enriched environment. The positively selected gene *RFC2* with ten positively selected sites played an essential role in DNA repair under extreme environments.

Conclusions: This first chromosome-level genome assembly of *C. heheva* reveals the hydrothermal adaptation of holothurians. As the first chromosome-level genome of Order Apodida, this genome will provide the resource for investigating the evolution of Class Holothuroidea.

Keywords: *Chiridota heheva,* Hi-C, positively selected gene, gene family, unique gene

Data Description

Context

Hydrothermal vents are one of the typical deep-sea chemosynthetically-driven ecosystems with a wide array of animals and chemosynthetic microbes. The hydrothermal vent environment is characterized by rapid changes in temperature, acidic pH, sulfur compounds, metal, methane, hydrogen, carbon dioxide, and other toxic chemistry, besides high hydrostatic pressure and darkness of the deep sea [1-9].

However, these inhospitable environments have been reported as crucial enrichment areas for deep-sea life.

Hydrothermal habitat fauna are commonly adapts to the unusual environment with the uncommon physical and chemical properties of vent fluids. Diverse fauna, including Annelida, Arthropoda, Mollusca, Echinodermata, Cnidaria, and Chordata have been described in hydrothermal vents; these vent faunas survive on their unique strategies in extreme conditions [9]. According to the previous studies of typical vent fauna, such as crab *Austinograea rodriguezensis* [10], *s*hrimps *Rimicaris kairei* [11], *Rimicaris* sp. [12], mussel *Gigantidas vrijenhoeki* [13], *Bathymodiolus* mussels [14], and scaly-food gastropods *Chrysomallon squamiferum* [11, 15-18], were evolved to enhance their tolerance of high temperature, metal ion enrichment, and sulfur-rich conditions. The adaptive mechanism is like a hard exoskeleton to endure the thermal stress [10], the ion binding enzymes or respiratory proteins for ion homeostasis and detoxification [11, 14-17]. Besides the unusual conditions of hydrothermal vents, the adaptations for high hydrostatic pressure and limited light are inevitable. Among various deep-sea fauna, DNA repair, degenerated ossicles, protein activity protection, and cell cycle maintenance have evolved to high hydrostatic pressure adaptation [5, 19-21]. The white body colour, unpigmented skin, scales, and long-wavelength light sensors of marine fauna were ubiquitous in the light-limited deep sea [6, 19-20, 22-23]. To gather knowledge about the genetic basis of adaptation to deep-sea extreme environments is particularly interesting.

Holothurians are widely distributed in several ecosystems' oceans, and more than

1,800 species have been accepted [24]. Few holothurian species can live in such extreme conditions of hydrothermal environments. *Chiridota heheva* (NCBI:txid2743191; marinespecies.org:taxname:242131), with the features of inhabiting in all biotopes of the deep-sea ocean [9, 21, 25-27]. The cold seep adaptations of *C. heheva* have been reported by Zhang et al. [21]. However, genome information on *C. heheva* in hydrothermal vent is currently unavailable. In the present study, we obtained the genome of *C. heheva* with the sample collected in the Kairei vent. Kairei vent is an ultramafic-hosted system that was discovered in the Indian Ocean. Kairei fluids are highly enriched in dissolved Fe (5,400 μM) that leach from the host rock [9, 15, 28-29]. We obtained a chromosome-level genome of *C. heheva* by Hi-C technology with an integrated comprehensive gene set. Moreover, comparative genomic analyses were performed to investigate the hydrothermal vent adaptive mechanisms of *C. heheva*. Finally, together with other published genomic data from vent animals, these assembly results can add more information which will help to gain insights into the adaptation of the whole vent fauna.

Methods

Sampling and sequencing

The *C. heheva* individual used for genomic sequencing was collected by the maned submersible vehicle '*Shenhaiyongshi*' from the Kairei vent field in the Mid-Indian Ocean (70.40°E, 25.32°S), with a depth of 2,428 m, on 7 February 2019 (Fig. 1). The sample was dissected and frozen in liquid nitrogen, then send to the Institute of Deep-

sea Science and Engineering, Chinese Academy of Science, Sanya, China, and subsequent storage at -80℃ for further analysis.

The high-molecular-weight genomic DNA (gDNA) was prepared manually from body-wall tissue following a modified protocol described previously [30]. Briefly, tissue was ground with liquid nitrogen freezing and digested at 65℃ in SDS (sodium dodecyl sulfate) buffer [50 mM Tris-HCl, 50 mM EDTA, 3% SDS (w/v)] for 1 h. Then the lysate was treated by Phenol/Chloroform isolated and Isopropanol precipitation. The gDNA was assessed and sheared to ~15 kb fragment length for Pacific Biosciences (PacBio) HiFi sequencing. The HiFi SMRTbell library was constructed with SMRTbell Express Template Prep Kit 2.0 (Pacific Biosciences, California, USA), and the HiFi reads were sequenced using one cell on SMRT cells 8M on a PacBio Sequel II platform (PacBio Sequel II System, RRID:SCR_017990). For genome annotation, the total RNA was isolated from gonad and body-wall tissues using an RNeasy Plus Universal Kit (QIAGEN, Hilden, Germany). The total RNA was used to obtain cDNA by reverse transcribing, and then 150 bp paired-end reads were generated on the Illumina NovaSeq 6000 platform (Illumina NovaSeq 6000 Sequencing System, RRID:SCR_016387). Novogene Company, Tianjin, China conducted the sequencing processes above.

Hi-C library preparation and sequencing from body-wall tissue have been done following the standard protocol described previously [31]. Briefly, crosslinking the grounded body-wall tissue with 4% formaldehyde, digesting the DNA with restriction enzyme MboI (GATC), making the DNA ends with biotin-14-dCTP, ligating the blunt-end fragments, shearing the DNA into 200- to 600 bp fragments by sonication. Finally,

the Hi-C sequencing library was constructed and conducted on the Illumina NovaSeq-6000 sequencing platform (PE 150bp). Novogene Company, Tianjin, China, performed the experiments and sequencing.

Genome assembly and annotation

Hifiasm version 0.16.1-r375 (Hifiasm, RRID:SCR_021069) with default parameters setting was used for PacBio HiFi reads assembly [32]. Purge_dups version 1.2.5 (purge dups, RRID:SCR_021173) was used for redundancy purge of the primary genome and obtained the clean genome without the duplicate contigs [33]. Juicer version 1.6 (Juicer, RRID:SCR_017226) was used to analyze Hi-C reads combined with contig-level genome [34]. 3D-DNA version 190716 (3D de novo assembly, RRID:SCR_017227) was used to primarily correct misjoin, order and orient, in the scaffold and obtain the potential chromosomal groups [35]. Juicebox version 1.11.08 was then used to manually order the scaffolds of the result from 3D-DNA [36]. The tool 3D-DNA was used again to obtain the final chromosome assembly for further analysis [35]. The completeness of the chromosome-level genome was assessed using BUSCO version 5.4.6 (BUSCO, RRID:SCR_015008) with the metazoa_odb10 lineage data set (954 orthologs) [37]. The assembly quality was also evaluated using Inspector version 1.0.1, which only relied on third-generation sequencing reads [38].

RepeatModeler version 2.0.1 (RepeatModeler, RRID:SCR_015027) [39] and RepeatMasker version open-4.0.6 (RepeatMasker, RRID:SCR_012954) [40] were used for searching repetitive elements in the final genome assembly and generated a soft-

masked genome with non-redundant data set of repetitive elements. Subsequently, gene structure annotation in the soft-masked genome was predicted by *ab initio* and evidence-based gene prediction. Augustus version 3.4.3 (Augustus, RRID:SCR_008417) [41], GlimmerHMM version 3.0.4 (GlimmerHMM, RRID:SCR 002654) [42], and GeneID version 1.4.5 (Entrez Gene, RRID:SCR_002473) [43] were used in *ab initio* gene prediction. Moreover, Exonerate version 2.2.0 (Exonerate, RRID: SCR_016088) was employed for protein homologous annotation in evidence-based gene prediction [44]. PASA version 2.5.2 (PASA, RRID:SCR_014656) was applied for transcriptomic annotation in evidence-based gene prediction [45]. EVidenceModeler version 1.1.1 (EVidenceModeler, RRID:SCR_014659) produced a weighed consensus protein set by combining the results from *ab initio* gene models and evidence-based gene models [46]. The protein set was used for gene functional annotation as follows. DIAMOND BLASTP version 2.0.14 was used to search protein function in the nr database of NCBI [47], Interproscan version 5 (InterProScan, RRID:SCR_005829) was employed to predict the protein family membership, functional domains and sites in Swiss-Prot, Pfam [48], and KAAS (KEGG Automatic Annotation Server) was applied for KEGG pathways annotated online [49].

Orthology prediction and phylogenomic analysis

Protein sets of night echinoderm species (*Anneissia japonica*, *Acanthaster planci*, *Asterias rubens*, *Plazaster borealis*, *Ophiothrix spiculata*, *Strongylocentrotus purpuratus*, *Lytechinus variegatus*, *Apostichopus japonicus*, and *C. heheva*) were

employed in the orthology identification with *Homo sapiens* as the outgroup (Supplementary Table S1). OrthoFinder version 2.5.4 (OrthoFinder, RRID:SCR_017118) was applied to determine and cluster gene families among these ten metazoan species [50]. A total of 495 single-copy orthologs among these species were multiple aligned with MAFFT version 7.475 (MAFFT, RRID:SCR_011811) [51], then concatenated and used for constructing a phylogenomic tree using RAxML version 8.2.3 (RAxML, RRID:SCR_006086) [52] based on the substitution model of GTRGAMMA with 100 bootstraps. The divergence time among these species was estimated using MCMCTREE in PAML version 4.9 (PAML, RRID:SCR_014932) [53]. Based on the TimeTree database (TimeTree, RRID:SCR_021162) [54], Deuterostomia (515.5 - 636.1 Mya), Echinodermata (509.0 - 549.0 Mya), and Eleutherozoa (480.0 - 488.0 Mya) were applied for the calibration time.

Genome synteny analysis

Chromosome-level genome in our study of *C. heheva* (CHEH_vent1.0) and *A. japonicus* (AJH1.0) [55] was selected as comparisons for syntenic analysis. BLAST version 2.9.0 (BLAST Similarity Search, RRID:SCR_008419) with parameter "-evalue 1e-10" was used to identify similar gene pairs [56]. JCVI version 0.18 (RRID:SCR_021641) was used to perform protein sequence alignment between CHEH_vent1.0 and AJH1.0 and filter the BLAST results with parameter "--cscore =0.5", then search for syntenic blocks in all the genes (jcvi, RRID:SCR_021641) [57]. Subsequently, JCVI was also used to visualize the syntenic results with the graphic

command.

Gene family analysis

Based on orthologous gene families and phylogenetic relationships above, CAFE version 4.2.1 (CAFE, RRID: SCR_005983) [58] was used to detect the gene family expansion and contraction. GO enrichment and KEGG pathway enrichment were performed online [59] and were used to investigate the functional properties of the expansion gene families. A conditional P value was calculated for each gene family, and a significantly accelerated rate of expansion families was left while P-values were lower than 0.05.

Genes under positive selection

As the number of single-copy orthologous genes from OrthoFinder is limited, orthologs were identified as reciprocal best blast hits using the RBH Ortholog pipeline [60]. A total of 3,269 orthologs identified above were used for tests for positive selection. MAFFT version 7.475 (MAFFT, RRID:SCR_011811) [51] was used for multiple alignments, and the alignments of the corresponding DNA codon sequences were further trimmed by trimAl version 1.4.1 (trimAl, RRID:SCR_017334) [61]. Positively selected genes and amino acid sites were assessed with the branch model and branch-site model using codeml in PAML package version 4.9 (PAML, RRID:SCR_014932) [53]. A likelihood ratio test was conducted, and the false discovery rate (FDR) correction was performed for multiple comparisons. Genes and

sites with a corrected FDR <0.05 were defined as putatively evolving under positive selection.

Unique genes

Protein sets of six echinoderms (*A. japonica*, *A. planci*, *O. spiculata*, *S. purpuratus*, the Haima cold seep *C. heheva*, and the Kairei vent *C. heheva*) were employed for orthologous clusters analysis. OrthoVenn3 [62] was used to identify clusters based on OrthoFinder algorithm among the six echinoderms. The cluster results were visualized by UpSet (RRID:SCR_022731) [63], which could support the overlapped clusters among diverse species and provide the unique clusters among each species. Based on the unique clusters of the Kairei vent *C. heheva*, the unique genes were collected from the unique clusters and were used for GO enrichment. The UpSet and GO enrichment analyses were automatically run on the OrthoVenn3 platform.

Results

Chromosome-scale genome assembly and completeness evaluation

The CCS HiFi reads with 29.25 Gb were sequenced on the PacBio Sequel II platform (Supplementary Table S2). In order to create continuity in the genome assembly, 159.59 Gb of Hi-C reads were further prepared on the Illumina NovaSeq 6000 sequencing platform (~ 111 × genome coverage) (Supplementary Table S2). RNA reads with 13.29 Gb were generated on the Illumina NovaSeq 6000 sequencing platform utilized for genome annotation (Supplementary Table S2).

Our chromosome-level genome assembly of *C. heheva* (CHEH_vent1.0) was performed using both HiFi reads and Hi-C reads. The total size of the final assembly was 1.43 Gb with an N50 of 53.24 Mb, consisting of 19 chromosome-level scaffolds with lengths ranging from 30 to 115 Mb (Fig. 2, Table 1). The genome size of this species in the Haima cold seep is 1.107 Gb [21], about three-quarters of the genome size in the Kairei vent. BUSCO [37] with a database of metazoan_odb10 was used to evaluate the completeness of genome, the BUSCO score is higher in the Kairei vent *C. heheva* (94.50%) when compared with the Haima cold seed *C. heheva* (89.60%) [21]. As the output of Inspector, a high mapping rate (99.76%) suggests better completeness of the assembly, the similarity of alignment depth (20.3745) and sequencing depth (20.3857) indicates a good assembly, a low error rate (E, 0.0011), which calculated as QV= $-10\log_{10}E$ based on quality value (QV, 29.7695), revealed high accuracy of the assembly [38].

Annotation of repetitive elements and protein-coding genes

Repetitive element annotation identified that in 70.80% (1.02 Gb) of the whole genome assembly, the long interspersed nuclear elements (LINEs) were the largest class of the transposable elements (TEs) annotated; other predominant repetitive elements are summarized in Table 2. Compared with other echinoderms (Supplemental Table S3), the repetitive genes percentage of *C. heheva* (Kairei vent) in this study is more than that of 56.64% in Haima Cold Seep [21] and only less than *Paelopatides sp.* Yap is 73.93% [23], and the percentage of shallow water *A. japonicus* only has 27.20% [55, 64]. After

repeat masking, protein-coding genes annotated were using a combination of *ab initio*, homology-based, and transcript-evidence predicted approaches, and a total of 32,434 were successfully identified (Table 3). Interproscan, KEGG, NR, and UniProt were employed for functional annotations, and 24,606 genes were mapped to at least one database (Table 3).

Phylogenetic and syntenic relationship

In order to investigate the phylogenetic relationship between *C. heheva* and other metazoans, nine species were selected for the phylogenomic tree reconstruction (Supplementary Table S1). A total of 495 single-copy genes in all species with high completeness genomes were used to construct a phylogenomic tree (Fig. 3A, B; Supplemental Fig. S1). *C. heheva* and *A. japonicus* appeared as a sister clade in holothurians, and diverged from other echinoderms approximately 438.1 Mya and the divergence time of holothurians in this study supported the view that holothurians had evolved by the Ordovician [65-68].

The syntenic blocks were detected between *C. heheva* and *A. japonicus* using JCVI [57] and shown as a dot plot (Fig. 3C). The results showed most of the chromosomes of *C. heheva* were highly conserved with *A. japonicus*, except for chromosomes 1, 3, 4, and 5 of *C. heheva*. The results also indicated that events of chromosomal fissions and fusions have occurred during the evolutionary history of Holothuroidea, resulting in the variable numbers of chromosomes between *C. heheva* and *A. japonicus*. Based on the chromosomal fissions and fusions, chromosome 1 of *C. heheva* corresponded to

chromosomes 4 and 12 of *A. japonicus*. In contrast, while chromosome 3 corresponded to chromosomes 10 and 21, chromosome 4 corresponded to chromosomes 7 and 17, and chromosome 5 corresponded to chromosomes 3 and 23, respectively. The findings of high identity between the 19 chromosomes of *C. heheva* and 23 chromosomes of *A. japonicus* suggested that they share similar gene sets of their origins despite the chromosomal fissions or fusions.

Gene family evolution

Based on the phylogenomic tree (Fig. 3A), gene family analysis was performed using CAFE [58]. In this analysis, 450 gene family expansions and six contractions were observed in the *C. heheva* lineage, since the last common ancestor of *A. japonicus* and *C. heheva* (Fig. 3A). Collectively, these expanded gene families of *C. heheva* were mainly enriched in membrane functions, nucleoside processes of DNA repair, and proteins activity (Fig. 4; Supplementary Table S4). Membrane-associated processes have been described that were particularly susceptible to perturbation under conditions of high hydrostatic pressure, including reducing the fluidity of lipid bilayers and denaturing membrane-associated proteins [19, 69-70]. Biological membranes mainly composed of phospholipids, sterols (generally cholesterol), glycolipids, and proteins [70-71]. Phospholipids, with hydrophilic the phosphate group head and hydrophobic the fatty acid tails, constitute a significant component that form the lipid bilayers [71]. Changes in lipid composition modulate membrane fluidity, especially the proportion of unsaturated fatty acids [19, 23, 70-72]. As our results, lipid metabolisms were activated

to response to high hydrostatic pressure, including essential fatty acid of arachidonic acid (AA) metabolism, linoleic acid (LA) metabolism, alpha-linolenic acid (ALA) metabolism, etc. (Fig. 4; Supplementary Table S4). AA, LA, and ALA are polyunsaturated fatty acids (PUFAs) metabolized into various PUFAs during stress (Supplementary Figure S2). Phospholipid-bound AA is the substrate for the synthesis of a range of biologically active compounds, including prostaglandins (PGs), thromboxanes (TXs), and leukotrienes (LTs), epoxyeicosatrienoic acids (EETs), and hydroxyeicosatetraenoic acids (HETEs) [73]. LA is important in the biosynthesis of AA, while ALA is the precursor of EPA and DHA and then converts into EPA and DHA through metabolism [72]. Some of the PUFAs may bind to receptors on the membrane of cells and relate to membrane fluidity adaptation to compensate for environmental changes, especially AA and DHA, which have been reported in previous studies [19, 23, 72-73]. Iron is crucial in living organisms and intimately involved in numerous biological processes [74-75]. Iron in organisms is mainly bound to heme (heme-iron), transported by transferrin (TF-bound iron), and stored in ferritin (FT-stored iron) for biological functions, which are redox-inert iron in nontoxic forms [74]. Nontransferrin-bound iron (NTBI) and other free iron released from iron-bound proteins are potentially toxic because excess redox-active iron induces oxidative stress and causes cell damage [74-75]. Deep-sea fauna explored in iron-rich hydrothermal vents have several mechanisms for maintaining iron homeostasis [9, 14, 16-17]. Cytochrome P450 (CYP) was reported to play an essential role in the regulation of iron levels to maintain cellular redox homeostasis and against oxidative stress [75]. P450 and transferrin are the

expanded gene families, which are enriched in iron ion binding and metal ion transport respectively, suggesting the adapting to the iron-rich environment of the Kairei vent. High hydrostatic pressure may damage DNA in deep-sea fauna susceptibly [20]. The response of DNA repair was variously described in previous studies, including DNA damage detection, replication, recombination, splicing, excision, endonuclease, and so on [5, 20, 23, 76]. The expanded gene families enriched in DNA replication, recombination, DNA-associated protein, and nucleic acid binding suggest a capability of DNA repair. Hydrostatic pressure inhibits protein functions by affecting folding and enzyme activity [19, 21]. These expanded gene families clustered in protein synthesis and activities of various enzymes may contribute to ensuring the functions of the protein.

Positively selected genes

The positively selected genes support the genetic basis for environmental adaptation. Compared with the other nine metazoans, 28 positively selected genes were identified in the *C. heheva* (Table 4). According to the GO enrichment analysis, positively selected genes were mainly enriched for various processes including cyclic compound binding, ion binding, nucleotide binding, ATP binding, DNA binding, DNA repair, and stress response (Supplementary Table S5). Based on the KEGG pathway enrichment, positively selected genes were mainly involved in Fanconi anemia pathway, an essential component of the DNA damage response and DNA repair [77]. Some genes of deep-sea fauna were positively selected during the adaption to the environment. DNA repair genes have been selected for deep-sea adaptation and may play an important role

in maintaining the fidelity of genetic materials in deep-sea environments [5, 20, 23, 76]. Among these processes, at least 11 positively selected genes (*POLB*, *FAN1*, *RFC2*, *KDM2A*, *FARSA*, *SPG7*, *BRCA1*, *TTLL9*, *DCLK1*, *LDHD*, and *SIRT4*) involved in DNA repair (Table 4; Supplementary Table S5). Therein, DNA repair gene *BRCA1* had been found to protect DNA from high pressure in hadal *Paelopatides* sp. Yap [23]. Furthermore, gene *RFC2*, which functions as DNA replication, nucleotide excision repair, mismatch repair, DNA repair and recombination proteins, has ten positively selected sites (Fig. 5A, B). These ten positively selected sites may enhance the DNA repair abilities of *RFC2* and reveal potential pathways for enhancing the high hydrostatic pressure tolerance. These results indicated that DNA damage repair was mainly reflected in the positively selected genes of the *C. heheva* genome.

Unique genes evolution

In order to investigate the environment-specific adaptation of the Kairei vent *C. heheva*, we identified the unique clusters based on the protein sets of six echinoderms. As results of UpSet show that, 14,032 clusters were identified in the Kairei vent *C. heheva* and 5,132 clusters were shared with the other five echinoderms. In contrast, 933 clusters were identified only in the Kairei vent *C. heheva* and were considered as unique clusters (Fig. 6). As the results of GO enrichment analysis, the unique genes of the Kairei vent *C. heheva* were significantly enriched in the category of ion binding and transport, including sodium ion transport, ion transport, sodium-independent organic anion transport, metal ion binding, and zinc ion binding (Fig. 7; Supplementary Table

S6). Among the ion-relative terms, metal ion binding (GO:0046872) exerts an iron ion binding function through CYP2B14P (Cytochrome P450, family 2, subfamily b, polypeptide 14), and CYP 450 has reported that acted as a function of iron homeostasis maintaining [75]. The metal ion binding enriched term of the unique genes of the Kairei vent *C. heheva* suggested environment-specific adaptation, due to the Kairei vent being rich in iron [15, 17, 29].

Conclusions

The first chromosome-level genome, *C. heheva* living in a hydrothermal vent, was assembled and annotated. A total of 19 chromosomal scaffolds are constructed with N50 of 53.24 Mb. The BUSCO score of 94.5% confirmed the completeness of the genome. Comparative genome analysis results indicated that several positively selected and expanded genes were involved in the DNA repair. Furthermore, the expanded genes and the unique genes contributed to iron ion binding for maintaining iron homeostasis in iron-rich environment adaptation. This data set will provide a valuable resource for further studies on hydrothermal vent adaptations of vent fauna.

Data Availability

The final genome assembly and other associated raw data described in this study are available on ScienceDB [78]. The raw sequencing reads were also deposited at NCBI under BioProject PRJNA934972. All additional supporting data are available in the *GigaScience* repository, GigaDB [79].

Abbreviations

Gb: Gigabase pairs; Mb: Megabase pairs; BUSCO: Benchmarking Universal Single-Copy Orthologs; PE: Paired End; HiFi: High-Fidelity; Hi-C: High-Throughput Chromosome Conformation Capture; BLAST: Basic Local Alignment Search Tool; NCBI: National Center for Biotechnology Information; KASS: KEGG Automatic Annotation Server; KEGG: Kyoto Encyclopedia of Genes and Genomes; GO: Gene Ontology; NR: NCBI's nonredundant database; RBH: Reciprocal Best Hit; CCS: Circular Consensus Sequencing; bp: Base Pairs; GC: Guanine Cytosine; LINE: Long Interspersed Nuclear Element; LTR: Long Terminal Repeat; SINE: Short Interspersed Nuclear Elements; FDR: False Discovery Rate.

Additional Files

Supplementary Figure S1. BUSCO completeness assessment of gene sets from ten genomes in phylogenomic analysis.

Supplementary Figure S2. Essential fatty acids metabolism in brief.

Supplementary Table S1. Data sets used for the ten species in phylogenomic analysis.

Supplementary Table S2. Statistics of raw sequencing data.

Supplementary Table S3. Statistics of repetitive elements percentage in related echinoderms.

Supplementary Table S4. GO and KEGG enrichment of expanded gene families of the Kairei vent *Chiridota heheva*.

Supplementary Table S5. GO and KEGG enrichment of positively selected genes of the

Kairei vent *Chiridota heheva*.

Supplementary Table S6. GO enrichment of unique genes of the Kairei vent *Chiridota heheva*.

Supplementary Table S7. Reagent and software.

Supplementary text S1. Commands for analyses.

Competing Interests

The authors declare that they have no competing interests.

Funding

This study was financially supported by the major scientific and technological projects of Hainan Province (ZDKJ2019011), Strategic Priority Research Program of the Chinese Academy of Sciences (CAS) (XDA22050303), National Key Research and Development Program of China (2016YFC0304905).

Authors’ Contributions

Haibin Zhang led the project. Haibin Zhang and Yujin Pu conceived this study. Yujin Pu performed the experiments by Jun Liu assistance. Yujin Pu assembled the genome and analyzed the genomic data with assistance by Yang Zhou. Yujin Pu wrote the first draft of the manuscript. All authors reviewed the manuscript.

Acknowledgments

We thank the captains, crews, and scientific staff on the R/V *Tansuo 1*, and the pilots of HOV *Shenhaiyongshi* for their support in sample collection. We also thank Dr. El-Hadji Malick Cisse (Beltsville Agricultural Research Center, Agricultural Research Service (USDA), Beltsville, United States) for improving the language and grammar of this manuscript. Special thanks to the reviewers for their helpful comments and constructive suggestions on the manuscript.

References

1. Miyazaki JI, Beppu S, Kajio S, et al. Dispersal ability and environmental adaptability of deep-sea mussels *Bathymodiolus* (Mytilidae: Bathymodiolinae). Open Journal of Marine Science 2013; 3(1):31-39.
2. Brazelton W. Hydrothermal vents. Curr Biol 2017; 27(11): 450-452.
3. Sun J, Zhang Y, Xu T, et al. Adaptation to deep-sea chemosynthetic environments as revealed by mussel genomes. Nat Ecol Evol 2017; 1(5): 1-7.
4. Wang ZF, Shi XJ, Sun LX, et al. Evolution of mitochondrial energy metabolism genes associated with hydrothermal vent adaption of Alvinocaridid shrimps. Genes Genom 2017; 39(12): 1367-1376.
5. Cheng J, Hui M, Sha ZL. Transcriptomic analysis reveals insights into deep-sea adaptations of the dominant species, *Shinkaia crosnieri* (Crustacea: Decapoda: Anomura), inhabiting both hydrothermal vents and cold seeps. BMC Genomics 2019; 20(1): 1-16.
6. Lutz RA. Hydrothermal vent fauna. Reference module in Encyclopedia of Ocean Sciences (Third Edition), 2019; 2: 715-727.
7. Snelgrove PVR, Grassle JF. Deep-sea fauna. Reference module in Encyclopedia of Ocean Sciences (Third Edition), 2019; 2: 706-714.
8. Methou P, Hikosaka M, Chen C, et al. Symbiont community composition in *Rimicaris kairei* shrimps from Indian Ocean vents with notes on Mineralogy. Appl Environ Microb 2022; 88(8): e00185-22.
9. Prakash LS, Fernandes SO, Ingole B,et al. Biogeochemical Characteristics of

Hydrothermal Systems in the Indian Ocean. Systems Biogeochemistry of Major Marine Biomes 2022; 285-313.

10. Cho B, Kim D, Bae H, et al. Unique characteristics of the exoskeleton of bythograeid crab, *Austinograea rodriguezensis* in the Indian Ocean hydrothermal vent (Onnuri vent field). Integr Comp Biol 2020; 60(1): 24-32.

11. Nakamura K, Watanabe H, Miyazaki J, et al. Discovery of new hydrothermal activity and chemosynthetic fauna on the Central Indian Ridge at 18-20 °S. PLoS one 2012; 7(3): e32965.

12. Zhang J, Sun QL, Luan ZD, et al. Comparative transcriptome analysis of *Rimicaris* sp. reveals novel molecular features associated with survival in deep-sea hydrothermal vent. Sci Rep-UK 2017; 7(1): 1-16.

13. Jang SJ, Ho PT, Jun SY, et al. A newly discovered Gigantidas bivalve mussel from the Onnuri Vent Field in the northern Central Indian Ridge. Deep-sea Res Pt I: Oceanographic Research Papers 2020; 161: 103299.

14. Zhou L, Cao L, Wang XC, et al. Metal adaptation strategies of deep-sea Bathymodiolus mussels from a cold seep and three hydrothermal vents in the West Pacific. Sci Total Environ 2020; 707:136046.

15. Warén A, Bengtson S, Goffredi SK, et al. A hot-vent gastropod with iron sulfide dermal sclerites. Science 2003; 302: 1007-1007.

16. Okada S, Chen C, Watsuji T, et al. The making of natural iron sulfide nanoparticles in a hot vent snail. PNAS 2019; 116(41): 20376-20381.

17. Sun J, Chen C, Miyamoto N, et al. The Scaly-foot snail genome and implications

for the origins of biomineralised armour. Nat Commun 2020a; 11(1): 1-12.
18. Sun J, Zhou YD, Chen C, et al. Nearest vent, dearest friend: biodiversity of Tiancheng vent field reveals cross-ridge similarities in the Indian Ocean. Roy Soc open Sci 2020b; 7(3): 200110.
19. Wang K, Shen YJ, Yang YZ, et al. Morphology and genome of a snailfish from the Mariana Trench provide insights into deep-sea adaptation. Nat Ecol Evol 2019; 3(5): 823-833.
20. Liu RY, Liu J, Zhang HB. Positive selection analysis reveals the deep-sea adaptation of a hadal sea cucumber (*Paelopatides* sp.) to the Mariana Trench. J Oceanol Limnol 2021; 39(1), 266-281.
21. Zhang L, He J, Tan PP, et al. The genome of an apodid holothuroid (*Chiridota heheva*) provides insights into its adaptation to a deep-sea reducing environment. Commun Biol 2022; 5: 224.
22. Yuan JB, Zhang XJ, Gao Y, et al. Adaptation and molecular evidence for convergence in decapod crustaceans from deep- sea hydrothermal vent environments. Mol Ecol 2020; 29(20): 3954-3969.
23. Shao G, He T, Mu Y, et al. The genome of a hadal sea cucumber reveals novel adaptive strategies to deep-sea environments. Iscience 2022; 105545.
24. WoRMS. https://www.marinespecies.org/aphia.php?p=taxdetails&id=123083. Accessed 11 October 2022.
25. Thomas EA, Liu RY, Amon D, et al. *Chiridota heheva*-the cosmopolitan holothurian. Mar Biodivers 2020; 50(6): 1-13.

26. Sun SE, Sha ZL, Xiao N. The first two complete mitogenomes of the order Apodida from deep-sea chemoautotrophic environments: New insights into the gene rearrangement, origin and evolution of the deep-sea sea cucumbers. Comparative Biochemistry and Physiology Part D: Genomics and Proteomics, 2021; 39: 100839.

27. Zhou Y, Zhang DS, Zhang RY, et al. Characterization of vent fauna at three hydrothermal vent fields on the Southwest Indian Ridge: Implications for biogeography and interannual dynamics on ultraslow-spreading ridges. Deep Sea Research Part I: Oceanographic Research Papers 2018; 137: 1-12.

28. Humphris SE, Fornari DJ. Hydrothermal vents in an unusual geotectonic setting: the Kairei and Edmond vent fields, Central Indian Ridge[C]//AGU Fall Meeting Abstracts. 2001, 2001: OS41A-0444.

29. Wang YJ, Han XQ, Petersen S, et al. Trace metal distribution in sulfide minerals from Ultramafic-Hosted hydrothermal systems: examples from the Kairei vent field, central indian ridge. Minerals 2018; 8(11):526.

30. Xia YM, Chen FS, Du Y, et al. A modified SDS-based DNA extraction method from raw soybean. Bioscience Rep 2019; 39(2): BSR20182271.

31. Belton J-M, McCord RP, Gibcus JH, et al. Hi-C: A comprehensive technique to capture the conformation of genomes. Methods 2012; 58(3): 268-276.

32. Cheng H, Concepcion GT, Feng X, et al. Haplotype-resolved de novo assembly using phased assembly graphs with hifiasm. Nat Methods 2021; 18: 170-175.

33. Guan DF, McCarthy SA, Wood J, et al. Identifying and removing haplotypic

duplication in primary genome assemblies. Bioinformatics 2020; 36(9): 2896-2898.

34. Durand NC, Shamim MS, Machol I, et al. Juicer provides a one-click system for analyzing loop-resolution Hi-C experiments. Cell Syst 2016; 3(1): 95-98.

35. Dudchenko O, Batra SS, Omer AD, et al. De novo assembly of the Aedes aegypti genome using Hi-C yields chromosome-length scaffolds . Science 2017; 356(6333): 92-95.

36. Durand NC, Robinson JT, Shamim MS, et al. Juicebox Provides a Visualization System for Hi-C Contact Maps with Unlimited Zoom. Cell Syst 2016; 3(1): 99-101.

37. Manni M, Berkeley MR, Seppey M, et al. BUSCO update: Novel and streamlined workflows along with broader and deeper phylogenetic coverage for scoring of eukaryotic, prokaryotic, and viral genomes. Mol Biol Evol 2021; 38(10): 4647-4654.

38. Chen Y, Zhang YX, Wang AY, et al. Accurate long-read de novo assembly evaluation with Inspector. Genome Biol 2021; 22:312.

39. Flynn JM, Hubley R, Goubert C, et al. RepeatModeler2 for automated genomic discovery of transposable element families. PNAS 2020; 117(17): 9451-9457.

40. Tarailo-Graovac M, Chen N. Using RepeatMasker to identify repetitive elements in genomic sequences. Curr Protoc Bioinform. 2009; 25(1): 4.10.1-4.10.14

41. Stanke M, Diekhans M, Baertsch R, et al. Using native and syntenically mapped cDNA alignments to improve de novo gene finding. Bioinformatics 2008; 24(5):

637-644.

42. Majoros WH, Pertea M, Salzberg SL. TigrScan and GlimmerHMM: two open source ab initio eukaryotic gene-finders. Bioinformatics 2004; 20(16): 2878-2879.

43. Alioto T, Blanco E, Parra G, et al. Using geneid to Identify Genes. Current Protocols in Bioinformatics 2018; e56.

44. Slater GS, Birney E. Automated generation of heuristics for biological sequence comparison. BMC Bioinformatics 2005; 6:31.

45. Haas BJ, Zeng Q, Pearson MD, et al. Approaches to Fungal Genome Annotation. Mycology 2011; 2(3):118-141.

46. Haas BJ, Salzberg SL, Zhu W, et al. Automated eukaryotic gene structure annotation using EVidenceModeler and the Program to Assemble Spliced Alignments. Genome Biol 2008; 9(1): R7.

47. Buchfink B, Reuter K, Drost H-G. Sensitive protein alignments at tree-of-life scale using DIAMOND. Nat Methods 2021;18(4):366-8.

48. Jones P, Binns D, Chang H-Y, et al. InterProScan 5: genome-scale protein function classification. Bioinformatics 2014; 30(9): 123-1240.

49. KAAS - KEGG automatic annotation server. https://www.genome.jp/tools/kaas/.

50. Emms DM, Kelly S. OrthoFinder: phylogenetic orthology inference for comparative genomics. Genome Biol 2019; 20(1).

51. Katoh K, Standley DM. MAFFT Multiple sequence alignment software version 7: Improvements in performance and usability. Mol Biol Evol 2013; 30(4): 772-780.

52. Stamatakis A. RAxML version 8: a tool for phylogenetic analysis and post-analysis

of large phylogenies. Bioinformatics 2014; 30(9): 1312-1313.

53. Yang Z. PAML 4: Phylogenetic analysis by maximum likelihood. Mol Biol and Evol 2007; 24(8): 1586-1591.

54. Timetree of life. http://www.timetree.org/.

55. Wang YX, Yang YJ, Li YL, et al. Identification of sex determination locus in sea cucumber *Apostichopus japonicus* using genome-wide association study. BMC Genomics 2022; 23: 391.

56. Camacho C, Coulouris G, Avagyan VM, et al. BLAST+: architecture and applications. BMC Bioinformatics 2009; 10:421.

57. Tang HB, Krishnakumar V, Li J. jcvi: JCVI utility libraries. Zenodo 2015.

58. De Bie T, Cristianini N, Demuth JP, et al. CAFE: a computational tool for the study of gene family evolution. Bioinformatics 2006; 22(10): 1269-1271.

59. Genescloud. https://www.genescloud.cn/.

60. Barker MS, Dlugosch KM, Dinh L, et al. EvoPipes.net: Bioinformatic tools for ecological and evolutionary genomics. Evol Bioinform 2010; 6.

61. Capella-Gutiérrez S, Silla-Martínez JM, Gabaldón T. trimAl: a tool for automated alignment trimming in large-scale phylogenetic analyses. Bioinformatics 2009; 25(15):1972-3.

62. Sun JH, Lu F, Luo YJ, et al. OrthoVenn3: an integrated platform for exploring and visualizing orthologous data across genomes, Nucleic Acids Research 2023; gkad313.

63. Lex A, Gehlenborg N, Strobelt H.et al. UpSet: visualization of intersecting sets. IEEE Trans. Vis. Comput. Graph. 2014; 20:1983-1992.

64. Jo J, Oh J, Lee HG, et al. Draft genome of the sea cucumber *Apostichopus japonicus* and genetic polymorphism among color variants. Gigascience 2017; 6(1): giw006.

65. Reich M. Different pathways in early evolution of the holothurian calcareous ring. Progress in Echinoderm Palaeobiology 2015; 19: 137-145.

66. Reich M. The early evolution and diversification of holothurians (Echinozoa). Echinoderms: Durham: Taylor and Francis Group, London, 2010a: 55-59.

67. Reich M. The oldest synallactid sea cucumber (Echinodermata: Holothuroidea: Aspidochirotida). Paläontologische Zeitschrift 2010b; 84(4): 541-546.

68. Pierrat J, Bédier A, Eeckhaut I, et al. Sophistication in a seemingly simple creature: a review of wild holothurian nutrition in marine ecosystems. Biol Rev 2022; 97(1): 273-298.

69. Siebenaller JF, Garrett DJ. The effects of the deep-sea environment on transmembrane signaling. Comparative Biochemistry and Physiology Part B: Biochemistry and Molecular Biology 2002; 131(4): 675-694.

70. Montagne K, Uchiyama H, Furukawa KS, et al. Hydrostatic pressure decreases membrane fluidity and lipid desaturase expression in chondrocyte progenitor cells. J Biomech 2014; 47(2): 354-359.

71. Tamby A, Sinninghe Damsté JS, Villanueva L. Microbial membrane lipid adaptations to high hydrostatic pressure in the marine environment. Front Mol Biosci 2023; 9:1058381

72. Shrestha N, Holland OJ, Kent NL, et al. Maternal high linoleic acid alters placental fatty acid composition. Nutrients 2020; 12(8): 2183.

73. Wang B, Wu LJ, Chen J, et al. Metabolism pathways of arachidonic acids: mechanisms and potential therapeutic targets. Sig Transduct Target Ther 2021; 6: 94.

74. Ravingerová T, Kindernay L, Barteková M, et al. The molecular mechanisms of iron metabolism and its role in cardiac dysfunction and cardioprotection. Int. J. Mol. Sci. 2020; 21: 7889.

75. Song YS, Annalora AJ, Marcus CB, et al. Cytochrome P450 1B1: a key regulator of ocular iron homeostasis and oxidative stress. Cells 2022; 11: 2930.

76. Gan ZB, Yuan JB, Liu XM, et al. Comparative transcriptomic analysis of deep-and shallow-water barnacle species (Cirripedia, Poecilasmatidae) provides insights into deep-sea adaptation of sessile crustaceans. BMC genomics 2020; 21(1): 1-13.

77. Walden H, Deans AJ. The Fanconi anemia DNA repair pathway: structural and functional insights into a complex disorder. Annu Rev Biophys 2014; 43:257-278.

78. Pu YJ, 张海滨. A dataset of Chiridota heheva [DS/OL]. V1. Science Data Bank, 2023[2023-11-22]. https://doi.org/10.57760/sciencedb.07077.

79. Pu Y, Zhou Y, Liu J, et al. Supporting data for "A high-quality chromosomal genome assembly of the sea cucumber *Chiridota heheva* and its hydrothermal adaptation" GigaScience Database. 2023. http://doi.org/10.5524/102481.

Figure legends

Figure 1. The sampling site at the Kairei vent filed of Indian Ocean and the photo in situ at a depth of 2428 m.

Figure 2. Genome assembly and sequencing analysis of the Kairei vent *Chiridota heheva*. (A) Hi-C interaction heat map. (B) High-quality assembly of 19 chromosomes with genes coverage, GC content, and repetitive elements of LTR, LINE and SINE.

Figure 3. Phylogenetic and syntenic relationships. (A) Phylogenetic relationship and divergence time based on ten metazoan species orthologous from OrthoFinder. The number on the branches represents of gene family expansion (red) or contraction (green) (B) Statistics of orthologous gene numbers in these species. Single-copy orthologs, gene that have only one copy in each species and have homologs in other species; Multiple-copy orthologs, gene that have more than one copy in each species, together with homologs in other species; Unique orthologs, gene in each species without homologs in other species; Other orthologs, orthologs that do not belong to any type of the above orthologs; Unclustered genes, gene that do not clustered. (C) Synteny between the Kairei vent *Chiridota heheva* and *Apostichopus japonicus* in dot plot.

Figure 4. GO enrichment analysis of expanded gene families of the Kairei vent *Chiridota heheva*.

Figure 5. Positively selected amino acid sites of gene *RFC2* in the Kairei vent *Chiridota heheva*. (A) Ten positively selected amino sites in protein sequence. (B) Distribution of ten positively selected amino sites in three dimensional structure from AlphaFold.

Figure 6. UpSet relationship of protein sets of six echinoderms (*A. japonica*, *A. planci*,

O. spiculata, *S. purpuratus*, the Haima cold seep *C. heheva*, and the Kairei vent *C. heheva*).

Figure 7. GO enrichment analysis of the unique genes of the Kairei vent *Chiridota heheva*.

Table 1. Assembly statistics of the *Chiridota heheva* genome assembly.

Assembly statistics	Value
Genome size (bp)	1,434,753,151
Number of scaffolds	1399
Number of chromosome-scale scaffolds	19
N50 of scaffolds (bp)	53,240,875
L50 of scaffolds	11
Chromosome-scale scaffolds (bp)	1,431,787,880
GC content of the genome (%)	37.1231
Error rate	0.0021
**BUSCO analysis**	
Library	Metazoan_odb10 (954)
Complete	94.50% (902)
Complete and single copy	93.50% (892)
Complete and duplicated	1.00% (10)
Fragmented	2.80% (27)
Missing	2.70% (25)
**Inspector analysis**	
Mapping rate	99.76%
Depth	20.3745
Quality value (QV)	29.7695
Error rate (E, from $QV = -10\log_{10}E$)	0.0011

Table 2. Repeatitive elements of the *Chiridota heheva* genome assembly.

Assembly feature	Number of elements	Value (bp)
DNA	15,768	87,173,116
LINE	305,867	353,178,525
SINE	63,461	11,551,395
LTR	16,380	23,197,590
Low complexity	27,548	1,906,077
Satellite	44,353	22,459,277
Simple repeat	365,433	399,894,357
Small RNA	18,648	2,502,674
Total	70.80%	1,016,101,549
Unknown	1,635,141	380,635,876

Table 3. Annotation statistics of the *Chiridota heheva* genome assembly.

Databases of genes annotation	Value
Number of predicted protein-coding genes	32,434
Number of annotated protein-coding genes	24,606

Number of genes annotation to Interproscan	16,086
Number of genes annotation to GO	10,566
Number of genes annotation to Pfam	14,171
Number of genes annotation to KEGG	7,244
Number of genes annotation to NR	18,038
Number of genes annotation to Swiss-Prot	10,711
Number of genes annotation toTrEMBL	17,697

Table 4. The positively selected genes of *Chiridota heheva* from the Kairei vent.

**Gene**	**Description**	**FDR**
MAEA	Macrophage erythroblast attacher	2.99E-04
POLB	DNA polymerase beta	2.99E-04
SOD1	Superoxide dismutase, Cu-Zn family	2.99E-04
URB1	Nucleolar pre-ribosomal-associated protein 1	1.21E-03
FAN1	Fanconi-associated nuclease 1	1.21E-03
RFC2	Replication factor C subunit 2	1.87E-03
FARSA	Phenylalanyl-tRNA synthetase alpha chain	4.87E-03
NUP88	Nuclear pore complex protein *Nup88*	5.02E-03
KDM2A	F-box and leucine-rich repeat protein 11	5.16E-03
RFT1	Oligosaccharide translocation protein *RFT1*	8.10E-03
RNF216	E3 ubiquitin-protein ligase *RNF216*	1.08E-02
SPG7	Spastic paraplegia 7	1.40E-02
BBOX1	Gamma-butyrobetaine dioxygenase	1.40E-02
RPS16	Small subunit ribosomal protein *S16e*	1.40E-02
BRCA1	Breast cancer type 1 susceptibility protein	1.60E-02
SDR42E1	Short-chain dehydrogenase/reductase family 42E member 1	2.05E-02
SSF1_2	Ribosome biogenesis protein *SSF1/2*	2.37E-02
LSM4	U6 snRNA-associated Sm-like protein *LSm4*	2.64E-02
PSTK	O-phosphoseryl-tRNA(Sec) kinase	3.13E-02
ESCO1	N-acetyltransferase	3.14E-02
TTLL9	tubulin polyglutamylase *TTLL9*	3.20E-02
HOGA1	4-hydroxy-2-oxoglutarate aldolase	3.43E-02
DCLK1	Doublecortin-like kinase 1	3.80E-02
LDHD	D-lactate dehydrogenase (cytochrome)	4.14E-02
TEP1	telomerase protein component 1	4.14E-02
SIRT4	NAD+-dependent protein deacetylase sirtuin 4	4.14E-02
SLC35F5	Solute carrier family 35, member F5	4.22E-02
SEH1	Nucleoporin *SEH1*	4.47E-02

Figure

Click here to access/download;Figure;Figure_revised.doc

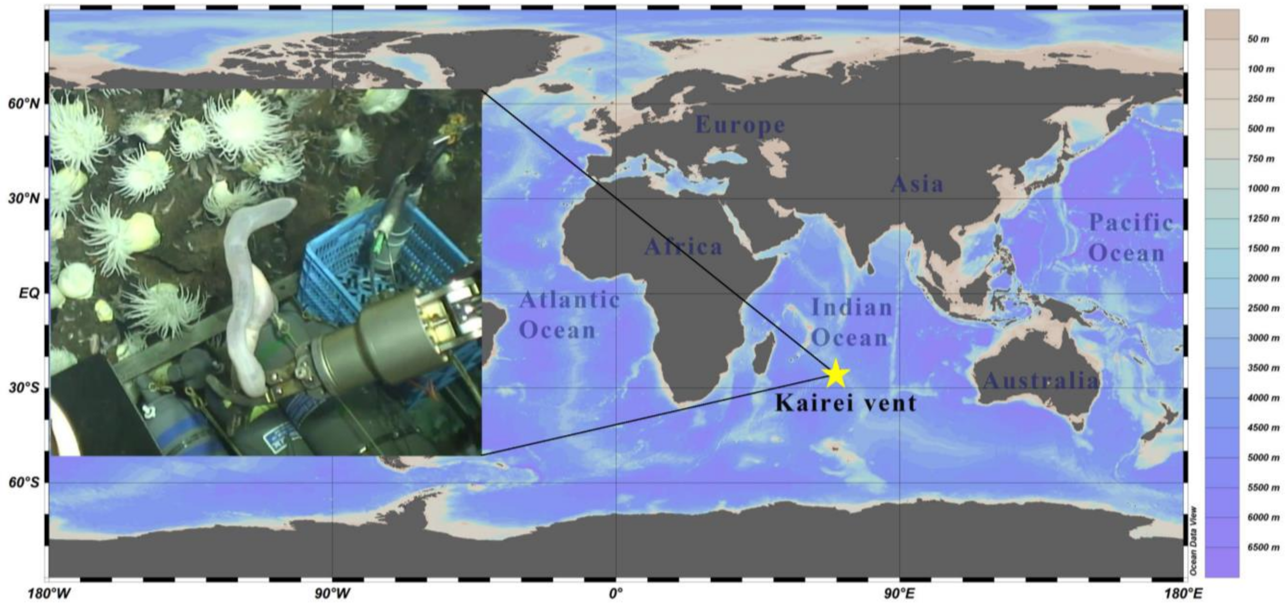

Figure 1

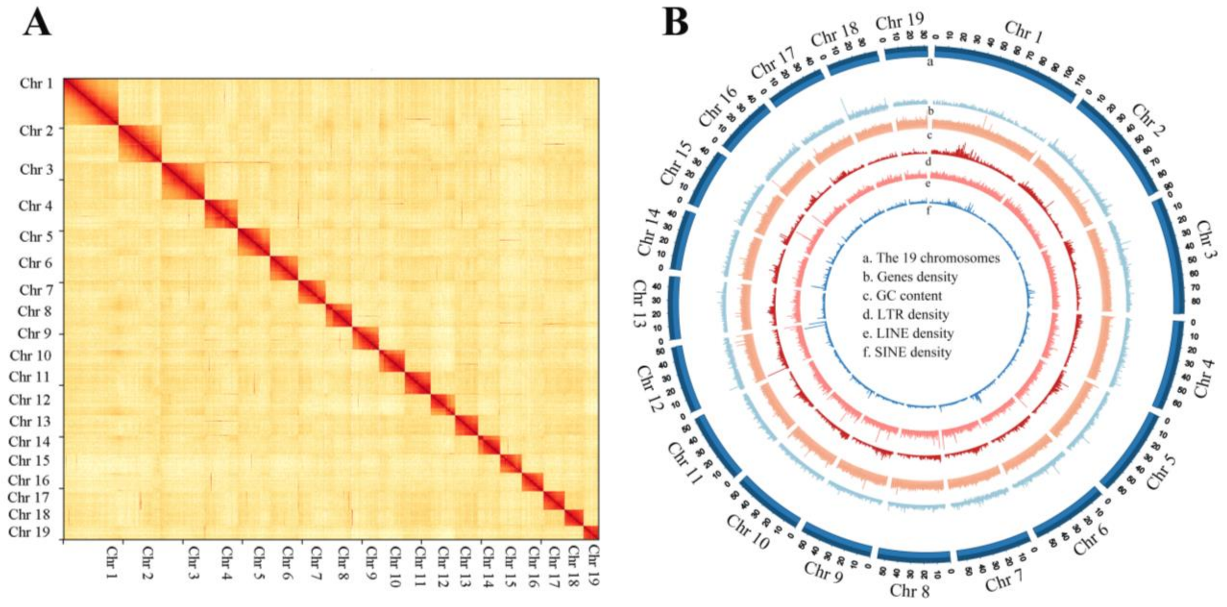

Figure 2

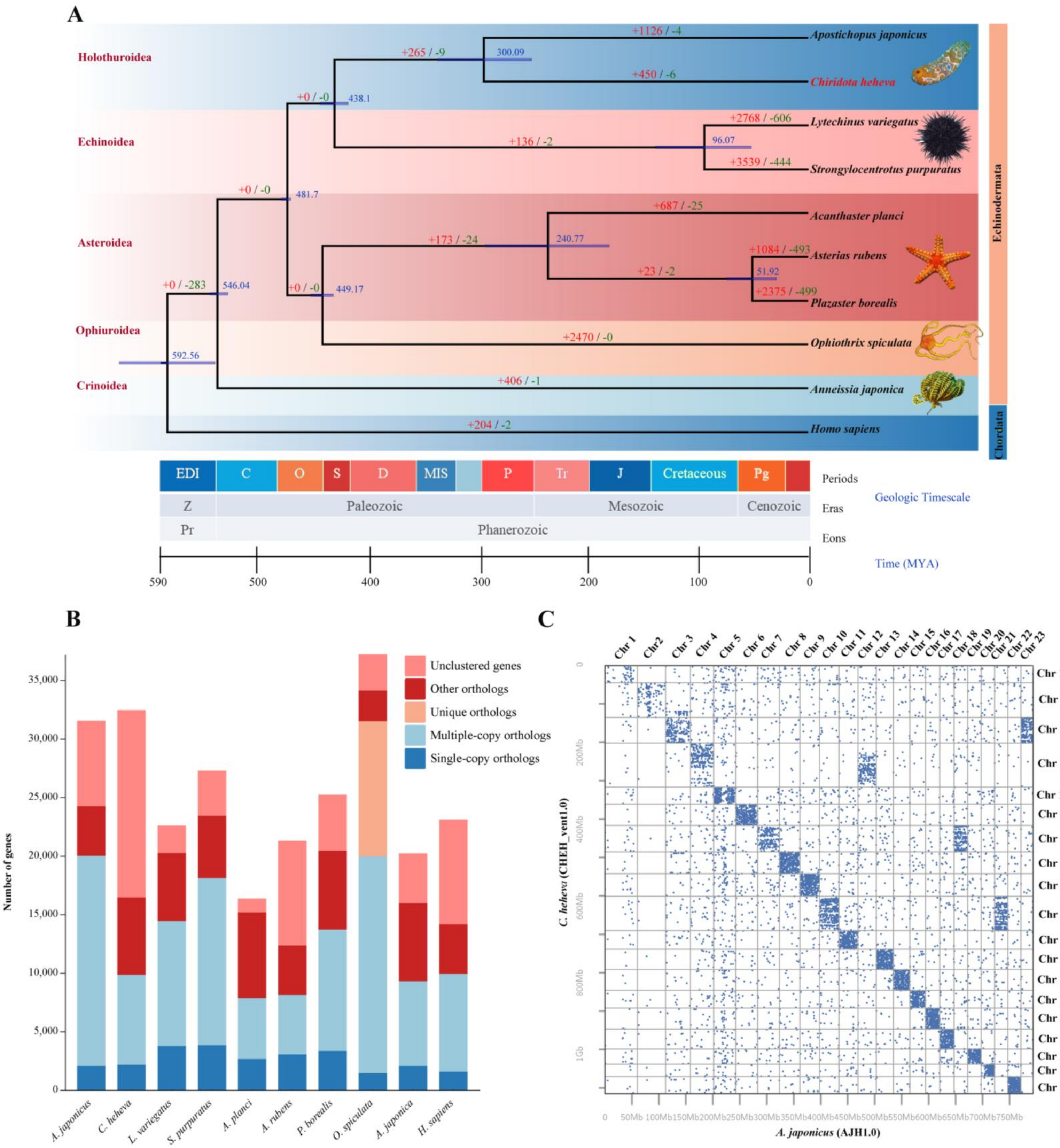
A
Holothuroidea
Echinoidea
Asteroidea
Ophiuroidea
Crinoidea
+1126 / -4
Apostichopus japonicus
+265 / -9
300.09
+450 / -6
Chiridota heheva
+0 / -0
438.1
+2768 / -606
Lytechinus variegatus
+136 / -2
96.07
+3539 / -444
Strongylocentrotus purpuratus
+0 / -0
481.7
+687 / -25
Acanthaster planci
+173 / -24
240.77
+1084 / -493
Asterias rubens
+23 / -2
51.92
+2375 / -499
Plazaster borealis
+0 / -283
546.04
+0 / -0
449.17
+2470 / -0
Ophiothrix spiculata
592.56
+406 / -1
Anneissia japonica
+204 / -2
Homo sapiens
Echinodermata
Chordata
EDI
C
O
S
D
MIS
P
Tr
J
Cretaceous
Pg
Periods
Z
Paleozoic
Mesozoic
Cenozoic
Eras
Pr
Phanerozoic
Eons
Geologic Timescale
Time (MYA)
590
500
400
300
200
100
0
B
Number of genes
35,000
30,000
25,000
20,000
15,000
10,000
5,000
0
Unclustered genes
Other orthologs
Unique orthologs
Multiple-copy orthologs
Single-copy orthologs
A. japonicus
C. heheva
L. variegatus
S. purpuratus
A. planci
A. rubens
P. borealis
O. spiculata
A. japonica
H. sapiens
C
Chr 1
Chr 2
Chr 3
Chr 4
Chr 5
Chr 6
Chr 7
Chr 8
Chr 9
Chr 10
Chr 11
Chr 12
Chr 13
Chr 14
Chr 15
Chr 16
Chr 17
Chr 18
Chr 19
Chr 20
Chr 21
Chr 22
Chr 23
Chr 15
Chr 2
Chr 5
Chr 1
Chr 16
Chr 9
Chr 4
Chr 7
Chr 6
Chr 3
Chr 13
Chr 11
Chr 10
Chr 14
Chr 8
Chr 12
Chr 18
Chr 19
Chr 17
C. heheva (CHEH_vent1.0)
0
200Mb
400Mb
600Mb
800Mb
1Gb
0
50Mb
100Mb
150Mb
200Mb
250Mb
300Mb
350Mb
400Mb
450Mb
500Mb
550Mb
600Mb
650Mb
700Mb
750Mb
A. japonicus (AJH1.0)

Figure 3

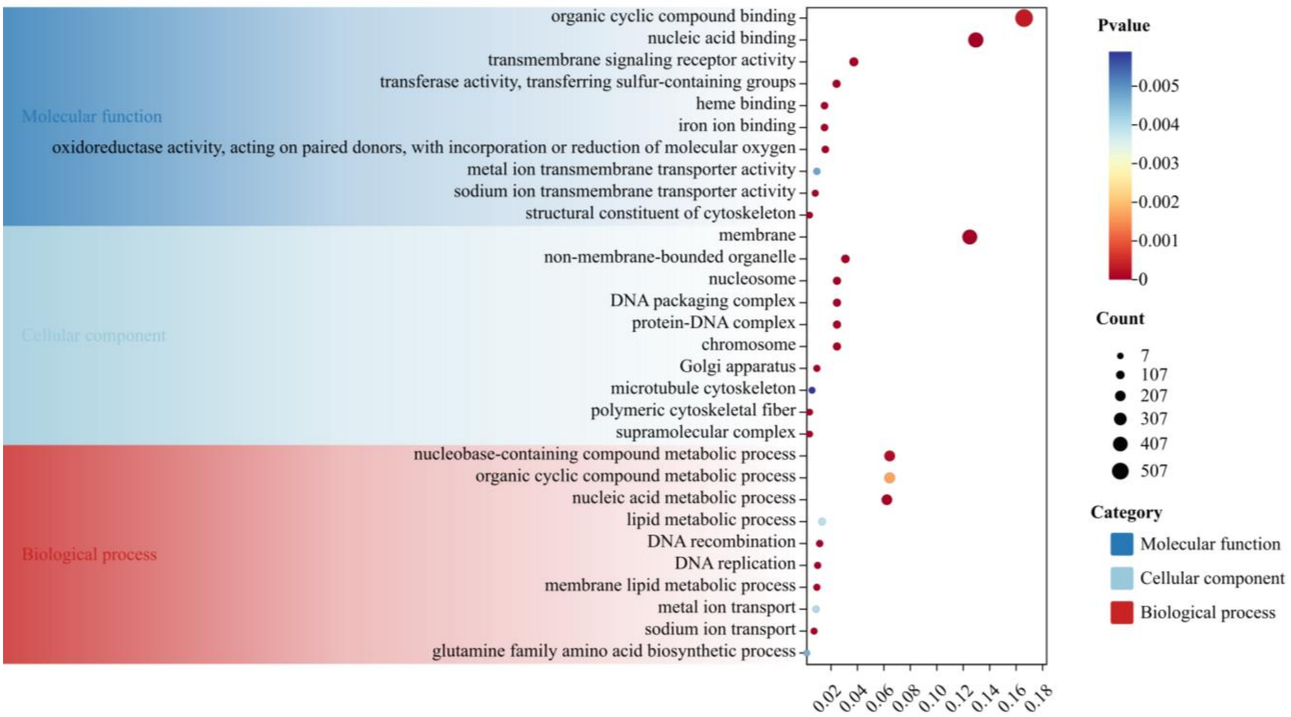

Molecular function
Cellular component
Biological process
organic cyclic compound binding
nucleic acid binding
transmembrane signaling receptor activity
transferase activity, transferring sulfur-containing groups
heme binding
iron ion binding
oxidoreductase activity, acting on paired donors, with incorporation or reduction of molecular oxygen
metal ion transmembrane transporter activity
sodium ion transmembrane transporter activity
structural constituent of cytoskeleton
membrane
non-membrane-bounded organelle
nucleosome
DNA packaging complex
protein-DNA complex
chromosome
Golgi apparatus
microtubule cytoskeleton
polymeric cytoskeletal fiber
supramolecular complex
nucleobase-containing compound metabolic process
organic cyclic compound metabolic process
nucleic acid metabolic process
lipid metabolic process
DNA recombination
DNA replication
membrane lipid metabolic process
metal ion transport
sodium ion transport
glutamine family amino acid biosynthetic process
0.02 0.04 0.06 0.08 0.10 0.12 0.14 0.16 0.18
Pvalue
0.005
0.004
0.003
0.002
0.001
0
Count
7
107
207
307
407
507
Category
Molecular function
Cellular component
Biological process

Figure 4

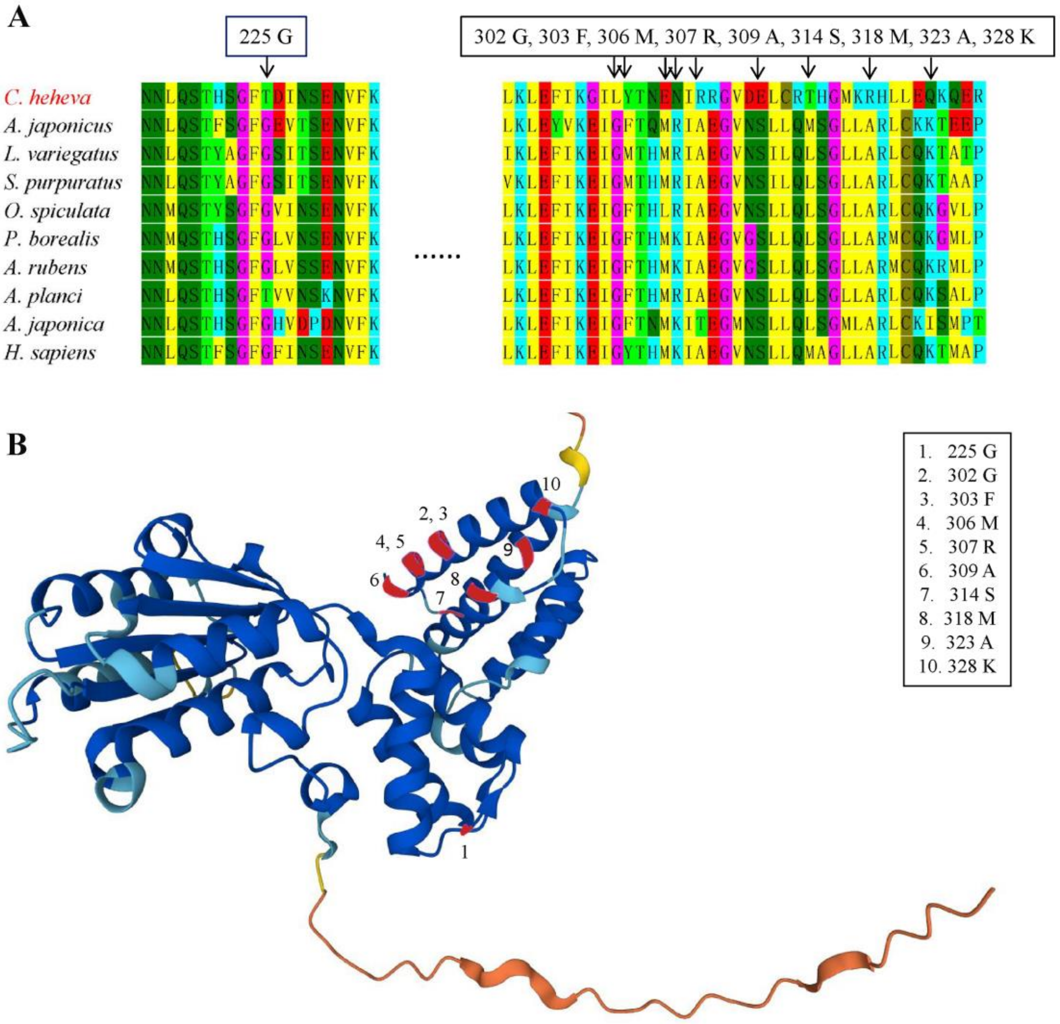

A
225 G
302 G, 303 F, 306 M, 307 R, 309 A, 314 S, 318 M, 323 A, 328 K
C. heheva
A. japonicus
L. variegatus
S. purpuratus
O. spiculata
P. borealis
A. rubens
A. planci
A. japonica
H. sapiens
......
B
1. 225 G
2. 302 G
3. 303 F
4. 306 M
5. 307 R
6. 309 A
7. 314 S
8. 318 M
9. 323 A
10. 328 K

Figure 5

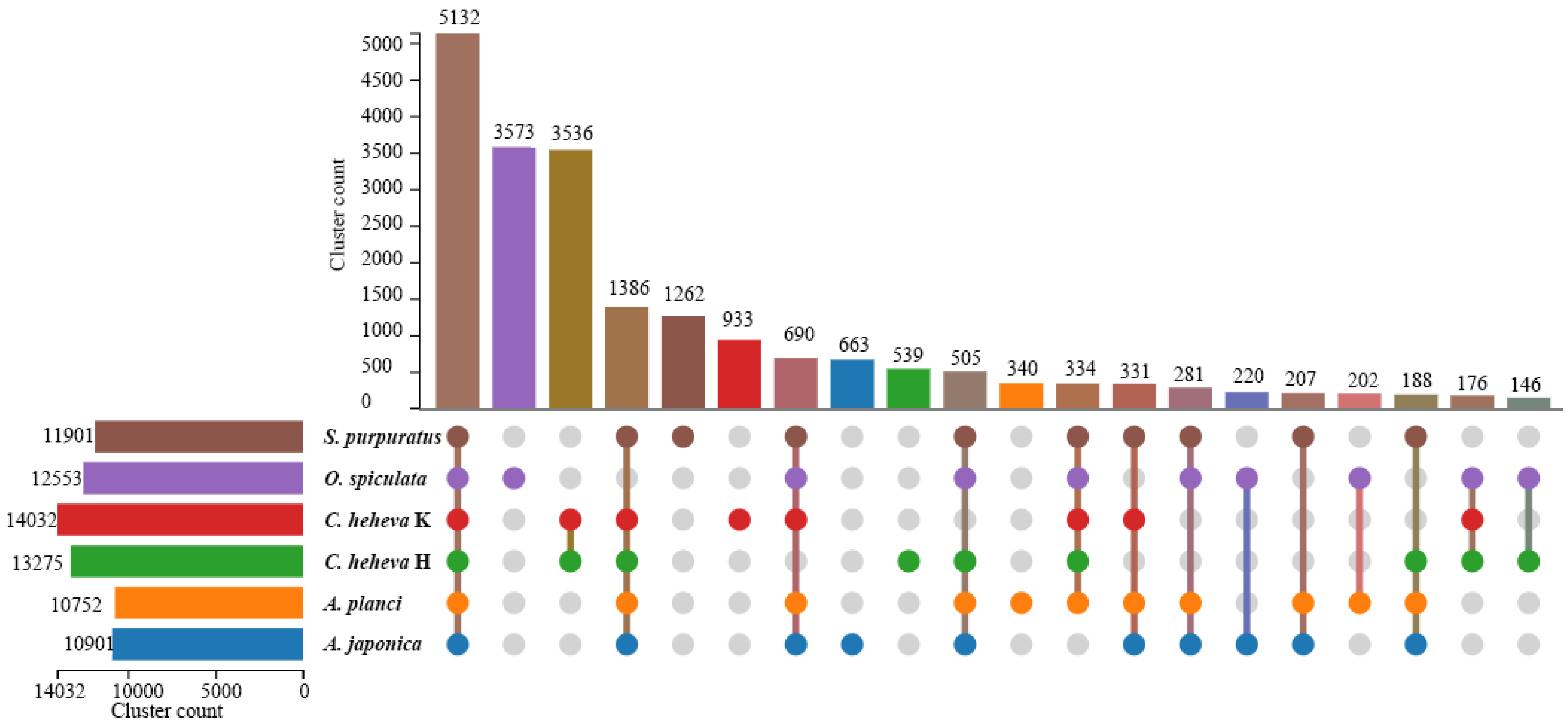

Figure 6

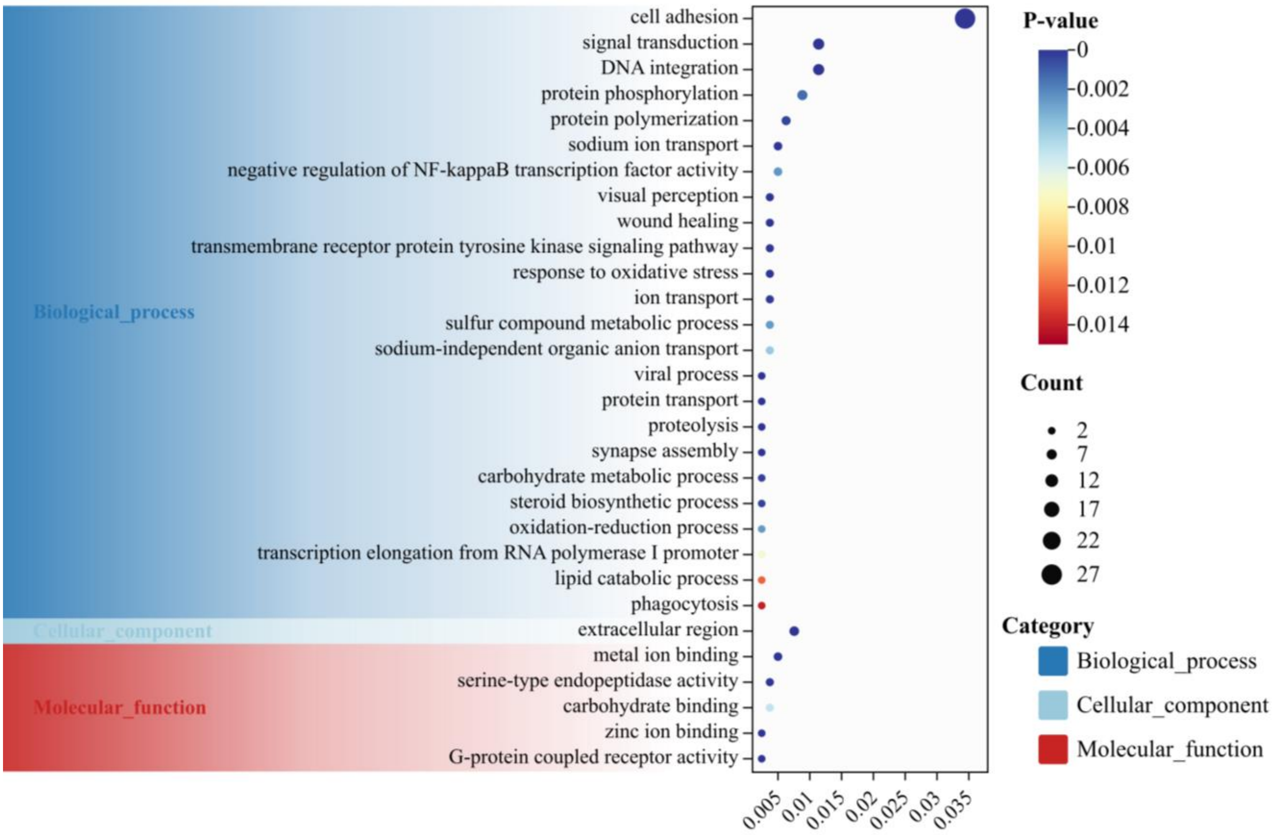
cell adhesion
signal transduction
DNA integration
protein phosphorylation
protein polymerization
sodium ion transport
negative regulation of NF-kappaB transcription factor activity
visual perception
wound healing
transmembrane receptor protein tyrosine kinase signaling pathway
response to oxidative stress
ion transport
sulfur compound metabolic process
sodium-independent organic anion transport
viral process
protein transport
proteolysis
synapse assembly
carbohydrate metabolic process
steroid biosynthetic process
oxidation-reduction process
transcription elongation from RNA polymerase I promoter
lipid catabolic process
phagocytosis
extracellular region
metal ion binding
serine-type endopeptidase activity
carbohydrate binding
zinc ion binding
G-protein coupled receptor activity
Biological_process
Cellular_component
Molecular_function
0.005
0.01
0.015
0.02
0.025
0.03
0.035
P-value
0
0.002
0.004
0.006
0.008
0.01
0.012
0.014
Count
2
7
12
17
22
27
Category
Biological_process
Cellular_component
Molecular_function

Figure 7

Click here to access/download
Supplementary Material
Supplementary Figure S1..png

Click here to access/download
Supplementary Material
Supplementary Figure. S2..png

Click here to access/download
Supplementary Material
Supplementary Table S1..xlsx

Click here to access/download
Supplementary Material
Supplementary Table S2..xlsx

Click here to access/download
Supplementary Material
Supplementary Table S3..xlsx

Click here to access/download
Supplementary Material
Supplementary Table S4..xlsx

Click here to access/download

Supplementary Material

Supplementary Table S5..xlsx

Click here to access/download
Supplementary Material
Supplementary Table S6..xlsx

Click here to access/download
Supplementary Material
Supplementary Table S7..xlsx

Click here to access/download
Supplementary Material
Supplementary text S1 Commands for analyses_revised2.docx

Click here to access/download
Supplementary Material
Responese To Reviewers Comments_revised2.docx
