# Supplementary material for: A high-quality chromosomal genome assembly of the sea cucumber Chiridota heheva and its hydrothermal adaptation
Source: Gigascience. 2024 Jan 4;13:giad107. doi: 10.1093/gigascience/giad107 (PMC10764150; doi:10.1093/gigascience/giad107)
Supplement: giad107_Supplemental_Files [file giad107_supplemental_files.zip › Supplementary text S1 Commands for analyses_revised2.docx]

**Supplementary text S1 Commands for analyses**

1. **Genome assembly**
   1. **Hifi reads assembly**

Hifiasm version 0.16.1-r375 was used to PacBio HiFi reads assembly.

hifiasm -o $name -t 32 $hifi_reads

Purge_dups version 1.2.5 (purge dups, RRID:SCR_021173) was used for redundancy purge.

Step 1: Run minimap2 to align pacbio data and generate paf files, then calculate read depth histogram and base-level read depth.

minimap2 -t 54 -x map-pb $name_asm $hifi_reads | gzip -c - > $name.paf.gz

Step 2: Split an assembly and do a self-self alignment.

bin/split_fa $name_asm > $name_asm.split

minimap2 -xasm5 -DP $name_asm.split $name_asm.split | gzip -c - > $name_asm.split.self.paf.gz

Step 3: Purge haplotigs and overlaps.

bin/purge_dups -2 -T cutoffs -c PB.base.cov $name_asm.split.self.paf.gz > dups.bed 2> purge_dups.log

Step 4: Get purged primary and haplotig sequences from draft assembly.

bin/get_seqs -e dups.bed $name_asm

The purged.fa genome was used for the next analysis.

- 1. **Hi-C reads combined**

Juicer version 1.6 (Juicer, RRID:SCR_017226) was used to analyze Hi-C reads combined with contig-level genome.

./juicer.sh -g $genome_name -s MboI -z $reference_genome -y $restriction_sites_MboI.txt -p $chrom.sizes_path -t 50 -D $scrDir -d $topDir

3D-DNA version 190716 (3D de novo assembly, RRID:SCR_017227) was used to primarily correct misjoin, order and orient in the scaffold obtained the potential chromosomal groups.

run-asm-pipeline.sh -m haploid -i 15000 -r 5 $reference_genome $merged_nodups.txt

Juicebox version 1.11.08 was then used to manually order the scaffolds of the result from 3D-DNA. The tool 3D-DNA was used again to obtain the final chromosome assembly for further analysis.

run-asm-pipeline-post-review.sh -r $juicebox.assembly $reference_genome $merged_nodups.txt

The chr_genome.fa genome was used for the next analysis.

1. **Genome annotation**
   1. **Repetitive elements annotation**

RepeatModeler version 2.0.1 (RepeatModeler, RRID:SCR_015027) and RepeatMasker version open-4.0.6 (RepeatMasker, RRID:SCR_012954) were used for searching repetitive elements in chr_genome.fa and generated a soft-masked genome chr_soft_genome.fa.

- 1. **Structure annotation**

Augustus version 3.4.3 (Augustus, RRID:SCR_008417), GlimmerHMM version 3.0.4 (GlimmerHMM, RRID:SCR 002654) , and GeneID version 1.4.5 (Entrez Gene, RRID:SCR_002473) were used in *ab initio* gene prediction.

Exonerate version 2.2.0 (Exonerate, RRID: SCR_016088) was employed for protein homologous annotation in evidence-based gene prediction.

PASA version 2.5.2 (PASA, RRID:SCR_014656) was applied for transcriptomic annotation in evidence-based gene prediction.

EVidenceModeler version 1.1.1 (EVidenceModeler, RRID:SCR_014659) produced a weighed consensus protein set by combining the results from *ab initio* gene models and evidence-based gene models.

1. **Orthology prediction**

OrthoFinder version 2.5.4 (OrthoFinder, RRID:SCR_017118) was applied to determine and cluster gene families among 10 metazoans.

python orthofinder.py -f $protein_set_path -t 30

1. **Phylogenomic analysis**

RAxML version 8.2.3 (RAxML, RRID:SCR_006086 ) was used to constructed a phylogenomic tree.

bin/clustalw2 -INFILE=connect.cds.fa -CONVERT -TYPE=DNA -OUTFILE=connect.cds.fa.PHYLIP -OUTPUT=PHYLIP

bin/raxmlHPC-PTHREADS -s connect.cds.fa.PHYLIP -n connect.cds.fa.PHYLIP -m GTRGAMMA -f a -x 12345 -N 100 -p 12345 -T 30 -o $outgroup_name

1. **Genome synteny analysis**

JCVI version 0.18 (RRID:SCR_021641) was used to perform protein sequence alignment and search for syntenic blocks in all the genes.

python -m jcvi.compara.catalog ortholog $name1_chr $name2_chr --no_strip_names

1. **Gene family analysis**

CAFE version 4.2.1 (CAFE, RRID: SCR_005983) was used to detect the gene family expansion and contraction.

cafe cafetutorial_run1.sh #edit tree imformation in shell script

1. **Positive selection analysis**

The codeml in PAML package version 4.9 (PAML, RRID:SCR_014932) was used to run the positive selection analysis.

Before the positive selection analysis, the tree and ctl files were prepared according to scripts which are available at https://github.com/yongzhiyang2012/genome_analysis/blob/c6db712090afe7488fc94483ca284daf3abebf10/evolution/03.paml/04.prepare.paml.Creat.TREE.pl

and <https://github.com/yongzhiyang2012/genome_analysis/blob/c6db712090afe7488fc94483ca284daf3abebf10/evolution/03.paml/05.prepare.paml.Creat.CTL.pl.>

perl $prepare.paml.Creat.TREE.pl tree species # tree file contains a tree that like (((((Apostichopus,Chiridota),(lva,spu)),((apl,(aru,pbo)),Ophiothrix)),anj),hsap); species is the branch that you want to mark, such as Chiridota in the tree file

perl $prepare.paml.Creat.CTL.pl $model #give the model type as branch or branch-site

And then run the positive selection analysis based on the tree and ctl files above.

paml4.9j/bin/codeml $model_ctl
