# Supplementary figures and images for: A high-quality chromosomal genome assembly of the sea cucumber Chiridota heheva and its hydrothermal adaptation
Source: Gigascience. 2024 Jan 4;13:giad107. doi: 10.1093/gigascience/giad107 (PMC10764150; doi:10.1093/gigascience/giad107)

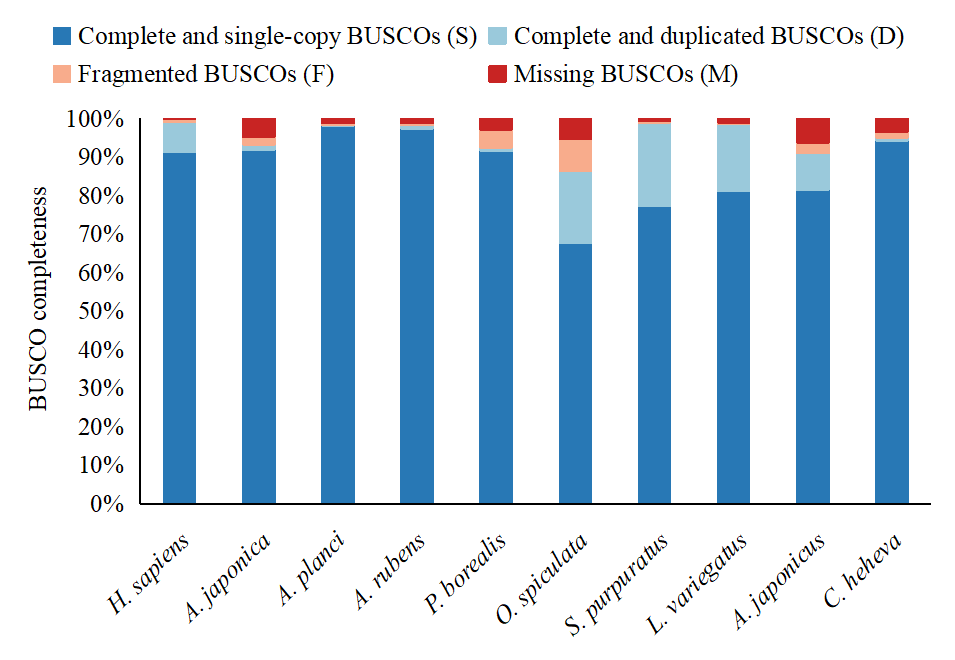

Supplement: giad107_Supplemental_Files [file giad107_supplemental_files.zip › Supplementary Figure S1..png]

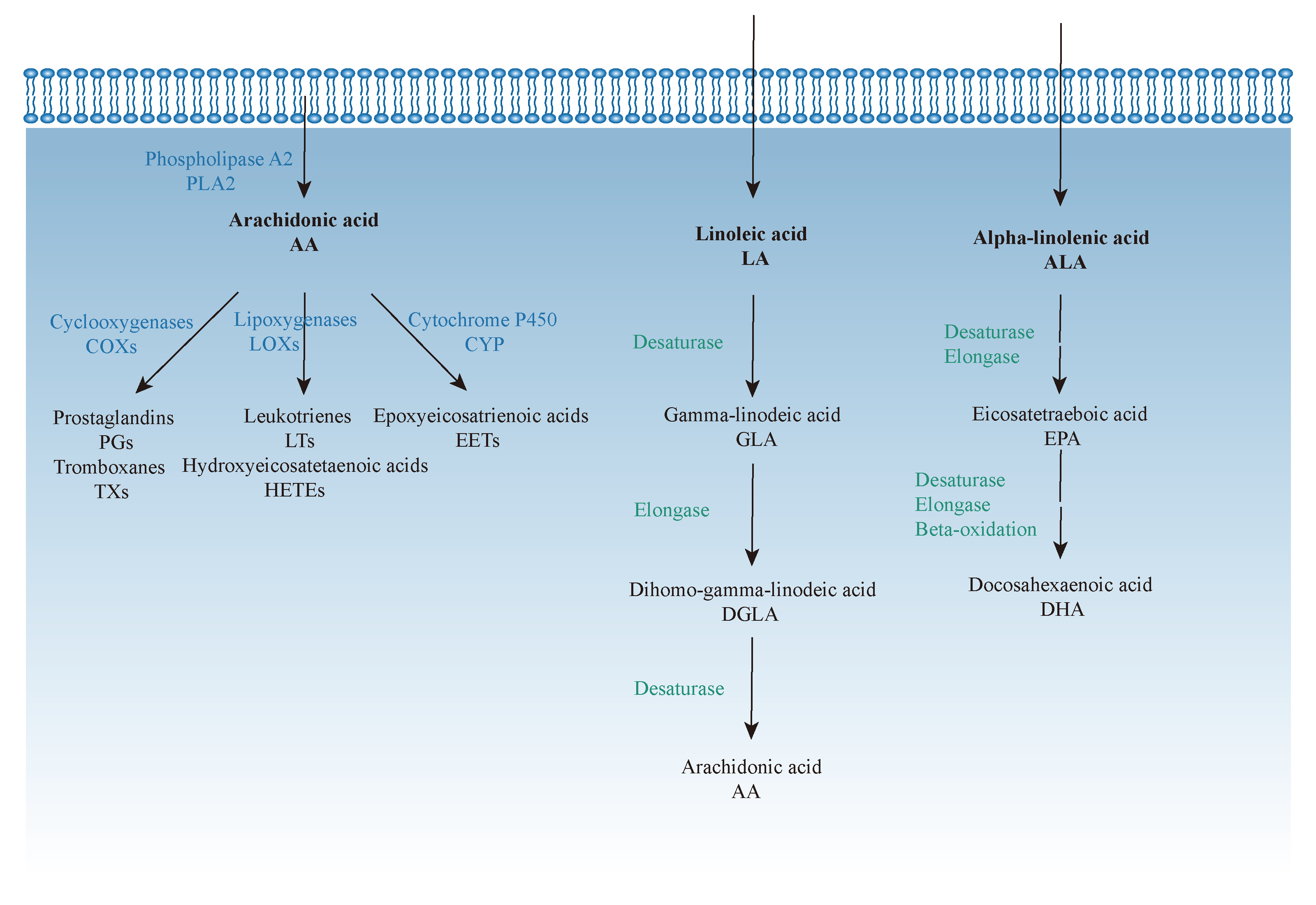

Supplement: giad107_Supplemental_Files [file giad107_supplemental_files.zip › Supplementary Figure. S2..png]
