# Supplementary material for: A high-quality chromosomal genome assembly of the sea cucumber Chiridota heheva and its hydrothermal adaptation
Source: Gigascience. 2024 Jan 4;13:giad107. doi: 10.1093/gigascience/giad107 (PMC10764150; doi:10.1093/gigascience/giad107)
Supplement: giad107_Response_to_Reviewer_Comments_Original_Submission [file giad107_response_to_reviewer_comments_original_submission.pdf]

Author's Response To Reviewer Comments

Response to Reviewers

Reviewer #1: Pu et al. reported a chromosome-level genome assembly of C. heheva collected from hydrothermal vent. The authors dissected the genetic basis of adaptation in vent-dwelling C. heheva by performing protein family expansion/contraction and positively selected gene analysis. Here are my comments of the manuscript:

1.There are many grammatical problems in the manuscript. These include errors in tenses, verb conjugation, and definite and indefinite articles. Please check the grammar of the entire manuscript again.

We have carefully revised the manuscript according to the reviewers' comments and suggestions, and also have re-scrutinized to polish the grammar with the help of a friendly native English speaker. These changes of grammar revision will not influence the content and framework of this paper. Here we did not list the changes but marked in blue in the revised manuscript.

2.Line 279, I am not convinced that expansion of protein family related to lipid metabolism suggested vent-dwelling C. heheva had more fatty acid in the membrane. Fatty acid is just one kind of lipid.

Thank you for pointing out this issue. According to the comment, we have added more information about the compositions of membrane, lipid metabolisms, and membrane fluidity in the revised manuscript (lines 258-276). Besides the revised part in the revised manuscript, supplementary figure S2 was made for clear understanding of essential fatty acid metabolisms.

3.Line 282, specify what are toxic iron and safe iron status.

Thank you for pointing out this issue. The redox-inert iron are in the safe form, which often bound to proteins or stored in ferritin. The redox-active iron are in the potentially toxic and fatal form, through the excess redox-active iron could generate reactive oxygen species (ROS) and lead to cell damages. We have re-written this part and added more information in the revised manuscript (lines 28-287).

4.Line 283, specify what are "These expanded gene families".

Thank you for pointing out this issue. “These expanded gene families” are the enriched gene families of iron ion binding and metal ion transport. We have re-written this part in the revised manuscript (lines 287-289).

5.Line 289, What is the relationship between DNA-protected abilities and DNA replication, recombination, DNA-associated protein? I am not convinced that expansion of protein family related to DNA replication implied vent-dwelling C. heheva had better ability in DNA protected ability.

We are very sorry for our incorrect using of “DNA-protected abilities” in here. We have modified this part in the revised manuscript (lines 293-295).

6.Line 301, how did "deep-sea fauna obtained PSGs through adaptive evolution"? Gene were positively selected during adaptive evolution.

Thank you for pointing out this issue. Actually, natural selection, both positive and negative, is pervasive in many genomes. The positive selection of amino acid site may causes changes in genetic traits, which changes of positively selected genes lead to well adaption to deep-sea environment through molecular adaptive evolution. We have re-written this part in the revised manuscript (lines 305-306).

7.Line 307, explain how genes "mainly induced by high hydrostatic pressure".

Thank you for pointing out this issue. The temperature of the chimneys is extremely high (~ 350 °C), and mix directly with ambient seawater (~ 2°C). Although the sample of this study was collected next to the chimney, the temperature of this sample may cold as ambient sea water. The effects of high hydrostatic pressure on DNA are often difficult to disentangle from effects caused by low temperature that are often characteristic of the deep sea. For the hydrothermal vent, DNA damages may causes by high hydrostatic pressure, low temperature, and other toxic components that emitted with fluid.
Therefore, we have deleted " that mainly induced by high hydrostatic pressure" in the revised manuscript.

8.Line 323, terms cannot be enriched to adapt the iron-rich environment. Only organisms can adapt to a specific environment.

Thank you so much for your careful check and your suggestion. All the part of unique genes evolution have re-written in the revised manuscript.

9. Line 330, explain the relationship between the unique genes and DNA repair. I don't think that the unique genes identified in the study are related to DNA repair.

Thank you for pointing out this issue. Some of the unique genes, such as TFIIH, SSB, and RAD51, are enriched in categories related to DNA repair. All the part of unique genes evolution have re-written in the revised manuscript.

Reviewer #2: In this manuscript, Pu et al present a chromosome-level genome assembly of the holothurian Chiridota heheva. The assembly and annotation process was carried out with an up-to-date sequencing and methodological strategy and has resulted in a high quality genome assembly of this species, such that two assemblies of these species are now available. This assembly has the nice addition of HiC scaffolding which has resulted in chromosome-length scaffolds. This work is accompanied by gene family expansion analyses and tests for positive selection in coding sequence to identify putatively adaptive changes in genes associated with living in hydrothermal environments.

For clarity, I've organized my comments into two sections: 1) Rational and Significance and 2) Methods and Results. These comments each have major and minor components. Overall, while the assembly itself is nice and now available at a chromosome-level, I think the description of the rationale of this study and how it is distinguished from the already published assembly is needed. As written, the ability to discern hydrothermal adaptations from a more general suite of adaptations associated with the deep sea is unclear. In addition, more methodological detail and revision is needed with each of the functional genomic analyses, and reconciling these results with those already published will be helpful in revising their interpretation.

Rational and Significance

Major Comments

1.The assembly reported in this manuscript represents the second assembly for this species. The rationale for generating this assembly is stated to be that the individual C. heheva was collected from population inhabiting the Kairei hydrothermal vent (herein C. heheva K) whereas the earlier published version of this species genome was from an individual collected from a population inhabiting a different deep sea environment, a cold seep (herein C. heheva H: Zhang et al, 2022 Comms Bio). However, C. heheva is a cosmopolitan holothurian found in several harsh deep sea environments, so it is not clear whether any genetic-signatures of adaptations detected in this assembly are truly associated with hydrothermal vent adaptation or not. Given these individuals (K and H) represent the same species, presently it is difficult to say whether the genome assembly of single individual is an appropriate measure of local adaption to hydrothermal vent habitats unless these populations been isolated for a very long amount of time (which should be reported). Otherwise, population genetic data would be needed in this case. It is possible that this species is instead adapted to harsh deep sea environments broadly-sepaking, so the utility of comparisons between single individuals is difficult to interpret. Given a genome assembly of this species is already published, more information justifying the rational of this study is needed in this regard.

We gratefully appreciate for your careful check and valuable suggestion.
In the one hand, chiridotid holothurians are often the most frequently observed in the hydrothermal vent according to previous reports (Zhou et al., 2018; Sun et al., 2020; Thomas et al., 2020; Sun et al., 2021; Prakash et al., 2022). This point was mentioned in the manuscript (lines 86-87). That indicates our sample of C. heheva that collected from the Kairei vent is not occasional.
In the other hand, Indian Ocean is well-known as the hydrothermal fields including Dodo, Solitaire, Edmond, Kairei, Longqi, Tiancheng, Pelagia, Wocan, Onnuri, Old City, Sonne, Yokoniwa, Mount Jourdanne, Tianzuo, Yuhuang-1, Duanqiao-1, and so on (Nakamura et al., 2012; Prakash et al., 2022). Besides the hydrostatic pressure, the high contents of chemicals, which diffusing in from the surrounding vent fluid, is the principal characters of hydrothermal vent. The effects of extreme hydrothermal vent conditions are often difficult to disentangle from effects caused by hydrostatic pressure that are often characteristic of the deep sea. Iron-rich condition is one of the signatures in the Kairei vent, the iron-rich adaptation has observed in the previous studies (Warén et al., 2003; Nakamura et al., 2012; Okada et al., 2019). In our study, we found some evidences for iron-rich adaptation in gene families enrichment and the unique genes. These evidences could be found in gene family evolution and unique genes evolution of results part in the manuscript.
Nevertheless, samples of C. heheva that collected from hydrothermal vent are limited up to now. The observation of population adaptation for hydrothermal vent is required in the further study.

The main references in here.
Zhou, Y, Zhang, DS, Zhang, RY, et al. Characterization of vent fauna at three hydrothermal vent fields on the Southwest Indian Ridge: Implications for biogeography and interannual dynamics on ultraslow-spreading ridges. Deep Sea Research Part I: Oceanographic Research Papers, 2018; 137: 1-12.
Thomas, EA, Liu, RY, Amon, D, et al. Chiridota heheva-the cosmopolitan holothurian. Mar Biodivers 2020; 50(6): 1-13.
Sun, J, Zhou, YD, Chen, C, et al. Nearest vent, dearest friend: biodiversity of Tiancheng vent field reveals cross-ridge similarities in the Indian Ocean. Roy Soc open Sci 2020b; 7(3): 200110.
Sun, SE, Sha, ZL, Xiao N. The first two complete mitogenomes of the order Apodida from deep-sea chemoautotrophic environments: New insights into the gene rearrangement, origin and evolution of the deep-sea sea cucumbers. Comparative Biochemistry and Physiology Part D: Genomics and Proteomics, 2021; 39: 100839.
Prakash, LS, Fernandes, SO, Ingole, B, et al. Biogeochemical characteristics of hydrothermal systems in the Indian Ocean. Systems Biogeochemistry of Major Biomes, 2022; 285-313.
Zhang, L, He, J, Tan, PP, et al. The genome of an apodid holothuroid (Chiridota heheva) provides insights into its adaptation to a deep-sea reducing environment. Commun Biol 2022; 5, 224.
Nakamura, K, Watanabe, H, Miyazaki, J, et al. Discovery of new hydrothermal activity and chemosynthetic fauna on the Central Indian Ridge at 18-20 °S. PLoS one 2012; 7(3): e32965
Okada, S, Chen, C, Watsuji, T, et al. The making of natural iron sulfide nanoparticles in a hot vent snail. PNAS 2019; 116(41): 20376-20381.
Warén, A, Bengtson, S, Goffredi, SK, et al. A hot-vent gastropod with iron sulfide dermal sclerites. Science 2003; 302, 1007.

2. Along the same lines, I think more effort is needed to distinguish this study from the previously published one of the same species in terms of the manuscript's writing. The phrasing of some of the writing is a little too close for comfort to what is already published. For example:
a. Introduction: Lines 86-90: "Chiridota heheva, a cosmopolitan Chiridotidae holothurian, has been found in hydrothermal vents, cold seeps, and other organic falls [25-26]. C. heheva may be an ideal model for studying how marine fauna have adapted to extreme deep-sea chemosynthetic environments." Vs Zhang: "Chiridota heheva Pawson & Vance, 2004 (Apodida: Chiridotidae) is one of the few echinoderms that occupies all three types of chemosynthetic ecosystems (hydrothermal vent, cold seep, and organic fall)… "The cosmopolitan distribution and special lifestyle of C. heheva make it an ideal model to study adaptation to deep-sea reducing environments in nonsymbiotic animals."

We gratefully appreciate for your valuable content and suggestion.
The main purpose of this study is reported the first chromosome-level genome of hydrothermal vent C. heheva with the preliminary investigation of hydrothermal adaptation. In this study, the adaptation for iron-rich environment was found in in the analysis of gene family evolution and unique gene evolution. It is

the main point that could distinguish between hydrothermal vent C. heheva and cold seep C. heheva. Considering the suggestion, we have added some information of iron-rich adaptation in gene family evolution and unique genes evolution of the revised manuscript.

According to the comment that the close writing, we have checked the writing around our manuscript carefully and re-written some sentences we found and the one you mentioned above, in order to clean up the closed sentences. Please check the changes in the revised manuscript.

b. the titles are similar: "The chromosome-level genome of Chiridota heheva (Holothuroidea, Apodida,Chiridotidae) provides insights into adaptation to the hydrothermal environment" vs Zhang: "The genome of an apodid holothuroid (Chiridota heheva) provides insights into its adaptation to a deep-sea reducing environment"

Thank you for your suggestion. We have changed the title from "Chromosome-level genome report and relevant analysis reveals insights into deep-sea adaptation of Chiridota heheva (Holothuroidea, Apodida,Chiridotidae), inhabiting in hydrothermal environment" to "A high-quality chromosomal genome assembly of the sea cucumber Chiridota heheva and its hydrothermal adaptation" in the revised manuscript.

3. This study replicates many of the same functional genomic analyses as Zhang et al (gene family expansion, tests for positive selection) but no overlap in results are reported. More on this in the next section below, but this represents a unique opportunity to compare two independent studies of the same species to validate predictions associated with deep sea adaptation. However, these cross-study comparisons are not mentioned even though they would be an effective way of distinguishing cold seep vs hydrothermal adaptation, should they exist.

We gratefully appreciate for your valuable comment. We also consider the works as reviewer suggested that comparative analysis between the hydrothermal vent C. heheva and cold seep C. heheva, but we did not found the useful information to support the adaption in this study. We have collected some chiridotid holothurians which distributed in a variety of habitats. The comparative genomic analysis of hydrothermal vent C. heheva and cold seep C. heheva will considered in our further study, that the comparative genomic analysis of the chiridotid holothurians in three types of chemosynthetic ecosystems. We really hope you will interest in our further study.
Moreover, in compared to the C. heheva genome of Zhang et al. (2022) reported, our C. heheva genome from hydrothermal vent while Zhang's genome from cold seep, our assembly level is chromosome while Zhang's is contig, our genome completeness is high as 94.50 % (BUSCO) while Zhang's completeness is 89.60 % (BUSCO). Thus, it is worth to report the chromosome-level genome assembly of hydrothermal vent C. heheva, and additional with preliminarily investigated of the vent-specific adaptation.

Minor

1. The second paragraph of the introduction is quite lengthy as it summarizes many adaptations associated with deep sea habitats. These species and adaptations list could probably be shortened, particularly for a Data Note type of manuscript.

Thank you for your nice suggestion. We have checked the second paragraph and re-written in the revised manuscript. We have tried our best to make this part clearly in short sentences.

Results and Methods

Major:

1. Tests were positive selection occurred at the species level as C. heheva was compared to several other metazoans. A very similar analysis was carried out in Zhang et al (also C heheva) yet there appears to be little to no overlap is genes with evidence of positive selection detected in both studies. I realize these animals came from different habitats, but shared species-level signatures of positive selection would be expected here, particularly in regard to deep sea adaption, broadly speaking. This large discrepancy is

concerning and needs justification or revision.

Thank you for pointing out this issue. We have noted this issue and repeated positive selected analysis again, but the results was same as before. We have checked some references and found similar problem (Liu et al., 2021; Shao et al., 2022).
In the one hand, positive selection is inferred if the ratio of nonsynonymous substitutions per nonsynonymous site to synonymous substitutions per synonymous site (dN/dS) is statistically significantly greater than one in a test of the neutral null hypothesis dN/dS = 1. Positively selected analysis is affected by multiple factors, such as the species in the background branches, the completeness of genome assembly, and the results of genomic annotation. In our study, the background branches (Homo sapiens, Anneissia japonica, Acanthaster planci, Asterias rubens, Plazaster borealis, Ophiothrix spiculata, Strongylocentrotus purpuratus, Lytechinus variegatus, and Apostichopus japonicus) are based on metazoan, while the background branches (A. japonicus, P. parvimensis, S. Purpuratus, L. variegatus, A. planci, and A. japonica) are based on Echinozoa. The different ranges of background branches and the some different species lead to the difference of positive selected analysis in both studies by the dN/dS accumulation. The similar issue have found in positive selected analysis for Paelopatides sp. (Liu et al., 2021; Shao et al., 2022).
In the other hand, we found the regulated genes of DNA damage repair in both cold seep C. heheva (MSH4) and hydrothermal vent C. heheva (POLB, FAN1, RFC2, KDM2A, FARSA, SPG7, BRCA1, TTLL9, DCLK1, LDHD, and SIRT4). The DNA damage repair adaptation were found in both studies, that suggested the similar adaptation to deep-sea environment.

The references in here.
Liu, RY, Liu, J, Zhang, HB. Positive selection analysis reveals the deep-sea adaptation of a hadal sea cucumber (Paelopatides sp.) to the Mariana Trench. J Oceanol Limnol 2021; 39(1), 266-281.
Shao G, He T, Mu Y, et al. The genome of a hadal sea cucumber reveals novel adaptive strategies to deep-sea environments. Iscience 2022; 105545.

2. To detect signatures of positive selection associated with hydrothermal vent adaptation, the protein set from the published C. heheva H assembly could have been included in the tests for selection alongside the other 9 species. This would help rule out cold seep vs hydrothermal adaptation, if such signatures exist.

Thank you for your valuable suggestion. Considering the suggestion, we have repeated the positive selection analysis that the protein set of Haima cold seep C. heheva has added to. We found that the positive selection genes are same as before.
According to the positive selection analysis is based on orthologous genes, we need to identify the candidate orthologous genes from protein sets of the two C. heheva and other 9 species in first. In theory, the protein sets of same species often has a high similarity and may provide the same orthologs for positive selection analysis. In order to confirm this view, we searched the orthogroups of the protein sets between hydrothermal vent C. heheva and cold seep C. heheva by OrthoFinder. The overlapped orthogroups (12608) between the two C. heheva cover 100 % of the orthogroups in hydrothermal vent C. heheva (12608) and 95.70 % of the orthogroups in cold seep C. heheva (13175). The candidate orthologs of the two C. heheva for positive selection analysis is provided from the overlapped orthogroups (12608), which result to as the same positive selection genes after the added protein set of cold seep C. heheva added. In addition, we found the candidate orthologs of hydrothermal vent C. heheva completely overlap whether the cold deep C. heheva was added or not. Therefore, the gene changes of cold seep vs hydrothermal vent adaptation is not including in those orthologs, and can not affected results of positive selection analysis in this study.

3. I have a similar concern with the gene family expansion analyses. Again, the overlap between the results in this study and Zhang are not discussed, yet this would be a straight forward way to validate the results. Given how distantly related C heheva is to the other species in the comparison, there would almost certainly be a number of shared expanded families in the C. heheva K (this study) and C. heheva H (Zhang) genomes, along with some population-specific adaptations.

Thank you for the valuable suggestion. According to gene-family analysis, we performed gene-family

analysis based on the phylogenetic tree of 10 metazoan species, while Zhang et al. (2022) performed that of 7 echinoderms. In theory, the different species included in gene-family analysis is the main that inevitably affect the results. In addition, we have searched our expanded gene families, which significantly enriched in the processes of membrane fluidity maintaining, iron homeostasis regulation, and DNA damage repair, then compared with the expanded families in C. heheva H (Zhang et al., 2022), the shared expanded gene families was found in DNA damage repair as HTH psq-type domain and HTH CENPB-type domain of PTHR19303 (in our study) vs transposon of PTHR19303 (in Zhang's study) . As we known, the DNA damage repair adaptation is broadly-speaking in various fauna in the deep sea.

4. The BUSCO completeness score of the entire genome is 92.4%, yet the BUSCO completeness score of the annotations (protein mode I assume) is reported at 95%. Given this difference is not modest, it is unclear to me how one can find more single-copy orthologs in the annotations of a genome rather than the genome itself. In theory, everything in a protein set derived from the genome would be in the genome itself. Typically it is the other way around - more BUSCOs identified across the entire genome, and a subset of these are annotated. To me this suggests a discrepancy is how the BUSCOs were searched (was Augustus optimized in genome mode?) or a versioning issue. This difference should be addressed.

Thank you for pointing out this issue. First of all, we have to update the BUSCO's completeness from 92.4 % (BUSCO version 4.1.4) to 94.5 % (BUSCO version 5.4.6) according to editor's requirement. The BUSCO results have uploaded to GigaDB as required. For the BUSCO's results, the completeness of the assembly was evaluated using BUSCO (version 5.4.6), and then the completeness of protein set after annotated was also evaluated using BUSCO (version 5.4.6), then the results were provided files that list all sequences and their matched status in BUSCO dataset. In order to obtain high complete protein set for next analyses, we compared the two files and found the intersection. Based on the intersection of protein set, we added the sequences, which missed in the BUSCO evaluated by genome or protein set, to the final protein set for followed analyses. Finally, the final protein set was evaluated using BUSCO (version 5.4.6) again. Thus, the completeness of annotated protein set may slightly higher than completeness of genome.
Here, we list the BUSCO results in short summary of genome and the final protein set as following.
Genome
BUSCO version is: 5.4.6
C:94.5%[S:93.5%,D:1.0%],F:2.8%,M:2.7%,n:954
902 Complete BUSCOs (C)
892 Complete and single-copy BUSCOs (S)
10 Complete and duplicated BUSCOs (D)
27 Fragmented BUSCOs (F)
25 Missing BUSCOs (M)
954 Total BUSCO groups searched
The final protein set
BUSCO version is: 5.4.6
C:95.0%[S:94.2%,D:0.8%],F:1.5%,M:3.5%,n:954
907 Complete BUSCOs (C)
899 Complete and single-copy BUSCOs (S)
8 Complete and duplicated BUSCOs (D)
14 Fragmented BUSCOs (F)
33 Missing BUSCOs (M)
954 Total BUSCO groups searched

5. The methodology underlying the "Unique Gene" set of analyses should be better explained in the results and the methods. Does this mean genes found in C. heheva K but not in C. heheva H? The phrase "unique genes in KEGG annotation and 330 unique genes in GO annotation" on lines 317-318 is unclear. Does this mean KEGG and GO enrichments analyses were run on the sets of unique genes? If so, what was the background set of genes for these enrichment analyses.

Thank you for pointing out this issue and the suggestion. In the former manuscript, we run the unique genes analysis based on KEGG annotation and GO annotation among three species of the Kairei vent C. heheva, the Haima cold seep C. heheva, and shallow water A. japonicus. Considering your comment in the next (6), we have run the unique genes analysis by OrthoVenn3 based on OrthoFinder algorithm, the

section of unique genes in method part and result have been re-written in the manuscript.

6. In the unique genes list, ferritin heavy chain 1 (FTH1) is highlighted. If my understanding of "uniqueness" is correct, this means FTH1 is not found in the published C. heheva H assembly, but is found in the C. heheva K assembly reported here. It is proposed that this gene may play an outsized role in iron processing in C. heheva K. However, a quick blast search of FTH1 identifies this a homolog of this gene in many metazoans (several echinoderms, mammals, among others). So, I am not sure this gene is unique to C. heheva K, but may instead be lost in C. heheva H (or not annotated for technical reasons). Including other echinoderms in the "unique gene" analyses would perhaps provide a better way of accessing species-specific gains in genetic content. I think the Orthofinder results would also provide this information. Overall, the methods and interpretation of the unique genes results need revising.

We gratefully appreciate for your careful check and your valuable suggestion. It is really true as your comment that ferritin, consists of heavy-chain subunits (FTH1) and light-chain subunits (FTL), is a homolog in many metazoans. Indeed, the loss of FTH1 in other two species may result from annotation methods and genome completeness.
In former manuscript, the protein sets of the three species (C. heheva K, C. heheva H, and A. japonicus) was annotated to database of KEGG and GO followed the same protocol. Then, the relative unique genes was performed by Venn analysis and based on results of KEGG annotation and GO annotation. The gene FTH1 was found in the subset of C. heheva K, that means FTH1 do not shared to the other two species and become the relative unique gene of C. heheva K. FTH1 has an function on iron-rich detoxifcation and iron homeostasis, which is great conform to high content of iron in Kairei vent.
Considering the suggestion of OrthoFinder that provide species-specific genes, we have performed orthologous clusters analysis by OrthoVenn3 based on OrthoFinder algorithm among the protein sets of 6 echinoderms (A. japonica, A. planci, O. spiculata, S. purpuratus, and C. heheva of the Kairei vent and Haima cold seep) .
The section of unique genes in method part and result have been re-written in the manuscript.

Minor:

1. Line 20: "scaffold N50"
2. Line 20-21: BUSCO completeness score
3. Line 22: Instead of "natural chromosome" another way of phrasing could be "19 large scaffolds predicted to represent each of the species' chromosomes"
4. Line 23: "Species-specific" genes instead of "unique" genes? Unique is ambiguous here
5. Line 136-137: After purging dups, the genome is still contig-level. Scaffolds are generated from HiC data later
6. Line 141: Juicebox*
7. Were any scaffolds manually repaired in JuiceBox. If so, this should be described
8. Line 190: JCVI should be expanded here for the first mention
9. Line 203: "tests for positive selection" rather than "positively selected analysis"
10. Line 211: "evolving under positive selection" rather than "positively selected"
11. Line 233: How does 92.4% compare to other quality sea cucumber or echinoderm genomes? Is this high?

We gratefully appreciate for pointing out these issues and your valuable suggestion in above. We have made the corrections according to the above comments in the manuscript. For the genome completeness, we have to update the value from 92.4 % (BUSCO version 4.1.4) to 94.5 % (BUSCO version 5.4.6) according to editor's requirement of using the latest BUSCO software (Version 5.2+). The 94.5 % (BUSCO version 5.4.6) completeness of the Kairei vent C. heheva is higher than that 89.6 % (BUSCO version 4.0.5) completeness of the Haima cold seep C. heheva.

Reviewer #3: This is a high-quality genome assembly of Chiridota heheva, an apodid sea cucumber. It is the first chromosome-level genome assembly of an apodid sea cucumber. The specimen comes from a very inaccessible part of the ocean and this fact alone makes this resource incredibly valuable. The genomic work is high quality. This resource will be invaluable to a wide range of researchers including those studying other sea cucumbers or echinoderms more widely, those studying deep-sea organisms, those

studying organisms associated with chemosynthetic environments. The following are a few general thoughts about the paper:

1. All command lines run as part of these analyses should be provided. Providing command lines removes any ambiguity as to how the analyses were carried out. See the docx file in the supplementary materials of Zhou et al. 2017 (https://doi.org/10.1093/molbev/msx302) for an excellent example of how to include command lines.

Thank you for the suggestion. We have checked the docx file in the supplementary materials of Zhou et al. 2017 (https://doi.org/10.1093/molbev/msx302), and have provided the command of analyses in the a word file as supplementary, please check the supplementary files.

2. The manuscript would be greatly improved if it included a resources table, which includes key reagents, software versions, etc. A good example of one is in the following publication just after the References section: https://www.cell.com/cell/pdf/S0092-8674(23)00107-1.pdf (this would not be a replacement for methods section)

Thank you for the suggestion. Considering to the suggestion, we have read the resources table of Shao et al. 2023 (https://www.cell.com/cell/pdf/S0092-8674(23)00107-1.pdf), the key reagent and resource of our study have added in a supplementary table, please the supplementary files.

3. The current version of the manuscript does not directly address the major differences between the cold-seep C. heheva and and vent C. heheva. The assemblies are >300 megabases different in size. There are >300 genes present in vent C. heheva that are absent from the cold-seep C. heheva assembly (LINE 317). Is this an issue with the assembly or are these separate species? Is there other evidence that can be brought to address this issue (e.g., nucleotide variability)?

Thank you for pointing out this issue.
Indeed, the differences between the cold-seep C. heheva and and vent C. heheva should be considered in the further study. In fact, we have collected some chiridotid holothurians that distributed in many ecosystems, the differences between the chiridotids is the essential parts for environment adaptation on chiridotids. However, compared to the C. heheva genome of Zhang et al. (2022) reported, our C. heheva genome was collected from hydrothermal vent while Zhang’s genome was colected from cold seep, our assembly level is chromosome while Zhang’s is contig, our genome completeness is high as 94.50 % (BUSCO) while Zhang’s completeness is 89.60 % (BUSCO). It is worth to report the chromosome-level genome assembly of hydrothermal vent C. heheva, and provide the high quality of chromosome-level genome in hydrothermal vent for studinng the adaptation in deep-sea extreme environment.
According to the assembly results and genome annotation, the difference of genome sizes is 323 Mb between the Kairei vent C. heheva genome (1.43 Gb) and the Haima cold seep C. heheva genome (1.107 Gb), the difference sizes of repetitive elements is 338 Mb between the of the Kairei vent C. heheva (70.80 %, 1.012 Gb) and of . Based on the repetitive elements, the Kairei vent C. heheva (56.40 %, 624 Mb). That suggested the bigger size of the Kairei vent C. heheva than the Haima cold seep C. heheva may result from the high percentage of the repetitive elements. Moreover, the different completeness of genome assembly also lead to the difference sizes (15 Mb, 338 Mb - 323 Mb) both genomes.

The following are some specific points:

1. LINE 271: "Compared with the other 9 metazoans, 450 gene families were expanded, and 6 were contracted in C. heheva (Fig. 3A)."
-- This is vague as written. The CAFE analyses provide information relative to ancestors and this should be communicated. For example, the 450 gene family expansions and 6 contractions occurred since the last common ancestor of A. japonicus and C. heheva.

Thank you for pointing out this issue. According to the suggestion, we have re-written the part in the revised manuscript (lines 251-253).

2. LINE 306: "involved in DNA damage"
-- More accurate to say the genes are involved in "DNA repair" or "response to DNA damage."

Thank you for your suggestion. According to the suggestion, we have re-written this part in the revised manuscript (line 311).

3. Figure 3 legend should mention that orthologous results are based on CAFE analysis.

Thank you so much for your careful check. According to the suggestion, we have added the orthologous results to Figure 3 legend in the revised manuscript.
