# Supplementary material for: A high-quality chromosomal genome assembly of the sea cucumber Chiridota heheva and its hydrothermal adaptation
Source: Gigascience. 2024 Jan 4;13:giad107. doi: 10.1093/gigascience/giad107 (PMC10764150; doi:10.1093/gigascience/giad107)
Supplement: giad107_Response_to_Reviewer_Comments_Revision_1 [file giad107_response_to_reviewer_comments_revision_1.pdf]

# Author's Response To Reviewer Comments

Response to Reviewers

Reviewer #1: The authors provided more information regarding the analyses of gene family and positively selected genes. However, there are still several problems of the manuscript.

1. There are still many grammatical problems in the revised manuscript. For example:
1) Line 33, "Date Description"
2) Line 178, "Genes under positlve selected"
Please check the grammar of the entire manuscript again.

Thank you so much for your careful check. We have checked the grammar and corrected the grammar in the revised manuscript.
For the example mentioned by the reviewer, we have corrected as follows:
Line 33, the statement "Date Description" was corrected as "Data Description."
Line 178, the statement "Genes under positive selected" was corrected as "Genes under positive selection."

2.Line 158, the authors should specify all the divergence time they used as calibration time in the MCMCtree analysis.

Thank you for pointing out this issue. We have specified the calibration time according per the reviewer's suggestion in the revised manuscript (lines 157-159).

3.Line 212, Zhang et al. (2022) estimated the size of C. heheva genome to be 1.2Gb. And the size of their assembled genome is 1.1Gb, which is closed to the estimated size. However, the authors didn't estimate the genome size of the sequenced individual, and claimed that their assembly is better as their assembly is three quarters larger than Zhang et al's assembly. This is not appropriate.

Thank you for pointing out this issue. On one hand, for the first report of one species' genome, the estimated genome size is often used to determine the coverage depth of Nanopore or PacBio reads. The estimated genome size is also used as the required parameter for WTDBG2 or NextDenovo running of genome assembly. In our study, the basic information of the C. heheva genome has described as Zhang et al. (2022). The sequencing amount and coverage depth of our individual were determined based on genome size as previous report (Zhang et al., 2022). The assembled genome size of our individual is 1.43 Gb, which is also close to the estimated size (1.23 Gb) of the same species (Zhang et al., 2022). The genome assembly of our individual was running by Hifiasm without the parameter of genome size. That is why we did not estimate the genome size of our individual.
On the other hand, to assess the quality of the C. heheva genome assembly, the completeness of the genome assembly was assessed using BUSCO. Complete BUSCOs percentage of C. heheva in the Kairei vent (94.5%) is higher than that in the Haima cold seep (89.6%) (Zhang et al., 2022), which indicate the integrity of our assembled genome is better than Zhang et al.'s (2022). We are very sorry for our incorrect writing. We have re-written this part in the revised manuscript (lines 215-222).

4.Line 214, There are several approaches and softwares (ie. Mercury) to evaluate the quality and completeness of genome assembly (ie. Mercury). However, the author just used the result of BUSCO to evaluate the completeness of genome assembly. It is important to evaluate the quality of the assembly.

BUSCO is the most widely used method for assessing genome assembly based on the conserved metazoan gene set database. We have performed the BUSCO evaluation of our genome assembly and compared the BUSCO results of genome assembly between our individual and Zhang et al. (2022). We have re-written this part in the revised manuscript (lines 215-217).
Considering the reviewer's suggestion, we also assessed genome assembly quality using Inspector, which evaluates assemblies with only third-generation sequencing reads. We have added the Inspector's result in the revised manuscript (lines 218-222) and Table 1. The recommended approach, Merqury, was not

performed here due to the lack of Illumina dataset.

5.Line 240, the authors identified the syntenic blocks between C. heheva and A. japonicus. But they didn't draw any conclusion about this analysis.

Thank you for pointing out this issue. According to the reviewer’s suggestion, we have re-written this part in the revised manuscript (lines 247-258).

6.Line 302, the authors should describe the results of GO and KEGG separately.

Thank you for pointing out this issue. We agree with the reviewer’s suggestion. We have re-written this part to separate the results of GO and KEGG in the revised manuscript (lines 313-318).

Reviewer #2: I thank the authors for their time and detailed revision of their manuscript and addressing the comments. I have a few remaining comments listed here. Overall, I think this genomic resource will be valuable to the broader scientific community. For clarity, my comments reference the line numbers on the version of the manuscript where changes are tracked:

Major Comment:

1.I very respectfully suggest that this manuscript receive another serious round of grammatical corrections from a fluent English speaker as there remain a number of issues, some of which make it difficult to understand the message that the authors are conveying. I think it is important that these language revisions occur prior to publication. I've listed the first three I have come across below as examples:

Line 14: change "is one of the" to "is a"
Line 17: change "environment" to "environments"
Line 25: change "condition" to "conditions"

We apologize for the errors of our manuscript. We have changed "is one of the" to "is a" (line 11), have changed "environment" to "environments" (line 14), and have changed "condition" to "conditions" (line 15). The manuscript has also been double-checked, and the grammar issues we found have been corrected in the revised manuscript by using the track changes mode. We hope that the language level has been substantially improved.

2.Data Availability: I can see the files uploaded to ScienceDB, however, I was unable to access or find any sequencing reads under the provided BioProject ID. Ensuring the accessibility of these data will be important prior to publication.

During the process of creating a data submission, we set the data protection period until December 31, 2023. Before this period, our BioProject's data is available at https://dataview.ncbi.nlm.nih.gov/object/PRJNA934972?reviewer=6lphqpajfogvnfb8laqf4ljajf for reviewers. Please check the reviewer’s link for more information. We have also provided this URL to the publisher to share with reviewers. After this period, the data is automatically converted to open access status and its data files are accessible to all visitors.

3.Other minor comments:
Line 46: Hydrothermal vents do not inhabit animals, animals inhabit hydrothermal vents.
Line 226: "aligned" to "alignment"
Line 231: "evolving" to "putatively evolving".
Line 291: "dot plot"
Line 356: "were contributed" to "may contribute"
Line 694: I think the word "genes" is missing after "unique"

We agree with these suggestions. We have modified “Hydrothermal vents are one of the typical deep-sea chemosynthetically-driven ecosystems that inhabit a wide array of animals and chemosynthetic microbes” to “Hydrothermal vents are one of the typical deep-sea chemosynthetically-driven ecosystems with a wide

array of animals and chemosynthetic microbes” (lines 35-36), have modified “aligned” to “alignments” (line 183), have modified “evolving” to “putatively evolving” (line 189), have modified “dot plotter” to “dot plot” (247), have modified “were contributed” to “may contribute” (line 306), and have modified “unique” to “unique genes” of Figure 7 legend (line 624).
We will be happy to edit the text further, based on helpful comments from the reviewers.

Reviewer #3: 1. The commands file (Supplementary file S1) is adequate although it is impossible to repeat the codeml analysis without details provided in the control file ("paml4.9j/bin/codeml $name_ctl #prepare tree file and ctl file before codeml"). Please provide the contents of the control file in this document.
The Resources file looks solid.

Thank you for pointing out this issue. According to the reviewer’s suggestion, we have added the contents of the preparations of tree and ctl files in “Supplementary text S1 Commands for analyses_revised2”.

2.In comment 3, I inquired about the difference between the genomes of C. heheva K and C. heheva H, in terms of size (>300MB difference in size) and gene number (>300 difference in number of genes). The authors address the difference in size. The authors do not address the difference in gene number.

We are very sorry for our negligence. Thanks for giving us the opportunity to respond to the comments. In the new version, we have re-written the parts of “Unique genes” in Methods and “Unique genes evolution” in Results after have carefully considered all comments from the reviewers. In this part, orthologous clusters were identified using OrthoVenn3 across a range of six echinoderms (A. japonica, A. planci, O. spiculata, S. purpuratus, the Haima cold seep C. heheva, and the Kairei vent C. heheva). The results of OrthoVenn3 presented orthologous clusters in each species, as well as unique and shared homologous gene clusters among species. Those unique genes of the Kairei vent C. heheva were included in the species specific clusters of C. heheva. We hope that this part is clearer now and thanks again for handling our manuscript.

3.I suggested an example of a sentence of how the CAFE analysis should be discussed relative to how it was discussed in the main manuscript. The authors incorporated this sentence into the manuscript. This is fine, except the sentence needs clarification. It currently reads: "Compared with the other 9 metazoans, the 450 gene families expansions and 6 contractions occurred since the last common ancestor of A. japonicus and C. heheva (Fig. 3A)." The sentence as it is written is ambiguous and the authors introduced a grammar mistake by making "family" plural. The following would make it more clear: "In this analysis, 450 gene family expansions and 6 contractions were observed in the C. heheva lineage, since the last common ancestor of A. japonicus and C. heheva (Fig. 3A)."

We apologize for the grammar mistakes of our manuscript. According to the reviewer’s suggestion, we have re-written this sentence in the revised manuscript (lines 261-263). We have now worked on both language and readability and have also involved English speakers for language corrections. We really hope that the flow and language level have been substantially improved.
