# Supplementary material for: A high-quality chromosomal genome assembly of the sea cucumber Chiridota heheva and its hydrothermal adaptation
Source: Gigascience. 2024 Jan 4;13:giad107. doi: 10.1093/gigascience/giad107 (PMC10764150; doi:10.1093/gigascience/giad107)
Supplement: giad107_Reviewer_1_Report_Original_Submission — Muhua Wang -- 3/22/2023 Reviewed [file giad107_reviewer_1_report_original_submission.pdf]

Reviewer Report

Title: A high-quality chromosomal genome assembly of the sea cucumber Chiridota heheva and its hydrothermal adaptation

Version: Original Submission **Date:** 3/22/2023

Reviewer name: Muhua Wang

Reviewer Comments to Author:

Pu et al. reported a chromosome-level genome assembly of C. heheva collected from hydrothermal vent. The authors dissected the genetic basis of adaptation in vent-dwelling C. heheva by performing protein family expansion/contraction and positively selected gene analysis. Here are my comments of the manuscript:1. There are many grammatical problems in the manuscript. These include errors in tenses, verb conjugation, and definite and indefinite articles. Please check the grammar of the entire manuscript again.2. Line 279, I am not convinced that expansion of protein family related to lipid metabolism suggested vent-dwelling C. heheva had more fatty acid in the membrane. Fatty acid is just one kind of lipid.3. Line 282, specify what are toxic iron and safe iron status.4. Line 283, specify what are "These expanded gene families".5. Line 289, What is the relationship between DNA-protected abilities and DNA replication, recombination, DNA-associated protein? I am not convinced that expansion of protein family related to DNA replication implied vent-dwelling C. heheva had better ability in DNA protected ability.6. Line 301, how did "deep-sea fauna obtained PSGs through adaptive evolution"? Gene were positively selected during adaptive evolution.7. Line 307, explain how genes "mainly induced by high hydrostatic pressure".8. Line 323, terms cannot be enriched to adapt the iron-rich environment. Only organisms can adapt to a specific environment.9. Line 330, explain the relationship between the unique genes and DNA repair. I don't think that the unique genes identified in the study are related to DNA repair.

Level of Interest

Please indicate how interesting you found the manuscript: Choose an item.

Quality of Written English

Please indicate the quality of language in the manuscript: Choose an item.

Declaration of Competing Interests

Please complete a declaration of competing interests, considering the following questions:

- Have you in the past five years received reimbursements, fees, funding, or salary from an organisation that may in any way gain or lose financially from the publication of this manuscript, either now or in the future?

- Do you hold any stocks or shares in an organisation that may in any way gain or lose financially from the publication of this manuscript, either now or in the future?
- Do you hold or are you currently applying for any patents relating to the content of the manuscript?
- Have you received reimbursements, fees, funding, or salary from an organization that holds or has applied for patents relating to the content of the manuscript?
- Do you have any other financial competing interests?
- Do you have any non-financial competing interests in relation to this paper?

If you can answer no to all of the above, write 'I declare that I have no competing interests' below. If your reply is yes to any, please give details below.

I declare that I have no competing interests

I agree to the open peer review policy of the journal. I understand that my name will be included on my report to the authors and, if the manuscript is accepted for publication, my named report including any attachments I upload will be posted on the website along with the authors' responses. I agree for my report to be made available under an Open Access Creative Commons CC-BY license (http://creativecommons.org/licenses/by/4.0/). I understand that any comments which I do not wish to be included in my named report can be included as confidential comments to the editors, which will not be published.

Choose an item.

To further support our reviewers, we have joined with Publons, where you can gain additional credit to further highlight your hard work (see: https://publons.com/journal/530/gigascience). On publication of this paper, your review will be automatically added to Publons, you can then choose whether or not to claim your Publons credit. I understand this statement.

Yes Choose an item.
