# Supplementary material for: A high-quality chromosomal genome assembly of the sea cucumber Chiridota heheva and its hydrothermal adaptation
Source: Gigascience. 2024 Jan 4;13:giad107. doi: 10.1093/gigascience/giad107 (PMC10764150; doi:10.1093/gigascience/giad107)
Supplement: giad107_Reviewer_1_Report_Revision_1 — Muhua Wang -- 7/10/2023 Reviewed [file giad107_reviewer_1_report_revision_1.pdf]

Reviewer Report

Title: A high-quality chromosomal genome assembly of the sea cucumber Chiridota heheva and its hydrothermal adaptation

Version: Revision 1 **Date:** 7/10/2023

Reviewer name: Muhua Wang

Reviewer Comments to Author:

The authors provided more information regarding the analyses of gene family and positively selected genes. However, there are still several problems of the manuscript.1. There are still many grammatical problems in the revised manuscript. For example:1) Line 33, "Date Description"2) Line 178, "Genes under positlve selected"Please check the grammar of the entire manuscript again.2. Line 158, the authors should specify all the divergence time they used as calibration time in the MCMCtree analysis.3. Line 212, Zhang et al. (2022) estimated the size of C. heheva genome to be 1.2Gb. And the size of their assembled genome is 1.1Gb, which is closed to the estimated size. However, the authors didn't estimate the genome size of the sequenced individual, and claimed that their assembly is better as their assembly is three quarters larger than Zhang et al's assembly. This is not appropriate.4. Line 214, There are several approaches and softwares (ie. Mercury) to evaluate the quality and completeness of genome assembly (ie. Mercury). However, the author just used the result of BUSCO to evaluate the completeness of genome assembly. It is important to evaluate the quality of the assembly.5. Line 240, the authors identified the syntenic blocks between C. heheva and A. japonicus. But they didn't draw any conclusion about this analysis.6. Line 302, the authors should describe the results of GO and KEGG separately.

Level of Interest

Please indicate how interesting you found the manuscript: Choose an item.

Quality of Written English

Please indicate the quality of language in the manuscript: Choose an item.

Declaration of Competing Interests

Please complete a declaration of competing interests, considering the following questions:

- Have you in the past five years received reimbursements, fees, funding, or salary from an organisation that may in any way gain or lose financially from the publication of this manuscript, either now or in the future?
- Do you hold any stocks or shares in an organisation that may in any way gain or lose financially from the publication of this manuscript, either now or in the future?

- Do you hold or are you currently applying for any patents relating to the content of the manuscript?
- Have you received reimbursements, fees, funding, or salary from an organization that holds or has applied for patents relating to the content of the manuscript?
- Do you have any other financial competing interests?
- Do you have any non-financial competing interests in relation to this paper?

If you can answer no to all of the above, write 'I declare that I have no competing interests' below. If your reply is yes to any, please give details below.

I declare that I have no competing interests

I agree to the open peer review policy of the journal. I understand that my name will be included on my report to the authors and, if the manuscript is accepted for publication, my named report including any attachments I upload will be posted on the website along with the authors' responses. I agree for my report to be made available under an Open Access Creative Commons CC-BY license (http://creativecommons.org/licenses/by/4.0/). I understand that any comments which I do not wish to be included in my named report can be included as confidential comments to the editors, which will not be published.

Choose an item.

To further support our reviewers, we have joined with Publons, where you can gain additional credit to further highlight your hard work (see: https://publons.com/journal/530/gigascience). On publication of this paper, your review will be automatically added to Publons, you can then choose whether or not to claim your Publons credit. I understand this statement.

Yes Choose an item.
