# Supplementary material for: A high-quality chromosomal genome assembly of the sea cucumber Chiridota heheva and its hydrothermal adaptation
Source: Gigascience. 2024 Jan 4;13:giad107. doi: 10.1093/gigascience/giad107 (PMC10764150; doi:10.1093/gigascience/giad107)
Supplement: giad107_Reviewer_2_Report_Original_Submission — Phillip Davidson -- 3/27/2023 Reviewed [file giad107_reviewer_2_report_original_submission.pdf]

Reviewer Report

Title: A high-quality chromosomal genome assembly of the sea cucumber Chiridota heheva and its hydrothermal adaptation

Version: Original Submission **Date:** 3/27/2023

Reviewer name: Phillip Davidson

Reviewer Comments to Author:

In this manuscript, Pu et al present a chromosome-level genome assembly of the holothurian Chiridota heheva. The assembly and annotation process was carried out with an up-to-date sequencing and methodological strategy and has resulted in a high quality genome assembly of this species, such that two assemblies of these species are now available. This assembly has the nice addition of HiC scaffolding which has resulted in chromosome-length scaffolds. This work is accompanied by gene family expansion analyses and tests for positive selection in coding sequence to identify putatively adaptive changes in genes associated with living in hydrothermal environments.For clarity, I've organized my comments into two sections: 1) Rational and Significance and 2) Methods and Results. These comments each have major and minor components. Overall, while the assembly itself is nice and now available at a chromosome-level, I think the description of the rationale of this study and how it is distinguished from the already published assembly is needed. As written, the ability to discern hydrothermal adaptations from a more general suite of adaptations associated with the deep sea is unclear. In addition, more methodological detail and revision is needed with each of the functional genomic analyses, and reconciling these results with those already published will be helpful in revising their interpretation.Rational and SignificanceMajor Comments1. The assembly reported in this manuscript represents the second assembly for this species. The rationale for generating this assembly is stated to be that the individual C. heheva was collected from population inhabiting the Kairei hydrothermal vent (herein C. heheva K) whereas the earlier published version of this species genome was from an individual collected from a population inhabiting a different deep sea environment, a cold seep (herein C. heheva H: Zhang et al, 2022 Comms Bio). However, C. heheva is a cosmopolitan holothurian found in several harsh deep sea environments, so it is not clear whether any genetic-signatures of adaptations detected in this assembly are truly associated with hydrothermal vent adaptation or not. Given these individuals (K and H) represent the same species, presently it is difficult to say whether the genome assembly of single individual is an appropriate measure of local adaption to hydrothermal vent habitats unless these populations been isolated for a very long amount of time (which should be reported). Otherwise, population genetic data would be needed in this case. It is possible that this species is instead adapted to harsh deep sea environments broadly-sepaking, so the utility of comparisons between single individuals is difficult to interpret. Given a genome assembly of this species is already published, more information justifying the rational of this study is needed in this regard.2. Along the same lines, I think more effort is needed to distinguish this study from the previously published one of the same species in terms of the manuscript's writing. The phrasing of some of the writing is a little too close for comfort to what is already published. For example:a. Introduction: Lines 86-90: "Chiridota

heheva, a cosmopolitan Chiridotidae holothurian, has been found in hydrothermal vents, cold seeps, and other organic falls [25-26]. C. heheva may be an ideal model for studying how marine fauna have adapted to extreme deep-sea chemosynthetic environments." Vs Zhang: "Chiridota heheva Pawson & Vance, 2004 (Apodida: Chiridotidae) is one of the few echinoderms that occupies all three types of chemosynthetic ecosystems (hydrothermal vent, cold seep, and organic fall)… "The cosmopolitan distribution and special lifestyle of C. heheva make it an ideal model to study adaptation to deep-sea reducing environments in nonsymbiotic animals."b. the titles are similar: "The chromosome-level genome of Chiridota heheva (Holothuroidea, Apodida,Chiridotidae) provides insights into adaptation to the hydrothermal environment" vs Zhang: "The genome of an apodid holothuroid (Chiridota heheva) provides insights into its adaptation to a deep-sea reducing environment"3. This study replicates many of the same functional genomic analyses as Zhang et al (gene family expansion, tests for positive selection) but no overlap in results are reported. More on this in the next section below, but this represents a unique opportunity to compare two independent studies of the same species to validate predictions associated with deep sea adaptation. However, these cross-study comparisons are not mentioned even though they would be an effective way of distinguishing cold seep vs hydrothermal adaptation, should they exist.Minor1. The second paragraph of the introduction is quite lengthy as it summarizes many adaptations associated with deep sea habitats. These species and adaptations list could probably be shortened, particularly for a Data Note type of manuscript.Results and MethodsMajor:1. Tests were positive selection occurred at the species level as C. heheva was compared to several other metazoans. A very similar analysis was carried out in Zhang et al (also C heheva) yet there appears to be little to no overlap is genes with evidence of positive selection detected in both studies. I realize these animals came from different habitats, but shared species-level signatures of positive selection would be expected here, particularly in regard to deep sea adaption, broadly speaking. This large discrepancy is concerning and needs justification or revision.2. To detect signatures of positive selection associated with hydrothermal vent adaptation, the protein set from the published C. heheva H assembly could have been included in the tests for selection alongside the other 9 species. This would help rule out cold seep vs hydrothermal adaptation, if such signatures exist.3. I have a similar concern with the gene family expansion analyses. Again, the overlap between the results in this study and Zhang are not discussed, yet this would be a straight forward way to validate the results. Given how distantly related C heheva is to the other species in the comparison, there would almost certainly be a number of shared expanded families in the C. heheva K (this study) and C. heheva H (Zhang) genomes, along with some population-specific adaptations.4. The BUSCO completeness score of the entire genome is 92.4%, yet the BUSCO completeness score of the annotations (protein mode I assume) is reported at 95%. Given this difference is not modest, it is unclear to me how one can find more single-copy orthologs in the annotations of a genome rather than the genome itself. In theory, everything in a protein set derived from the genome would be in the genome itself. Typically it is the other way around - more BUSCOs identified across the entire genome, and a subset of these are annotated. To me this suggests a discrepancy is how the BUSCOs were searched (was Augustus optimized in genome mode?) or a versioning issue. This difference should be addressed.5. The methodology underlying the "Unique Gene" set of analyses should be better explained in the results and the methods. Does this mean genes found in C. heheva K but not in C. heheva H? The phrase "unique genes in KEGG annotation and 330 unique genes in GO annotation" on lines 317-318 is unclear. Does this mean KEGG and GO enrichments

analyses were run on the sets of unique genes? If so, what was the background set of genes for these enrichment analyses.6. In the unique genes list, ferritin heavy chain 1 (FTH1) is highlighted. If my understanding of "uniqueness" is correct, this means FTH1 is not found in the published C. heheva H assembly, but is found in the C. heheva K assembly reported here. It is proposed that this gene may play an outsized role in iron processing in C. heheva K. However, a quick blast search of FTH1 identifies this a homolog of this gene in many metazoans (several echinoderms, mammals, among others). So, I am not sure this gene is unique to C. heheva K, but may instead be lost in C. heheva H (or not annotated for technical reasons). Including other echinoderms in the "unique gene" analyses would perhaps provide a better way of accessing species-specific gains in genetic content. I think the Orthofinder results would also provide this information. Overall, the methods and interpretation of the unique genes results need revising.Minor:1. Line 20: "scaffold N50"2. Line 20-21: BUSCO completeness score3. Line 22: Instead of "natural chromosome" another way of phrasing could be "19 large scaffolds predicted to represent each of the species' chromosomes"4. Line 23: "Species-specific" genes instead of "unique" genes? Unique is ambiguous here5. Line 136-137: After purging dups, the genome is still contig-level. Scaffolds are generated from HiC data later6. Line 141: Juicebox*7. Were any scaffolds manually repaired in JuiceBox. If so, this should be described8. Line 190: JCVI should be expanded here for the first mention9. Line 203: "tests for positive selection" rather than "positively selected analysis"10. Line 211: "evolving under positive selection" rather than "positively selected"11. Line 233: How does 92.4% compare to other quality sea cucumber or echinoderm genomes? Is this high?

Level of Interest

Please indicate how interesting you found the manuscript: Choose an item.

Quality of Written English

Please indicate the quality of language in the manuscript: Choose an item.

Declaration of Competing Interests

Please complete a declaration of competing interests, considering the following questions:

- Have you in the past five years received reimbursements, fees, funding, or salary from an organisation that may in any way gain or lose financially from the publication of this manuscript, either now or in the future?
- Do you hold any stocks or shares in an organisation that may in any way gain or lose financially from the publication of this manuscript, either now or in the future?
- Do you hold or are you currently applying for any patents relating to the content of the manuscript?
- Have you received reimbursements, fees, funding, or salary from an organization that holds or has applied for patents relating to the content of the manuscript?
- Do you have any other financial competing interests?

- Do you have any non-financial competing interests in relation to this paper?

If you can answer no to all of the above, write 'I declare that I have no competing interests' below. If your reply is yes to any, please give details below.

I declare that I have no competing interests

I agree to the open peer review policy of the journal. I understand that my name will be included on my report to the authors and, if the manuscript is accepted for publication, my named report including any attachments I upload will be posted on the website along with the authors' responses. I agree for my report to be made available under an Open Access Creative Commons CC-BY license (http://creativecommons.org/licenses/by/4.0/). I understand that any comments which I do not wish to be included in my named report can be included as confidential comments to the editors, which will not be published.

Choose an item.

To further support our reviewers, we have joined with Publons, where you can gain additional credit to further highlight your hard work (see: https://publons.com/journal/530/gigascience). On publication of this paper, your review will be automatically added to Publons, you can then choose whether or not to claim your Publons credit. I understand this statement.

Yes Choose an item.
