# Supplementary material for: A high-quality chromosomal genome assembly of the sea cucumber Chiridota heheva and its hydrothermal adaptation
Source: Gigascience. 2024 Jan 4;13:giad107. doi: 10.1093/gigascience/giad107 (PMC10764150; doi:10.1093/gigascience/giad107)
Supplement: giad107_Reviewer_2_Report_Revision_1 — Phillip Davidson -- 7/18/2023 Reviewed [file giad107_reviewer_2_report_revision_1.pdf]

Reviewer Report

Title: A high-quality chromosomal genome assembly of the sea cucumber Chiridota heheva and its hydrothermal adaptation

Version: Revision 1 **Date:** 7/18/2023

Reviewer name: Phillip Davidson

Reviewer Comments to Author:

I thank the authors for their time and detailed revision of their manuscript and addressing the comments. I have a few remaining comments listed here. Overall, I think this genomic resource will be valuable to the broader scientific community. For clarity, my comments reference the line numbers on the version of the manuscript where changes are tracked:Major Comment:I very respectfully suggest that this manuscript receive another serious round of grammatical corrections from a fluent English speaker as there remain a number of issues, some of which make it difficult to understand the message that the authors are conveying. I think it is important that these language revisions occur prior to publication. I've listed the first three I have come across below as examples:Line 14: change "is one of the" to "is a"Line 17: change "environment" to "environments"Line 25: change "condition" to "conditions"Data Availability: I can see the files uploaded to ScienceDB, however, I was unable to access or find any sequencing reads under the provided BioProject ID. Ensuring the accessibility of these data will be important prior to publication.Other minor comments:Line 46: Hydrothermal vents do not inhabit animals, animals inhabit hydrothermal vents.Line 226: "aligned" to "alignment"Line 231: "evolving" to "putatively evolving".Line 291: "dot plot"Line 356: "were contributed" to "may contribute"Line 694: I think the word "genes" is missing after "unique"

Level of Interest

Please indicate how interesting you found the manuscript: Choose an item.

Quality of Written English

Please indicate the quality of language in the manuscript: Choose an item.

Declaration of Competing Interests

Please complete a declaration of competing interests, considering the following questions:

- Have you in the past five years received reimbursements, fees, funding, or salary from an organisation that may in any way gain or lose financially from the publication of this manuscript, either now or in the future?
- Do you hold any stocks or shares in an organisation that may in any way gain or lose financially from the publication of this manuscript, either now or in the future?

- Do you hold or are you currently applying for any patents relating to the content of the manuscript?
- Have you received reimbursements, fees, funding, or salary from an organization that holds or has applied for patents relating to the content of the manuscript?
- Do you have any other financial competing interests?
- Do you have any non-financial competing interests in relation to this paper?

If you can answer no to all of the above, write 'I declare that I have no competing interests' below. If your reply is yes to any, please give details below.

I declare that I have no competing interests.

I agree to the open peer review policy of the journal. I understand that my name will be included on my report to the authors and, if the manuscript is accepted for publication, my named report including any attachments I upload will be posted on the website along with the authors' responses. I agree for my report to be made available under an Open Access Creative Commons CC-BY license (http://creativecommons.org/licenses/by/4.0/). I understand that any comments which I do not wish to be included in my named report can be included as confidential comments to the editors, which will not be published.

Choose an item.

To further support our reviewers, we have joined with Publons, where you can gain additional credit to further highlight your hard work (see: https://publons.com/journal/530/gigascience). On publication of this paper, your review will be automatically added to Publons, you can then choose whether or not to claim your Publons credit. I understand this statement.

Yes Choose an item.
