# Supplementary material for: A high-quality chromosomal genome assembly of the sea cucumber Chiridota heheva and its hydrothermal adaptation
Source: Gigascience. 2024 Jan 4;13:giad107. doi: 10.1093/gigascience/giad107 (PMC10764150; doi:10.1093/gigascience/giad107)
Supplement: giad107_Reviewer_3_Report_Original_Submission — Joseph F. Ryan -- 4/5/2023 Reviewed [file giad107_reviewer_3_report_original_submission.pdf]

Reviewer Report

Title: A high-quality chromosomal genome assembly of the sea cucumber Chiridota heheva and its hydrothermal adaptation

Version: Original Submission **Date:** 4/5/2023

Reviewer name: Joseph F. Ryan

Reviewer Comments to Author:

This is a high-quality genome assembly of Chiridota heheva, an apodid sea cucumber. It is the first chromosome-level genome assembly of an apodid sea cucumber. The specimen comes from a very inaccessible part of the ocean and this fact alone makes this resource incredibly valuable. The genomic work is high quality. This resource will be invaluable to a wide range of researchers including those studying other sea cucumbers or echinoderms more widely, those studying deep-sea organisms, those studying organisms associated with chemosynthetic environments. The following are a few general thoughts about the paper:1. All command lines run as part of these analyses should be provided. Providing command lines removes any ambiguity as to how the analyses were carried out. See the docx file in the supplementary materials of Zhou et al. 2017 (https://doi.org/10.1093/molbev/msx302) for an excellent example of how to include command lines.2. The manuscript would be greatly improved if it included a resources table, which includes key reagents, software versions, etc. A good example of one is in the following publication just after the References section: https://www.cell.com/cell/pdf/S0092-8674(23)00107-1.pdf (this would not be a replacement for methods section)3. The current version of the manuscript does not directly address the major differences between the cold-seep C. heheva and and vent C. heheva. The assemblies are >300 megabases different in size. There are >300 genes present in vent C. heheva that are absent from the cold-seep C. heheva assembly (LINE 317). Is this an issue with the assembly or are these separate species? Is there other evidence that can be brought to address this issue (e.g., nucleotide variability)?The following are some specific points:1. LINE 271: "Compared with the other 9 metazoans, 450 gene families were expanded, and 6 were contracted in C. heheva (Fig. 3A)."-- This is vague as written. The CAFE analyses provide information relative to ancestors and this should be communicated. For example, the 450 gene family expansions and 6 contractions occurred since the last common ancestor of A. japonicus and C. heheva.2. LINE 306: "involved in DNA damage"-- More accurate to say the genes are involved in "DNA repair" or "response to DNA damage."3. Figure 3 legend should mention that orthologous results are based on CAFE analysis.

Level of Interest

Please indicate how interesting you found the manuscript: Choose an item.

Quality of Written English

Please indicate the quality of language in the manuscript: Choose an item.

Declaration of Competing Interests

Please complete a declaration of competing interests, considering the following questions:

- Have you in the past five years received reimbursements, fees, funding, or salary from an organisation that may in any way gain or lose financially from the publication of this manuscript, either now or in the future?
- Do you hold any stocks or shares in an organisation that may in any way gain or lose financially from the publication of this manuscript, either now or in the future?
- Do you hold or are you currently applying for any patents relating to the content of the manuscript?
- Have you received reimbursements, fees, funding, or salary from an organization that holds or has applied for patents relating to the content of the manuscript?
- Do you have any other financial competing interests?
- Do you have any non-financial competing interests in relation to this paper?

If you can answer no to all of the above, write 'I declare that I have no competing interests' below. If your reply is yes to any, please give details below.

I declare that I have no competing interests.

I agree to the open peer review policy of the journal. I understand that my name will be included on my report to the authors and, if the manuscript is accepted for publication, my named report including any attachments I upload will be posted on the website along with the authors' responses. I agree for my report to be made available under an Open Access Creative Commons CC-BY license (http://creativecommons.org/licenses/by/4.0/). I understand that any comments which I do not wish to be included in my named report can be included as confidential comments to the editors, which will not be published.

Choose an item.

To further support our reviewers, we have joined with Publons, where you can gain additional credit to further highlight your hard work (see: https://publons.com/journal/530/gigascience). On publication of this paper, your review will be automatically added to Publons, you can then choose whether or not to claim your Publons credit. I understand this statement.

Yes Choose an item.
