# Supplementary material for: A high-quality chromosomal genome assembly of the sea cucumber Chiridota heheva and its hydrothermal adaptation
Source: Gigascience. 2024 Jan 4;13:giad107. doi: 10.1093/gigascience/giad107 (PMC10764150; doi:10.1093/gigascience/giad107)
Supplement: giad107_Reviewer_3_Report_Revision_1 — Joseph F. Ryan -- 7/29/2023 Reviewed [file giad107_reviewer_3_report_revision_1.pdf]

Reviewer Report

Title: A high-quality chromosomal genome assembly of the sea cucumber Chiridota heheva and its hydrothermal adaptation

Version: Revision 1 **Date:** 7/29/2023

Reviewer name: Joseph F. Ryan

Reviewer Comments to Author:

The commands file (Supplementary file S1) is adequate although it is impossible to repeat the codeml analysis without details provided in the control file ("paml4.9j/bin/codeml $name_ctl #prepare tree file and ctl file before codeml"). Please provide the contents of the control file in this document.The Resources file looks solid.In comment 3, I inquired about the difference between the genomes of C. heheva K and C. heheva H, in terms of size (>300MB difference in size) and gene number (>300 difference in number of genes). The authors address the difference in size. The authors do not address the difference in gene number.I suggested an example of a sentence of how the CAFE analysis should be discussed relative to how it was discussed in the main manuscript. The authors incorporated this sentence into the manuscript. This is fine, except the sentence needs clarification. It currently reads: "Compared with the other 9 metazoans, the 450 gene families expansions and 6 contractions occurred since the last common ancestor of A. japonicus and C. heheva (Fig. 3A)." The sentence as it is written is ambiguous and the authors introduced a grammar mistake by making "family" plural. The following would make it more clear: "In this analysis, 450 gene family expansions and 6 contractions were observed in the C. heheva lineage, since the last common ancestor of A. japonicus and C. heheva (Fig. 3A)."

Level of Interest

Please indicate how interesting you found the manuscript: Choose an item.

Quality of Written English

Please indicate the quality of language in the manuscript: Choose an item.

Declaration of Competing Interests

Please complete a declaration of competing interests, considering the following questions:

- Have you in the past five years received reimbursements, fees, funding, or salary from an organisation that may in any way gain or lose financially from the publication of this manuscript, either now or in the future?
- Do you hold any stocks or shares in an organisation that may in any way gain or lose financially from the publication of this manuscript, either now or in the future?

- Do you hold or are you currently applying for any patents relating to the content of the manuscript?
- Have you received reimbursements, fees, funding, or salary from an organization that holds or has applied for patents relating to the content of the manuscript?
- Do you have any other financial competing interests?
- Do you have any non-financial competing interests in relation to this paper?

If you can answer no to all of the above, write 'I declare that I have no competing interests' below. If your reply is yes to any, please give details below.

I declare that I have no competing interests

I agree to the open peer review policy of the journal. I understand that my name will be included on my report to the authors and, if the manuscript is accepted for publication, my named report including any attachments I upload will be posted on the website along with the authors' responses. I agree for my report to be made available under an Open Access Creative Commons CC-BY license (http://creativecommons.org/licenses/by/4.0/). I understand that any comments which I do not wish to be included in my named report can be included as confidential comments to the editors, which will not be published.

Choose an item.

To further support our reviewers, we have joined with Publons, where you can gain additional credit to further highlight your hard work (see: https://publons.com/journal/530/gigascience). On publication of this paper, your review will be automatically added to Publons, you can then choose whether or not to claim your Publons credit. I understand this statement.

Yes Choose an item.
